# A Framework for Classification of Prokaryotic Protein Kinases

**DOI:** 10.1371/journal.pone.0010608

**Published:** 2010-05-26

**Authors:** Nidhi Tyagi, Krishanpal Anamika, Narayanaswamy Srinivasan

**Affiliations:** Molecular Biophysics Unit, Indian Institute of Science, Bangalore, India; Science Commons, United States of America

## Abstract

**Background:**

Overwhelming majority of the Serine/Threonine protein kinases identified by gleaning archaeal and eubacterial genomes could not be classified into any of the well known Hanks and Hunter subfamilies of protein kinases. This is owing to the development of Hanks and Hunter classification scheme based on eukaryotic protein kinases which are highly divergent from their prokaryotic homologues. A large dataset of prokaryotic Serine/Threonine protein kinases recognized from genomes of prokaryotes have been used to develop a classification framework for prokaryotic Ser/Thr protein kinases.

**Methodology/Principal Findings:**

We have used traditional sequence alignment and phylogenetic approaches and clustered the prokaryotic kinases which represent 72 subfamilies with at least 4 members in each. Such a clustering enables classification of prokaryotic Ser/Thr kinases and it can be used as a framework to classify newly identified prokaryotic Ser/Thr kinases. After series of searches in a comprehensive sequence database we recognized that 38 subfamilies of prokaryotic protein kinases are associated to a specific taxonomic level. For example 4, 6 and 3 subfamilies have been identified that are currently specific to phylum proteobacteria, cyanobacteria and actinobacteria respectively. Similarly subfamilies which are specific to an order, sub-order, class, family and genus have also been identified. In addition to these, we also identify organism-diverse subfamilies. Members of these clusters are from organisms of different taxonomic levels, such as archaea, bacteria, eukaryotes and viruses.

**Conclusion/Significance:**

Interestingly, occurrence of several taxonomic level specific subfamilies of prokaryotic kinases contrasts with classification of eukaryotic protein kinases in which most of the popular subfamilies of eukaryotic protein kinases occur diversely in several eukaryotes. Many prokaryotic Ser/Thr kinases exhibit a wide variety of modular organization which indicates a degree of complexity and protein-protein interactions in the signaling pathways in these microbes.

## Introduction

Archaea and eubacteria respond to a wide array of environmental stimuli including alterations in nutrient availability, temperature, osmolarity and host proximity. The primary sensing machinery present in eubacteria and archaea, which senses temperature, light, chemical concentration, viscosity, osmolarity etc, is the Two-component system. Serine/Threonine or Tyrosine phosphorylation by prokaryotic Ser/Thr or Tyrosine protein kinases is also recognized as another signaling mechanism in prokaryotes [1], [2], [3], [4], [5], [6], [7], [8], [9]. The well known signal transduction systems in prokaryotes are: i) the Two-component system [10] also referred as His-Asp phosphorelay system consisting of a sensor protein (histidine kinase) and response regulator [11], [12] ii) the phosphoenolpyruvate transferase system [13] iii) the bacterial Tyrosine kinase system [7] and iv) the eukaryotic Ser/Thr or tyrosine kinase-like system [14]. The main focus of the current study is detailed analysis of prokaryotic Serine/Threonine protein kinases involving clustering on the basis of amino acid sequence similarity of catalytic regions leading to identification of sub-families.

Pkn1 from *Myxoccocus xanthus* (*M. xanthus*) was the first prokaryotic Serine/Threonine protein kinase identified in prokaryotes which is known to autophosphorylate serine when incubated with radiolabelled ATP (Adenosine Tri Phosphate) [15]. Later phosphotransfer to an exogenous protein substrate by prokaryotic Serine/Threonine protein kinase, AfsK from *Streptomyces coelicolor* was demonstrated [16]. Sequencing of eubacterial and archaeal genomes later facilitated identification of prokaryotic Serine/Threonine protein kinases encoded in various eubacterial and archaeal genomes [3], [5], [15], [17], [18], [19], [20], [21], [22], [23], [24] which suggested that phosphorylation-dephosphorylation of proteins on hydroxyl group plays important roles in prokaryotes also. This mechanism of regulation is quite ancient than previously assumed suggesting high complexity of the system. Prokaryotic Serine/Threonine protein kinases identified in eubacteria and archaea exhibit moderate to low sequence similarities to their eukaryotic counterparts. Recently, analysis of 619 prokaryotic genomes revealed presence of Serine/Threonine protein kinases in approximately two-thirds of the prokaryotes analysed [22].

Biological roles of some of the Ser/Thr kinases in prokaryotes have been investigated by laboratory experiments. Several Ser/Thr kinases from prokaryotes have been characterized biochemically which has revealed roles of these kinases in sugar transport [25], adaptation to light [26], flagellin phosphorylation and export [27], aggregation and sporulation [28], cell division and differentiation [29], morphological differentiation, secondary metabolism [30], oxidative stress response [31], sporulation and biofilm formation [32], glucose metabolism and glycogen consumption [33], [34], carbon catabolite repression [35], glucose transport and cell division [36], purine biosynthesis [37]. Ser/Thr kinases also play crucial role in the virulence of pathogenic prokaryotes [19], [38], [39], [40].

Little was known about the structures and functions of the prokaryotic Serine/Threonine protein kinases until the three-dimensional crystal structure of *Mycobacterium tuberculosis* protein, PknB in complex with a nucleotide triphosphate analog was solved [41], [42] which revealed that tertiary structure of PknB has similarity to eukaryotic Ser/Thr and Tyr kinases (STYKs). This study supports a universal activation mechanism of Ser/Thr kinases in prokaryotes and eukaryotes [41], [42]. Recently, crystal structure of one more prokaryotic protein kinase, YihE from *Escherichia coli* [43] has been solved which has significant similarity with choline kinase which shares the fold with eukaryotic STYKs. YihE is a protein of Cpx signaling system of *Escherichia coli* and *Salmonella enterica* which senses extra-cytoplasmic stress, and further, controls expression of factors that allow bacteria to adapt to stress. Functional analysis revealed that this protein kinase is most abundant in stationary phase and is important for long-term cell survival. The autophosphorylation and phosphorylation of protein substrates at Ser/Thr residues *in vitro* by YihE has been observed suggesting that it is a novel Ser/Thr kinase in prokaryotic cells [43].

Database of Kinases in Genomes (KinG) version 1.5 (http://hodgkin.mbu.iisc.ernet.in/~king) contains information on protein kinases encoded in hundreds of genomes and is updated periodically. Current release of KinG contains information on kinases from 47 archaeal and 256 eubacterial organisms. Sequence analysis of these prokaryotic Ser/Thr kinases depicts that these are distantly related to eukaryotic protein kinase superfamily [44]. Apart from KinG, SENTRA [45], [46], [47] which is a manually curated database provides information on signal transduction proteins. SENTRA has information for Two-component histidine kinases and response regulators, Serine/Threonine protein kinases and protein phosphatases, as well as adenylate and diguanylate cyclases and c-di-GMP phosphodiesterases from 202 completely sequenced prokaryotic genomes. However the present work is confined only to Ser/Thr kinases of prokaryotes.

With the advent of genome sequencing projects, several prokaryotic protein kinases have been identified and many more prokaryotic kinases are likely to be identified in the future. From the genome analysis of prokaryotic kinases and from the work described in KinG database, it is clear that many prokaryotic kinases can not be classified into one of the known groups or subfamilies of eukaryotic protein kinases originally defined by Hanks et al [44]. Hence, there is a need for the classification of prokaryotic Ser/Thr protein kinases as there is no classification scheme available for these kinases similar to the classification framework proposed by Hanks et al [44] for eukaryotic protein kinases. In the present analysis, an attempt has been made to develop a classification scheme for prokaryotic Ser/Thr kinases and to study these kinases further for their potential biological roles based on tethered domains.

## Results and Discussion

### Classification of prokaryotic serine/threonine protein kinases

Based upon sequence similarity over the length of kinase domains (File S1), 993 Ser/Thr kinases from 303 prokaryotic genomes have been clustered into 270 clusters (File S2) with minimum sequence identity of 40% within a cluster. The members of these 270 clusters (sub-families) are quite divergent from each other as can be seen from multiple sequence alignment which is presented in File S3. Seventy two clusters out of total 270 clusters, which have four or more members, have been used for the classification of prokaryotic protein kinases into various sub-families based on their common properties. It should be noted that the sole consideration for the classification of these prokaryotic protein kinases into various families is the sequence similarity in the catalytic kinase domain region as this has been proved to be a good indicator in the classification of eukaryotic protein kinases [44].

In this analysis, there are different types of sub-families which have been observed based on common feature/s shared by the members within a cluster:

- Sub-families which show specificity or predominance at taxonomic level
  - Phylum specific/predominant subfamilies
  - Order specific/predominant subfamilies
  - Sub-order specific/predominant subfamilies
  - Class specific/predominant subfamilies
  - Family specific/predominant subfamilies
  - Genus specific/predominant subfamilies
- Subfamilies which show organism diversity

However these initially derived taxonomic specific/predominant clusters are only tentative as they have been derived from a limited dataset of 303 prokaryotic organisms. So, each of these (tentative) taxonomy specific or predominant clusters were probed, using PSI_BLAST, in the Uniref90 sequence database which is a comprehensive collection of protein sequences from diverse organisms. A tentative taxonomy-specific cluster is confirmed only in case the close homologues (indicated by ≥40% sequence identity for catalytic kinase region) from Uniref90 are in the same taxonomic classification as the cluster in question.

Thus, finally, 38 subfamilies of prokaryotic Ser/Thr kinases have been identified which are specific at certain taxonomic level. For example, genus specific cluster contains members and close homologues from a particular genus of the eubacteria or archaea and so on. Table 1 lists these 38 subfamilies. Subfamilies which are organism diverse, share high sequence similarities with homologues from various organisms belonging to different taxonomic levels such as bacteria, eukaryotes, viruses and archaea.

**Table 1:** Brief description of 38 subfamilies of prokaryotic Ser/Thr kinases which show specificity/predominance at certain taxonomic level.

S. No.	Cluster number(s)	Specificity/predominance at taxonomic level
1	6, 23, 38, 63	Phylum Proteobacteria specific
2	3, 4, 25, 29, 44, 59, 62	Phylum Proteobacteria predominant
3	15, 19, 24, 37, 54, 67	Phylum Cyanobacteria specific
4	60	Phylum Cyanobacteria predominant
5	10,14, 26	Phylum Actinobacteria predominant
6	32	Phylum Euryarchaeota predominant
7	5	Class Gammaproteobacteria specific
8	8, 27, 34, 43, 46	Order Actinomycetales specific
9	22	Order Actinomycetales predominant
10	49	Order Chlamydiales specific
11	31, 72	Suborder Cystobacterineae specific
12	28	Family Thermoproteaceae specific
13	7	Genus Sulfolobus specific
14	45	Genus Bacillus and Geobacillus specific
15	52, 56	Genus Metallosphaera and Sulfolobus specific
16	48	Genus Chlamydia and Chlamydophila specific

**Figure 1:**
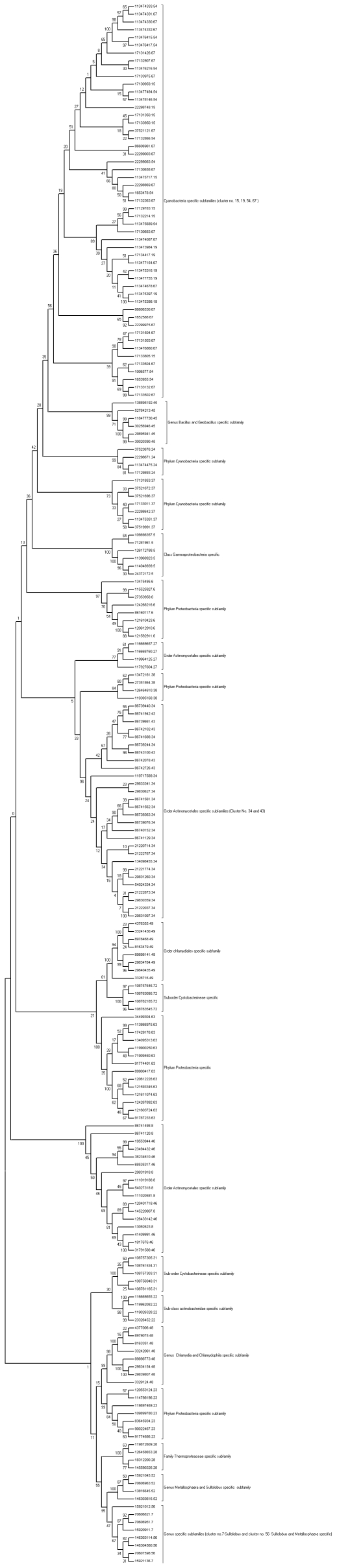
Dendrogram representation of prokaryotic protein kinase catalytic domains which are specific to certain taxonomic levels. Bootstrap values are provided at the main branches. Each protein kinase is represented as GI number and serial number of that particular cluster after dot. Taxonomic specificity of cluster is written at right hand side. For example, 120553124.23 represents GI number 120553124, it belongs to cluster number 23, which is a phylum Proteobacteria specific cluster and bootstrap value for the branch which encompasses this cluster is 99.

A dendrogram depicting protein kinases specific to certain taxonomic levels are represented schematically in figure 1. The distance matrices representing extent of sequence dissimilarities at the catalytic kinase domains in every cluster are provided in File S4.

### Ia) Phylum specific subfamilies of prokaryotic Serine/Threonine protein kinases

In our analysis we find certain subfamilies which are phylum specific. There are four clusters (numbered as 6, 23, 38, 63), with members exclusively from phylum Proteobacteria. Bacteria belonging to this phylum are Gram-negative phototrophic and heterotrophic which are generally referred as “purple bacteria and their relatives” [48]. There are 7 other clusters (numbered as 3, 4, 25, 29, 44, 59 and 62) with members predominantly from the same phylum. Clusters 62 and 3 contain substantial representation of members from *Myxococcus xanthus*. *M. xanthus* whose developmental cycle and multicellular morphogenesis resemble those of eukaryotic slime molds such as *Dictyostelium discoideum* is reported to contain a large family of Ser/Thr kinases [49], [50]. Transmembrane protein kinase pkn6 from *Myxococcus Xanthus* shows close similarity with a predominantly proteobacterial cluster. This kinase is expressed constitutively and is responsible for growth and development of the bacterium and it also has been speculated to sense the external signals for developmental process [51].

We also report here Tyrosine-protein kinase masK which happens to be a member of cluster 62 which belongs predominantly to phylum Proteobacteria. Protein kinase masK interacts with GTPase MglA to control social gliding motility of bacterium. masK is also reported to be essential for growth of the bacterium [52].

Another category in phylum specific clusters belongs to phylum Cyanobacteria. This phylum comprises oxygenic photosynthetic prokaryotes [53]. We report here 6 clusters (numbered as 15, 19, 24, 37, 54 and 67) members exclusively from Cyanobacteria. In addition, another cluster (numbered as 60), contains members mainly from cyanobacterial species.

*Synechocystis sp*. protein Ser/Thr kinases, spkA belonging to cluster 60 and spkB from very specific cyanobacterial cluster 54 are required for the normal motility of this unicellular cyanobacterium [54], [55]. Another protein kinase spkD from same organism is apparently essential for survival and protein spkF from *Synechocystis sp*. which is member of cyanobacteria specific cluster 67 has also been reported as a functional eukaryotic-like protein kinase [56].

Filamentous cyanobacterium *Anabaena* has differentiated cells called heterocysts which have a specialized function of nitrogen fixation. Protein kinase pknA has been shown to be important for normal cellular growth as disrupted pknA leads to formation of light green and rough colonies when adequate amount of nitrogen is not supplied. Moreover, a mutant results in formation of lower number of heterocysts as compared to wild-type filaments [57].

There are three clusters (numbered as 10, 14 and 26) with members predominantly from phylum Actinobacteria. This phylum comprises of Gram-positive bacteria with generally high G+C content in their DNA [58]. The phylum includes pathogens (*Mycobacterium spp*., *Nocardia spp*., *Corynebacterium spp*., and *Propionibacterium spp*), nitrogen fixing symbionts (*Frankia*).

Cluster number 32 has members predominantly from phylum Euryarchaeota of archaea, which comprises of methane producing methanogens and their phenotypically diverse relatives [59]. However, this subfamily members share close similarity with some of the kinases from eubacteria and hence this subfamily has members both from eubacteria and archaea.

### Ib) Order specific subfamilies of prokaryotic Serine/Threonine protein kinases

There are six clusters which are Order specific. Cluster 49 is Chlamydiales specific, member organisms of which are exclusively obligate intracellular parasite. For example, *Chlamydia trachomatis* belonging to same order causes trachoma which leads to blindness and sexually transmitted disease in human beings [60], [61], [62], [63]. Other members include *Chlamydia pneumoniae* which causes pneumonia and bronchitis in human beings [64].

Serine/threonine-protein kinase pknD from bacterium *Chlamydia trachomatis* (Chlamydiales specific cluster 49) is a functional kinase and is expressed at early mid-phase of developmental cycle. This protein also has been predicted to have transmembrane domain and might serve as a receptor to sense environmental stimuli to regulate cellular functions. Protein kinase, pknD has been shown to interact with another protein kinase pkn1. All these factors may help the pathogen to exploit the host signaling pathways and supporting its own growth [65].

Other 5 clusters are exclusively Actinomycetales specific. Members of cluster number 22 predominantly contain homologues from order Actinomycetales. One of the members of this order is *Arthrobacter aurescens* which is a soil dwelling aerobe capable of surviving in extreme conditions like starvation, temperature changes, ionizing radiation, oxygen radicals, and toxic chemicals etc and also has the ability to degrade pollutants [66]. Other members from genus *Corynebactrium* belonging to same order are *Corynebacterium diphtheriae* which produces diphtheria toxin and causes the symptoms of diphtheria [67], *Corynebacterium urealyticum* which causes urinary tract infection [68], multiresistant nosocomial pathogen *Corynebacterium jeikeium* [69], *Mycobacterium abscessus* which causes skin, soft tissue and pulmonary infections [70], *Mycobacterium tuberculosis* causing tuberculosis [71] and an unculturable obligate pathogen *Mycobacterium leprae* which is responsible for causing leprosy [72]. Soil dwelling bacteria corresponding to genus *Streptomyces* also belongs to Order Actinomycetales which produces over two-thirds of naturally derived antibiotics [73]. Ser/thr protein kinase afsK (member of Actinomycetales specific cluster) is reported to phosphorylate AfsR, a transcription factor which is involved in regulation of production of secondary metabolites such as actinorhodin and undecylprodigiosin *in Streptomyces coelicolor* [16]. It also has been shown to regulate aerial mycelium formation and spore formation, thus morphological differentiation in *S. griseus* [74].

Protein kinase, pknG from organism *Mycobacterium tuberculosis* (Actinomycetales specific cluster 46) has been shown to regulate glutamate/glutamine level in the cell as pknG deletion results in accumulation of these amino acids and is also important for the growth of bacterium [75]. Another study suggests that Mycobacterial pknG is secreted in macrophage phagosome and inhibits phagosome-lysosome fusion and thus enable the pathogen to survive in the host cell [76].

### Ic) Sub-order specific subfamilies of prokaryotic Serine/Threonine protein kinases

There are two clusters under this category (numbered as 31, 72). Based on the analysis of our main dataset of kinases these two clusters are comprised of members only from *Myxococcus xanthus.* But when kinase sequences from this subfamily were searched in a large sequence database Uniref90 (see Materials and methods section) close homologues, which can be considered to be members of this subfamily, were identified from prokaryotes *Angiococcus disciformis, Myxococcus xanthus and Stigmatella aurantiaca*. Interestingly all of these organisms belong to sub-order Cystobacterineae of order Myxococcales under phylum Proteobacteria. *Angiococcus disciformis* and *Stigmatella aurantiaca* produce antibiotics angiolam A and stigmatellin, respectively [77], [78]. Importance of *Myxococcus* is described in phylum specific cluster and organism diverse cluster section.

### Id) Class specific subfamily of prokaryotic Serine/Threonine protein kinases

We report here a class specific cluster. Cluster number 5 is Gammaproteobacteria specific. Members are included from genus *Shewanella* (bacterium belonging to this genus are exclusively marine) such as metal reducing *Shewanella amazonensis* [79], denitrifying *Shewanella denitrificans* [80] and respiratory luminous bacterium *Shewanella woodyi* [81]. Other members come from genus *Marinobacter*, *Pseudoalteromonas* and *Alteromonas*.

### Ie) Family specific subfamily of prokaryotic Serine/Threonine protein kinases

This subset corresponds to family Thermoproteaceae under archaea (Cluster number: 28). This family is characterized by hyperthermophilic archaeans. This includes members from genus *Pyrobaculum* such as *Pyrobaculum aerophilum, Pyrobaculum arsenaticum, Pyrobaculum calidifontis, Pyrobaculum islandicum* and also from *Thermoproteus tenax*. Protein kinases falling into this category are all single domain proteins.

### If) Genus specific subfamilies of prokaryotic Serine/Threonine protein kinases

There are five clusters (numbered as 7, 45, 48, 52 and 56) under this category. Cluster 7 is specific to genus *Sulfolobus* from archaea. Genus *Sulfolobus* represents sulfur-oxidizing microorganisms living at low pH and high temperature. Different thermoacidophilic species belonging to this genus are *S. acidocaldarius* [82], *S. solfataricus* [83] and *S. Tokodaii*. Protein phosphorylation studies have been carried out and Ser/Thr protein kinase has been reported from this archaeon [23], [84]. Protein kinase domains appearing in this cluster are not tethered with any other domain.

Clusters 52 and 56 are specific to genus Metallosphaera and Sulfolobus from family sulfolobaceae under archaea. Genus Metallosphaera comprises aerobic, metal-mobilizing, thermoacidophilic microorganisms [85].

One of the clusters (No. 45) is specific to genus *Bacillus* and *Geobacillus* of family bacillaceae which comprises of all Gram-positive bacteria. Interaction of phosphatase, BA-Stp1 with Ser/Thr protein kinase BA-Stk1 from *Bacillus anthracis*, has been shown to be responsible for the virulence of the bacterium [86].

Above mentioned clusters in this section have members which correspond to eukaryotic like protein kinase (epk) mentioned by Kannan et al [6].

Another cluster (numbered 48) is specific to genus *Chlamydophila* and *Chlamydia* from family chlamydiaceae. Genus Chlamydia comprises bacteria which are intracellular obligate parasites and causes diseases such as sexually transmitted diseases, blindness, pneumonia and bronchitis as mentioned above. A member of this cluster is present in pknb (bacterial specific) subfamily according to Kannan et al [6].

### II) Organism diverse subfamilies of prokaryotic Serine/Threonine protein kinases

There are 34 clusters falling under this category. Members of these clusters show close similarity to members from diverse organisms. Search, in Uniref90, for closely related kinases of one of the clusters (number 68) resulted in recognition of serine/threonine-protein kinase pkn1 of Gram-negative soil bacterium *Myxococcus xanthus*. This protein kinase is required for normal development of this bacterium and deletion of pkn1 gene results in premature differentiation and poor spore formation [87].

Cluster number 71 has pknD, a protein kinase member from *Mycobacterium tuberculosis.* It has extracellular highly symmetric six-bladed β-propeller structure which could bind a multivalent ligand and can act as a sensor domain [88]. PknD has been reported to phosphorylate MmpL7 which is associated with the formation of cell wall of bacterium and serves as virulence factor [89]. Studies also suggests role of this class of kinase in regulation of transcription of numerous genes in bacterium [90]. Same cluster has pknF, a protein kinase from *M. tuberculosis* which interacts with ABC transporter containing a Forkhead-associated domain to play role in virulence and cell growth of bacterium [91]. PknF involvement has been suggested in glucose uptake, cellular growth and septum formation also [36]. PknE, a transmembrane protein kinase, which phosphorylates multiple FHA domain is also a member of organism diverse cluster 61 [92].

PknH from *Mycobacterium tuberculosis,* is a member of a subfamily (cluster number: 71) and it has been shown to phosphorylate EmbR, which is mediated by FHA (forkhead-associated) domain [93]. Protein EmbR is associated with regulation of activity of enzyme arabinosyltransferase involved in arabinan biosynthesis of arabinogalactan which is an important molecule of the *Mycobacterial* cell wall. PknH deletion also leads to survival and higher bacillary loads in BALB/c mice, suggesting a role of the protein in regulating the growth profile of the bacterium [94].

A member of cluster number 17 protein kinase pknB from *Mycobacterium tuberculosis* phosphorylates PBPA, a penicillin binding protein and regulates the growth and cell division of the bacterium [95].

Serine/threonine-protein kinase prkC from Gram-positive bacterium *Bacillus subtilis* is a member of organism diverse cluster (cluster 33). PrkC participates in developmental processes like spore formation and biofilm formation as mutant of this gene show decreased efficiency of both the processes [32].

Members of cluster number 35 show high similarity to StoPK-1, serine/threonine protein kinase from *Streptomyces toyocaensis.* Disruption of StoPK-1 leads to unusual mycelium morphology. StoPK-1 is also associated with signal transduction pathways which are sensitive to oxidative stress as inactivation of same gene results in increased sensitivity towards oxygen radical-generating compound [31]

Clusters 11 and 55 have protein kinases pknA and pknB respectively from soil-borne, non-pathogenic Gram-positive bacterium *Corynebacterium glutamicum,* which is used for production of L-lysine and L-glutamic acid on commercial scale. These kinases are absolutely essential for *Corynebacterium* growth. Partial depletion of both kinases results in defect in cell division and formation of elongated cell [96].

Cluster 13 has transmembrane protein Ser/Thr kinase pkn2 from *Myxococcus xanthus* which has been shown to phosphorylate beta-lactamase and restrict its secretion across the membrane in *E.coli*. Enzyme beta lactamase are produced by certain bacteria and are responsible for bacterial resistance against beta-lactam antibiotics such as penicillins. Thus pkn2 is speculated to regulate the activity of penicillin binding proteins. Disruption of pkn2 also results in low yield of myxospores [97].

There are two organism diverse subfamilies (numbered 30 and 9) with members from both archaea and eubacteria. However most of the members in these two clusters are from eubacteria with only a minor representation from archaeal organisms (∼1% and ∼2% in subfamilies 30 and 9 respectively).

### Domains associated with prokaryotic Ser/Thr protein kinases

It is well known that many of the prokaryotic protein kinases are not multi-modular in nature. However, there are few prokaryotic Ser/Thr kinases identified which have other domains tethered to the protein kinase domain which adds complexity to the type of function they are performing. Domain architectures of all the prokaryotic kinases in each of the 72 clusters are provided in File S5.

The most commonly tethered domains to the protein kinase domain are Tetratricopeptide (TPR) repeats, PASTA, WD40 repeats, GAF, PD40 repeats and APH domains. TPR repeats are involved in variety of functions such as extensive protein-protein interaction in the assembly of multiprotein complexes [98]. WD40 repeats containing proteins are involved in wide range of functions like signal transduction, RNA processing, gene regulation and regulation of cell cycle [99]. WD40 repeats help in coordinating multi-protein complex assemblies, where the repeating units of WD40 serve as a rigid scaffold for protein-protein interactions [100]. PASTA domain is found in both archaea and bacteria, occurs at C-terminus of several penicillin-binding proteins and bacterial serine/threonine kinases [101]. While the GAF domain is known to participate in photo transduction in plants and vertebrates [102], it has been reported to have role in change of pigment-protein composition according to light color changes in cyanobacteria [103]. This domain has also been speculated to participate in regulation of various signalling events in non-photosynthetic bacteria. PD40 protein domain family is related to WD40 domain family and is a cell surface protein [104]. Another most commonly tethered domain is APH (aminoglycoside phosphotransferase) domain. Aminoglycoside phosphotransferases are proteins which inactivate aminoglycoside antibiotic substrate by phosphorylating the same in prokaryotes [105].

Domains which are generally tethered to Ser/Thr kinases specific to Proteobacteria are SAF domain which is found in antifreeze proteins, flagellar proteins and pilus proteins [106], Universal stress protein (Usp) family which is expressed in response to stress agents in bacteria [107] and APH domain which is reported to inactivate the antibiotics in prokaryotes.

Domains generally tethered to Cyanobacteria specific Ser/Thr kinase are Pentapeptide repeats which are most commonly found in Cyanobacteria and speculated to be involved in Cyanobacteria-specific metabolism [108], WD40 repeats, APH domain, TPR repeats, CHASE2, which is an extracellular sensory domain present in various classes of transmembrane receptors that takes part in signal transduction pathways in bacteria and archaea [109], SH3_3 (src Homology-3 ) domain which is involved in signal transduction and cytoskeletal organization [110] and FHA (forkhead-associated) domain which is a phosphopeptide binding motif [111].

In addition to supporting information files with this paper details of prokaryotic Ser/Thr kinases identified in this study can be found at KinG database (http://hodgkin.mbu.iisc.ernet.in/~king/) in the link “A Framework for Classification of Prokaryotic Protein Kinases”.

## Materials and Methods

From 303 prokaryotic genomes, 993 non redundant eubacterial and archaeal Ser/Thr protein kinases have been retrieved from KinG (database version 1.5) [112]. Briefly, protein kinases are identified using a combination of profile-based search methods such as PSI-BLAST [113] and RPS-BLAST [114] using multiple profiles (MulPSSM) [115], [116] and HMMER search [117], which have been previously benchmarked and has been used in our earlier kinome analysis for several other genomes [118], [119], [120], [121].

Multiple Position Specific Scoring Matrices (MulPSSM) from 2810 sequence profiles have been generated from different groups of kinases as mentioned in www.kinase.com. In case of single profile approach a reference sequence is chosen arbitrarily for building a PSSM and the query sequence is searched in database of PSSMs of various protein families. But in the case of multiple profile approach every sequence from a given multiple sequence alignment of a protein domain family is used for building PSSMs which increases the search space as well as removes bias toward the reference sequence. Protein sequences form prokaryotic genomes have been searched in database of Multiple PSSMs using RPS-BLAST. Conditions for hit in RPS-BLAST searches include an e-value cut-off of 10^−4^ and more than 70% of profile should be covered by the query in the alignment. Ser/Thr kinases have also been identified using HMMER against Pfam [122] (release 23) protein kinase (Pfam code: PF00069) profiles. E-value cut off used in HMM search is 0.01. Amino acid sequences from prokaryotes have been searched, using PSI_BLAST, in a database of kinases procured from Pfam. E-value cut-off used in this method is 0.0001. Query should cover greater than or equal to 70% length of the sequence in the database in the alignment for considering the database entry as a hit.

CD-HIT [123], a program for clustering large protein database at specific sequence identity threshold has been used to make the prokaryotic protein kinase domain dataset non-redundant at the sequence identity cut off 40%. Hence no two sequences have more than 40% sequence identity to each other across any two clusters. The number of clusters generated by the CD-HIT program is 270. There are 126 clusters with only one member in each suggesting their high sequence divergence. There are 72 clusters which have four or more members in each cluster. These 72 clusters are considered as prominent subfamilies of prokaryotic Ser/Thr kinases.

A randomly chosen member from each of these 72 clusters was searched, using PSI_BLAST, in Uniref90 (www.ebi.ac.uk/uniref/) dataset which is a comprehensive collection of amino acid sequences of non-redundant proteins. Uniref90 represents the best current knowledge on amino acid sequences from diverse organisms. Our kinase dataset is derived from KinG (version 1.5) and has information for only for 303 prokaryotic genomes. However to classify any cluster as, for example, phylum Cyanobacteria specific, we have not only considered phylum of member organisms from that clusters, we also ensured by searching into comprehensive database Uniref90 that member of this cluster picks up homologues from Cyanobacteria phylum only. If proteins from other than Cyanobacteria are picked-up as close homologues of the query then the cluster concerned is not considered as Cyanobaceria specific. Close homologues have been identified with sequence identity of 40% or greater and greater than or equal to 70% query coverage. These conditions should be satisfied over and above the E-value cut-off of 0.00001.

Multiple sequence alignment program, CLUSTALW has been used to align kinase domain sequences from each of the 72 clusters [124]. The tree was generated using neighbor-joining (NJ) method [125]. NJ method provides topology as well as branch length of final tree. This method is based on principle of finding pairs of operational taxonomic units (OTUs), “neighbors” that minimizes the sum of branch lengths at each stage of clustering of OTUs. Tree is annotated with the bootstrap values (1000 iterations).

MEGA program (version 4.0) has been used to draw the tree [126]. Many of the prokaryotic protein kinases are not multi-modular in nature but some of them have domains tethered to the protein kinase domains. The domain architectures of these prokaryotic Ser/Thr kinases have been identified on the basis of searches using HMMER [117] against the Pfam (release 23) profiles [122] containing 10340 families. E-value cut -off used in this search is 0.01.

### Conclusions

The present study involving identification and analysis of Ser/Thr kinases in prokaryotic genomes has provided insights into signal transduction and metabolic processes in prokaryotes. The extensive dataset of prokaryotic kinases obtained from KinG has given us the opportunity to classify these kinases into different categories based upon their occurrence in particular taxonomic group.

Specificity of Protein Ser/Thr kinases at particular taxonomic level suggest requirement of these Ser/Thr protein kinases for certain specific function which is lineage specific and not needed for all the prokaryotes. It is interesting to note that occurrence of several taxonomic specific subfamilies of prokaryotic kinases contrasts with classification of eukaryotic protein kinases in which most of the popular subfamilies of eukaryotic protein kinases occur diversely in several eukaryotes. Clusters representing prokaryotic protein kinase subfamilies which are taxonomic level specific suggest role of these Ser/Thr protein kinases in some specific function being carried out by limited sets of prokaryotes. Finally, organism diverse subfamilies of prokaryotes suggests wide spread occurrence of such Ser/Thr kinases. Almost 50% of the clusters obtained in this analysis have only one member suggesting their sequence and, probably, functional divergence. Genomic data of many more prokaryotes is not yet available. With the completion of genome sequencing of many more prokaryotes, some of these clusters may have additional members. Ongoing efforts are directed towards development of profiles of clusters in the present classification scheme for prokaryotic Ser/Thr protein kinases. This should allow convenient classification of prokaryotic Ser/Thr kinases in the future.

## Supporting Information

File S1 Amino acid sequences of prokaryotic protein kinases (catalytic domain).
(0.29 MB TXT)

File S2 Taxonomic level specificity details of clusters.
(0.08 MB XLS)

File S3 Multiple sequence alignments of clusters.
(0.35 MB PDF)

File S4 Distance matrices of clusters.
(0.11 MB TXT)

File S5 Domain organization of kinases in various clusters.
(0.10 MB XLS)
