# Supplementary material for: A Framework for Classification of Prokaryotic Protein Kinases
Source: PLoS One. 2010 May 26;5(5):e10608. doi: 10.1371/journal.pone.0010608 (PMC2877116; doi:10.1371/journal.pone.0010608)
Supplement: File S3 — Multiple sequence alignments of clusters. (0.35 MB PDF) [file pone.0010608.s003.pdf]

# Multiple sequence alignments of clusters

This file contains multiple sequence alignments of clusters (generated by Clustalw2) which have 4 or more members (only catalytic kinase domain). First column in multiple sequence alignment of any cluster represents GI number of protein.

## Cluster No. 1 multiple sequence alignment

```
CLUSTAL 2.0.10 multiple sequence alignment


113476443       YKLTKQIYESPNSLVFQGLLKPN--NQPIILKFLKENYPTPSELTRYKQEYEITRSLNKR
113477314       YKLTKQIYESPNSLVFQGLLKPN--NQPIILKFLKENYPTPSELTRYKQEYEITRSLNKR
17130246        YQLIEQLYHGSKTLVYRGIRRTDSAAQPVVIKLLQREYPSFSELLQFRNQYTIAKNLNIP
83312035        YRLTEEVYSAGNTRVCRAVSLAD--GKPVILKILSGEQLSPDAFARYQREYEITSSLAEV
                *:* :::* . :: * :.:  .:   :*:::*:*. :  : . : :::.:* *: .*

113476443       EQIIIVYDLHRYNNSLVILLEDFGGKSLKLLLPQTQFTLEEFLTIAIQITKGLAVIHTNN
113477314       EQIIIVYDLHRYNNSLVILLEDFGGKSLKLLLPQTQFTLEEFLTIAIQITKGLAVIHTNN
17130246        G-IVHPTSLEPYGNSYALVMEDFGGVSLGSYSQTHPLSLGDVLAIAIQLANILHDLYQNR
83312035        EGVTTVLAMENVQDSVMIVQEDVGGTSLARILEAGRLDLEEALALAVRITQILGRIHRHR
                  :     :.   :*  :: **.** **        : * : *::*::::: *  :: :.

113476443       IIHKDINPSNIIYNPQTGQLKIIDFGISTRLSQEFIKVFPPHQLEGTLAYIAPEQTGRMN
113477314       IIHKDINPSNIIYNPQTGQLKIIDFGISTRLSQEFIKVFPPHQLEGTLAYIAPEQTGRMN
17130246        VIHKDIKPANLLIHPETKEIKLIDFSIASLLPKETEAVKHPKVLEGTLAYLAPEQTGRMN
83312035        VIHKDFNPTNIIWNRQTGTVRVIDFGISSQLSQERAEFQSVKQLEANLAYASPEQTGRVN
                :****::*:*:: : :*  :::***.*:: *.:*   .   : **..*** :******:*

113476443       RGIDYRSDFYALGVTFYELLTNQLPFETNDSIELVHCHIAQQPLPPHKLNPNIPNSLSNI
113477314       RGIDYRSDFYALGVTFYELLTNQLPFETNDSIELVHCHIAQQPLPPHKLNPNIPNSLSNI
17130246        RGIDYRSDFYAFGVTLFELLTGKLPFISDDPLELVHCHIAKPAPSVCALRPEIPAVIGEI
83312035        RALDSRSDLYSLGVTLYQMLTGTLPFLSRGGIELVHAHIALMPPPPHEVSHGIPPILSQI
                *.:* ***:*::***::::**. *** : . :****.***  . .   :   **  :.:*

113476443       IIKLLAKSPEERYQNALGIEADLKKCLSQL
113477314       IIKLLAKSPEERYQNALGIEADLKKCLSQL
17130246        IGKLMAKNAEDRYQSALGLQYD--------
83312035        VMRLMAKMADDRYQSARGLEHDLQ------
                : :*:** .::***.* *:: *
```

Cluster No. 2 multiple sequence alignment

```
CLUSTAL 2.0.10 multiple sequence alignment


17130231        YQNLTQIYAGTRTLVYRGIREEDQKPIVIKMLRYEYPSFSELVQFRNQYTITKNLHISGI
17133824        YRITEQLYFGSKTIVYRGLRKQDQKPVVIKLMRNEYPTFQEIAQFRNQYTITKDLDIPGI
17130054        YNINEELYHGSRTLVYRGYREVDQQAVVVKLLKNPYPSFNELLLFRNQYTIAKNLNSPLI
17130055        YSISEELYNGSRTQVYGGYREVDQQPVVIKLLKNPYPSFNELLLFRNQYTIAKNLNSPLI
17129699        YAISEELYNGSRTLVYRAVREVDCLPVVIKLLKNHYPSFSELVQFRNQYTIAKNLDYPGI
17132690        YHISSQLYAGSKTRVYRAIREQDQRPVVIKLLASDYPNFHELLQFRNQYTISKNLNVTGI
17135445        YQISSQIYAGSKSRVYRATREQDTLTVIIKVLTSEYPSFNELLQFRNQYTISKNLNVTGI
113475755       YQITQEIYTGTKSKVYRGINTITQQPVVIKVLKKEYPTFSELLQFRHQYTITKNLNLAGV
17131374        YQIVEKIYDSANSEVYRSIRQEDNQSVIIKVLKQEYPTPIELTRYKQEYEITRNLDFDGV
17131349        YQIIELIGEGVKTNVYHAISIPDSKLVVIKILKTEYPELKDIAALKHEYELIKNLDIPGV
24195011        YEIKDRMNTDSSTEVYKAVRSKDKTEVVVKYIPILDELHPAVVNLRNEYEILSYLSSEKM
                *     :  .  : ** .        :::* :         :   :::* :   *    :

17130231        IKTYSLENYENSYALVMEDFGGVSLKDWSIQNSE-----ISLDEFFHIAIQIVTILDGLY
17133824        VKPLSLETYRNSYALVMEDFGGLSLKDWGQISKDGNEYGTVLKRFFHIAIAIASTLESLH
17130054        VQTYSLEPCKNGYALVMEDFGGISLKEWGTRETQ-----QSLEEFLMIAIALCNILDLLY
17130055        VQNYSLEPYQNGYALVMEDFGGISLKKWGTRQTQ-----QSLEEFLVIAIALCDIFNLLY
17129699        IKTYSLEPLQNGYQLVMEDFGGISVKDYFANNNV-----ASLEKFLQIAIALCDILDILY
17132690        IRPLSLETYGNGYILVMEDTGGIALREY-IKTTP-----FPLVEFLAIAIQITNILQELH
17135445        THPLSLDVYGNSYILVMKDTGGISLRQY-TETAK-----LTLIEFLAIAIQLTNILHGLH
113475755       AKPLSLEKYGNGYALIMEDIGAVSLNTYLSNQKL------NLQKFLELAISMVEILEGLY
17131374        IKAYSLEPYYRTLVIILEDFGGTDLRDFHNINSI--------EKFLAIAIKITDTLAQIH
17131349        VKAHGFEKYNNCFALVLEEFDGTSLHKVIQEKKIG------LLDFCKIGIQITQALGELH
24195011        IRAFGMEKIPEGFILILEFVPGQSLKHFSQKRPVN------LKDFFKIAIDLAEKLGEIH
                 :  .::   .   ::::   .  :.                  *  :.* :   :  ::

17130231        RSRVIHKDIKPSNILINPITKEIRLIDFGIASLLPRETQTLTSPNVLEGTLAYLSPEQTG
17133824        RSRIIHKDIKPANILINPTTLEIRVTDFSIATLLPREVQVLTNHNVLEGTLAYLSPEQTG
17130054        HQRIIHKDIKPSNILINPQTKQVKLIDFSIASLLPRETQTLVNPNVLEGTLGYISPEQTG
17130055        HERIIHKDIKPSNILINPETQQVKLIDFSIASLLPRETQTLVNPNVLEGTLAYISPEQTG
17129699        RERIIHKDIKPANILINPKTTQVKLIDFSIASLLPRETQTLINPNVLEGTLGYISPEQTG
17132690        LNRVIHKDIKPANILIHPQTKQVQLIDFSIASLLPKETQEIRSPNVLEGTLAYISPEQTG
17135445        QNRVIHKDIKPANILINPHTKQVELIDFSIASLLPKETQEIKSPNILEGTLVYISPEQTG
113475755       HHQIIHKDIKPSNILINPDTGDVKLIDFGISTLLTRETQEVINPNILEGSLTYISPEQTG
17131374        AANVIHKDINPSNIVFNPKTGEVKIIDFGIATVFTRENTTLKHPNVLEGTLAYISPEQTG
17131349        RKYIIHKDIKPQNIIVNLETHQVKIIDFSISSLLFQEKAKLNNPNLLEGTIAYMSPEQTG
24195011        NKKVIHKDLKPDNIIFNPDENILRIVDFGISTRLSKEESSWSNPNRLEGSIHYVSPEQTG
                   :****::* **:.:     :.: **.*:: : :*       * ***:: *:******

17130231        RMNRGIDYRSDFYSLGVTFFELLTGQLPFYCDDPMKLLHCHIAKRPPQVHDINSTIPLAV
17133824        RMNRGIDYRSDFYSLGVTFFELLTGQLPFMTIEPMELVYCHIAKQPPKASDINPKIPTIL
17130054        RMNRGIDYRTDFYSLGVTFYELLTGGLPFASNDPIELVHSHIAKIAPLAHEINSEIPSVL
17130055        RMNRVVDYRTDFYSLGITFYELLTGELPFASNDAMELVHSHIAKTVPLAHEINLDIPSVI
17129699        RMNRGIDYRTDFYSLGATFYELLTGTLPFPSEDAMELVHCHLAKAATLVHETNTTIPSVL
17132690        RMNRGIDYRSDFYSLGVTLYELLMGELPFSSDDPMELVHCHIAK-TPIALGHQQHIPLVL
17135445        RMNRGVDYRSDFYSLGVTFYELLTGELPFNSNDPMELVHSHIAK-QPNKF-NPESIPQVI
113475755       RMNRGIDYRSDFYSLGITFYEMLTKQLPFVSSDPIELVYFHIAKEPTPPANLNKEIPLMV
17131374        RMNRALDYRTDFYSLGVTFYELLTKQLPFDTTDSLELVYCHLAKQPIPPHEIHSNIPKTL
17131349        RMNRSIDYRTDFYSLGVTFYEMLTGQLPFIVTDPMELVHCHIAKQPISINQLIPEIPEVV
24195011        RMNRSVDYRSDFYSLGITFYELLTGKLPFESEDLLELVHFHLAKSPVDPRKIRSEIPEAL
                **** :***:****** *::*:*   ***   : ::*:: *:**           **  :

17130231        SNIAFKLMAKNAEDRYQSALGLKHDLE-------
17133824        SDLISKLMAKNAEDRYQSAYGLAYDLE-------
17130054        SKIVSKLMEKNAEDRYQSALGLKFD---------
```

```
17130055        SEIIRKLVAKNAEDRYQSALGLKFD---------
17129699        SDIVNKLMAKNAEDRYQSALGLKYD---------
17132690        SDIIMKLMAKNAEDRYQSALGLKHDLE-------
17135445        SDIVMKLMAKNAEDRYQSALGLKHDLE-------
113475755       NNIILKMIAKNAEERYQNAIGLKHDLQ-------
17131374        SDIVMKLMAKTAEERYQSALGIKADL--------
17131349        SEIIMKLLSKTAEERYQSALGIKADLEKCLNQL-
24195011        SHVILKLLSKTAEERYQTSEGLKNDLEIIRDKWL
                ..:  *:: *.**:***.: *:  *
```

Cluster No. 3 multiple sequence alignment

```
CLUSTAL 2.0.10 multiple sequence alignment


108757395       FIPLKLLGQGGMGAVFAAYDPDLDRKVALKLLSVE-ARSADEEGGRARLLREAQAMARVS
108761044       YFLLKRLGQGGMGVVYAAYDPDLDRKVALKLLHAD-SRTDSEE-ARARLLREAQAMARVS
108761750       YHILDTLGAGGMGAVYSAYDPELHRRVAIKLLHPD-ANPAARSDGSSRLLREAQAMARLS
108757860       YVVQQRIGRGAMGEVYAAHDPELDRRVALKLLRPE-GRH--LEELRLRLLREAQALARLA
108763079       YVVRECLGAGAMGIVYAADDPELGRRVALKVLRPK-GSQ--REELQQRLLREAQALARLS
108763872       YVVLGLLGEGGMGRVHVAYDPELDRKVALKLLKPERFHEDSLALARQRLEREARTMAKLS
108764042       YVVLGLLGEGGMGRVHAAYDPELDRKVALKLLNLARLGEDSLAQARQRLEREARTMARLS
                :     :* *.** *. * **:* *:**:*:*               ** ***:::*:::

108757395       HPNVIPIYEVGTWDTQVFFTMEWVAGGTLADWRREKLRSWREVLEKYLQAGRGLEAAHAA
108761044       HPNVIPIFDVDVWGDRVFLAMELVDGGTLASWVKEGQRSWREILESFLAAGRGLQAAHEA
108761750       HPNVVSVYDAGTFAGRVFIAMERVDGLSLRHWLRAEHRTWREVLDIFRQAGRGLAAAHAA
108757860       HPHVVTVYDVGVCEDCVFLALELVEGASLAEWLEAP-RPWQDVVRVFVDAGRGLAAAHAA
108763079       HPNVVTLYDVGAYGDGVFLTMELVEGTTLAEWMKER-RPWKEVLRVFLEAGKGLAAAHAA
108763872       HPHIASLHDVGEYQGQLFLVMEFLEGGTLRRWLTEQPRPLREVLERFRQAADGLAASHAL
108764042       HPHVAQLHDVGEFQGQLFLVMELVEGGTLRRWLAEKPRTRREILARFTQAADGLAATHAL
                **::  :.:..     :*:.:* : * :*  *     *. ::::  :  *. ** *:*

108757395       GLVHRDFKPANVLVGRDGRVYVTDFGLARPVDNLPLDEQ---PTLARVRDASETSRTLHD
108761044       GLVHRDFKPANVLVNKAGRVFVTDFGLARPVGTLPKEEP---LSEDAEALIPSERRMLDT
108761750       GLVHRDFKPANVLVSKDGRAQVTDFGLARTTADLDAAEAGRGTTPALPRLAPED--LLQT
108757860       GLVHRDFKPGNVLVGKDGRVRVTDFGLAR----PSHRGVSGHAAPASLDTVTAPASLVDS
108763079       GLVHRDFKPANALIGKDGRVFVTDFGIAR----LLQQ----EEGASHQEHIEAPVTPTGR
108763872       GIVHRDFKPDNVLLTKGGLVRITDFGLAN------------ATL---VPGTAAPGTVSPA
108764042       GIIHRDFKPDNVLLTRDGQVRITDFGLAN------------AAVGMGPPREDAAIDSCEA
                *::****** *.*: : * . :****:*.

108757395       PLTETGVVLGTPPFMSPEQFRGESLDPRSDQFSFCAALYRALYNQRPFDPDELSRAAKAL
108761044       PLTEAGLIIGTPSYMSPEQFRGDDLDPRSDQFSFCVALYWALYRQRPFEPAKMEAYASSR
108761750       ALTEAGLVMGTPAYMPPEQHEDGRIDARGDQFSFCASLYEALYGQLPFNGKRPEDYLEEA
108757860       PLTHSGALLGTPAYMAPEQLQGHGVDARSDQFSFCVALYEALHGVRPFEGRTLEALGQAA
108763079       -LTRTGQLLGTPAYIAPELVRGQRADARSDEFSFCVALHEALFGARPFQGETLQEVVLAA
108763872       SLTVTGTLLGTLAYGAPEQLRGEHGDARSDQFSFCVALYEALNGQRPFEGKTREALLEQV
108764042       PLTVTGAFLGTPAYGAPEQLRGERGDARSDQFAFCVALYEALNGQRPFAGATREELLASM
                 ** :* .:** .: .**  ..   *.*.*:*:**.:*: **    **     .

108757395       R---------SAPAKVTAPLERAGREPSP----IHEPPSDARVPAWVRRAVMRGLSLEPE
108761044       KPQAQPVVEATEPLNVTAPLEREAPKPLPPPVLIQEPPRDVKVPAWVKQAMMRGLSLDPA
108761750       R---------------------AGR--------VRPPPRGSRVPTWVLRAVTRGLSPAPE
108757860       R---------------------EGR--------IRAPERESKIPARVRRAVLRGLRAQPE
108763079       Q---------------------QGR--------MSPPKREVKVPTRVRRAVFRGLSAKPE
108763872       A-----------------------R--------KAVRPERAGVPAWLRAVVRRGLSADPA
108764042       Q-----------------------R--------QEVRPEQRGVPSWLKAVVRRGLSVDPS
                                                          :*: :  .: ***   *

108757395       ARFASMGALLEAL-
108761044       ARFASMEALLGAL-
108761750       ARFPSMGVLLDAL-
108757860       ERFPSMEALLTELA
108763079       DRFPTMQSLLAAL-
108763872       ERFSSMEVLRARLS
108764042       RRFESMQVLRARLA
                 ** :*  *   *
```

Cluster No. 4 multiple sequence alignment

```
CLUSTAL 2.0.10 multiple sequence alignment


28855779        YRIERLLGAGGMGAVYRAKDLLSEQFGEPDPYVALKILSEEFAESPDASALLYSEFALTR
28872524        YRIERLLGAGGMGAVYRAKDLLSEQFGEPDPYVALKILSEEFAESPDASALLYSEFALTR
9947642         YRLERMLGAGGMGTVYRARDLLHEQFGDPAPLVALKLLNESVAESPDASALLYSEFALTR
104779802       YRLERLLGAGGMGRVYRARDLLQERFGDPQPLLALKLMSDQLATAPDANALLFNEYALTS
                **:**:******* ****:*** *:**:* * :***::.:..* :***.***:.*:***

28855779        RLRHNNVLRLHSFEVDTNCQRAFITMELMRGLTLDKLLCERPLGLPWRELRDIALSLLDA
28872524        RLRHNNVLRLHSFEVDTNCQRAFITMELMRGLTLDKLLCERPLGLPWRELRDIALSLLDA
9947642         RLRHPNVVRLFTFDVDTACQRAYIVMELMPGLPLDRLLCERPEGLPWSELSAIARPLLDV
104779802       RLRHPNLVRLQGFAVDPESDRGFITMELMRGTSLDRLLCDQPMGLAWAQVREIAVPLLDV
                **** *::**  * **. .:*.:*.**** * .**:***::* **.* ::  ** .***.

28855779        LAYSHARGVLHGDMKPSNVMLSEEGVRLFDFGLGQAEEGVMPGLPHLSRERFNAWTPGYA
28872524        LAYSHARGVLHGDMKPSNVMLSEEGVRLFDFGLGQAEEGVMPGLPHLSRERFNAWTPGYA
9947642         LAYVHEQGVLHGDLKPSNVMLGEEGVRLFDFGLGQAQAGILDGLPQLSRGRIDAWTPGYA
104779802       LACVHAQGVVHGDLKPSNVMLTDDGLRLFDFGLGMALEGPLAGLPHLSHGRFEAWTPAYA
                **  * :**:***:******* ::*:******** *  * : ***:**: *::****.**

28855779        APELLEGQALSASADVYGVACVIFELAGGKHPFRRLPSTQARDERLERELKAPRNLPKHC
28872524        APELLEGQALSASADVYGVACVIFELAGGKHPFRRLPSTQARDERLERELKAPRNLPKHC
9947642         APELLEGAPLSPAADLYALACVLYELADGRHPFRRLPSNQAREQGLERQLRAPRHLPRRC
104779802       APELFEGGTPSTASDLYAVACVLYELVTGRHPFDRVLSLKAREQGLQHALRVPENMPAKA
                ****:** . *.::*:*.:***::**. *:*** *: * :**:: *:: *:.*.::* :.

28855779        WPALRSALRFDAQDRKITAKQLRDAF
28872524        WPALRSALRFDAQDRKITAKQLRDAF
9947642         WPALRTALSLDPERRCIGVRELQEAL
104779802       WTALRTALVFDPGQRSIGAAQLRDAF
                *.***:** :*.  * * . :*::*:
```

Cluster No. 5 multiple sequence alignment

```
CLUSTAL 2.0.10 multiple sequence alignment


113968923       YRDIEFIGKGAYGFVFAGINQADEAH-----VFKFSRINLPQHIQDRLEEEAFMLSLLKH
24372172        YREIEFIGKGAYGFVFAGINQADEAH-----VFKFSRINLPQHIQDRLEEEAFMLSLLKH
114048939       YRDIEFIGKGAYGFVFAGINQADEAH-----VFKFSRINLPQHIQDRLEEEAFMLSLLKH
126172788       YTDIELVGKGAYGFVFAGINQAGDAH-----VFKFSRINLPQHIQDRLEEEAFMLSLLKH
109898357       YQDIELIGKGAFGFAFAGKPTHDPSK---HYVFKFSRANLPQHVQDRLEEEAYMLGHVAH
71281961        FEDVALLGKGAYGFVFSGIKRKGSSADQLPVVFKFSRINLPQHVQDRLAEEAEIQGQLSH
                : :: ::****:**.*:*    . :      ****** *****:**** *** : . : *

113968923       PNIPGAIKFERVGKQGILVMERAKGEDLEQLCRRLGALPPAMVMSIARQLADILYYLRTG
24372172        PNIPGAIKFERVGKQGILVMERAKGEDLEQLCRRLGALPPAMVMSIARQLADILYYLRTG
114048939       PNIPGAIKFERVGKQGILVMERAKGEDLEQLCRRLGALPPAMVMSIARQLADILYYLRTG
126172788       PNVPGAIKFERVGKQGILVMERAKGEDLDQLCRRMGALPTAMVMSIARQLADILYYLRTG
109898357       AHVPSLVTFQRIKKQSILVMQRAQGKDLEKVSLEHGPLSPRLVVKIAVQLADILQTLRTF
71281961        PQIPEVIDYQKIKRQSILQMTRAPGIDLEQLSLQVGPLAPELVVSIALQLADILLYLRQA
                .::*  : :::: :*.** * ** * **:::. . *.*.. :*:.** ******  **

113968923       ------KPLVHGDIKPSNLVYDIESQHLSLIDWGSAVFAQRDEHNKAVEDNVMTLMSSDQ
24372172        ------KPLVHGDIKPSNLVYDIESQHLSLIDWGSAVFAQRNEHNKAVEDNVMTLMSSDQ
114048939       ------KPLVHGDIKPSNLVYDIESQHLSLIDWGSAVFAQRDEYNKAVEDNVMTLMSSDQ
126172788       ------KPLVHGDIKPSNLVYDIEQQHLSLIDWGSAVFAQRDEYNKPVEDNVMALMSSDQ
109898357       SQNGHPKPIVHGDIKPSNIVFDPTTEEVGLIDWGSSVFAQLDAQGNYLANNVMDLMSNDM
71281961        NNHRQLKPIVHGDIKPSNLVYDKSTGKVQLIDWGSSVSAQLDCDKQSTSTNVMDLMSSDL
                      **:*********:*:*    .: ******:* ** :   :    *** ***.*

113968923       QHTNARMGDVYFIGEEQLNGALSSPRFDEQGVAATLYALASGQASRFGAQVIPPTSIGLP
24372172        QHTNARMGDVYFIGEEQLNGALSSPRFDEQGVAATLYALASGQASRFGAQVIPPTSIGLP
114048939       QHTNARMGDVYFIGEEQLNGALSSPRFDEQGVAATLYALASGQASRFGAQVIPPTSIGLP
126172788       QHTNARMGDVYFIGEEQLNGALSSPRFDEQGVAATLYALASGQASRFGAQVISPTSIGLP
109898357       QQTNARLGDVYFIGPEQLSGNLSSPRFDEQGLASTLYALASGQSSRFGAQVIRPNALGLP
71281961        QHSNARLGDVYFIGPEQLNGGLSSPRFDEQGLAATLYALASGQSCRYGVKVIPPNALGLP
                *::***:******* ***.* **********:*:*********:.*:*.:** *.::***

113968923       MELARTLEGMLSSDAVQRNLAGDYFLKSM
24372172        MELARTLEGMLSSDAVQRNLAGDYFLKSM
114048939       MELARTLEGMLSSDAVQRNLAGDYFLKSM
126172788       MELARTLEGMLSTDPKQRNIAGDYFLKSM
109898357       KMLAEILTAMLSDDKQKRAQGGDYLLNNI
71281961        KLLAQVLTGMLSDDEKLRAQAGDYF----
                  **. * .*** *   *  .***:
```

Cluster No. 6 multiple sequence alignment

```
CLUSTAL 2.0.10 multiple sequence alignment


121592911       FVVHECIHAGGMAHIYRVGYANASRDPGFPMAMKIPRMTAGDGAENIVGFEVELTILPTL
121610423       FRVRECLHAGGMAHIYTVDYAQAGRSPGFAMAMKIPRMTAGDGAENIVSFEVELQILPVL
120612910       FVVHECLHAGGMAHIYRVSCADPAQDPGFPLVMKVPRMTAGDGAENIVGFEVELQILPAL
86160117        FRIGERFHAGGMGVIYRVT----GPDPGFPMIMKIPRLGPGEPASSVISFEVEQTVLAAL
115525927       FVIEDRIHQGGMATLLIVR----RPDQSMPMVMKLPKIGEGEDPAAIVSFEMEQMIMPRL
27353958        YTIGECVHAGGMATLWTVT----HPGIDVPLLMKIPRVSEGEDPAAIVSFEMEMMILPRL
124268216       FRLEEHLHQGGMANLWRVT----HPDHAQPMLMKLPRIKGGEDPATIVGFEVEQMILPRL
13475495        FELVEKLPSGGMASLWRAT----NPRYDFPLVLKIPFLDPGADVSVILGFEAEELILKRL
                : : : .  ***. :  .           .: :*:* :  *     ::.** *  ::  *

121592911       SGHHAPRFVAAGDLMRLPYLVMEYVQGDTLQHWLDDRPPGDTAARSAETIARIGGAMALA
121610423       TGPHVPRFVAAGDLLRLPYLVMEYIPGQTLQHWIDAPERSDSS-----TIARLGTAVAHA
120612910       QGPHAPRFVAAGDLARLPYLAMEYVEGDTLQHRLDAGHRPGAD-----DIARLGAAMALA
86160117        KGPHVPRFVAAGDLERQPYLVMEEVRGRSLKEWVGDEPVPLDE------VVRIGIALATA
115525927       SGPHVPAYVAAGDFSAQPYIVMERIAGKALLSRLPELPLPYAD------AVDIAARIAAA
27353958        AGPHVPSCFGTGDFAHQAYVVIERIPGTTLYKRLADLPLPYEE------ARLLVAKIATA
124268216       QGPHVPRYVAQGDFTAQPYLVMEQLPGESLRPRLDRAPLPIDE------VVTIGAKVATA
13475495        SGPHVPRFAASGSLAQVPYIAMEFVVGKGLADIVGNAPLPMDE------VARIGIEIATA
                 * *.*   . *.:   .*:.:* : *  *   :                  :   :* *

121592911       AHSIHQQNVCHLDLKPANVLLRPDG-----SVVLLDFGLSCHAHYPDLLAEEMRQAVGSP
121610423       AHSLHQQNVCHLDLKPANVLIRDDG-----SAVLLDFGLSCHAHYPDLLAEEMRQAVGSP
120612910       AHSLHQQNVCHLDLKPANVLLRPDG-----SAVLLDFGLSFHAHYPDLLAEEMREAVGSP
86160117        LHDLHQQEAIHLDLKPANVVFRPTG-----EAVLVDFGLAHHAHYPDLLAEEFRRPIGSA
115525927       LADLHRQHVIHHDIKPSNIMFRESG-----EAVLLDYGLACSDVLPDLMQEEFRLPFGTA
27353958        LADLHRQNVIHHDIKPSSIMFRESG-----EAVLIDYGLSHHNHLPDLLQEEFRLPYGTA
124268216       LHDLHRQHVIHLDVKPSNILFRRAADGSEAEAVLVDFGLSRHDHLPDLLDEEFTLPMGTG
13475495        LAALHRQKVVHLDLKPENVVLANRG------AVLLDFGLARHEELPDFLGAESSVPMGTA
                   :*:*.. * *:** .:::   .      .**:*:**:     **::  *   . *:

121592911       TWIAPEQVVGVRGDLRSDIFAIGVMLYEMATGELPFGSPTTQGGMRQRLWMTPAPPRQYR
121610423       AWIAPEQVVGVRGDPRSDIFAIGVMLYELATGELPFGAPGTRSGMRQRLWMTPAPPRQHR
120612910       AWIAPEQVVGVRGDLRSDVFAIGVMLYELATGELPFGAPATRGGLRQRLWVTPRPPRQHR
86160117        PYIAPEQVVGARSDPRSDLFALGVVLYELATGRLPFGAPTTPGGLRRRLYRDPTPPRALV
115525927       PYMAPERLLGVRDDPRSDLFALGVLLYFFTTGERPFGETETMYGMRRRLWRDPPPPRKLK
27353958        PYMAPERLLGVRDDPRSDLFSLGVLLYFFTTGERPFGEGETLRAMRRRLWRDPHPPRALR
124268216       PYMSPEQIQFVRDEPRSDLYALGVMLYHLTTGERPFGQPTSIAGLRQRLYREPVPPRVLR
13475495        AYISPEQVLGERSDPASDLFALGCILFQLATGEEPFGRPATLAGMKRRLYHAPRSPRDIN
                .:::**::   *.:  **::::* :*: ::**. ***   :  .:::**:  * .**

121592911       PDLPEWLQEVILRCLEPEAAQRYPSAAHLAF---
121610423       ADIPPWLQEVILRCLEPEAANRYPSAAHLAF---
120612910       PDIPPWLQEVILRCLEPEAAQRYPSAAQLAF---
86160117        PALPEWFQELVLRCLEPDAGRRHASAAQVAF---
115525927       PDYPPWLQEVVMRCLEIEPAWRYPTAAQLLFDL-
27353958        ADYPPWLQEVVLRCLEIEPVRRYPTASQLAFDL-
124268216       PDCPPWLQEILLRCLEPRADQRHDTAAQLAFDLM
13475495        DAIPRWLEAIILKCMEVDRSQRYIEAAHILSDL-
                   * *:: ::::*:*     *:  *:::
```

Cluster No. 7 multiple sequence alignment

```
CLUSTAL 2.0.10 multiple sequence alignment


70606821        YKVIDVVGQGGNGYVVKAERESKYYAIKILSPENLSKS--------SFDTLFKESENLKE
70606951        YRVIEVIGLGGNGYVMKVEKNGLLYAMKVLSVNKFTNSLE------HFDNLLKESENLEK
15920911        YRVVSVIGSGGSGYVLKAEKDNVFYAVKVFSLSQLSRAQLTISASSSFDEMFKESETLKQ
15921136        YKISEIIGEGGGSYVLKGEKDNKYYAIKVLKVQPTKSQTVALR---DFIDLFKESNSLIE
                *:: .::* **..**:* *::.  **:*::. .  .           *  ::***:.* :

70606821        LSKNDKIVSIYGIYADSNYINSIIKGNAEAYFNYPPAIIMEFMDGGTIADLIN--EIRSS
70606951        LSKDPRLVSIYGSFVDKNNIQSALAGDYTSYYKYPPAIIMEFMEGGTLFDLISRVDLVQS
15920911        LSRNPKFVTILGFYIDSNNIKSALKGDVNTYYNYPPAIVMEFMEGGTAKDLLT-TSIIYS
15921136        LSNHEGLVKLFGIFVDINQIGSIMRGDGETYLRYPPSIVMEFMEGGTVKDLLK--FYYTD
                **..  :*.: * : * * * * : *:  :* .***:*:****:***  **:.      .

70606821        PYFQYIVKAIIREVGLALKYLHKRGYVHLDVKPRNIFLSVIPPKDEKLLLDQISSR-GII
70606951        KYWQYIVKLVIKEIAKALTFLHKRGYVHLDVKPQNIFLKEKINGEPEVVYKILSSTPGII
15920911        TYWPLIVKEIIKEIAYALDFLHSKGFVHLDVKPENIFFSRNLGNNPEEIYKNISSS---I
15921136        KKWYDLVRIILLRVSLALSHIHKSGYVHLDIKPQNIFFSETLPNNIADILSFLSVKPQVV
                  :  :*: :: .:. ** .:*. *:****:**.***:.     :   : . :*     :

70606821        KLGDLGSAVRVGEKITQATPAYSPPEQIEAVITGKGAQPSMDNYALGVTAYYLLTGNV-S
70606951        KLGDLGSAVRVGEKITQATPAYSPPEQIEAVITGKGAQPSMDNYALGVTLYKLLTMKN-L
15920911        KLGDLGSAVRIGERFYQATPSYSPPEQIEAIITGKGADPKMDIFALGMTAYVLLTGKNDN
15921136        KLGDLGSATKIGGKITQITPEYSSPKQIENAILGLGATTDMDIFSFGILAYHLLTGGKVS
                ********.::* :: * ** **.*:***  * * ** ..** :::*:  * ***

70606821        PITKYVERAVDLYLQNKFNDALEEIDNSKKVLEGFKPSLPINTLPELNRVIQGTILSDPT
70606951        DYVNYLDKAFDEYIKGDPSIAMKYINMAKMSMVNFKPKLPHNTLPELANVVQGTLVVDPK
15920911        PISDNLNKAIDAYMAGNVGEALKLIQNAKQILSSWRPILPQNTPSELTRVIVYSININPL
15921136        PTAKLIDETIELYNNNKIRDALLKIDEAKKVLESWNIDLPSGVPLSLEEVVKGCIKG---
                   . ::.:.: *  ..   *:  *: :*  : .:.  ** ..  .* .*:   :

70606821        KRLTSDDIVQIL
70606951        RRLTSYDIVKIL
15920911        SRPSAKQIVDLL
15921136        KINSMEEIVKKL
                   :  :**. *
```

## Cluster No. 8 multiple sequence alignment

```
CLUSTAL 2.0.10 multiple sequence alignment


13092623        YEVKGCIAHGGLGWVYLAFDHNVND----RPVVLKGLVHSGDAEAQASAVAERQFLAEVV
145220807       YEIKGCIAHGGLGWVYLAFDKNVND----RPVVLKGLVHSGDAEAQAIAMAERQFLAEVT
111019188       YEVQGCIAHGGLGWIYLAIDRNVSD----RWVVLKGLLHFGDAEAQAVAVAERQFLAEVA
54027318        YEIQGCLAHGGLGWIYLAIDRNVSD----RWVVLKGLLHAGDAEAQAVAVAERQFLAEVA
111020581       YEVLGCLAHGGVGWIYLALDHNVSD----RWVVLKGLLHSGDPEAQEVALAERRFLAEVT
29831918        YEVVGCLAHGGLGWVYLAVDRAVSD----RWVVLKGLLDTGDQDAMAAAISERRFLAEIE
86741120        YEIAGALAHGGQGWIYLARDPSVAEG---SWVVLKGLLDSGDREAQAAAIAERRFLASVD
86741498        YTVHGVIAHGGLGWVYAATDDNLGGDGVRAWVVLKGLLDAANPEARRIAEGERRILTTVS
                * : * :**** **:* * *  :        ******:. .: :*    * .**::*: :

13092623        HPQIVQIFNFVEHKDT-SGDPVGYIVMEYIGGRSLKRGS--KKGNVEK------LPVAEA
145220807       HPGIVKIYNFVEHEDK-HGNPVGYIVMEYVGGTSLKQATLLKHSSRIR------LPAAEA
111019188       HPSIVKIFNFVEHPRS-DGTPMGYIVMEYVGGHSLRDVLSTHEKPER-------MPVEQA
54027318        HPSIVKIHNFVEHPGR-DGVPIGYIVMEYVGGRSLRALLDDHRRPQR-------MPVTEA
111020581       HPSIVKIYNFVEHPGM-DGNPIGYIVMEYVRGRTLRDLLTELKEDAGPDDPKPMLPVEHA
29831918        HANIVRIYNFVEHLDQRTGSLDGYIVMEYVGGKSLKEIANDRRTPTGKRDP---LPVEQA
86741120        HPAIVRIFTFVEHAGT------SYIVMEYIGGTSLREVLCRRRADAGRPDP---LPVTQA
86741498        HPGIVKILDYVTHHGE------DYIVMEYVPGVSLAGLAEVGVAGPDGRSAP--PSAADV
                *. **:*  :* *         .******: * :*                    .. ..

13092623        IAYLLEILPALSYLHSIGLVYNDLKPENIMLTEE--QLKLIDLGAVSRINSFG---CIYG
145220807       IGFMLEILPALGYLHSLGLVYNDLKPENIMVTED--QLKIIDLGAVSRINSFG---YLYG
111019188       IAYVLEILPALAYLHSTGLVYNDLKPDNVMVTED--ALKLIDLGAVAGIEDYG---YLYG
54027318        IAYLLEILPALEYLHSIGLAYNDLKPDNIMVTED--QVKLIDLGAVTPLEAYG---NLYG
111020581       IRYLLEVTPALGYLHSMGLVYNDLKPENIMIGDDYDQLKLIDMGAVARIGDHG---YIYG
29831918        CAYGIEALEALGHLHSRNLLYCDFKVDNAIQTEN--QLKLIDMGAVRRMDDEES--AIYG
86741120        VAYLLAALPAFAYLHRNGLVFGDFTPDNVMLGRE--TPRLIDLDAIRRIDDGAVAGAGCG
86741498        IRYLLRVLPALGHLHRLGLVYCDLKPENVMVTAE--DVKLIDLGGARRLDDRVS--GYLS
                  : :    *: :**  .* : *:. :* :   :    ::**:..   :          .

13092623        TPGYQAPEIVRTG----------PTVATDIYTVGRTLAALTLN-LRTRNGRYMDGLP--E
145220807       TPGYQAPEIVRTG----------PTVATDIYTVGRTLAALTMR-LPTRKGRYLDGLP--E
111019188       TPGFQAPEIVKTG----------PTVASDIYTVGRTLAVLTLD-MPSDKGRYLDGIPSPE
54027318        TKGFQAPEIATTG----------PTVATDIYTVGRTLAVLTLD-LPMEHGRYRDGIPDPD
111020581       TQGYQAPEIATTG----------PTVASDIYTVGRTLAVVTLD-MPTAGGCYVDGLPTPE
29831918        TVGYQAPEVADVG----------PSVASDLYTVARTLAVLTFD-FQGYTNVFVDSLPDPD
86741120        TLGYQAPEVPTTG----------PSVASDLFAVGRTLATLILD-FRGNTSTYLHTMPPAA
86741498        TPGYRAPELDDDGERPAGIARTAPTVTTDIFAVARTLARLVLGRFPGFLGAYRHALPPRR
                * *::***:   *          *:*::*:::*.**** : :  :    . : . :*

13092623        DDPVLTTYDSFARLLHRAINPDPRRRFSSAEEMSAQLM
145220807       DNPVLEEYDSFGRLLRRAIDPDPRRRFQTAEELSSQL-
111019188       QAPLLAEYEFFHRLLLRATDPDPSRRFASAEEMAGQL-
54027318        QHQVLRRYEFFHRLLLCATDPDPARRFPSARAMSAQL-
111020581       EAPLLAEYEFYHRLLLRAIHPDPARRFASAEEMSTQL-
29831918        HIEVFRQYESFYRLLVRATDPDPVRRFASAQEMTEQL-
86741120        DHPVLARHESLYRFLLKATAPDPDRRFTGAEEMHDELL
86741498        AHAPLRDFESLDRLLRRATATDPDQRFQSTTELADELV
                    :  .:   *:*  *  .** :**  :  :  :*
```

Cluster No. 9 multiple sequence alignment

```
CLUSTAL 2.0.10 multiple sequence alignment


116749962       FRLDQFIARGAMGAVFKAFDAVLARTIALKLIPKEIEEGLSEAEVFTREESKKRLIQEAK
116749963       FRLDQYLARGAMGLVFRAFDTVLVRTVALKLIPKAIEEGLSKEELAAREEARKRLIQEAK
21222230        YRLTRRLGRGGMAEVFAAEDVRLGRTVAVKLL---------RADLAEDPVSKARFTREAQ
48477207        YSVIKKLGEGGKGIVYKCIDNNLNRIVALKLI---------KSESLDDE-SYSRIIREAE
                : : : :..*. . *: . *  * * :*:**:         . :      :  *: :**:

116749962       AAGRLAHPNIVTIHSYGE----TDEFQYICMEYVSGKTLTQVLSERKPLSEEEAVSIFVQ
116749963       AAGRLTHPNIVTIHSYGE----SEEFEYICMEFVTGETLAHMLRERKAVPIEDAIPIVEQ
21222230        SVAGLNHHAIVAVYDSGEDVVGGQSVPYIVMEIVEGRTIRDLLLNAEAPGPEQALIIVSG
48477207        TTARLSHPNIVSIYDMQK----EDNRFFMVIEYIDGKDLLDYIDN-KPLKLDEILSLIIP
                :.. * *  **:::.  :     :.  :: :* : *. : . : : :.   :: : :.

116749962       VLSALEAANKEEIIHRDIKPANIMITDENRVKVMDFGIAKLPS---LSMTVTGMVLGTPY
116749963       ILLALQTANQEKIVHRDIKPSNVMVTLDKRVKVMDFGIAKLPS---LSMTITGTVLGTPY
21222230        VLEALAYSHQHGIVHRDIKPANVIITNTGAVKVMDFGIARALHGAQSTMTQTGMVMGTPQ
48477207        ITSALSYAHKNGVLHRDIKPENIMVSKNGEPKLMDFGLAKALD--RPGITRAGTIVGTPA
                :  **  :::. ::****** *::::     *:****:*:        :* :* ::***

116749962       YMSPEQIAGKKVDIRSDIFSVGTVLYEVMTGEKPFVGESTATITYKIMALDPTPPAVINR
116749963       YMSPEQISGQKVDIRSDIFSLGAVFYEMLTGERPFEAENTATLAYKIVQVEPIPPKVLNI
21222230        YLSPEQALGKAVDHRSDLYATGCLLYELLALRPPFTGETPLSVVYQHVQDIPTPPSEVSD
48477207        YLSPESALGKESDNRSDLYSLGCVLYYMSTGRPPFTINDTIKLIYSHIHDYPAEPSKINK
                *:***.  *:  * ***::: * ::* : : . **  : . .: *. :   *  *  :.

116749962       NLSQPMADIIVKALAKNPDQRFQTPTEMMLAL
116749963       HIPSTVGSIISKALAKDPTLRYQAPSEMLRDL
21222230        ATPPELDGLVMRSLAKEPDDRFQTAEEMRGLV
48477207        DIPRQLDSIIMKLLRKNPDDRFQSADELLYAL
                  .  : .:: : * *:*  *:*:. *:   :
```

Cluster No. 10 multiple sequence alignment

```
CLUSTAL 2.0.10 multiple sequence alignment


111023174       YRLRSKLGGGGMGAVWLARDTLLHRDVAIKQVTTTAG-LDPDE---ARRVRERTMREGRN
54022871        YRLQSKLGGGGMGAVWLAHDRLLDRDVAIKQVLSTAG-LPEAE---AARIREQIMHEGRV
134103120       YRVQRRIGSGAMGVVWECVDERLHRTVAVKQLLLQPG-LDPGE---AEEARQRAMREGRI
134103121       ---EQQIGSGAMGVVWRAVDERLHRTVAVKQLLLQPG-YTPEE---TEEARQRAMREGRI
54025161        YRLAERIGTGAMGVVWRATDERLQRVVAVKQLLLAPG-LTREQ---AQDAKQRAMREGRI
54023812        YRLVERIGSGGTGVVWRATDERLRRSVAIKQIHIQPS-LPEAE---RDIMRQRAIREARN
21223154        YRLGDVLGRGGMGTVWRAEDETLGRTVAVKELRFPGN-IDEEE---KRRLITRTLREAKA
29831548        YRLGDVLGRGGMGTVWRAEDETLGRTVAVKELRFPTS-IDEDE---KRRLITRTLREAKA
145593410       YRLLSPLGQGGMGRVWKARDEVLHRNVAIKELVPPPS-LTDEE---RREMRERSLREARA
29833120        YRLLSPLGEGGMGTVWRAHDEVLHREVAVKEVRAPAG-LPAAE---IERMYTRLEREAWA
21223156        YRLGEAIGSGGMGRVWRAHDEVLHRTVAIKELTAALY-VSESD---QAILLARTRGEARA
29831550        YRLVDSIGSGGMGRVWRAHDEVLHRAVAVKELTAALY-VSESD---RPRLLARTNAEARA
21223285        YELLEPIGSGGMGEVWKAHDRRLRRFVAVKGLLDRRA-MTPDT---QKAAMQRARREAEA
29827516        FELEARLGGGGMGTVWRARDLLLHRSVAVKEVRPPDVDFAEYDPDGAHLLRERVLREARA
29831551        YRLDARIGRGGMGVVWRATDQLLGRQVAVKEL-AFDTSLSDEE---ARLQRERTLREARA
29831553        YRLLAKLGHGGMGTVWRAKDETVDREVAVKEPRVPDH-LPERE---RANVFERMRREARA
145593389       YSLRSAVGNGGMGTVWRAADTLLRRDVAVKEVVLPQG-LAPSD---RDAMYERTLREARA
86739075        YRLVARLGAGAMGTVWRAFDSVLETEAALKEIEFAGG-VAEAE---RADRVERALREARH
86740348        YRLVERIGSGGMGTVWRAHDDVLRVEVAIKEIRVSAD-LDDDE---RAAGVETAMREARN
29831162        YRLLRTLGAGGMGRVWLAYDAELACEVAVKEIALPDLPIDASE---PVQRIARARSEARN
86740837        YRLQDRLGAGGMGAVWRATDQMLRVDVALKEVSIPVD-STPGE---WTERIARARREGMN
134102348       YQVTGELGRGGMGIVWRAWDQVIGREVAIKELHLPDG-VPPAE---RQVYEERVLREART
86739395        YRLDTPIGRGGAGVVWRGEDELLQRPVAIKEILVPMA-GAQNE---RDAIRARVLREARA
86739396        YRLDGVIGRGGFGVVHRATDELLQRVVAVKEVRLPLG-ENADE---RGLTRERVLREARA
134100369       YRMDSRIGTGAMGAVWAGTDVLLHRPVAVKEVRLSPR-VPEEE---AAEFRERALREARS
134102978       YRLESRLGGGAMGTVWSAIDELLRRPVAVKEVRLPPG-MPEEE---AAELRERALREARA
119716210       YTLEREVGRGGMGAVWLGRDEVLGRQVALKRVGLAPG-VTTPD-------LARAEREARL
21222892        YRLLGRLGSGGMGRVYLGR-SAGGRTVAVKIVHPHFA--LDEE------FRARFRREVAA
                      :* *. * *           .*:*                          *

111023174       AAKLA-HSHSIAMYDVALEAG---EPWLVMEYLP-SRSLAQAMNIADTLPPLEVAQIGAQ
54022871        AAKLS-HEHAIAVYDVVLEAG---EPWLVMEHLP-SRSVARALGLVDTLPVLEVAQIGAQ
134103120       AARLQ-HPHAISVYDVAEDEG---QPVLVMEYLP-STSLAAMMSEHGPLPPREVARIGAQ
134103121       AARLQ-HQNAIVVFDVAEDEG---QPVLVMEYLP-SQSLASVISEKGLVPPQQVARIGAQ
54025161        AARLH-HPHAITVFDVAEEDG---QPWLVMEYMP-APSLAAEIAGGRTLPPHQVADIGAQ
54023812        AARFQ-HPNAIVVFDITEHDG---DPCLVMEYLK-SRSLAAVLSAQGTLPLTQVARIGEQ
21223154        IARIR-NNSAVTVYDVVEEDD---RPWIVMELVE-GKSLAEAIREDGLLEPRRAAEVGLA
29831548        IARIR-NNSAVTVFDVVDEDN---RPWIVMELVE-GKSLAEAIREDGLLEPKRAAEVGLA
145593410       IARLN-HVNVVRIFDVLRTDG---DPWIVMEYVA-SKSLQDTLAEQGPVSPARAVQIGLG
29833120        AARVA-NRNVVTVYDVAHEDG---RPWIVMELIR-GIALSDLLDAEGPLSPQRAAHIGAE
21223156        AARIN-HSAVVTVHDVLEHDG---RPWIVMELVE-GRSLADAVKEEERVDPREAARVGLW
29831550        AARIN-HSAVVTVHDVLEHDN---RPWIVMELVE-GGSLADAVKERGRVEPVEAARIGLW
21223285        LAKIE-HQNVVTVHDQIETAD---QVWIVMKLLE-GRSLADLLSRDRVLGVPRAAEIGLQ
29827516        LARVE-HPNVVTIHHIVDGGEG-TYPWIVMELVG-GGSLADRL-ARGPMTPAEAAHIGRG
29831551        VAQLS-HPNIIVVHDVVVDDE---RPYIVMELIG-GGSLADRISADGPVDAREAARIGID
29831553        AARLD-HPAVVNVHDVAVVDG---RPWIVMELVQ-GRSLGAAL-QEGTLGARETAKIGLE
145593389       AAAIQ-HPAVVQVYDVVTEGG---RPWIVMELLD-SRSLADMVIEDGPLAQRVVAKIGVS
86739075        AAKLRGHPHVVTILDVLLENG---LPWIVMELVP-SRSLFEVVRSDGPLPVAEVARIGTA
86740348        AARLRGNPHVVTVHDAVEHDG---LPWIVMELVR-AGTLATTVNRDGPLPPERAIQVGLA
29831162        AARLRGHPHVATVHDVVVHEG---LPWIVMEHVPDAIDLQAVVRRSGPLTPARTARIGVA
86740837        AARLRGHPGIVSVHDVVEDGG---LPWIVMDLIIPARSVADRLRGSGGLRPDETASIGAA
134102348       AGRLN-DPAVVTVHDVLAEAG---TTYIVMELVR-AVTLTELVAQRGPLAPEQAADLARQ
86739395        LARLH-SPAIVSVYDVVEEHQ---RHWIIMELVD-ADSLGDVIRNQGPLPFDQVAAIGLA
86739396        AGRLH-HPGAVAVLDVIDDGE---LPWIIMEYVD-GRSLATIINDRGPLPVEETCRIGIS
```

```
134100369       LAVVT-HPNVVMLYDVADDAG---GPFVVMELVP-AESLSSVLKRTR-LSHEQLAVFVDG
134102978       IAVVT-HPNVVTLYDVAREAG---EPFVVMELVP-SQSLAAVLDEHGPLDDHQLALIADG
119716210       AARLN-HPHVVAVYDLVIEGPDNTEQWLVMEYVE-GSTLAELLRRDGAMTPDRAAAFLGQ
21222892        ARRVG-GAWTAPVLDADPEAR---VPWVATAYAA-GPSLTAAVADGGPLPAHSVRALGAG
                   .        : .            :       .  :   :     :       .

111023174       VADALTAAHAAGIVHRDIKPGNILVADRGR--ELGTVKISDFGIARAK----------GD
54022871        VADALAAAHAVGIVHRDIKPGNILVADRGP--RVGFAKLSDFGISRGA----------GE
134103120       VAAALGAAHAAGVVHRDIKPGNILLGD------NGTVKITDFGISRAQ----------GD
134103121       VAGALAAAHMAGIVHRDLKPGNILLGD------NGMAKITDFGISRAI----------GD
54025161        AAAALAAAHAAGIMHRDVKPANLLVG-------NGTVKITDFGISRAV----------GD
54023812        VASALIAAHQAGIVHRDVKPGNVLLDD------HGTVKITDFGISRAT----------GD
21223154        VLDVLRSAHREGILHRDVKPSNVLIA-ED-----GRVVLTDFGIAQVE----------GD
29831548        ILDVLRAAHREGILHRDVKPSNVLIA-ED-----GRVVLTDFGIAQVE----------GD
145593410       VLGALKAAHKAGVMHRDVKPGNVLLG-ED-----GRVVLTDFGLATVP----------GD
29833120        VLAALRAAHTAGVLHRDVKPGNVLMA-ND-----GRVVLTDFGIATVE----------GS
21223156        VLRALRAAHTAGVLHRDVKPGNVLLA-DD-----GRVLLTDFGIAQIE----------GD
29831550        VLRGLRAAHSAGVLHRDVKPGNVLLA-TD-----GRVLLTDFGIAQVE----------GD
21223285        MAQGLRAVHEASVLHRDVKPGNVLVR-DG-----GQVVLVDFGIATFE----------GA
29827516        VLAALRAAHAAGVQHRDVKPANVLLRPDG-----S-PVLTDFGIAAIR----------ES
29831551        LLGALGRAHGAGVLHRDLKPANVLLEADS-----DRVVLTDFGIAQVA----------GA
29831553        VLGALEAAHAAGILHRDVKPDNVLLG-RH-----DRVVLTDFGIAQIE----------GE
145593389       LLGALEVAHAIGVLHRDVKPANVLIC-SD-----GRCVLTDFGVARMP----------TD
86739075        VLDALVAARAHGIVHRDVKPSNVLIGTDG------RVVLTDFGIATG----------DGD
86740348        VLDALVAGQRMGVLHRDVKPSNILLADDG------RVLLTDFGIATH----------AAD
29831162        VLDALTAGHRIGILHRDVKPANILLAPDASGDPYARVLLTDYGIALQPE--------SRE
86740837        VADALAFAHAKGVVHRDIKPGNILLAESG------RALVTDFGIAAH----------NDD
134102348       VLSALENAHAAGIVHRDVKPSNIMVADGR-------VKLADFGIAQT----------LDD
86739395        LTDALAAAHSAGVLHRDVKPGNVLLGRDG------RVRLTDFGIAATE----------GD
86739396        LAYALEAAHRLGVVHRDVKPSNVLVTADG------RARLTDFGIAVSQ----------GD
134100369       VAAALQAAHRVGIVHRDVKPGNVLLGKHG------QVKLGDFGISRNA----------AE
134102978       VASALEAAHRAGIVHRDVKPGNVLIGDDG------RIKLSDFGISRNI----------SE
119716210       AADALAAAHAAGIVHRDVKPSNILVAPDG------QVKLSDFGIARAE----------AD
21222892        LGEALAAVHELGLVHRDVKPSNVLLTLDG-------PLLIDFGIARATGGTSPTQSGGGT
                    *   :  .: ***:** *:::             : *:*::

111023174       SADTTNGVITGTPAYFSPEVARGQDPTEA--SDVFSLGSTLYTVVEGQPPFGIDSDSIAL
54022871        TSDEPDGIITGTPAYLPPEVARGAQPTAA--SDVFSLGATLYTAIEGQPPFGMDDDSDAV
134103120       VQVTKTGMLAGTPAYLSPDVAMGQEPTPA--SDVFSLGATLYAAIEGRPPFGLNENTLAL
134103121       VSVTKSGILAGTPAYLAPEVALGRDPAPA--SDVFSLGSTLYAAIEGEPPFGVDENAISL
54025161        VTVTATGFLAGTPAYLAPEVARGEDPSPA--SDVFALGATLYAAVEGAPPFGEGDNPLAV
54023812        ATLTETGLICGTAAYLAPEVARGADPTPA--ADVFSLGATLFHALEGEPPYGASANPLAV
21223154        PSITSTGMLVGAPSYISPERARGHKPGPA--ADLWSLGGLLYAAVEGTPPYDRG-SAIAT
29831548        PSITSTGMLVGAPSYISPERARGHKPGPA--ADLWSLGGLLYASVEGVPPYDKG-SAIAT
145593410       PNVTRTGMVLGSPAYIAPERARDGTAGPE--ADLWSLGATLYAAVEGKSPYART-SAIAT
29833120        SALTMTGEVIGSPEFLAPERALGRTPGPE--SDLWSLGVLLYAAVEGNSPFRHD-TPLST
21223156        STITRTGEVVGSVDYLAPERVRGHDPGPS--SDLWALGATLYTAVEGRSPFRRT-SPLTT
29831550        TTITRTGEIVGSVDYLAPERVRGHDPGPS--SDLWALGATLYAAVEGKSPFRRT-SPLST
21223285        DRVTRHGGIIGTPPYLAPELFAPAAPGPTSASDLWALGVTLYEMVEGRLPFGGN-EVWEV
29827516        TTLTATGSIIGTPDYMAPERVTGDRGGPG--ADLWSLAMMLYVAVEGHHPLRRG-TTLAT
29831551        TTLTESGSFVGSPEYTAPERMSGVRTGPE--SDLWSLGALLCTVLSGESPFRRD-SLGGI
29831553        TNLTDTGGFVGSPEYIAPERVLGQRPGPA--SDLWSLGVVLYAAAEGVSPFRRS-NTPAT
145593389       VQLTTPGMVLGSPHFISPERAMGQDFGPP--SDLFSLGVTLYTAVEGRPPFDKG-DPIET
86739075        PTLT-VTGVLGTPLYMAPERLNNQPATFE--ADLFSLGGTLYFAVEGRPPFERD-TFGAM
86740348        PTLTGGIGSGGTPAYMAPERLLGGPATLA--GDLFALGATLYFAVEGVSPFQRD-TLPTT
29831162        PRLTATAGILGTPGYLAPERARGEPPTPA--ADLFSLGATLYATVEGRGPFDRH-GEYAT
86740837        SRMT-AAGVVGTIAYVAPERLGGQPADGR--SDVFSLGVTLYQMVEGRLPFQAD-TTAGL
134102348       PRLTSSGAIVGSPSFMAPERIQGADASPA--GDLWSLGATLFFAVEGWMPFERQ-TTAAT
86739395        VTLTGTGALVGSPAYIAPERVRGSSGTPA--SDLWGLGATLYSAVEGQPPFEGP-ETYAV
86739396        PRLTSTGMVMGSPAYLPPERARGDAGSAA--GDRWGLGATLFTTVEGYPPFTGG-DPISV
134100369       STLTRTGIVLGTPAYVAPEIAQGEAPSPA--ADLWSLGATLYSAAHGRLPYESDSDPLIT
134102978       QTITRTGIMLGTPAFIAPEIASGDGVTAS--ADLWGLGATLFAAAEGRPPYDAGDDPVAT
119716210       ASLTQTGLVTGSPAYLSPEVASGQQATDA--SDVWSLGATLFHALAGHPPYEVGDNLLGA
21222892        ASLTSTGVSIGSPGYMSPEQILGKGVTGA--ADVFSLGAVLAYATTGQPPFPGD-SSAAL
```

```
                          *:  : .*:            .* :.*.  *     *  *

111023174       LHRVAKAEIYRPSKAG-PLTDTLLHLLEPDPARRPTMAEARDAL---
54022871        VQRAAMAQIIPPSRSG-VLTDALLHMMEPAPQRRPTMAEARDEI---
134103120       LHAVAAGKVDPPQQAG-PMADPLMAMMQNRVEDRPDMAQVREML---
134103121       LHRVARGQIEPPRQAG-PMTAALMQLLRPDPVDRPTMAQARNLL---
54025161        LHAVARGEIPPPRRAG-PLAPVLMRLLAADPAARPSMPEAARAL---
54023812        LYAAANGQVSQPRNAG-PATDFLLALLSPDPDDRPTMRVARDTL---
21223154        LTAVMTENLEEPKNAG-PLRDVIYGLLTKDPDQRLDDAGARAMLNKV
29831548        LTAVMTEPLEEPKNAG-PLRSVIYGLLAKDPEQRLDDAGARAMLNEV
145593410       LAALATEPPPPPKNAG-PLRPVLNGLLRKDPTDRITAEVAERLL---
29833120        LRAVVDEELPPPFRAG-PLAPVIEGLLRKDPADRVPAEQAEQDL---
21223156        MQAVVEEEATEPRYAG-ALAPVISALLRKDPAERPDATEAEHLL---
29831550        MQAVVGEEPGAPQYAG-ALAPVITALLRKEPAERPGSDEAEQML---
21223285        QANIQQAPDPVLRYAG-PLGPVIQGLLTTDPDRRLDAAGAEEMLRDV
29827516        LAAVLHDDVPHPVKAG-PLTPVLTRVLVRDPAARPDAEAFDRM----
29831551        LHAVVVDDIRPPAQAE-PILPVVRGLLERDPDRRLDAAEAARLL---
29831553        LQSVLNATPAAPAAAKGTLAQVINGLLNKDPARRPNAAEVRRLL---
145593389       MHSVVEDPPAPPQRSG-PLVPVLMGLLEKDPDRRLPVHTARAML---
86739075        LAAILLQPPAQAHRAG-ELAAVLDGLLEKDPGRRMTPARAHELL---
86740348        IGAVLHADPPPFLRGG-RLSAAIAGLLAKNPASRLRAEGAQALL---
29831162        LTALLGEEPTPPVRAG-ELGAVLHGLLIKDPVRRLAPEAVARG----
86740837        LSAILFEPPRPTVLAG-PLRPVLDAMLEKDPVVRLDAAAAARALASL
134102348       LNAVLNETPRATRPHG-VVGPVITGLLIADPKARFTAAQVRALL---
86739395        LTAVVEGRRRAFRLAG-PLRNLLSDLMDRPAEERPDVTEIRRRL---
86739396        LAALVQGRRQPFRLAG-PLIPVIDDLMAPHEASRPPLTVVRRRL---
134100369       LSAIIHGPVPQHQMSG-PLGEVLSGLMVKDPARRMPLHEVRRRL---
134102978       VTEVVRGPVPSSARPG-PVGEVIAGLMVKDPAERMSLTEVRRRM---
119716210       LYRIVHEDPPRLPEAG-WLAPLLLATMCREPADRWSMARVRDVL---
21222892        LYKVVHEEPNLDGLDDGELRELVASCLAKDPSARPAPAEVARRL---
                                      :   :      *
```

Cluster No. 11 multiple sequence alignment

```
CLUSTAL 2.0.10 multiple sequence alignment


1552570         YRLQRLIATGGMGQVWEAVDNRLGRRVAVKVLKSEF-SSDPEFIERFRAEARTTAMLNHP
13879059        YRLQRLIATGGMGQVWEAVDNRLGRRVAVKVLKSEF-SSDPEFIERFRAEARTTAMLNHP
31791192        YRLQRLIATGGMGQVWEAVDNRLGRRVAVKVLKSEF-SSDPEFIERFRAEARTTAMLNHP
13092427        YRLHRLIATGGMGQVWEAVDSRLGRRVAVKVLKGEF-SSDPEFIERFRAEARTTAMLNHP
120401052       YRLQRLIATGGMGQVWEGVDSRLGRRVAIKVLKAEY-STDAEFVERFRAEARTVAMLNHP
145221405       YRLQRLIATGGMGQVWEGVDSRLGRRVAIKVLKAEY-STDSEFVERFRAEARTVAMLNHP
126432629       YRLQRLIATGGMGQVWEAVDSRLGRRVAIKVLKAEY-STDPEFVERFRAEARTVAMLNHP
111020684       YRLIRLIATGGMGQVWEATDNRLNRRVAVKVLKSEF-SSDPEFVERFRFEARTTAQLNHS
54022045        YRLQRLIATGGMGQVWEALDTRLNRRVAVKVLKAEF-SADPTFRQRFRTEAQTTAQLNHS
134096663       YRLGQRIAVGGMGEVWKATDVRLDRTVAIKVLKAEL-CGNAEFLHRFRTEARTTASLNHP
19551292        YRLQWIIGHGGMSTVWLADDVVNDREVAIKVLRPEF-SDNQEFLNRFRNEAQAAENIDSE
23491869        YTLQWIVGHGGMSTVWLADDNVNDREVAVKVLRPEF-SDNTEFLSRFRNEARAAENIHSE
38232690        YALQWVVGNGGMSTVWLADDLRNQREVAIKVLRPEF-SDNEEFLSRFRNEALASEHIDSD
68535100        FELQWIIGRGGMSTVWLAHDVEQQRDVAVKILKPEY-TENEEFRARFRNEASAAQDLDSP
116668589       FQLTTRIAIGGMGEVWKAKDLILGRIVAIKVLKEEY-TGDPGFLQRFRAEARHTALLNHV
119963247       FQLTSRIAIGGMGEVWKAKDQILGRIVAIKVLKEEY-TGDPGFLQRFRAEARHTALLNHV
28493744        YRLIARIAIGGMGEVWKAHDEVIQRHIAIKILKEEY-VGSRDFIERFRTEARHAAIVNHV
119025057       YRLDSRLAQGGMGEVWKGYDIQLGRPVAIKALRGDLGVTQEAKLLRLRAEAHNSANLAHP
23325796        YRLDSRLAQGGMGEVWKGYDIQLGREVAIKALRSDV-TNAEAKLRRLRAEAHNSANLAHP
119714295       YRLDSRIATGGMGEVWRGTDTTLGRQVAVKLLKNEY-ADNPSFRTRFETEAQHAASLHHP
145592613       YRLDERIASGGMGDVWRGTDEVLGRTVAVKSLLPAL-LDDPDFAERFRGEARTMATINHP
145594644       YRLDERIATGGMGDVWRGVDLVLDRPVAVKVLLPAL-VCDPGFITRFRSEARIMAALRHP
21222257        YELGPVLGRGGMAEVYHAHDTRLGRQVAVKTLRADL-ARDPSFQARFRREAQSAASLNHP
29830881        YELGHVLGRGGMAEVHLAHDTRLGRTVAVKTLRADL-ARDPSFQARFRREAQSAASLNHP
50841673        YELQNLIGRGGMADVWKARDHRLGRDVAVKKLRTDL-ASDDTFQARFQREAQSAARLNHP
145592614       YQVGELIGYGGMAEVHRGRDLRLGRDVAIKMLRADL-ARDATFQMRFRREAQNAASLNHP
22777192        YKIIKKIGGGGMANVYLARDTILERDVAVKALRMEY-IHDQEFIARFDREAQSATSLSHP
119900063       FKILAKVGEGGMGNVFRGRDLMLERDVAIKSLRPEL-ACYPEVVERFRTEAIALARLQHQ
2911096         YLVQAKIASGGTSTVYRGLDVRLDRPVALKVMDSRY-AGDEQFLTRFRLEARAVARLNNR
13881919        YLVQAKIASGGTSTVYRGLDVRLDRPVALKVMDSRY-AGDEQFLTRFRLEARAVARLNNR
31793354        YLVQAKIASGGTSTVYRGLDVRLDRPVALKVMDARY-AGDEQFLTRFRLEARAVARLNNR
116670115       YRVQSRLARGGMSTVYLATDQRLERDVALKVLHPHL-VNDGNFLDRLGREAKAAAKLSHA
                : :   :. ** . *   . *    * :*:* :              *:  **     :

1552570         GIASVHDYGESQMNGEG--RTAYLVMELVNGEPLNSVLKR-TGRLSLRHALDMLEQTGRA
13879059        GIASVHDYGESQMNGEG--RTAYLVMELVNGEPLNSVLKR-TGRLSLRHALDMLEQTGRA
31791192        GIASVHDYGESQMNGEG--RTAYLVMELVNGEPLNSVLKR-TGRLSLRHALDMLEQTGRA
13092427        GIASVHDYGESHMDGEG--RTAYLVMELVNGEPLNSVLKR-TGRLSLRHALDMLEQTGRA
120401052       GIAGVYDYGETDIDGEG--RTAYLVMELVNGEPLNSVIKR-TGRLSLRHALDMLEQTGRA
145221405       GIAGVYDYGETDIDGEG--RTAYLVMELVNGEPLNSVIKR-TGRLSLRHALDMLEQTGRA
126432629       GIASVYDYGETDMDGEG--RTAYLVMELVNGEPLNSVLKR-TGRLSLRHALDMLEQTGRA
111020684       GIAGIYDYGEVRDATGD--STAYLVMELVNGEPLNAVLSR-VGRLAVPHALDMLEQTGRA
54022045        GIAGIYDYGETYDPSAG--ETSYLVMELVQGEPLNAVLSR-LGKLSVNQGLDMLEQTGRA
134096663       GIAAVHDYGETAAIPDGPEDTAYLVMELVEGEPLAATLAR-EGRIKAEHALDMLEQAGHA
19551292        HVVATYDYREVPDP-AG-HTFCFIVMEFVRGESLADLLER-EGRLPEDLALDVMEQAAHG
23491869        HVVTTYDYREVADP-AG-HTFCFIVLEYIRGESLADMLER-EGALPEELALDVMEQAAHG
38232690        NVVRTYDYREVTDD-MG-RTLCFIVMEYVRGESLADMLAR-KGRLEEDLALDVLEQAAHG
68535100        NVVRTYDSGEVEDPDNG-TVFCYIIMEYIRGESLADVLSR-ESSLPQNLALDVLTQTAAG
116668589       GIANVFDYGE----EEG---SAYLVMELVPGQPLSSIIEH-EQVLSPDRTLSMIAQTARA
119963247       GIANVFDYGE----EAG---SAYLVMELVPGHPLSGILER-EQVLSPDMTLSIISQTARA
28493744        GIANIFDYGE----EKG---IAYLVMELVDGEPLSAVLER-ERMLPVEEVLRIVVQISRA
119025057       NIAALFEYYEH---DGIG----FLIMEYVPSKSLADLYHEQNGPMDPIKLLPILIQTARG
23325796        NIAALFEYYEH---DGIG----FLIMEYVPSKSLADLFHS-KGAMDPIELLPILIQTARG
119714295       NIAAVYDFGEAPAADGSGVQRPFLVMELVDGQPLSALLRP-GAPMDPDAVRELLAQAADG
145592613       GVVDIYDFGSDQ-------QIAFLVMEYVEGDALSATLNR-VGRLTSARTMALVAQAADA
```

```
145594644       GVVQVFDCGADELPTGD--QANYLIMEFVTGEPLSRRIEA-AGRLDVAETMSIVEQAAQA
21222257        AIVAVYDTGE---DYIDNVSIPYIVMEYVDGSTLRELLHS-GRKLLPERTLEMTIGILQA
29830881        AIVAVYDTGE---DYIEGISIPYIVMEYVDGSTLRELLHS-GRKLLPERTLEMTIGILQA
50841673        NIAAVYDTGET-KDPATGLPVPFIVMELIDGHTLRDVLRD-GRKILPRRALEFTQGVLDA
145592614       AIVAVYDTGE--EQAPTGETLPFIVMEFVGGRTLKEVLGG-EGRFQPRRALEICADMCAA
22777192        NIVNIFDVGE-------EDQLLYMVMEYVDGMTLKEYIHQ-HGPIDVPEALDIMKQLTSA
119900063       NIANVFSFFA-------EDGQYYMVMEFVDGEPLNRLARR-RGALPWREAVALVVQALYG
2911096         ALVAVYDQGKDG-------RHPFLVMELIEGGTLRELLIE-RGPMPPHAVVAVLRPVLGG
13881919        ALVAVYDQGKDG-------RHPFLVMELIEGGTLRELLIE-RGPMPPHAVVAVLRPVLGG
31793354        ALVAVYDQGKDG-------RHPFLVMELIEGGTLRELLIE-RGPMPPHAVVAVLRPVLGG
116670115       HVVGVLDQGSDG-------HTAYLVMEYIKGHTLRDVIKE-KGALSPRLALALIDPVVEG
                 :.   .               ::::* : . .*          :       .      .

1552570         LQIAHAAGLVHRDVKPGNILITPTGQVKITDFGIAKAVDA--APVTQTGMVMGTAQYIAP
13879059        LQIAHAAGLVHRDVKPGNILITPTGQVKITDFGIAKAVDA--APVTQTGMVMGTAQYIAP
31791192        LQIAHAAGLVHRDVKPGNILITPTGQVKITDFGIAKAVDA--APVTQTGMVMGTAQYIAP
13092427        LQIAHAAGLVHRDVKPGNILITPTGQVKITDFGIAKAVDA--APVTQTGMVMGTAQYIAP
120401052       LQVAHSAGLVHRDVKPGNILITPTGQVKLTDFGIAKAVDA--APVTQTGMVMGTAQYIAP
145221405       LQVAHSAGLVHRDVKPGNILITPTGQVKLTDFGIAKAVDA--APVTQTGMVMGTAQYIAP
126432629       LQVAHTAGLVHRDVKPGNILITPTGQVKLTDFGIAKAVDA--APVTQTGMVMGTAQYIAP
111020684       LQVAHDAGVVHRDVKPGNILITPTGQVKITDFGIAKAVEN--SPITRTGMVMGTAQYIAP
54022045        LEVAHAAGVVHRDVKPGNILVTPTGQVKITDFGIAKAVDA--SPVTKTGMVMGTAQYIAP
134096663       LQAAHERGLVHRDVKPGNILITPNGKVKLTDFGIAKAADA--APVTRSGMVMGTAHYIAP
19551292        LSVIHRMDMVHRDIKPGNMLITANGIVKITDFGIAKAAAA--VPLTRTGMVVGTAQYVSP
23491869        LSVIHRMGLVHRDIKPGNMLITANGILKITDFGIAKAAAS--VPLTRTGMVVGTAQYVSP
38232690        LSIIHRMGMVHRDIKPGNLLITQNGQVKITDFGIAKAAAA--VPLTRTGMVVGTAQYVSP
68535100        LKAIHEAGLVHRDIKPGNLLITSDGFVKITDFGIAKAAAA--VPLTRTGMVVGTAQYVSP
116668589       LSVAHSQGLVHRDIKPGNLLITPDGRVKVTDFGIARLADQ--VPLTQTGQVMGTAQYLAP
119963247       LAVAHAQGLVHRDIKPGNLLITPDNRVKVTDFGIARLADQ--VPLTQTGQVMGTAQYLAP
28493744        LQAAHSAGLVHRDIKPGNILISESGLVKITDFGIARIGDQ--VPLTTTGQVMGTVHYLSP
119025057       LFVAHSHGVIHRDVKPANIMVSDSGEVKITDFGVSYSTNQ--EQITQDGMVVGTAQYISP
23325796        LFVAHSHGVIHRDVKPANIMVSDTGEVKITDFGVSYSTGQ--GQITQDGMVVGTAQYISP
119714295       LAAAHAAGIVHRDVKPANLLVTPDRQVKITDFGIARAAEG--IGLTGTGEVMGTPQYLSP
145592613       LHAAHLEGIVHRDVKPGNLLVRPNGTLVLTDFGIARSDLV--AQLTAAGSVLGTASYISP
145594644       LETVHRRGVVHRDIKPSNLVVQENGSVVLVDFGVARSTNV--TSITNTNAVPGTALYMAP
21222257        LEYSHRAGIVHRDIKPANVMLTRNGQVKVMDFGIARAMGDSGMTMTQTAAVIGTAQYLSP
29830881        LEYSHRAGIVHRDIKPANVMLTRNGQVKVMDFGIARAMGDSGMTMTQTAAVIGTAQYLSP
50841673        LSYSHAAGIVHRDIKPANVMLTREGYVKVMDFGIARAVADTSATMTQTAAVIGTAQYLSP
145592614       LEFSHRHQIIHRDIKPGNVMLTQTGQVKVMDFGIARALASGATTMTQTSAVIGTAQYLSP
22777192        IAHAHANEIVHRDIKPQNILINSTGQAKVTDFGIAMALS--ATALTQTNSILGSVHYLSP
119900063       LEHAHHAGVVHRDIKPSNMIVTADGTLKLMDFGIARILEK--VGLTRTGCVVGTLLYVSP
2911096         LAAAHRAGLVHRDVKPENILISDDGDVKLADFGLVRAVAA--ASITSTGVILGTAAYLSP
13881919        LAAAHRAGLVHRDVKPENILISDDGDVKLADFGLVRAVAA--ASITSTGVILGTAAYLSP
31793354        LAAAHRAGLVHRDVKPENILISDDGDVKLADFGLVRAVAA--ASITSTGVILGTAAYLSP
116670115       LGAAHAAGLIHRDIKPENVLIADDGRIKVGDFGLARAVTT--S--TSTGALIGTVAYLSP
                :   *   ::***:** *:::       : ***:           *    : *:  *::*

1552570         EQALGHDASPASDVYSLGVVGYEAVSGKRPFAGDGALTVAMKHIKEPPPP---LPPDLPP
13879059        EQALGHDASPASDVYSLGVVGYEAVSGKRPFAGDGALTVAMKHIKEPPPP---LPPDLPP
31791192        EQALGHDASPASDVYSLGVVGYEAVSGKRPFAGDGALTVAMKHIKEPPPP---LPPDLPP
13092427        EQALGHDATPASDVYSLGVIGYEVVSGKRPFTGDGALTVAMKHIKEPPPP---LPADLPP
120401052       EQALGHDATAASDVYSLGVVGYEAVSGKRPFIGDGALTVAMKHIKETPPP---LPADLPP
145221405       EQALGHDATAASDVYSLGVVGYEAVSGKRPFTGDGALTVAMKHIKETPPP---LPADLPP
126432629       EQALGHDATAASDVYALGVVGYESVSGKRPFTGDGALTVAMKHIKETPPP---LPADLPP
111020684       EQALGQDATAASDVYSLGIVGYEALSGRRPFVGDGAITVAMKHVQEAPAP---LPSDLPP
54022045        EQAVGEDATAASDVYSLGVVGYEALAGKRPFSGDGAITVAMKHVRETPPP---MPADVPA
134096663       EQALGSEATPASDVYSLAVVGYECLKGHRPFLSDNAVTVAMMHIREVAPP---LPPDVPP
19551292        EQAQGKEVTAASDIYSLGVVGYEMMAGRRPFTGDSSVSVAIAHINQAPPQ---MPTSISA
23491869        EQAQGHQVTPASDVYSLGVVGYEMLSGRRPFTGDSSVSVAIAHINEAPPQ---MPTSVSA
38232690        EQAQGRDVTAATDVYSLGVVGYEMLVGQRPFTGDSSVSVAIAHINQAPPA---MPTSVSA
68535100        EQAQGDQVGPASDVYSLGVVGFEMLAGYRPFSGESTVSVAIKHISETPPE---LPEEIDP
116668589       EQATGQTATGASDIYSLGVIGYECLTGHRPFSGESQIAIALAQVNDAPPP---LPETLPT
119963247       EQATGQTATGSSDIYSLGVIGYECLTGHRPFSGESQIAIALAQVNDAPPP---LPETLPT
28493744        EQAAGKKADAMTDVYSLGVVAYECLTGKRPFVGDSQVAIAVAHVGDTPAN---LPKDIPR
```

```
119025057           EQAQGKHATPQSDIYSLGVVAYEGLCGHRPFTGATPVDIAAAHVNNPVPP---LPDTVDV
23325796            EQAQGQQATPQSDIYSLGVVAYEGLAGHRPFTGTTPVDIAAAHVNNPVPP---LPDSVDV
119714295           EQAQGQTATPASDVYSLGVVAFECLAGRRPFVADTAVATALAHLREPVPP---LPESVPA
145592613           EQATGAVATPSSDVYALGVVAYQCLSGRRPFEGDNPLDIALRHVRDTPRA---LPADIPS
145594644           EQAAGRPVSGATDIYALGAVAYCCLSGSPPFTGDSPLQVAVAHLDEDPPE---LPHDIPE
21222257            EQAKGEQVDARSDLYSTGCLLYELLTVRPPFVGDSPVAVAYQHVREEPQAPSVFDPEITP
29830881            EQAKGEQVDARSDLYSTGCLLYELLTVRPPFVGDSPVAVAYQHVREEPQPPSVFDPEITP
50841673            EQARGETVDNRADIYATGCLLYELLVGRPPFIGDSPVSVAYQHVREIPAPPSSLDAEITH
145592614           EQARGESVDARSDVYAAGCVLFELLCGHPPFVGDSPVSVAYQHVREAPPTPSDLNPEVPP
22777192            EQARGGMATKKSDIYSMGIVLYELLTGKLPFSGQSPVSIALKHLQNNTPSVKKANPSIPQ
119900063           EQARGGDIDARSDLYSMAVVLYELLTGRAPFDSPSEFELMRAHIELPPPPPSGLVSGLPP
2911096             EQVRDGNADPRSDVYSVGVLVYELLTGHTPFTGDSALSIAYQRLDADVPRASAVIDGVPP
13881919            EQVRDGNADPRSDVYSVGVLVYELLTGHTPFTGDSALSIAYQRLDADVPRASAVIDGVPP
31793354            EQVRDGNADPRSDVYSVGVLVYELLTGHTPFTGDSALSIAYQRLDADVPRASAVIDGVPP
116670115           ELVLGRQADARSDIYSVGIMLYEMITGSQPFDGDVPIQVAYQHVNSSVEAPSAVVPGLAA
                    * . .      :*:*: . : :  :    ** .   .     ::             :

1552570             NVRELIEITLVKNPAMRYRSGGPFADAVAAV------
13879059            NVRELIEITLVKNPAMRYRSGGPFADAVAAV------
31791192            NVRELIEITLVKNPAMRYRSGGPFADAVAAV------
13092427            NVRELIEITLVKNPGMRYPSGGLFAEAVAAV------
120401052           NVRELIEITLVKNPGMRYKSGGPFADAVAAV------
145221405           NVRELIEITLVKNPGMRYKSGGPFADAVAAV------
126432629           NVRELIEITLVKNPGMRYRSGGPFADAVAAV------
111020684           NIRELIDITIAKDPSTRYASGGEFADAVAAV------
54022045            NVRELIEITMGKDPQLRYASGGEFADAVAAV------
134096663           GARALIEATLVKDPRRRYATGGEFANAVAAV------
19551292            QTRELIGIALRKDPGRRFPDGNEMALAVSAV------
23491869            QARELIGIALRKDPARRFADGNELARAVSAV------
38232690            PARELIGIALRKDPAHRYADGNELALAVSATRMG---
68535100            NLRELIRVCLRKSPRTRYADGAELATATVMV------
116668589           PVRALLMSMLAKDPKNRPANAIKLSEAAEAI------
119963247           PVRALLMSMLAKDPKNRPANAIKLAEAAEAI------
28493744            PVARLVLGCLEKQPQRRPKSAGNLADAASALLKDDLA
119025057           QLREFVMSMLAKDPLDRPKDALVVSRTL---------
23325796            QLREFVMSMLAKDPLDRPKDALVVSRTL---------
119714295           DLAAVVRRALAKLPQDRFADGAALAAALRD-------
145592613           QVRAVVERAMAKDPADRWPSAAALAGV----------
145594644           AVRALVRRALAKNPESRFGSGSAMAAAARAAV-----
21222257            EMDAIVLKALVKDPDYRYQSADEMRVDI---------
29830881            EMDAIVLKALTKDPDYRYQSADEMRIDI---------
50841673            QMDAITLKALAKDPADRYQTAKQMRDDI---------
145592614           AVDAIILKALSKNPLNRYQSAGEMRADL---------
22777192            SVENIVLQATAKDPFHRYNNIYELEEALET-------
119900063           GLEAVILRALAKDPADRFQHAEAFRSELEHL------
2911096             QFDELVACATARNPADRYADAIAMG------------
13881919            QFDELVACATARNPADRYADAIAMG------------
31793354            QFDELVACATARNPADRYADAIAMG------------
116670115           EVDELVQWCTARDPEQRPVDGNALLSELRHI------
                        .      : *  *      .
```

Cluster No. 12 multiple sequence alignment

```
CLUSTAL 2.0.10 multiple sequence alignment


126432846       YLLVELVGRGGMGEVWRAHDTATNRTVALKVLPAHLVHDTEYRVRFQREARAAARLSDPH
41407147        YRILELLGRGGMGRVYRAYDATTDRVVALKVLPPHLAEDQDFQQRFRREARIAAGLNDPH
54022953        YRLERPLGSGGMGQVWAAHDTRRGRRVALKLLPADLAADDGYRARFEREAELAATLRHPH
111021257       YVIERVLGRGGMGTVYLAQHPNLPRKIALKLLDTSWTNDDYVRSRFESEADHAAHLDHPN
108760681       WLVEQVHYRGPVSTLYRARHVRTGALAALKVLLPQPS-DVALR-RFRREAETLQRLRHPH
86158242        FEIEGVIGRGGFGEILSARRTRDGTAAAVKISQ---GSTPRARDQLLREAAALREIGPEL
                : :      * .. :  *         *:*:           : ::  **     :

126432846       IVPIHGFGEI-DGRLYVDMRLIEGRDLEHLLRE--GPLDPARAVMIIDQVAAALEAAHEV
41407147        VVPIHGYGEI-DGRLYVDMRLIEGRDLAHYITENGGRLSPQRAVAVIEQVAAALDSAHRA
54022953        IVPIHGHGAV-DGRLYIEMELVEGTDLGARLAAG-GPLDPPTALDILDQVAAALDAAHAA
111021257       IVTVHDRGRE-GGRLWIAMQYVPGSDARKALGS--GALDVERAVHIVSETGKALDYAHEA
108760681       IVDVLGYGTLADGRPFIAMEWLEGRDLAAELASR-GPLSPGEALEVLEQVGGALRTAHQA
86158242        APAVLASGMLDDGRAWVALERLTAPSLAERVRAEDAPLDLEALRDAVLALADALATLHGR
                   :   *   .** :: :. : .    :    . *.       :   . **   *

126432846       GLVHRDVKPSNILVG----KFDFTYLIDFGIARPIDDT-----GLTSVNRAVGTFHYMAP
41407147        GLIHRDVKPMNVLVTT---ARDFVYLIDFGLARAQADT-----ALTQTGATMGTVAYMAP
54022953        GLVHRDVKPSNILLR----PDGFAYLIDFGIARGVGQT-----SLTATGLTIGTWAYMAP
111021257       GILHRDVKPANILLAPG--DPERVLLTDFGTAKALDETH----QLTRTGMLVATLHYAAP
108760681       GVVHRDLKAQNVVRLSTGSGAPRVKLVDFGVAKGLTPDAPGASTLTLTGVSLGTPLSMAP
86158242        GWRHLDLKPRHVFVE-----PGGARLVDFGLAHGPASVAT---EDAPAGASAGTAAYMAP
                *  * *:*. ::.          . * *** *:            : ..   .*    **

126432846       ERFRDGRGDPRSDTYALACVLYQCLTGSRPFHGDSLEQQIAGHLMTPPPRPSTVQPGVPA
41407147        ERFT-GTTDHRADVYSLACVLHECLTGKRPFAGDSLEEQLNAHLNTAPPRPSATAPEVPA
54022953        ERFS-GHADARSDVYSLACVLFESLTGRRPYGDTDPAQQMHGHLMSDPPRAAAVDPTIGP
111021257       EQIEGEKLDHRVDIYALGCTFFHLLTNEPPYPGNTASSVMHGHLNGPIPQPSVVRPGLPT
108760681       EQIRGEPPDARTDLYAMGVLLFQLVTGQPPFQGATRHEVEDLHLNAPPPRPSERAP-VPA
86158242        EQVRGDAADARADVYAAGAILFELVTGAPPFEG-SPGEQRQAHLARRPGRPS-EHAGVPP
                *:.     * * * *: .  :.. :*.  *: .    .    **     :.:   . : .

126432846       KFDAVIARGMAKNAAERYRSAI-------------
41407147        AFDAVIARGMAKDPERRYQSVTELAEAARAAL---
54022953        RLDAVIAHGMAKDPAHRHASAGEFLRA--------
111021257       GVDAVVARALAKNREDRYSTCREFSDAV-------
108760681       ALDAVVLRSMGKRREDRYPDIDAWLDAL-------
86158242        ALEEVILRCLAKLPEARYADGSALRVALREAFASV
                 .: *: : :.*    *:
```

Cluster No. 13 multiple sequence alignment

```
CLUSTAL 2.0.10 multiple sequence alignment


86157501        FRIVRMLARGGMGTVYLAEHPVIGSRVAIKFLHESMAADAELVQRFYDEARAVNLIGHEN
86160067        YRVLSQLGEGGMGAVYLCEHSVLGRRFAVKVLRAERATDAELRERFRNEALAASRIGQEN
86156544        YRLVSLLGEGGMGAVYRAEHVQMGKALAVKVLRGDFARDPSAAERFRAEARIVSRLSHPH
108764043       FRVLRPLGSGGMGEVYLGEQVSLGRKVAIKVLHHDLHAQAGMAERFKREARLLSAVEHPA
116624752       YRILEKLGQGGMGEVYRAEDERLKRSVAIKVLRHHENRD--AALRFLQEARAASALNHPN
86743111        YELGEGIGYGGMAEVFRGRDIRLGREVAVKTLRPDLARDPTFLARFRREAQSSAALNHPA
                :.:   :. ***. *:  ..  :   .*:* *:     :     **  **     : :

86157501        IVGVYDLS---SLPSGRRYYVMELLEGETLAERLAR-GPLPRSVALQVLLQLCDALQCAH
86160067        VVDVLDFG---EEADGTLYYVMEALEGRSLGAVLREEGALPVGRALGLLDHVCRALAAAH
86156544        TIAVFDFG---ELPERGFYLAMEYVPGQDLARALREGGAFSEARAVGVAQQVLGSLAEAH
108764043       VVRIVDFG---ESGD-HACLVMEFVEGESLYDVLTP-GPMPPGRALPLLQQLAEGLAAIH
116624752       IVQIYD----LESHDGRDFIVMELASGRTL-AALVRDHPLRVEEALDYASQLASALAAAH
86743111        IVSVYDTGEDLINGAQIPYIVMEFIEGRTLRDALQTEGRFTERRAMEITSDVCAALDYSH
                 : : *              .**   *. *   *     :    *:    .:  .*   *

86157501        ERGVVHRDLKPENVFLVPRRGRPDFVKLVDFGIAKLR--------SATGHATGASGAIIG
86160067        ARGVVHRDVKPENVFVVRGPDGVERAKVLDFGISHVEQ-------PGRADRITRAGAIVG
86156544        EAGVVHRDMKPANVMLMQARPGEDFAKVLDFGIAKLR--------DEAGGATTGAGAVVG
108764043       DKGIIHRDLKPENVFISKSARG-EQARLLDFGIARLVE-------PDAASSVSQIGVVLG
116624752       AGGIVHRDIKPANIVVSE----SGTIKVLDFGIAKLEPHGPSGDSTETAAPETAAGSFLG
86743111        RMGIIHRDIKPANVMLSP----DGSVKVMDFGIAR--------ATTATSSTMTATAAVIG
                  *::***:** *:.:          :::****::             .      . .:*

86157501        TPEYMAPEQCEGGPVDARTDVYALGVMAYELLAGRRPFDSGPVQRLLLAHLREPPPPPS-
86160067        TPEYMAPEQAVGGPVDHRSDVYALGVLAYEMLTGALPIVGESAIATLVAHQTQAPEPPSR
86156544        TPSCLAPEQARGGPVDARADLYAMGCLLYELVSGRPPFTAASPVAVITAHLHDPPPPLRQ
108764043       TPEYLSPEQAVGAKVDTRSDLYSFGVLTYRVLSGRLPFDGPLPRNFLSQHASAAPLPLDR
116624752       TVAYASPEQAQGRPVDARSDIFSAGAVMYEMLTGTRAFDGDSTPGILSKVLRDQPRAICE
86743111        TAQYLSPEQARGARVDARSDVYTTGVLLYELLTGSPPFRGDNPVAVAYQHVREDPLPPSA
                *    :***. *  ** *:*::: * : *.:::*    .: .            * .

86157501        AFADLE--PDLEAAVLRALAKAPADRFQDMAELADAL
86160067        RRAGIP--PEVDALVLRALAKQPEGRFPSMLDF----
86156544        VAPGVS--PRLAEVVHRALRKDPAERFPSADAMRAAL
108764043       AAPTLSRYVGLLSLVMRLLEKDASKRPQSAHELADAL
116624752       LRAEVP--PAVARIVNRCLEKDIALRYPSGTELAADL
86743111        HDRDIS--PEADAIVLKAMEKDADDRYGTAGEMRDDL
                    :         * : : *    *      :
```

Cluster No. 14 multiple sequence alignment

```
CLUSTAL 2.0.10 multiple sequence alignment


111024860       FAEVSEIGHGGFGVVFRCREPSLDRTVAVKVLNSTFDE-DGYARFLREQRAMGQLSGHPN
111025675       FEDACIAGRGGFGIVYRCRQPALDRVVAVKVLSPDPDH-MDRARFLREQQAMGRLSGHPN
3261694         FDNVEEIGRGGFGVVYRCVQPSLDRAVAVKVLSTDLDR-DNLERFLREQRAMGRLSGHPH
111017776       FDDAQEIGRGGFGVVYRCTQEDLERTVAVKVLTVELDD-ENRARFFREQRAMGRLTGHPN
111020008       -----------------------------KVLTVELDE-ENRARFFREQRAMGRLTGHPN
111025634       FDDAQEIGRGGFGVVYRCTQADLERTVAVKVLTVELDD-ENRARFFREQRAMGRLTGHPN
111019707       FEDAHEIGRGGFGIVYRCVQPALDRTVAVKVLAADLDE-ENRVRFFREQRAMGRLTGHPH
111020362       FEDAQEIGRGGFGVVYRCTQAALDRTVAVKVLTADLDE-QNRERFVREQRAAGRLTGHPN
111026885       FEDAQEIGRGGFGVVYRCTQAALDRTVAIKVLTADLDE-QNRERFLREQRAAGRLTGHPN
111020359       FEDAQEIGRGGFGVVYRCTQAALDRTVAVKVLTADLDE-ENRARFLREQRAAGRLTGHPN
111023893       FEDAREIGRGGFGVVYRCLQISLARTVAVKVLIADLDE-LNRTRFLREQRAAGQLTGHPN
111026298       FADAAEIGHGGFGVVYRCRQTALDRTVAVKVLTGDLDE-NSRARFLREQRAAGRLTGHPN
111026453       FDDVHEIGRGGFGVVYRCIQSTLDRTVAVKVLTTDMDN-DNRARFLREQRAMGRLTGHPN
111022297       FDDAREIGRGGFGVVYRCTQPALDRTVAVKVLTNDLAE-ENRERFFREQRAMGRLTGHPN
111026426       FGDASEIGRGGFGVVYRCVQTVLHRTVAVKVLTADVGD-PNWKRFLREERVMGQLTGHPN
111019396       LEDAREIGHGGFGVVYRCAQPELDRTVAVKVLTAALDA-ENLERFLREQRAMGRLSGHPH
111027133       FDDAQEIGRGGFGVVYRCAQPALERTVAIKVLTTEVDR-DDRDRFVREQRAMGKLSGHPN
111022800       FDDAREIGRGGFGVVYRCLQTALDRTVAIKVLSSDLDG-EDRERFLREQRAMGKLSGHPH
54022251        ----MEIGRGGFGVVYRCLQTALERVVAVKVLPPDMDA-ESRERFLREEQAMGRLSGHPN
120402091       FVDAVEVGRGGGGVVYRCHQQSLGRTVAIKVLASSLDE-DDRERFLREGYAMGGLSGHPN
126437293       FTDAVEIGRGGGGVVYRCNQKSLGRSVAIKVLASDLDDKDDRERFLREGYAMGGLSGHPN
111017305       FAGAEEVGRGGFGVVYRCLQRSLGRIVAIKVLTSDLDP-ENRERFLREGYAMGGLSGHPN
                                             ***        .  **.**  . * *:***:

111024860       IVNVLRIGATASGRQFLVMQYHARGSLDAVLRSRGPLEWSAAVRIGVKLAGALETAHRAG
111025675       IVHVLQAGITYTGRPYIVMPFHRRDSLDSWITKHGALRAAEALAVGVKLAGALETAHRAG
3261694         IVTVLQVGVLAGGRPFIVMPYHAKNSLETLIRRHGPLDWRETLSIGVKLAGALEAAHRVG
111017776       IVNILQVGATDSELPYIVMPYHPQDSLDVRIRRHGPLPVEEALRLGVRMAGAVETAHRLG
111020008       IVNILQVGATDSGLPYIVMPYHPQDSLEARVRTRGPLPLDQVLRLGVKMAGAVESAHRLG
111025634       IVNVLQVGATDSGRPFIVMPFHPQDSLDARIRRDGPLPLGEALRLGVKMAGAVESAHRLG
111019707       IVSALHVGATDSGRPYIVMPYHSQDSLDVRIRRDGPLPLEEALRLGVKMAGALETAHRLG
111020362       VVNVLHVGVTDNGRPYIVMPYHAQDSLDTRIRRHGPLPLDEALRLGVKMAGALETAHRLG
111026885       VVNVLHVGVTDTGRPYIVMPYHAQNSLDARIRRHGPLSLDEALRLGVKMAGALETAHRLG
111020359       IVNVFHAGVTDNGRPFIVMPYHAQGSLDERIRRHGPLPLDEALRLGVKIAGALETAHRLG
111023893       VVNVLQVGVTDNGRPFIVTPYLAQGSLDARIHRDGTLPLEEVLRFGVKLAGALGTAHRQG
111026298       VVNVLQVGVTENDRPYIVMPFYAHDSLDAWIRRRGPLTLEDMLRVGVKIAGALEAAHGLG
111026453       IMSVLEVGATDSGRPYLVMPFHSQDSLDIRIRRHGPLTVEEVLRLGVKTAGGLETAHRAG
111022297       IVNVLQVGVTDSGQPFIVMPYHPQGSLDERIRRHGPLGVDEVLELGVKLAGALEAAHGEG
111026426       IVSALQVGVTEGGRPFIVMPYHEQGSLDDRIRRHGPLPLEEVLRLGVRLAGALETAHRLG
111019396       IVNIMQVGTTDTGRPYIVMQYHPHDSLDAQIRRRGPIPWSDALRVGVKLAGALETAHRVG
111027133       IVPVLQVGITDAGRPFIVMPYHSRDSFDAEVRRHGPLPWPDVLAIGVKLAGALETAHRLG
111022800       VVDILQSGVTRSGRPYIVMPYHSRNSLDAWIRREGPLPWSETLRVGVKLAGALETAHRLG
54022251        IVDILQVDVTASGLPFIVMPYCTHGSLEQLIHEQGPLGWADTLRVGVKLAGAIESAHRAQ
120402091       IVNILQVGMTEQDRPFIVMPYHARGSLADQVRREGRIPWPDALRIGVKLCGALETAHRTG
126437293       IVNILQVGVTDGNRPFIVMPYHARGSLAQRVRREGRIAWPEALRIGVKLCGALETAHRTG
111017305       IVNILQVGVTDAGRPYIVMHYHPRDSLAVQIRREGPLPWPEAISIGVKLAGALETAHRTG
                ::  :. .       ::*  :  :.*:   :   * :     : .**: .*.: :**

111024860       ILHRDIKPGNVLLTSYGEPQLSDFGIARIAGGFETDTGTITGSPAFTAPEVLSGDPPTLA
111025675       VLHRDIKPGNILLTEYGEPQLTDFGIARITGGEETTRGLVAGSPAYTAPELLSGSDASVV
3261694         TLHRDVKPGNILLTDYGEPQLTDFGIARIAGGFETATGVIAGSPAFTAPEVLEGASPTPA
111017776       ILHRDIKPANILLTDYGEPELTDFGIAHISGGFETATGAVTGSPAYTAPEVLGGEPPSPA
111020008       ILHRDVKPANILLTDYGEPELADFGIAHISGGFETATGAVTGSPAYTAPEVLGGEPPSPA
111025634       ILHRDVKPANILLTEYGEPELTDFGIAHISGGFETVTGAVTGSPAYTAPEVLRGDPPSPA
111019707       VLHRDLKPGNILLTDYGEPALTDFGIAHIVGGFETATGTVTGSPAFTAPEVLSGEPPTEA
```

```
111020362       ILHRDVKPGNILLTDYGEPALSDFGIARIAGGFETTAGVVTGSPAFTAPEVVTGEPPSAA
111026885       ILHRDVKPGNIMLTDYGEPALTDFGIAHIAGGFETTAGVVTGSPAFTAPEVVTGEPPSAA
111020359       ILHRDVKPGNILFTDYGEPALTDFGIAHIAGGFETTAGVVTGSPAFTAPEVVAGEPPSAA
111023893       ILHRDVKPGNILLTDYGEPALTDFGIAHIAGGFETTTAVVTGSPAFTAPEILAGESASAA
111026298       ILHRDVKPANILLTDYGEPALTDFGIAHISGGFETTAGVVTGSPAFTAPEVIAGGAPSPA
111026453       IVHRDVKPANILLSDFGEPVLADFGIAHVVDAFKTTTGTVTGSPAFTAPEVLSGDSPTPA
111022297       IVHRDVKPANILLTDYGEPALTDFGIAHISGGFETATGIVTGSPAFTAPEVLGGAAPSPA
111026426       IVHRDVKPANILRTDYGEPALTDFGIARIAGAFESATGVITGSPAFIAPEVLSGQAPSPA
111019396       ILHRDVKPGNILLTEYGEPQLTDFGIARMSGAFETTAGTVTGSPAFTAPEVLSGRTPTAA
111027133       ILHRDVKPANILITDYGEPQLTDFGIARVGGAFQTTTGTIAGSPAFTAPEVLDGAPSTAA
111022800       TLHRDVKPANILLTGYGEPQLTDFGIARVTGGFETTSSMITGSPAFTAPEVLRGDAPSAA
54022251        ILHRDVKPGNVLLSSYGEPQLTDFGIARIPGGFRTSSSMITGSPAFTAPEVLKGEEPTVR
120402091       TLHRDIKPANVLVNDYGDPQLSDFGTARIVGGYKTVTGFFTGTLSYTAPEVLTGKPPTVE
126437293       TLHRDIKPANVLVNDYGEPQLSDFGTARIVGGYKTVTGFFTGTLSYTAPEVLSGTPPTVA
111017305       TLHRDIKPANILLTAYGEPQLTDFGIAHIAGGYETATGGFTGSLAFTAPEVLNGESPTAR
                 :***:**.*:: . :*:* *:*** *:: .. .:  . .:*: :: ***:: *  .:

111024860       SDVYGLGATLFCLLTGHAAFERRSGEQIVAQFLRIAAESAPRLPAAGIPAD------VRA
111025675       TDVYGLGATLFTALAGRPAFARRRGEQVFAQLLRIGTEPLPDLRDIGVPEA------VCT
3261694         SDVYSLGATLFCALTGHAAYERRSGERVIAQFLRITSQPIPDLRKQGLPAD------VAA
111017776       ADVYGLGATLFSTLTGHAAFERRSGEQVVAQFLRITTQPVPDLREHGIPDD------VSA
111020008       ADVYGLGATLFSAFTGHAAFERRSGEQVVAQFLRITTQEVPDLRERGLPDD------VSA
111025634       ADIYGLGATLFSALTGHAAFERRSGEQVVAQFLRITTQEVPDLREQGIPED------VSG
111019707       SDIYGLGATVFCAVTGHAAFERHSGEHVVAQFLRITTQPVPDLREHGVPDD------VSE
111020362       ADVYGLGATLFAAMTGHAAFERRSGEQVVAQFLRIAAEPGPDPRKHGIPED------VST
111026885       ADVYGLGATLFAAMTGHAAFERRSGEQVVAQFLRITAEPVPDPRKHGIPED------VSM
111020359       ADVYGLGATLFAAITGHAAFERRSGEQVVAQFLRITSEPVPNPREHGISEG------VSA
111023893       ADVYALGATLFAAITGHAAFERRSGEQLVAQFLRISSAPVPDPREHGVLED------VSA
111026298       ADVYGLGATVFAAATGHAAFERRSGEQLVAQFVRITTAPTPDPREHGIADD------VST
111026453       SDVYGLGATLFTALTGHAAFERRSGEQVVAQFLRITTQPVPDLRIHGFADE------VSA
111022297       SDIYGLGATLFCAVTGHAAFERRSGEQVVAQFIRITTESAPDLRDQGLADD------VAT
111026426       ADIYGLGATLFCAHTGHAVFERRSGEQVLAQFLRLATEPAPGLREQDLPDD------VAA
111019396       SDVYSLGATLFSAITGHAAFERHNGEEVIAQFLRITTQPVPDLRTEGFPED------LSA
111027133       SDVYGLGATLFCLLTGHAAFERRAGEGVIAQFVRITTTPTPDLREPRIGPGRRSKWAARP
111022800       SDVYSLGAALFCLLTGHAAFERRSGERLVAQFLRIATQPVPDLRGEDIPDD------VCS
54022251        SDVYGLGATLFALLTGHAAFERQAGEKVVAQFLRITTQPVPDLREQDIPAP------VAE
120402091       ADVYSLGATLYAMIAGKAAHERNTDEELIAHYLRITSQPVPDLRHLGIPSD------VCS
126437293       ADVYSLGATIYALIAGNPPHERKADEDLIAHYLRITSTPVPDLRPEGIPVD------VCA
111017305       SDVYGLGATIFSLIAGRAAFERKTGEELVAQFVRISSQPVPDLRAQGIPDG------VCA
                :*:*.***::    :*.. . *. .* :.*: :*: :   *      .

111024860       AVESAMARDPGRRPGTAAEFGHQL-------
111025675       VIESAMARDPAERPATAADLGGALRRAGEHI
3261694         AIERAMARHPADRPATAADVGEEL-------
111017776       AIAHAMSREPDQRPATAADFGEEL-------
111020008       VIAHAMSREPGQRPATAADFGEQL-------
111025634       TIARAMSREPGQRPASAADFGEEL-------
111019707       IVDRAMAADPRQRPATAADFGDEL-------
111020362       IIERAMSGTSEGR-PSATELGQQL-------
111026885       IIEHAMSGTPASR-QSATELGQQL-------
111020359       VIERAMSAEAGAR-PSAVELGRQL-------
111023893       IIEHAMSGDPGRR-PSAAELGELL-------
111026298       VIEAAMANDPAGR-PSVEKLGQQL-------
111026453       AIEYAMSRTAADR-PSAAAFGERLQQIQAHL
111022297       VIEQAMAGRPEDRQPSAAVLGERL-------
111026426       AIERAMARAPQDRQASAAVLGEEL-------
111019396       VIQRAMSGKPEDRPASAAAFGDELR------
111027133       ELPSPTRSPTGQITPPTDTNAPHL-------
111022800       AIERAMSEEPTDRPASAAEFGQEL-------
54022251        AIERAMSPDPQQRPESAFEFGEML-------
120402091       AIEKAMSLESAARFDSAAEFGRAL-------
126437293       AIEKAMSREPADRYASAEEFGLELQL-----
111017305       VIERAMATNPAARPGSAEEFGHEL-------
                 :  .    .     ..   .  *
```

Cluster No. 15 multiple sequence alignment

```
CLUSTAL 2.0.10 multiple sequence alignment


17131350        YQIRQMLG-GGGFGKTYIALDTQRPGQPKCVVKHFQPVTHNPEFMETARRLFTSEAETLE
17133950        YQVIRVLA-IGGFGQTYIAQDTRRPGNPTCVVKHLKPATSDPRVFETAKRLFNSEAETLE
17130959        YSISSVLG-AGGFGRTYLAQDTQRPGNPTCVVKKLMPARKDTKFLQVARRLFITEAEILE
22298748        YKIQKVLG-SGGFGRTYLAQDTQRPGNPLCVVKHLRPGRTDERFMQVARRLFNTEAEILE
113475717       YKIIKPIG-KGGFGVTFLAKDISLPGAPICVIKELRPSSTKASELEMARKLFQREAETLG
17129783        FRVVRVLSDEGGFGRTYLSEDKDK-LNEPCVIKQLAPKFQGTWSQKKAVELFAEEAKRLQ
17132214        YRIIKVLSDEGGFGRTYLSEDIDK-LNELCVVKQFAPKVQEHSAMKKAVELFKQEAQRLQ
17133805        YRIVQILG-QGGFGRTYLAEDQRR-FNELCAIKELIPTATGTVAWEKAQELFHREAAILY
                : :   :.  **** *::: *        *.:*.: *        : * .**  **  *

17131350        RLGHHDQIPRLLAYFEEHQEFFLVQEFIDGHSLKAEMPP----NQPWTEKKVIILLQQAL
17133950        NLGHHDQIPRLLAYFDENQEFYLVQEFIDGHTLTEELIP----GNRWSEGQVTHLLQEIL
17130959        SLGQHQQIPALLAYFEVNEEFYLVEQYIAGHTLHEELPPV---AGLQSEAFVVEMLKGVL
22298748        KLGRHDQIPLLLAYVEENREFYLVQEFIDGVSLSEELK------RKHTEAEAIQLLREIL
113475717       KIGHHPQVPRLLDYFEWKKRFHLVQEYVSGLTLKQEVKK----QGIFTEEQGKKFLIEVL
17129783        ELGEHPQIPTLMAYFEQDNCLYLVQQFINGQNLLKELQQR----KNYRPGEIQAILLDLL
17132214        HLGEHHQIPTLLAYFEQDNYLFLVQQFINGNNLLQELRQG----VVYNESTIVEFLLDLL
17133805        QI-ENPQVPKFREKFEQDQRLFLVQDYVAGKTYWDILKERQAIGQAFTELEVLQLIRSLL
                 : .: *:* :   .: .. :.**:::: * .    :                 ::   *

17131350        DILQFIHLHKVIHRDIKPENILRRLHDGKLVLIDFGAVKEVQTQI---SAISGQTEMTVA
17133950        CILEFVHHQGVIHRDIKPDNIIRRTADNKLVLVDFGAVKQLRTQL---VTVGGQSSATVA
17130959        EVLAFVHEHRVIHRDVKPTNIIRSAEDNRLVLIDFGAVKLMQPPT---DQT--TELATVA
22298748        EVLNFVHSHYVIHRDIKPDNIIRRASDRKLVLIDFGAVKQIQPQE---AER--TQGSTVV
113475717       SVIDYIHTQGVIHRDIKPTNIIRRSEDGHLVLIDFGAVKDHVNQTYISGGTGETAFTNIS
17129783        PVLKFIHDRGVIHRDIKPENIIRCRTDGRLNLIDFGSSKQLTAKVQN---------FGTS
17132214        PVLKYIHERGVIHRDIKPQNIIRRQSDGGLVLIDFGAAKQLKATMQTQ--------LGTT
17133805        PVLEHLHGRGIIHRDISPDNIILRDSDSKPVLIDFGVVKELATRLQSPEMT----TPVTS
                 :: .:* : :****:.* **:   *    *:***  *

17131350        IGTPGYMSLEQFR-GKPRLNSDIYSLGIVCIQALTGVHPRELA-EDPVSGEVLWQNSAE-
17133950        IGTPGYMPTEQGQ-GKPRPNSDIYALGIIAIQALTGILPTQLQ-EDPQTGELIWRHLVN-
17130959        IGTRGYAPPEQFA-GHPRLASDIYAVGMMGIQALTGIPPQELP-PDPETGNVMWRSHAT-
22298748        IGTMGYAPPEQLS-GQPTLSSDIYAVGMIVLQALTGIKPRDLP-RDPRTGEVDWQQCVT-
113475717       IGTSGFSPPEQVL-LRPVYASDIYALGVTTIFLLTGKTPNNLG-YNQVNGEILWHSQVN-
17129783        IGSHGYSPLEQIRDGKAYAASDLFALGATCFHLITSVSPFQLWMEHGYSWVSNWRQYLHS
17132214        IGSLGYTPIEQMQYGKAYPASDLFSLGATCFHLLTGINPSNLFVEQGYSWVESWQQYWNT
17133805        VGKLGYSPSEQMQTGRAYPSSDLYALAVTAIVLLTGKEASELFDENQLSWT--WQRWVR-
                :*. *: . **    :.   **::::.   :  :*.  . :*   .  .    *:

17131350        -VSP-----VLASVLSKMVINNFKLRYQSATEVLEAL
17133950        -VSD-----RLAVVLNKMVRYHFKDRYQTASEALQAL
17130959        -VSE-----ELALILDKMVRYHFSDRYQSATTVLQDL
22298748        -VSE-----GLAVVLNKMVKFNFSDRYPSAKEALQD-
113475717       -VSD-----MFREILKKMLEVSVEDRFQSAEELLAAL
17129783        PLTP-----ELDFVLDKLLQKDLKHRYTSADEVIKDI
17132214        SNSDRNEGEYLVKVLNKLLETDIQRRYQSADEVMNDL
17133805        -VNP-----VFAQIINRMLSHAPGDRFQTAAEVSQAL
                  .       :  ::.:::      *: :*
```

Cluster No. 16 multiple sequence alignment

```
CLUSTAL 2.0.10 multiple sequence alignment


27359109        FDELGRIGDEGRNSVVLKVRDVCLDAIMAVKKIKKGSRALPNPAEYFNEARILYANPHPY
90022722        FHIRHEIGLEGKNSTVFTATDLQLNAEIVVKKMLKS--DFSDVNEYFKEASLLHLSSHPN
12514297        ---ISDLEEQGCFSKVYLAHDRHLAHDLVIKEIEKK--ENTNHDDYFNEARLLYKHAHPN
15800944        ---ISDLEEQGCFSKVYLAHDRHLAHDLVIKEIEKK--ENTNHDDYFNEARLLYKHAHPN
                      :  :*  * *  . *  *   :.:*:: *     .:  :**:** :*:  .**

27359109        VVQVNYACEDSSHVYIAMPLYKNGSLSKYMSERYLTLGEIVRFSCQFLSGLHNIHSNQLI
90022722        VVPIYYACQDDDHIFLAMPYFELGSLKKRIRESPISVREIIIWSTQVLSGLHNIHSKRLI
12514297        IVQVQYAAQCESNIYIAMPFYHNGSLNQLMKKNNLTSREIIRYSIQFLSGLYHIHSKGLM
15800944        IVQVQYAAQCESNIYIAMPFYHNGSLNQLMKKNNLTSREIIRYSIQFLSGLYHIHSKGLM
                :* : **.: ..::::*** :. ***.: : :  ::  **: :* *.****::***: *:

27359109        HFDIKPDNILLSDRNEAMLSDFGLAKFSDEDGFATPNQLYGTHMPPEAFE--GGEYTNLL
90022722        HFDVKPDNILFSKRGEALLSDFGLTKQTTFSGVAGQDRIYGNMVPPEAFS--TQNFNNQF
12514297        HFDIKPNNIMISNRNEAMLSDFGLSQLVNEESRAAPEFGYHFHVPPEYFSLSTNDYNFTY
15800944        HFDIKPNNIMISNRNEAMLSDFGLSQLVNEESRAAPEFGYHFHVPPEYFSLSTNDYNFTY
                ***:**:**::*.*.**:******::    .. *  :  *   :*** *.    ::.

27359109        DIYQAGLTMYRMCVGSDEFKRQVDTFREPDGTLGHTFIQAVQAGDFPARNAFPAHIHSKV
90022722        DIYQFGLTLHRMCVGDDIFYAEYATFIEGGDLNRHRFRHAVVNGQFPNKNDYPEHIPQAL
12514297        DIYQAGLTIYRMCVGHDNFERERSAFS-----TIEQLRESIINGCYPLK-EYPPHIHKKL
15800944        DIYQAGLTIYRMCVGHDNFERERSAFS-----TIEQLRESIINGCYPLK-EYPPHIHKKL
                **** ***::***** * *  :  :*        . : .::  * :* :  :* ** . :

27359109        KNVITKCMSPDPDDRYMSAIEVVNDL
90022722        INTIKKCLATDLKERYVSAVDVVNDL
12514297        ITIVNKCIHVDPNERYQSVLDVLNDL
15800944        ITIVNKCIHVDPNERYQSVLDVLNDL
                 . :.**:  * .:** *.::*:***
```

Cluster No. 17 multiple sequence alignment

```
CLUSTAL 2.0.10 multiple sequence alignment


1552569         YELGEILGFGGMSEVHLARDLRLHRDVAVKVLRADLARD----PSFYLRFRREAQNAAAL
31791191        YELGEILGFGGMSEVHLARDLRLHRDVAVKVLRADLARD----PSFYLRFRREAQNAAAL
13879058        YELGEILGFGGMSEVHLARDLRLHRDVAVKVLRADLARD----PSFYLRFRREAQNAAAL
13092426        YELGDILGFGGMSEVHLARDIRLHRDVAVKVLRADLARD----PSFYLRFRREAQNAAAL
41406114        YELGEILGFGGMSEVHLARDVRLHRDVAVKVLRADLARD----PSFYLRFRREAQNAAAL
120401051       YEVGEILGFGGMSEVHLARDLRLHRDVAIKVLRADLARD----PSFYLRFRREAQNAAAL
145221406       YEVGEILGFGGMSEVHLARDLRLHRDVAIKVLRADLARD----PSFYLRFRREAQNAAAL
126432628       YELGEILGFGGMSEVHLARDNRLHRDVAIKVLRADLARD----PSFYLRFRREAQNAAAL
54022044        YELGEIIGFGGMSEVHKARDLRLSRDVAIKVLRADLARD----PTFYLRFKREAQNAAAL
134096662       YEIGDTLGYGGMSEVHRGRDIRLGRDVAVKVLRADLARD----PTFQLRFRREAQNAAAL
119900197       YEILGVLGKGGMGTVYKAHDPVIDRTVALKTIRRELLDGGS-GESMVERFRNEARAAGRL
108757164       FRLVRRLGRGGMGAVYLGEHVSIGSRVAVKVLHAHLTMY----PELVQRFHAEARAVNLI
83644775        YEIVRELGRGAMGVVYLGRDPKISRQVAVKTLNYR-QFDSHRLTDLKARFFREAEAAGRL
90020763        YEIRSALGKGAMGQVYLGFDPRIARQVAIKTLNYN-QFDAAALPDIKQRFFREAEAAGRL
71282323        YQVEGVLGKGAMGIVYQGVDPKINRHVAIKTLQLSDDIDSPEFGEAKARFFREAQTAGGL
119897189       HTLHGELGRGAISVIYRGYDPDIGRMLAIKTLRREYAER----EDYRERFLAEARVAGTL
71906564        YRVVRELGKGATATVYLCDDPDSDRQVAVKVIRFGQDSAAMS-RRMRKLFQNEGMVSKRL
                . :   :* *. . ::   .      :*:*.:.                *  *.     :

1552569         NHPAIVAVYDTGEAETPAGPLPYIVMEYVDGVTLRDIVHTEGPMTPKRAIEVIADACQAL
31791191        NHPAIVAVYDTGEAETPAGPLPYIVMEYVDGVTLRDIVHTEGPMTPKRAIEVIADACQAL
13879058        NHPAIVAVYDTGEAETPAGPLPYIVMEYVDGVTLRDIVHTEGPMTPKRAIEVIADACQAL
13092426        NHPSIVAVYDTGEAETSAGPLPYIVMEYVDGATLRDIVHTDGPMPPQQAIEIVADACQAL
41406114        NHPSIVAVYDTGEAETPSGPLPYIVMEYVDGVTLRDIVHTDGPLPPRRAIEIIADACQAL
120401051       NHPAIVAVYDTGEAETPTGPLPYIVMEYVDGVTLRDIVHNDGPMAPQRAIEVIADACQAL
145221406       NHPAIVAVYDTGEAETPTGPLPYIVMEYVDGVTLRDIVHSDGPMPPQRAIEVIADACQAL
126432628       NHPAIVAVYDTGEAETPAGPLPYIVMEYVDGVTLRDIVHSEGPMPPKRALEVIADACQAL
54022044        NHPAIVAVYDTGEAEIDGGPLPYIVMEYVDGETLRDIVRGKGPLPPRRAMEIIADVCAAL
134096662       NHPAIVAVYDTGETDSENGPLPYIVMEYVDGRTLRDIVKSEGPLAPRRAMEVMADASAAL
119900197       NHPGIVAIYDFGED----ADVAYIAMEYVDGCGLGQFMKQDARMPVGDVVSIMVQLLEAL
108757164       GHENIVSIFDMDATP----PRPYLIMEFLDGAPLSAWVGT--PLAAGAVVSVLSQVCDAL
83644775        DHPNIVTVYDVGEEA----DLAFIAMDYIQGKSLNVYAKPGRLLSVEVVYMLMAKVADAL
90020763        NHPNIVAVYDLGEEP----DLAFIAMDYVDGSPLSAFVNADNLLPVFEVYRVIHDVAVAL
71282323        SHANIVTIYDVGEDN----HLGYIAMDLLTGAPLSLFTQADKILPTPLVYQLLIQITDAL
119897189       SHPGIVTIFDVGVSD----GEPFIAMELLQGPTLAAFVEQRGRLPVRTVIRIAIQIADAL
71906564        NHPNIVRVYEAVVEE----DFAYLAMEYVEGFSLEEHIRIDKLLPMHRVIGIIFKCCMAL
                .*  ** :::            :: *: : *  *         :.   .  :  .   **

1552569         NFSHQNGIIHRDVKPANIMISA----TNAVKVMDFGIARAIADSGNSVTQTAAVIGTAQY
31791191        NFSHQNGIIHRDVKPANIMISA----TNAVKVMDFGIARAIADSGNSVTQTAAVIGTAQY
13879058        NFSHQNGIIHRDVKPANIMISA----TNAVKVMDFGIARAIADSGNSVTQTAAVIGTAQY
13092426        NFSHQNGIIHRDVKPANIMISA----TNAVKVMDFGIARAIADS-TSVTQTAAVIGTAQY
41406114        NFSHQNGIIHRDVKPANIMIST----TNAVKVMDFGIARAIADSGNSVTQTAAVIGTAQY
120401051       NFSHQHGIIHRDVKPANIMISK----TGAVKVMDFGIARALADAGNPVTQTAAVIGTAQY
145221406       NFSHQHGIIHRDVKPANIMISK----TGAVKVMDFGIARALADAGNPVTQTAAVIGTAQY
126432628       NFSHQHGIIHRDVKPANIMISK----TGAVKVMDFGIARALADS-SSVTQTAAVIGTAQY
54022044        DFSHKHGIVHRDMKPANIMINR----SGAVKVMDFGIARALADSSNPMTQTAAVIGTAQY
134096662       DFSHRHHIIHRDVKPANIMITR----SGAVKVMDFGIARALTDGQAAVTQTAAVIGTAQY
119900197       EYVHLRGVIHRDIKAANLLITA----AGKLKITDFGIARIDTAN---LTQVGAIVGTPTA
108757164       QAAHARGIVHRDLKPDNIFLVRRKRNAPFVKVLDFGIAK-LADAHMPQTHAGIIVGTPEY
83644775        EYAHQKNIVHRDIKPANIIFNP---DNQQVKVTDFGIARISDKS---KTRTGQMLGSPLY
90020763        EYAHQHQIVHRDIKPGNIMYRP---VPYHLKVADFGIARLTDNS---QTTTGEILGSPLY
71282323        EYAHNQNVVHRDIKPANIIYDD---DLLKVTVTDFGIAYVSDNS---NTRTGIIMGSPYY
119897189       DYAHRQAVVHQDIKPDNIAVTS---LNGNVKVMDFGIARLRSGSAASRGTAHVIAGTPDY
71906564        DAAYRQGIIHRDIKPANIMVAA----NDEAKIADFGLALNMNKDMDRDSTFIMGVGSPAY
```

```
                :  : . ::*:*:*. *:            .: ***:*                 *:.

1552569         LSPEQARGDSVDARSDVYSLGCVLYEVLTGEPPFTGDSPVSVAYQHVREDPIPPSARHEG
31791191        LSPEQARGDSVDARSDVYSLGCVLYEVLTGEPPFTGDSPVSVAYQHVREDPIPPSARHEG
13879058        LSPEQARGDSVDARSDVYSLGCVLYEVLTGEPPFTGDSPVSVAYQHVREDPIPPSARHEG
13092426        LSPEQARGDSVDARSDVYSLGCVLYEILTGEPPFIGDSPVSVAYQHVREDPIPPSQRHEG
41406114        LSPEQARGDAVDARSDVYSLGCVLYEILTGEPPFTGDSPVAVAYQHVREDPVPPSQRHEG
120401051       LSPEQARGVKVDARSDVYSLGCVLYEVLTGEPPFVGDSPVAVAYQHVREDPVPPSQRHAG
145221406       LSPEQARGVKVDARSDVYSLGCVLYEMLTGEPPFVGDSPVAVAYQHVREDPVPPSQRHTG
126432628       LSPEQARGEKVDARSDVYSLGCVLYEILTGEPPFVGDSPVAVAYQHVREDPVPPSQRHEG
54022044        LSPEQARGETVDARSDVYSVGCVLFEILTGEPPFTGDSPVAVAYQHVREDPRLPSHVHHG
134096662       LSPEQARGESVDARSDVYASGCVLFELLTGEPPFTGDSPVAVAYQHVREDPRKPSDVNQQ
119900197       MAPEQFLGLGIDHRADLFASGVVFYELLTGRRPFSG-PIEAMAYQVCNVVQAPPSAQVAG
108757164       MAPEQSLGRGVDGRADLYALGVIAYQLLTGRLPFNDEGLAAQLVAHQLRPPPPPSSVYPA
83644775        MSPEQLKGGKVNGVSDIFSLGVTFYQLLTGEPPFNADSLPELTQQILNKKHRSVRELRPE
90020763        MAPEQLKGKKVDYAADLFSLGVTFYQLLSGELPFKGDNLASLTYEIIHGKHKSVRQVRKD
71282323        MSPEQILGLKVDGRSDIFSLGVTFYQLLCGHLPFEGESIATVAYQITKAKAIAVNQRNTN
119897189       MSPEQIRGKGIDGRSDLYSLGVVLYWLLAGHTPFHGDEVNELLRRILNDAAPPCRPLDPD
71906564        MSPEQIKGYPLNQKTDLYSLGVLLFQLLTGRLPFRANNQATLIYRIINTDAPAVTALNPN
                ::***  *  ::  :*::: *   : :* *. **

1552569         LSADLDAVVLKALAKNPENRYQTAAEMRADL----
31791191        LSADLDAVVLKALAKNPENRYQTAAEMRADL----
13879058        LSADLDAVVLKALAKNPENRYQTAAEMRADL----
13092426        ISVDLDAVVLKALAKNPENRYQTAAEMRADL----
41406114        ISADLDAVVLKALAKNPDNRYQTAVEMRADL----
120401051       ISPELDAVVLKALAKNPDNRYQTAAEMRTDL----
145221406       ISPELDAVVLKALAKNPDNRYQTAAEMRSDL----
126432628       VSRDLDAVVLKALAKNPDNRYQTAAELRSDL----
54022044        VPRELDSVVLKAMSKNPANRYQTAAEMRADL----
134096662       VPASLDAVVLKALSKNPANRYQSAAEMRADL----
119900197       LAPAFDAIAAKALAKRADDRYADAACFRL------
108757164       VSAALEHVILRALAKKPEDRYASIAAFRNAL----
83644775        LPASAVRITNKALQKDPAKRYANAGEMAEQL----
90020763        LPTSAARITNQALQKDASDRYSSAADMAAALNKAI
71282323        LPTSAMRICSKAMHKDIDKRFQSMSEF--------
119897189       TPDALLDVVRTLLAKDPAERYQSGAELIDDL----
71906564        LPEGLNAVLRRALEKDLYSRYRNGAEFAKDL----
                 .     :    : *   .*:     :
```

Cluster No. 18 multiple sequence alignment

```
CLUSTAL 2.0.10 multiple sequence alignment


6457717         YEVLERVGIGGMGSVYRAKRRQDGRVVALKVPQEK-YLADAKFVKR--FYREAEVLKRFT
94984835        YEVQERIGVGGMGSVYRARRKVDGRMVALKVPQEK-YLADAKFVKR--FYREAEVLKRFN
111020683       YELGEILGFGGMSEVHLARDVRLSRDVAIKVLRAD-LARDPTFYLR--FRREAQNAAALN
29831967        YRLVESIGQGGMGRVWRATDEMLDRQVAVKEMRIDGLDAEDTRTRRERTLREARATARIS
                *.: * :* ***. *  *     .* **:*  : .    : .   *    ***.    :.

6457717         HPNIVRVYDYRMQAPEH----YIAMEYLDGDSLEDLLERQ-PFTFSESVQMLRAMSDALR
94984835        HPNIVRVYDYRMEEGEH----YIAMEFLDGESLETLLEDR-RLSFEESTQVLRALADALR
111020683       HPAIVAVYDTGEAETEAGPLPYIVMEYVDGDTLRDIVRSEGPMAPRRAMEVISDVCAALD
29831967        HPNVVRVYDVVDESDRL----WIVMELVDGRSLERIVVEDGPLGPGETARIGRELVAALR
                ** :* ***      .     :*.** :** :*. ::     :   .: .:   :  ** 

6457717         HIHMQNVVHRDIKPANVMVLRGAFENGKLREGGIKLMDFGIA--VGKVLTRLTMPGARVG
94984835        HIHLHNVVHRDIKPANVMILKGAFVDGRLREGGVKLMDFGIA--VGKVLTRLTMTGARVG
111020683       FSHRNGIVHRDVKPANVMIN---------RAGAVKVMDFGIARAISDASSPMTQTAAVIG
29831967        QVHAGGVLHRDIKPGNVLVEN--------LGHRVVLTDFGIA--AIQDAKALTMVGMLVG
                  *  .::***:**.**::              : : *****    .  . :*  .  :*

6457717         TPIYMAPEQAKGNRVDARSDVYSLGLLAYEMVTGVTAFRG-SYEAVVHQQVFEQPKPPKQ
94984835        TPIYMAPEQAKGQRVDTRSDVYSLGLLAYEMVTGQTAFKG-SYEAVIHQQVFETPKPPKQ
111020683       TAQYLSPEQARGEQVDARSDVYSLGCVLFEILTGEPPFKGDSPVAVAYQHVREDPQTPSA
29831967        SPDYMAPERVSGRPQGPPSDVWSLGATLCAALGGHSPFSRSTTLATLHAVLYEEPELPTE
                :. *::**:. *.  .. ***:***      : * ..*   :  *. :  : * *: *. 

6457717         VNLEVPGKLSDLILHMIEKDPALR-PTLDEVIARI
94984835        VRLEVPGRLSDLILHMIEKDPDAR-PTLDDVIARL
111020683       VNPDIPRELDSVILKAMSKNPANRYQTAADMRSDL
29831967        A-----GGLRQILAALLEKEPSVR-PGLEEVAAAL
        .       * .::   :.*:*  *     :: : :
```

Cluster No. 19 multiple sequence alignment

```
CLUSTAL 2.0.10 multiple sequence alignment


113475397       YWIKSIIGEGNFCRTFLAVDELKPSKPFCVIKQFLSQAQGSENVEKA----AELFAEEGE
113475398       YWIKSLIREGNFCRTFLAVDEFQKSQPLCVIKQFILPYQKNE------------FAQEAE
113475316       YRPLNLIGQGGFGRTFLATDEDKPSKPYCVIKQFFPASQGTNNINKA----AQLFEKEAT
113477755       YRPLKIIGEGGFGRTFQAVDEDKPSKPFCVIKQFFPQAQGTKNLEKA----AKLFAQEAE
17134417        YRAIKPIGQGGFGRTFLAVDEDKPSKPRCVIKQFYPQAQGTNTVQKA----VELFTQEAV
113473984       YQVIKPLGESVWNHTFLAIDHDKPSKPPCIIKEFVNLDDIKDLVFQSQEVIPTFFSCDPV
                *   . : :. : :** * *. : *:* *:**:*    : ..            *  :

113475397       KLKLLGKHPQIPELYTYFTVDSRQYLVQEFIQGKTLEQELDNYGVFNESQIREFLIDLLS
113475398       KLKLLGRHPQIPKTYTYFTVDSRQYLVQEFIQGKTLKQELNNYGVFNEAQIRELLIDLLS
113475316       RLDQLGKHSQIPELMAHFTQDGRQYLVQEFIAGQNLAEALKIENSYNEDQIRGLLKNLLP
113477755       RLDGLGRHSQIPELLAYFTQNNRQYLVQEFIDGQNLKQELEESGAFSENQILELLKSLLP
17134417        QLDELGKHPQIPELLAYFTQDDRQYLVQEFIDGLNLAQELAHKGVFQETHIIQLLNDLLP
113473984       QLDKIGKHPHIPELLASIEHDGHHYLVQEYIEGSNLATELIEYGVFSESKIWQILNQLLL
                :*. :*:*.:**:  : :  :.::*****:* * .*   *   . :.* :*  :* .**

113475397       LLEFVHSYDVIHQDIKPENIIRRET----DKKLVLVDFRISKIIPKFQRFNVTGTVLNSA
113475398       LLQFVHSYDVIHQDIKPENIIRRET----DKKLVLVDFGVSNIIPKVQRNTVTLTITGSA
113475316       VLQFIHSHNVIHRDIKPENIIRRQNS---DRELVLVDFGAAKYALGTALL-KTGTTIGTP
113477755       VLEFIHSQQIIHRDIKPENIIRRRK----DNQLVLVDCGAAKYATMTALG-RTGTVIGSA
17134417        VLQFCHNRQVIHRDIKPENIILRNS----DNKLVVVDFGASKSATNTALN-HTGTSIGSP
113473984       VLELIHDHHLIHGDIKPENIICRRKQGTNTQEFVLVDFGVTISATGDLFS--IGSINGSA
                :*:: *. .:** ******** *..     .::*:**   :             :  .:.

113475397       EYSPPEQSVGKLKLASDLYSLGLVCLYLLTQMTPFDIYDVIEMEWVWRDFLNKTFISDNL
113475398       EYSASEKSIGKSHLASDLYSLGVVCLHLLTNIRPLNLYDVMEMEWVWRDSLNGNFVSDKL
113475316       EYTAPEQNRGKAVFASDLYSLGVTCLHLLTKISPFELFDVGEGTWVWRRYLQNNSISEKL
113477755       GYVAPEQSVGKASFVSDIYSLGVTCIHLLTQIEPFDLFDVSENDWVWRDYLQSN-VSNEC
17134417        EYVAPEQMRGRAIFASDIYSLGVTCINLLTGRSPFDSYDTNNDTWIWQQYLKPP-VSNHL
113473984       EYAAPEQTQGKVYPNSDIYSLGVTCLHLLTMVSPFELFDIKAEKWVWQDYLKHP-ISRHL
                 * ..*:  *:    **:****:.*: ***   *:: :*     *:*:  *:   :* .

113475397       GKVLDGLVELKLRKRYQNVQSVLDDL
113475398       GKVLDGLVEPKLRKRYQNVQSV----
113475316       GDILDKLVENAVNRRYQSADEVMENL
113477755       GEILDKMIVGATKKRFQNVGEILSVI
17134417        HKIINKMTASVPARRYQNVEEVLKDL
113473984       GRILDKMIQRDHRQRYQSAAEVIKDI
                  ::: :      :*:*.. .:
```

Cluster No. 20 multiple sequence alignment

```
CLUSTAL 2.0.10 multiple sequence alignment


6460339         YELLALLGEGGSAQVYRAQDGLLGREVALKVMHD-YLPESDR-SRFLREVRTLARLTHPG
94985497        YVLLALLGEGGSAKVYRARDNRLDRDVAVKVLHP-HLPEGDR-ARFLREVRILARLTHPG
108762507       YVLEDLAGRGGMGAVYRARDTLVGDVVALKLLELGASPAPEWLERFRREVRLARRITHRH
83815929        YRLDAVIGRGGMGTVYRATDQALEKTVALKVIAPHLADDDTFVRRFREEAKALARLDADG
                * *  : *.** . **** *  :   **:*::            ** .*.:   *:

6460339         VVPVLDLGQEP-EAG---RPFFTMPLLTGGPITRLGPLED-APGPLARFLTAAAFASRAL
94985497        VVSVLDLGETR-QANGTARAFFTMPLLTGGPITALGPLED-APGALAHFLTAAAFAARAL
108762507       VARTFDLGEHSG------RLYLTMEYVEGESLQTLMDREHTVPPARAARLVLALCEG--L
83815929        IVDVYTLRETE------EALFFVMEHVEGPSLETVLRRRGQLEPPQALSLLRQVLTA--V
                :. .  * :           ::.*  : * .:  :   .     . *  *      .  :

6460339         HHVHSHGIVHRDLTPGNVLLDDTGLPRIMDFGLVALSEQTRHLTRSGVTLGTPAYMAPEQ
94985497        GYIHAQGIVHRDLTPGNILLDDAGMPRIMDFGLVALSEQSRQLTRSGVTLGTPAYMAPEQ
108762507       AAAHAAGVVHRDLKPANVLVEARGRVVLTDFG-IARALAGEGASRTQGLVGTPMYMAPEQ
83815929        GHAHASGVLHRDLKPSNILIDADGQAVITDFGLAKILASDADLTATHDQLGTVAYMSPEQ
                   *: *::****.*.*:*::  *   : ***           : :   :**  **:***

6460339         AKGGG-VDARSDLYALGAVLYRVACGSPPFVGD-SDQSVLYQHVYEPVPDPRDLNPAVPD
94985497        ARGVG-VGPRSDLYALGAVLYRVACGSPPFVGD-SDQSVLFQHVYEKVPDPRDLNPAVPD
108762507       LESGE-VDARADLYATGLVLYQLLTGAPPFAGD-SPMAVAVARLRQPPPDPR-LLAAVPD
83815929        VKGLQNVDAASDLFAVGLLAYEVLTGRLPFDRSGSDFVVQRAIVDASFPPPSTHAPEVPP
                 ..   *.. :**:* * : *.:  *  **  . *   *    :    * *    . **

6460339         AVARVLLWLLAKRADRRPQSGAALAHLWA---
94985497        AVARVLLALLAKNPDDRPESGEAVAHLWA---
108762507       ALAELVLACLSREPSGRPRDAACVADALRQWL
83815929        AVEQVVLDLLSKDPAARPPDARAALDRL----
                *: .::*  *:: .  ** .. .  .
```

Cluster No. 21 multiple sequence alignment

```
CLUSTAL 2.0.10 multiple sequence alignment


116624299       YEIVALLGAGGMGEVYKARDTRLNRTVAIKVLS--ALPVADAERRRRLLQEARAASALKH
116625880       YEILALLGAGGMGEVYKARDSRLDRAVAVKVLP--SGKVADSDRKRRFIQEAKAASALND
116626667       YEVVAPLGAGGMGEVYKANDPRLARSVAIKVLP--ESLARDGDRLRRFEIEAKAAGGLNH
94969246        YEIRAPLGAGGMGEVYRAHDRRLDREVAIKVLP--SSVTDDPERLLRFEQEARAAGALNH
116624825       YRISGKLGEGGMGAVYRATDTKLDREVAIKILP--DAFAGDTDRMARFTREAKLLASLNH
116626062       YQITAKLGRGGMGQVYAAHDSELGRLVALKLLSGQTGSPGKMGSAERLIREAKAASALNH
119717310       YRIGARIARGGMASVYEATDIRLDRTVAVKVMH--PGLGDDDEFAARFVREARSAARLSH
                *.: . :. ***. ** * * .* * **:*::        .     *:  **:  . *..

116624299       PNIITLHDILTED-GRDGLVMEYVEGRSLDERIAGK-RLPLREALCYALQIAGALAASHA
116625880       PHIVAIYDISADA-GVDFIVMEYVPGKPLDQMIPQK-GLRTAETLRYAIQIADALAKAHA
116626667       SNILVVHDIGTEN-GVPYLVSELLDGESLRARLTLG-KIPTTRAVELARQIASGLAAAHA
94969246        PNILAVYQFATDPTGVSYLVTELLDGETLRERLKHG-PIPLRKAIEYGVQIANGLAAPHE
116624825       PNIAAIYGVEDRA-----LIMELVEGHDLRG------PLPTAIALNYARQIAGALDAAHE
116626062       PNIVTVYDVVRHG-SEVALAMELVEGQSMREVCGQ--PQPVGQVIHWARQIVQALAATHA
119717310       PNVVAVYDQGDDH-GTVFLAMELVEGVTLRDVIRKESPMPPARALALIEPVLSALAAAHR
                .:: .::           :  * : *  :              .:     :  .*  .* 

116624299       ANIVHRDLKPGNIMLSGEETGRGMVTVLDFGLAKFTGPESQEER------TQAMATGEGR
116625880       AGIIHRDLKPANLMVTVDDQ----VKVLDFGLAKLIEPSIGAEAAT---LTACDATEAGA
116626667       RGIVHRDIKPDNLFLTRDGR----VKILDFGLAKVTPGEVVTD------ATRTLHTSAGT
94969246        KGIVHRDLKPENLFITKDGR----VKILDFGLAKIGQAQSATVG-----PTLTRVTEPGL
116624825       KGIVHRDLKPANIRITPDGV----VKVLDFGLAKLAEPVSDSADPSNSPTMTSSHTRAGV
116626062       RGIVHRDIKPENVMLRPDGY----VKVLDFGLARRTRLLEGAPS--------ASASLFGF
119717310       AGIIHRDVKPENVLIAADGR----VKVADFGLARAVSAD-----------TQHTATG-GV
                 .*:***:** *: :  :      *.: *****:                     :  * 

116624299       IFGTVAYMSPEQAEGRKVDARSDIFSFGVVFYEMLTGRKAFLRTTTTSTRAAILGDDLGP
116625880       ILGTISYMSPEQAEGKSVDARSDIFSFGSVLYEMLTGQKAFQGDSKLSTLSAILREEPKP
116626667       VLGTVSYMSPEQVRGQTVDHRSDIFSFGAVLLEMLTGEKAFAAPTAADTMSAILNSDPAI
94969246        VMGTVGYMSPEQVRGQDVDYRSDIFAFGGILYEMITGKQTFDKPTSPDIMAAILNEDPRP
116624825       IMGTAAYMSPEQARGHKVDKRADIWSLGVVLYEMLAGRKLFGGDTVSDTLADVLKTDPDW
116626062       LGGTLSYMSPEQARGENPTQASDIFSLGSMLYELLCGVHPFQSASPIDTAYAIAHHEPKR
119717310       LIGTVSYLAPELVVDGRADARADVYAAGVVLFELLTGGKPHEGETPIQVAYKHVHEDVPA
                : ** .*::** . .      :*::: * :: *:: * : .   :  .        :   

116624299       ----APGIPESLQTLLSRCLRKDRDKRAQSMADI----
116625880       IAQIAADIPRDLGKIIQRCLRKDPDRRFQHM-------
116626667       SPEDEAAIPTAHLRTIRHCLEKNADDRFQTCKDL----
94969246        ISQVVPSTPPGLQRVVSRCLEKNPEQRFQSSSDL----
116624825       S-ALPPDTPPAIRRLLRRCLERDRKQRLADIADARLEI
116626062       AGSVRPEISAALSSLVAAMMAKDAADRPAAVELEQLL-
119717310       PSTLEPGIPAYVDALVARATARDRGLRPADAGVLLHQL
                     .  .      :     ::   *           
```

Cluster No. 22 multiple sequence alignment

```
CLUSTAL 2.0.10 multiple sequence alignment


116669855       FTYVSLLGSGGFSDVYLYEQDRPRRKVAVKVLLSDLKTEGARRRFESEANLMAQLSSHPY
119962082       FKYISLLGSGGFSDVYLYEQDRPRRKVAVKVLLSDLKTEGARRRFESEANLMAQLSSHPY
119026328       YTLIKRLGSGSEADVYLYQQLSPARQVAIKISKNTLDPR-AAVRFRSEANFMGQISTHPY
23326452        YDFVQMLGSGATATVYLYNQRMPARPVAVKASAKTLDPR-AAALFNREANFMAKLSSHPY
                :  :. ****. : ****:*  * * **:*   . *... *   *. ***:*.::*:***

116669855       IVTIFEAEVTEDGHSYLAMEYCSRPSLDVRYRRQRFSVDEVLAVGIQVASAVETAHRAGI
119962082       IVTIFEAEITETGHSYLAMEYCSRPSLDVRYRRQRFSVDEVLAVGIQVASAVETAHRAGI
119026328       ILSVYENGVTVNGRGYTVFEYAPGGNYKTFLEHGRLTADQMLTVGIDLASALFTAHRKGI
23326452        ILPVYGAGVTSDGHGYIVIEYAPGGTYNSIMKARSMTCEQVLDLGIKLSSALFTAHRSNI
                *:.::   :*  *:.* .:**..  . .   .   :: :::* :**.::**: **** .*

116669855       AHRDIKPANILVTDYNRPALTDFGISGTLGSDTDEDAGMSIPWSPPEQFRDGPVDGVMVD
119962082       VHRDIKPANILVTDYNRPALTDFGISGTIGADTEDDAGMSIPWSPPEQFRGGAVDGVPVD
119026328       IHRDIKPSNILINAHNMPMLADFGIAGTVYGRPG--VGFTIAWAAPEVLAQGGGGNESSD
23326452        IHHDIKPSNILITSQGLPVLGDFGISTDVYDRTE--TGFSPPWAPPEVLQHLTNGSEAAD
                 *:****:***:.  . * * ****:  :   .   .*:: .*:.** :     ..   *

116669855       VWALGATLYTLLAGRSPFVLP-------GADNSQRE-LISRITNSPVPRLGRADVPESLE
119962082       IWALGATLYTLLAGRSPFVLP-------GQDNSQRE-LISRITNSPLPRLGRADVPESLE
119026328       IYSLGATLFAMLIGQSPYEYFYADVLGDSQRQVRAERLKSVILNNPLPKLNRSDVPAEVE
23326452        IYSLAATLYAMFVGCSPFQH-------DYHPRSQSE-LISLIVNQPLPRLNRPDVPADVE
                :::*.***:::: * **:             . : * * * * *.*:*:*.*.*** .:*

116669855       LALATAMAKSAASRYSSAHAFALALQRI--
119962082       LVLATSMAKSPESRYSSAHAFALALQRI--
119026328       WVLRKALSRAPEDRYYSALEFARDM-----
23326452        AVLRRALNKDPDQRYYSALEFARAMQRVQF
                 .*  :: : . .** **  **  :
```

Cluster No. 23 multiple sequence alignment

```
CLUSTAL 2.0.10 multiple sequence alignment


109899780       YRVVRSLHVSARSHVYLVDDERTHKQWVLKTPSVDLSDSDEYLERFCLEEWIGRRIHNAH
83645934        YQILRCLQTSRRSHVYLALDIVSKTKVIIKTPATDQQGDPAYLERLLMEEWIARRINSLH
90022467        YIILRELHFTSRSRVYLAQDKQTKHNVVLKVLSTELSNTSASLERFLLEEWVARRINSSH
91774686        YHILRELHHSHRSHLYLAIDNATGQQVALKTPSTDLVANPAQLNRFALEEWVARRLNHAH
119897469       FTIVRELQVSARSHVLLATDKDSGRPVVLKAPSVDLRGDAAYLDSFVLEEWVARRVDSPH
120553124       YRIIRALHISSRSHVYLARDERSGKQVAIKFPAMELRQYPDELERFVTERWIAQRVQSPH
114798196       LRILRKLHANARSHVYLVLDPATHRRLVLKAPAAELCADPVLLRRFMMEEWVARRVSSPH
                  ::* *: . **:: *. *  :     :*  : :       *  :  *.*:.:*:   *

109899780       VLHVPPAEHPKQYIYCVAEYVEGQSLSQWLTDNPKPDLEVVRGIIEQVAKGLQAFHRLDM
83645934        VAKAAQFERPRAYLYTVSEYIEGRTLSQWLRDHPRPDLETVRGIVEQAARGLMAFHRMDM
90022467        VLKADLPDRKRNYIYTVFEYIEGQTLAQWALDNPKPKLEIVRDIIEQIAKGLQAFHRMEM
91774686        VLKPCKRERKPQYLYVAMEFIEGITLAQWMRDHPRPSLETVRGIVEQIAKGLQAFHRAEM
119897469       VIKAWPGERRRRHLYVAMEYIEGQTLAQWMTDHPAPPLEAVRAIVEQLAQGVQALHGRDM
120553124       VVAIFAPERPQSCLYLATDFVEGCTLRQWMLDHPQPDLETVRQLAEQIARGLRAFHRLEM
114798196       IVPAADLGRARSYLYVLLEFVDGQSLRQLMNDQPELPLETVRTLLEQIVAGVRALHRKEI
                :       :    :*   ::::* :* *   *:*   ** ** : ** . *: *:*  ::

109899780       LHQDIRPENIMIDRKGTVKIIDFGSTTVAGLQEIGQGCGNSELLGTALYMAPEYFLGDVG
83645934        LHQDLKPDNLMIDASGIVKIIDFGSTRVGGVAENDQGVRQPNLLGAALYAAPEYFLGEVG
90022467        LHQDLRPENIMLDKDGTVKIIDFGAVSVAGLNEAVLFNPDTYLQGTALYSAPEYFLGQPG
91774686        LHQDLRPENIMIDRNSVVKIIDFGAVRVAGLEESHPLESLP-VPGAIQYMAPEYFLGEPG
119897469       LHRDLRPENVMIDRDGTVKLIDLANVHVAGLAEGRGGAEQGAPPGTLQYMAPECLLGQPA
120553124       VHQDLKPENVLITPDGRAMLVDFGATRVAGLEETG-KPAACYPVGAALYSAPELFLGEPG
114798196       AHGDLRPENILIDGTGGVQLIDFGSAHVAGIADSAIALPPA-ALGALQYAAPELLLGEPP
                 * *::*:*:::   . . ::*:. . *.*: :           *:  * *** :**:

109899780       TSRSDIFSLAVLAYHMLSGRFPYATHVAKTRTRAQQNRLIYQSVRDEKSPVPAWLDATLA
83645934        SPGSDLYSLGVLTYHMLSGGFPYGADVAKTRTAGAQKRLTYKPVLSEERDIPAWIDATLK
90022467        SPRSELFSLGVITYFLLSGEYPYGTNVPKAKTISAQRNLWYNSLLSDDSEIPAWVDDAIR
91774686        SPQSDQFSLGVIAYQMLTGKLPYGAQAAKIKSRTQLQRLSYLSAIDGHNGIPSWIDDALA
119897469       STAADLFSLAAITYQMLCGQLPYGLSVTRVRTQQDLRNLRYVPLRHHRPDLPAWLDGVLA
120553124       SWQSDQYALGVIIYQLLTGRLPYHTDVPRIRTRKQLQALTYRPARAWREDLPPWLDATLA
114798196       GRASDQFAIGVIAYELLTGRLPYGAAGGKVRSDKNRRALTYQPATRAGQPLPAWINGALR
                   :: :::..: * :* *  **     : ::    . * * .       :*.*:: .:

109899780       KALKVDPFKRYAELSEFIHDL
83645934        RAVHPNPHKRYTELSEFLHDL
90022467        KAVHPLPDRRQEEIFEYI---
91774686        KAVHPEPHKRYPALSEFMHDL
119897469       KALQVAPAKRQEAVSEF----
120553124       RATHPEPHRRYPALSEL----
114798196       TATDPLARRRYEALSEFLADL
                 * . . :*   : *
```

Cluster No. 24 multiple sequence alignment

```
CLUSTAL 2.0.10 multiple sequence alignment


113474475       YQVLRILGQGGMGTTYLAWDQEPHNFQGMAVSPAAALVVLKEMNADMAQIRKAQELFERE
17129893        YQVLRTLGQGGMGTTYLAWDAA-----GVIVG-HPKLLVLKQMNADMARIAKAQELFERE
22298671        YHILRLLGQGGIGRTYLACEAQS---PGEWIGKVPQVRVLKELRSEMADNEKAQELFERE
37523676        YERVATLAQGGMSTTYLVFNHQN-----------DRLAVLKEIDADLSRKAKARELFLRE
                *. :  *.***:. ***. :                : ***:: ::::   **:*** **

113474475       AKALKLLSHPGIPKFFDFFVEEDKKYLVMEIIHGQDLEKRIITQGPILPQQAIGWMVQTC
17129893        AYTLKSLDHPGIPRYYDFFVEGGKKYLAMELVHGQDLEKRIYATGPVIPSLAIAWMIQTC
22298671        ARILQLLDHAGIPRFYDFFVEDGKSYLVMELIHGIDLERWVLRNGPVRIPQAVQWMIQTC
37523676        AQVLAELDHGGIPRFYDYFSSDERHYLVMEMIHGLTLEQVQPRS----AAQAAGWMIEAC
                *  *  *.* ***:::*:* .  : **.**::**  **:            *  **:::*

113474475       EVLEYIHSQEPPLIHRDIKPANLLVRRRDNQIVVLDFGAVKEIGTPLGTRIGAEGYSAPE
17129893        DILDYLHRQEQPLIHRDIKPANLMVRTANNQIAVLDFGAVKEIGTTPGTRIGAEGYCAPE
22298671        GVLDYLHNQEPPVIHRDLKPSNLLVRSRDNQIVVIDFGAVKELGHSP-TRIAVEGYSAPE
37523676        NVLVYLHGLQPPVIHRDIKPANLILRYNPREVVLIDYGAVKLAGGRQGTRIATPGYSPPE
                 :* *:*  : *:****:**:**::*   .::.::*:****  *    ***.. **..**

113474475       QDRGQPLTQSDLYAVGATLIFLLTAKSPMHFYDNHGNGYRFYIENVPNITPQLREVIEKV
17129893        QERGQPLTQSDLYAIGPTIIFLLTGENPFKFYRQKGRGFRFDVSKIPTITPKLRDVIERT
22298671        QMMGKPTLQSDLFAVGATLAFLLLGDHPIRFYHHRQGYHRLDVSDRPEIPDPVKEVIHRA
37523676        QGRGRPCLQSDIYGVGMTLVFLLTRQFPGRFYHPRER--RLVGLEEAGIEAPLAAVIAKA
                *  *:*  ***::.:* *: ***  . * :**  :    *:   . . *   :  ** :.

113474475       TQPLPRDRYKTAPALAEALNS--
17129893        TEPLPRDRFQSAKELAAAL----
22298671        TAPLMSERYPSAVALAKALRQAL
37523676        TAYLPQERHRDSQELAHALAPF-
                *  *  :*.  :  ** **
```

Cluster No. 25 multiple sequence alignment

```
CLUSTAL 2.0.10 multiple sequence alignment


119944475       YEILREIYISSRSHVFLALDSESGQTVVIKTPSTELRNNRQYLESFMLEEWIAKRLNNPH
71906459        YQIVREIKGSSRSHIYLAVDSESGEKVVIKTPSINLQGDPAYLERFLMEEWIARRINSAH
27351057        YRIVREIHGSSRSHIYLAVDAETEGPVALKLPSIDLRDNAAYLKRFLMEEWIARRIDSPH
83648458        YRVDRILHESARSQVYQVTDMETGERAVMKTPSLNFVDDPAYIERFTMEEWIARRVHNPH
90021924        LQVEKILHESERSQVYLVKD-ENGTPYVMKTPSINYDDDPAYIERFVMESWIGTRIQNQH
83646708        LVVEEEIFASSRSQLYLVRDQDSGERWVMKTPSRNYLGDVSYIDRFIQEEWIGKRIDNPH
                  : . :  * **::: . * :.    .:* ** :  .:  *:. *  *.**. *:.. *

119944475       VAKAIEPTRKRNFLYLVSEYIDGITLTQWMTDNPKPTINQVRKIIEQTAKGLQAFHRQEM
71906459        VLKPCLQTRKRHYIYVVTEFIEGQTLSQWMIDNPKPDLASVRGILEQAAKGLQAFHRLEM
27351057        VLKPMSQSRRRSYLYVATEFIEGQTLRQWMLDNPRPDLETVRGIVEQIATGLRAFHRMEM
83648458        VAGLVPCKRARSCLYYLTEFVPGAPLSRWIRDNSRSDIHRVTRILEQIVKGVRALHRKET
90021924        VVRIATPPESRSTLYYLTEHIAGPTLGQLIRERAPMDITDAVELVEQLAKGVRAFHRRDT
83646708        VVRIIEQPQARTCLYYLMEHVEGVTLQQWIKDHPFPKPKTAFALIAQVADGLSALHSQDT
                *       . *  :*   *.: * .* : : :..      .  :: * . *: *:*  :

119944475       IHQDLRPNNIMIDCNNVVKIIDFGSTFIAGVTDIKN---EEAVRGTMRYSAPEYFLGEVG
71906459        VHQDIRPDNIMIDSTGTVKIIDFGATRVAGVMEIATPIEQINLLGSATYAAPEYFLGENG
27351057        LHQDLRPDNVLIDKTGTAKIIDFGSVRVAGVAEAAPKSADEEILGTVQYTAPEYFLGEGG
83648458        LHQDIKPDNLLIGNDDHVTLVDFGSCRIPGVEEMYLPIERERALGTASYSAPEYVLGAKA
90021924        LHQDIKPDNVIVGRN-GAVIIDFGSCYVAGVHEVASSFERDKILGTLTYSAPEYRCGGNV
83646708        IHQDLKPANIIVSPDNRATIIDFGSVYVAGVAEVFRPLEHIAALGTASYSDPLYLLGQNT
                :***::* *:::.    . ::***:  :.** :           *:  *: * *  *

119944475       SSRSDIYALAVITYQMLSGRFPYHAEIAQVRSLSAQRRLIYKSLINDDIEFPIWIDDTLR
71906459        SSRSDIFSLGVIAYQMLSGRLPYGVEVAKSRTRAAQRKLVYQTVLNDEKEIPAWVDDAIR
27351057        SPRSDMFSLAAICYHMLTGHLPYGAQLAKIRGRSDAWKLRYRPAIDAERGIPGWIDGALR
83648458        TTRSELFSVAVIGYEMLTGHSPYGEAYEKAASPTALAKLKYTPAYHHNPMVPVWMDGALK
90021924        GMHSDQYSIAVILYEMLTGKLPFGEAYSEASSLKDFQKLKYHPARIHNPLVPVWLDKALE
83646708        GARGDLYALATIAYEMFTGELPYGDQINECRSRFDYDRLRYRSAMQFNPVIPLWFDRALQ
                  :.: :::..* *.*::*. *:     :        :* * .    :  .* *.* ::.

119944475       KALNVEPLKRYEELSEFV---
71906459        KAVDPDPFERYEELSEFV---
27351057        KALHPDPYKRHEDLSEFV---
83648458        KALQLDPRRRYDSFSEWLQDM
90021924        KALSLQTSARYPALSEWL---
83646708        KGVAFDLDQRYQTMDALLQDL
                *.:  :   *:  :.  :
```

## Cluster No. 26 multiple sequence alignment

```
CLUSTAL 2.0.10 multiple sequence alignment


54026216        YELRSLLGKGGMGEVYEAFDTSRHRLVAVKLLADELAKDPVYQERFRRESQAAARLAEPH
86742304        YVLHEILGQGTTGQVWRGAAVSDGAPVAIKVLRPELADDPEIVERFLREWDLLIDLDSPD
29831644        YTANQILGRGSAGTVWLGEGP--EGPVAIKLLREDLASDQELVGRFVQERTALLGLDHPN
50843112        YVLDDVLGRGSMGTVWRGHDLNDGSPCAIKILNPTLTTDKGATRRFIDERDIFMSVDDDA
                *   .:**:*  * *: .         *:*:*   *: *     **  *      :

54026216        VIPIHDWGVLDGVLFIDMRLVAGTDLRTMLHHT--GPLEPERAVRLVEQVAAALDAAHAD
86742304        LVAVRDLVNEPDVLAIVMDLVDGPDLRTHLREF--GPRPVEEAVRLVVGTLWALDSVHAA
29831644        VVSVRDLVVDGNDLALVMDLVRGTDLRTRLDRE--RRLAPEAAVAIVADVADGLAAAHAA
50843112        VVRVTDMVVESSTFAIVMELVDGPDLAQVLKSLPDHRLDQGTAVSLGIEVCQALAAIHRV
                :: : *     . : : * ** *.**   *            ** :   .  .* : *

54026216        GLIHRDVKPANILVTDADFAY--------LADFGIAHTEGDSAITQVGMAVGSYIYMAPE
86742304        GVVHRDVKPENILIDTSDPAHP----IVRLTDFGIAQMINGSSRTSVTGPIGTPLYMAPE
29831644        GVVHRDVKPENVLLDMQGPLGPGGSHPALLTDFGVAKLIDSPRRTRATKIIGTPDYLAPE
50843112        GIRHLDIKPANILIEDPENVSG-----ARITDFGVSEVVAFHG---PREVVGTPYYQAPE
                *: * *:** *:*:               ::***::.             :*:  * ***

54026216        RFDSGTVTGRADIYSLACVLHECLTGAAPFPSASMNVLVKAHLSEPPPRASALRPGLPAS
86742304        LSTGAPPTPAADVYSAGVVLYELLAGSPPFDSPNPAELLQAHREKQP----QPIQGVPAP
29831644        IIEGLPPRASVDIYALATVLYELLAGFTPFGGGHPGAVLRRHVTETV----VPLPGIPEE
50843112        VAAGWTPTAASDLYSFGVTLYEILAGHMPFQANSAGNLLRNQ---PP----PAIPGVDPR
                   . .     *:*: . .*:* *:*  ** .     ::: :             *:

54026216        IDAVIARGMAKNPADRF-ASALEM--------
86742304        VWGVLAGMLAKSPRGRP-VSAADAAEDLVEAL
29831644        LWQLLVQCLAKAPASRLRASELGARL------
50843112        LWNTILTCLAPDPSQRPDSAEVLASELR----
                :   :   :*  *  *   :
```

Cluster No. 27 multiple sequence alignment

```
CLUSTAL 2.0.10 multiple sequence alignment


116669657       YRLGEVIGRGGMASVYSARDENLGRDVALKLFAPQSADADELKRQEAEIQLLATLNHPSL
119964125       YQLGPVLGRGAMSTVYRAKDLLLGREVAIKIFLPGSGDDSFRGRQENEMRLLAGFDHPGL
116668760       YRIEALIGRGSQSGVYRAFDELLQREVAVKLFRDDPGNLDHTRRQGEEVRILAGMSHHAL
117927604       YELRTLVGHGSMGEVYAAYDRRLDRVVAVKVLRRDTTTASAAERLRQEMRVLAQLDHPHI
                *.:  ::*:*. . ** * *  * * **:*::   .   .   *   *:::** :.*  :

116669657       VTLFDAGIDSRVPEEPRPFLTMELVEGQDLRSRIRHSPVPLDELAVIGAGVADALAYVHS
119964125       VAAFDAGVDTRNDEE-RAFLVMELAEGRDLRAVLSDGPLTVPETARIGIRIAGALSQVHE
116668760       VTLLDAGADFSDPRHRRTYLVMELVRGPDLRARAAQGPINAAHLALIGHDLADGLAYIHH
117927604       VDLLDAGFR-----DHTTYLVLRWIDGQSLAEVLTHGPLDVTRTAAIAAQAADALAYLHS
                *  :***       .  .:*.:.   * .*     ..*:   . * *.   *..*: :*

116669657       LGIIHRDIKPANILLVQVR-PGEPLRPKLTDFGIARIVDGTRLTATGTMVGTAAYLSPEQ
119964125       HGVIHRDIKPANILVSDD--DGLAQTVKLADFGVALVMNDNRLTATGFTVGTAQYLSPEQ
116668760       HGIVHRDVKPANILLVDYNNDDRRPRAKLSDFGVAMILGDGNRTGPAESSGTPQYLSPEQ
117927604       RGIVHRDVKPANILLDAAD------YAYLTDFGIARFADATRLTATGQTIGTASYLAPEQ
                 *::***:******:             *:***:* . .  . *...   **. **:***

116669657       ARGADLGPASDIYSLGLVLLECIKAAVEYPGSAIESAVARLHRAPEIPGDVPAEWATLIR
119964125       AQGLSLTPASDIYSLGLVLLECLTGRAEYPGTPIETASARLHRAPQVPVELPGPLRELLE
116668760       AASEPVGPPSDVYSLGLVLLEGLTGNPVYPGAPIASAMARLLRDPHIPDVVAPKWAALLS
117927604       VRGGAVGPAADVYALGLVILECLTGRREYDGTPVEAAVARLTRPPRIDVGTPPGLRPMLA
                . .  : *.:*:*:****:** :..   * *:.: :* *** * *.:    .     ::

116669657       SMTALEPMDRPTAPDLETAL
119964125       AMTDMDPEKRPTAQHVEQAL
116668760       SMLSREPSARPGARDVSLAL
117927604       AMTATDPAARPSAVQVARFL
                :*   :*  ** * .:   *
```

Cluster No. 28 multiple sequence alignment

```
CLUSTAL 2.0.10 multiple sequence alignment


145590326       YKVIRLLGVGGFSYVLLVKFKKSMYAVKILRFADDQGAPLAGDENILYLFGQEMNRYLEL
18312200        YKVIRLLGVGGFSYVLLVRQKGKVYAAKILRYADDYGVPLASDENILRLFGYEMNRYLEI
119872609       YKVVRLLGVGGFSYVFAVRHKKSIYAVKILRYVDDYGNPLASDENILRIFGQEMQRYLEI
126458653       YKVVKLLGVGGFSYVLAVKRGGKIYAAKILRYTDDYGVPLAGDEKVLAFFGQEMNRYLEI
                ***::**********: *:   .:**.****:.** * ***.**::* :** **:****:

145590326       KSDHVVRAYEVYLPAVGYKDIGQYMRNPPYILLEYMDGGTLRDLLRAKKRLPAGYVAELF
18312200        KSDYIVKAFEVYLPAVGYRDIKQYMKNPPYILLEYMEGGTLRDLLRARKRLPVQQVIELF
119872609       KSDYVVKAYEVYLPAVGYRDVVQYMKNPPYILLEYMEGGTLRDLLRTRKKLPVAQVVELF
126458653       KSERIVKAYEVYLPAVGYKDLRHYMRDPPYILLEYMRG-TLRDVLRIKKTLPVPQVVEMF
                **: :*:*:*********:*: :**::********* * ****:** :* **.  * *:*

145590326       RQLAQGLYDIHRRNIIHLDIKPENILFTKDRKVAKIGDMGIAKVAIGGRVQSSFMSPAYA
18312200        KQLAQGLYDIHKHNIVHLDIKPENIMFTRDRKIAKIGDMGIAKVVTGGYVHSSYMSPAYA
119872609       RQLAQGLSDVHRHNVVHLDIKPENIMFTKDRKTAKIGDMGIAKVVSGGYVRSSYMSPAYA
126458653       KQLAAGLYDIHRHNVVHLDIKPENIMFG-EGGVVKIGDMGIAKVVAGGYVHSSYMSPAYA
                :*** ** *:*::*::*********:*  :   .**********. ** *:**:******

145590326       APEVKNGEASFSSDIYSLGCVIYEALTGINPNVFVENGYAIPPPSAYAADIPPWMDEIIL
18312200        APEVKKGLATFSSDIYSLGCVIYEALTGINPNVFVENGYQIPPPSAYVADVPRWIDEIIL
119872609       APEVKKGLASFASDIYSLGCVIYEMLTGINPNVFVENGYQIPPPSTYNPEVPTWLDQLVL
126458653       APELKRGVASFASDVYSLACVIYEALTGINPNVFVENGYPVPPPSSYNKEVPPWLDELLL
                ***:*.* *:*:**:***.***** ************** :****:*  ::* *:*:::*

145590326       KMLDLDPAKRPTVADLVSFI
18312200        KMLDLNPNARPNSAELVNYL
119872609       KMLEVEPTKRPSAEEIVTFL
126458653       KMLAPDPARRPSAAEVLKAL
                ***  :*  **.  :::. :
```

## Cluster No. 29 multiple sequence alignment

```
CLUSTAL 2.0.10 multiple sequence alignment


108758818       YLVLERLASGGMGVVYSAYDPELDRRVALKLLRVAALGLEAEQGRAHLLHEAQAMARVSH
108763383       YLLQGVLGMGGMGVVYAADDLRLGRRVALKLLRPLLAGAQD-QGRERLLREAQAMARLSH
86159218        FEIVRELARGGFGVVYAARDRTLGRSVAFKLVPSAASGAAD----DRVLREAEAAARLTH
119960545       YTVRSRLASGGMSTVYLATDQRLERDVALKVLHPHLANDPT--FLERLSREAKAAASLSH
                : :   *. **:..** * *  * * **:*::     .        :: :**:* * ::*

108758818       PHVVPVYDVGTFGPQVFLTMELVDARTLRPWLKD-APRTWRQVLALFLDAGRGLAAAHAA
108763383       PNVLPLFELGTAEGHDFLAMEWVDGTTLADWLRE-RERPWRDVLDVFLAAGAGLAAAHRA
86159218        PNIVTLFDVGRCAAGPYLVLELLRGETLAARLRR-GPLPLREALRVAEAVARGLAHAHEA
119960545       PHIVGVLDQGEDGTIAYLVMEYVKGHTLRDTLNEQGALQPRLALALIDPVIEGLAAAHRS
                *::: : : *      :*.:* : . **   *.       * .* :   .  *** ** :

108758818       GVVHGDFKPENLLVGQDGRVRVTDFGLARN-----ANPLDDVSP------------LAGG
108763383       GVVHRDFKPSNVLVGRDGRVRVTDFGLARRGTGESPEAPAGVSEGASKASMLTEWGQAAG
86159218        GVFHRDLSPGNVFLCEDGQVKVLDFGLAHA---------FGRPR------------TAGG
119960545       GLIHRDVKPENVLIADDGRIKVGDFGLARAIS-------ANTSTG-----------ALIG
                *:.* *..* *:::  **:::* *****:           . .                *

108758818       TPAYMAPEQMEAHAPADARSDQFAFCVTLFEALYGERPFAATTPHELLTE-VRAGKVRPA
108763383       TPAYMSPEQRAGRA-VDARGDQYSFCVALHEALHGERPGHLAAS--------------AA
86159218        TPQYMAPEQRRGAP-EDERTDVFALGAILFEMLTGEQPFPSAAPG-------RTPRRAPA
119960545       TVAYLAPELVLGQP-ADARSDIYSAGIMLYEMLTGKQPYAGDSPIQVAYQHVNAAVGRPS
                *  *::**   . .  * * * ::    *.* * *::*    :.              .:

108758818       PRGTHVPPWLRRVLLRGLSASAMDRYTDLNALLVALQH-
108763383       PRTSSVPRHLRTALARGLANAPEDRFPSMEALMAAL---
86159218        LQVPDLP-ALADLLGRMLAADPVRRPRDGGAVVAALGAL
119960545       DAAPGLAEDLDELVQWCTAVDAENRPVDGTALLTEL---
                   . :.  *   :    :  .  *  .  *::. *
```

Cluster No. 30 multiple sequence alignment

```
CLUSTAL 2.0.10 multiple sequence alignment


108759654       YRVLGELGRGGMALVYRGLHEMLQREVAIKELLP-DGQRDR---ETLSRFRREALALAAF
86156646        SRIVREIGRGGMGVVYEAFQEGLERPVAVKALDQ-KLVRSS---EVVERFRREGRAYAQL
116619364       YQVVGILGAGGMGQVYKVRNVISDRVEAMKVLLP-DLVNQP---DLADRFLREIKVQASL
108803345       YVVRERLGSGGMAVVYRAEDTLLGRDVAIKTLHR-RYAEMP---AFRRRFRQEARAMASL
120404511       YRVDSLIATGGMSAVYRGLDQRLDRPVALKIMDS-RYAGDQ---DFLTRFQREARAVARL
116619720       YEILSALGAGGMGEVYRARDARLGRDVALKVLPA-EVASDP---TRRQRFEIEARAIAAL
116622428       YEIRAPLGAGGMGEVYRARDTRLGRDVALKILPP-ELAEDP---SRRARFEQEARAVAAL
116620277       YEIIAPLGAGGMGEVYRARDLRLDREVALKLLPP-HGD--------RSRFQAEAKAVAAL
116623521       -----------MGEVYRATDTKLSRDVAIKVIPE-NFAADA---DRMARFAREAQVLASL
116624929       YEILGRLGAGGMGAVYRARDSRLHREVAIKVAAA-KFSD---------RSAREARTVAAL
116620426       YRLAEKLGEGGLGAVYRARDIRLGRDVALKFLQP-ALAQDE---GRRERFLNEARAAAAL
94968763        -----------MGVVYRAEDTRLGRQVALKCLPA-EYAADP---QMVERFLREARSASAL
71906078        FKITGTLGQGAMGTVYRAHDPLIERTVAIKTVAC-AGLSKDEALAFEQRFYREAKSAGRL
32471474        YQIESVIGRGGMGSVYRAKHAKSGEEVAVKLIAQ-HVADDMR---FRRRFDAEVETLRRL
21220218        YRLLSPLGEGGMGTVWRARDEVLRREVAVKEVRAPAGLSQPDVGRMYARLEREAWAAARI
                           :. *:.  .    .  *:*                 *    *      :

108759654       RHQNIVTLYDMVEKGESLFMVMELVDGPTLHTLIKEG-PLPADVTGVIAARIAS---ALD
86156646        RHEAIVAVHDLVEKDDQLYLVTDLVDGADLARVLAQGGALPADCVAVIGARLAE---ALD
116619364       EHPNIAALHTAVRVDNQLLMLMEFVEGVTLDQKLKNGPLPPANAVDHIMQVLA----ALE
108803345       DHQNIVKVYDISPDGEAPFIVEEYVGGRDLGSLLSRQRGGRLEEPFAVRVAVQ-LLKALA
120404511       KDPGLVAVYDQGIDGQHPFLVMELVEGGTLRELL-RERGP--MPPHAVAAVLRPVLSGLA
116619720       NHPNIVAVYDVGSESGVLYIVTELVEGETLVGAKA-----GLRKTIEIAVQIA---GGLA
116622428       NHPNIVAVFDVGDG----YMVSELVEGEPLRASHP-----GLRKTLDLAGQIA---SGLA
116620277       SHPNIVAIFDVGEN----YLVTELVDGHPFPVPTT-----PLRRLLDLAVQIA---DGLA
116623521       NHPNIAAIYGVEER----ALVMELVEGQTLAELIAQ-GAMPLAEAVPIARQIA---EALE
116624929       NHPNICHIYDVGPN----YLVLELVEGTTLAERLKQ-GPLPIEEALAIARQIG---DALE
116620426       NHPGIATIHSIEEDDGSLFIVMELIEGQTLRQLSGG-RPMPLARALQYCTAAA---DALR
94968763        NHPNICTIHEVDVANGQHFLTMELLEGQSLRDRIAS-GPIATDESIRIAVAVA---DALD
71906078        SHPNIVTIHDVGRNESLAFIAMEYLNGQSLRELLDSGVVLPPERCTEIAADVA---DGLA
32471474        RHPGIVRLIGYGEEAGQLFYSMELVRGETLQKRIRDVKRLGWLPTLDIASQVCS---ALK
21220218        SHPNVVTVYDVATDGGRPWIVMELVRGLSLADLLDAEGPLEPRRAALIGAEVL---AALR
                  .  :  :               : : *  :                          .*

108759654       HAHFRHIIHRDLKPANVMLTKSGEVKLMDFGIAKDVGMEALT----------------QQ
86156646        YVHWSGLLHRDVKPANVMISRLGEVKLMDFGIAKGAEDPSLT----------------RV
116619364       YAHARGVVHRDIKPANMMLTPSGAMKLMDFGIARSSADHKLT----------------QT
108803345       YAHWRGVIHRDIKPSNILITPGGIVKVADFGIARLVEEDGE-----------------GD
120404511       AAHSAGLVHRDIKPENVLISDDGEVKIADFGLVRAVAEAKIT----------------ST
116619720       AAHEAGIVHRDLKPSNIVLARDGRVKILDFGVAKVTPAIAA-----GAAITETITVNTRP
116622428       AAHAAGIVHRDLKPENVLVTKDGRAKILDFGLARIRRNAAV-----ASGQTETATVKTEP
116620277       AAHAAGFIHRDLKPANILLSRDGRAKILDFGLAKRVGGLQS-----DSNQTQTA---TQA
116623521       YAHEKGIIHRDLKPANVKVTPEGRVKVLDFGLAKALASEGPVSGDPQSSPTMTMR-ATMA
116624929       AAHERGIVHRDLKPGNIIVRPDGTVKVLDFGLAK-IVEETPPEGDPEHSPTVTLEQLTRA
116620426       AAHHKGIIHRDIKSSNIMVTAEDRVKVLDFGLAKLPESSLV-------TQENI-------
94968763        AAHEKGIVHRDIKPANIFLTERGEAKVLDFGLAKLESSNLA-------MSATVDANLTSP
71906078        FAHANGVVHRDIKPANIMVLDNGSVKISDFGVALIPSGSTT-----------------VE
32471474        HAHDIGVIHRDLKPANLILTDAGEVKLVDFGIAKLFGFGEQT----------------LH
21220218        AAHAAGVLHRDVKPANVLLANDGRVVLTDFGIARVEGSEALT----------------MT
                  .*    .:***:*. *: :    .    : ***:.

108759654       GMAVGTPSYMSPEQVTGVPV-DGRTDIFSLGVLLYEALSGARPFHGKTAGEVFAKIRDGK
86156646        GMLVGSPSYMAPEVLAGDEG-GPAADVWALGVTLYELVCGEKPFRGANADELFRLVRRGR
116619364       GTTVGSLYYMSPEQIQGVVAPDARSDIYSVGVSLYELVTGKRPFDGDSQFAIMSAHLAGT
108803345       GEIIGSARYMSPEQLRGLEA-TQRSDVYSVGVLLYHCLTGRPPFSGDVRSLVR-QHLREA
```

```
120404511       SVILGTAAYLSPEQVATGET-DSRGDVYSVGILVYELLTGRTPFTGDTALAVAYQRMDRD
116619720       GAVMGTVGYMSPEQVLGNSA-DHRSDIFSFGIILNELLTGKRAFQGATSVDTMQAILRQD
116622428       GTVMGTVGYMSPEQVRGLEA-DHRSDIFSFGVMLHEMLAGTRPFLGETSVETMMAILKQD
116620277       GVVVGTVAYMSPEQARGVAL-DIRSDQFSFGLVLYEMITGKRPFVRQTAPETMTAILREQ
116623521       GMIMGTAGYMPPEQAKGKPV-DRRADIWAFGVVLAEMLSGRQLYCGETISETLAAVLLKD
116624929       GSVFGTAAYMAPEQARGNPV-DKRADIWAFGVVLYEMLVGKRPFQGETVSDTLAAVLTRE
116620426       --LMGTMAYMSPEQAIGAAV-DPRCDLWSLGIVLYELLTGRLPFTGENYRAVLHGLLNTE
94968763        GQAIGTIAYMSPEQARGIDV-DARSDLFSLGVLIYEMATGVPPFPGATSALIFDAILNRQ
71906078        GTAFGSPKYMSPEQVTGQKV-DGRSDIFSLGAVLYEMLAGRPAFDGEELTAVLYQVLHAA
32471474        GSVLGTADYMAPEQAGSHSI-TPRTDLYALGSVMYAMLAGRAPFAGKKVTQVVEALQRDR
21220218        GEVVGSPEFLAPERALGRTP-GAASDLWSLGVLLYATVEGVSPFRQGTPLSTLRAIVDEA
                   .*:  ::.**            * ::.*  :     *   :

108759654       YTPLSKVAPNVPAPLVRIIQRAMEVKPEDRFPDAAAMRRELDVF----
86156646        FRRVRSLAPGCPARVAAAIERCLARAPERRWKSAGALARHL-------
116619364       PVPPVKVDPRLPQSLNDVILMSVAKDASARFQTAGAVRNAL-------
108803345       PRPPRELNRGISPQMEAVILRAMAKDPEARYPSATAMLDEI-------
120404511       VAPPSTVIDGVPRQFDDLVLCATSRDPADRFADAAEMAEEL-------
116619720       P---PLLPETVPVPVRQIVQHCLEKEPGNRFQSARDLAFA--------
116622428       A---PDLPGSVPETVRTIVEHCLEKEPANRFQSAKDLGFA--------
116620277       A---EPLPADVPPPLRWIVERCLAKEPADRYDTTRGLYLELRNLRDHM
116623521       PD-LSGLPAGTPGTIRRLLQRCLDKDPRSRLRDIGEARVI--------
116624929       PE-WTGIPP----KVERLLRRCMERDPKRRLRDIGDAQFL--------
116620426       PEPVSRYRGDVPATVHRILARALTKDRELRYASADLMLSDLR------
94968763        ATRASKVNAATPAGLENIIAKLLEKDPRLRYQSAADLLADL-------
71906078        PPLPSSFNPELPRVFDRLIAKSMAKDPDKRYQNATDLAADL-------
32471474        PVPLDLINPDIPAEVVEIVHQLLEKDPADRPPTALAVMNRL-------
21220218        VP-----PPRRAGALGPVVEGLLRKDPAERLPAEEAERAL--------
                              .   :          *
```

Cluster No. 31 multiple sequence alignment

```
CLUSTAL 2.0.10 multiple sequence alignment


108756948       FEFVRMLERRANGEELLLADRYQRHGLAGPVVIKRVRSPASSARRHRLVEEVQLAYRLHH
108761165       YAALRTLERRGNGEVVLLAERHLPHGLAGHVTIKRLRNPASFERCQRLIEEVQLAFRLHH
108757305       YELIRYLGARGSGE-LLLARRHYAGVPGDLVLVKRLQDVGDAQGRARLREEVKLLMRLSH
108761534       FEFVRKLEMRSTGELLMLAQRRYRNGLSGPVVVKRLRNPATFVERRRLVEEVDLTFRLNH
108757303       ---------------LKAERRVADGVLAGPCLVRRIATPSTYMMRTRLIEEVQLAFRLNH
                               :    *      ..   ::*:   .      ** ***.*  ** *

108756948       PTIAQVHYFKIHRGK--PYIIMEHVDGPSLETVLDLMAMRGQPVSLAFALHVAAELADAL
108761165       PAIAQVHHLKIHADR--PHIIAEYVDGPTLDTVISLATMRERPLCAPFALYIAAEIADAL
108757305       PAIVQVFLVRVHEGL--PHLVMEHVDGKSLETLISYAALRRRPFSEAFAAYVGVEVADAL
108761534       PAIAKVMLLKLYRGA--PHVVMEYVEGRSLDTVLNLAAMRRRPLSAEFAAHVTAEVADAL
108757303       PNIAQVFHMQVDEEADAPFAVMEYVGGPSLETLVTAALVRGKPLSEGFALYVGAEVADAL
                * *.:*  .::      *. : *:* * :*:*::    :* :*..  ** :: .*:****

108756948       HHAHSLKDAQGRSLGLIHRDVSPRNVRVARTGEVKLTHFGVAYSHLVGREETAEALLKGD
108761165       HHSHTLRDSENRPLGIIHRDVAPRNIRVARSGEVKVTDFGAAYSLMVGREETPGLLLKGD
108757305       HHAHTLEDARGRPLGIVHRDVSPRSLRLDARGHVKLSDFAMAWAKLPGRIVTETHVVRGD
108761534       SHAHGLTDEWSRPLNIVHRDVSPRNIRVGVHGEVKLTHFTVAASSLSGREVTSQELVKGD
108757303       HYAHTLTSEEGVPLGIVHRDVSPRRLALGPHGEVKVLDFGAAYSLMVGREESPENLLRGD
                 ::* * .  . .*.::****:** : :   *.**: .*  * : : **  :   :::**

108756948       VAYASPEYLAGKTLTAASDLFSLGLVLLELATGRHLFAAAAEALEPPRAKSHE----LRL
108761165       VAYASPEYLLRKPMDGRSDIFSLGLVLMEMLTCKHLFD-LEDAQAP--IAALD----VKT
108757305       LAYASPEALERKPLDGRADLFSLGVVLLEVLTGLHLLDLEDVERAALAAGPLPDAEVLLA
108761534       IAYASPEALRRRKVDARSDLFSLGLVLLELLTGRHPLMVDDLA-PVLESEPL----ELHA
108757303       VAYASPEYLRKEGLTPRSDVFSLGVLLVEVLTSKHLFEVHDVPVVASKETRFV------T
                :****** *  . :   :*:****::*:*: *  * :

108756948       EEPPSLPLTQMLMLLEDHGPQDVERAAASLPPKVRAVLQGMLHKDATKRFGSAEALCEAL
108761165       EEVPSVPLTQMIALVSRYRSEDVEHAMAGLPDALKAIIHKALQRKPSERYTSAAELRDAL
108757305       EVPSWLPAPLMAARMACLAPAHVERATQGLSPAMRAILGRLLRREPSERFQSGQELADAL
108761534       QGPTWMPVKEVAARMAQVGPEQVERLAAGVAEPLRCILLRALRQNPSERFQTGAEMAEAL
108757303       ESPPTLPLQQMQALMETFTPDVVEKAVAHLSEDLKAVLHTALRLNPEERFATAADMRDAL
                :  . :*   :   :    .  **:    :.  ::.::   *: .. :*: :.  : :**

108756948       ----
108761165       ----
108757305       RAV-
108761534       RAWL
108757303       ----
```

Cluster No. 32 multiple sequence alignment

```
CLUSTAL 2.0.10 multiple sequence alignment


126179714       YDRIAFVGRGGLGQVFSAVRRDDRRTVAVKIPAAYDEATGKTFMKEMRFWEDLAHPNIVT
154151509       YSDIRHIGRGGVAQVFAAHRKSDNLLVAVKIPISFDEVTGKCFLNEIAAWQTLRHKNIVE
154150293       YPGAEYLAEGGVSRVFKARDEKNGRDIAVKVPIRFDEVTGTQFTKELSIWEGLHHENIVE
18977027        YRPLEVLGEGGFAEVYKVVRKNDGKIVAIKVP-RINQGTSKLFIKEVSIWLHLNHPNIVR
                *     :..**...*: .  ..:   :*:*:*   :: *.. * :*:  *  * * ***

126179714       VHSVNILPVPFVEMEYLP---------RTLDDLEKPLPVETAARIAAGIAAGLAYAHEKG
154151509       VLEVNILPVPYVEMEYVP---------GSLESVEKPLPVWKAVHLVHGIADGLAYAHTHG
154150293       LYAANIFPLPFIEMEYVH---------RSLAEVPFPIDETRAVGILMGVAEGLRYAHGQG
18977027        LYDADILPIPHLEMEYVEGVKINGKIVRNLDEYPKPMREDIALKIIKGIAEGLKHAHSRG
                :  .:*:*:*.:****:           .* .   *:    *  :  *:* ** :** :*

126179714       VIHRDIKPRNILLDDGLTPKITDWGMSTTVAASGDTG-VAGFTLAYAAPEQIAPERFGRA
154151509       IIHRDIKPHNILVTSDFVPKITDWGMSKVIATTMDKSNVAGFSLSYAAPEQVSPAEFGRT
154150293       IVHRDIKPGNILLTPDGIPKITDWGLSKAQGTK--QSGIIGFSLEYAAPEQLAPNLFGEP
18977027        IYHLDLKPLNILLTRDLTPKITDWGLAKIAVRTFSGS-ISGYTPLYAAPEQLAPSKYGGV
                : * *:** ***:  .  *******::.    .   . : *::  ******::*  :*

126179714       DARTDIYQLGAVFYELVTGKAPFARESLAGLAEAVIRE--QPAPPSEIDPTLAALDPIIL
154151509       DIRTDIYQLGALFYELVTGSIPFGGDSIVEVGNAIVRD--DPLPPTEYNPDAEVVEKIIL
154150293       GPWTDIYQLGVLFYEMLAGHVPFSGAGVGEITHAILHD--APAPVVTGDKNEEIINTIIA
18977027        DERTDVWQIGVIFYELLTGKLPFDGYTYEEIRGKIVDEGYNFKPPSTFSPKLAKYNKIFE
                .  **::*:*.:***:::*  **       :   :: :     *    .      : *:

126179714       RCLAKDPADRYQSAGEVLEAI
154151509       KCLAKNPADRYQSADELLNAL
154150293       RCLRKRPEDRYASVADLITDL
18977027        KLLAKKKENRYQTIPEFLTDL
                : * *   :** :  :.:  :
```

Cluster No. 33 multiple sequence alignment

```
CLUSTAL 2.0.10 multiple sequence alignment


2633949          YQILRVIGGGGMANVYLAEDIILD-REVAIKILRFDYANDNEFIRRFRREAQSASSLDHP
52785557         YEILRAIGGGGMANVYLALDIILD-REVAIKVLRFDFVHDADFIRRFRREAQSAASLDHP
118479084        YKLLKMIGGGGMANVYLAHDDILG-RDVAVKILRLDYSNNEEFIKRFHREAQSVTTLSHP
30258509         YKLLKMIGGGGMANVYLAHDDILG-RDVAVKILRLDYSNNEEFIKRFHREAQSVTTLSHP
29897506         YKLLKMIGGGGMANVYLAHDDILG-RDVAVKILRLDYSNNEEFIKRFHREAQSVTTLSHP
30021950         YKLLKMIGGGGMANVYLAHDDILG-RDVAVKILRLDYSNNEEFIKRFHREAQSVTTLSHP
138894697        YKIISLIGGGGMANVYLARDIILE-RDVAVKVLRLDFANDEQFIKRFRREAQAATSLNHE
16411274         YKILHAIGGGGMANVYLAHDIILD-RDVAVKILRIDLADESNLIRRFQREAQSATSLVHP
16414435         YKILHAIGGGGMANVYLAHDIILD-RDVAVKILRIDLADESNLIRRFQREAQSATSLVHP
14973227         YRIVKQIGRGGMADVYLAKDLILDGEEVAVKVLRTNYQTDPIAVARFQREARAMADLDHP
15459243         YRIVKQIGRGGMADVYLAKDLILDGEEVAVKVLRTNYQTDPIAVARFQREARAMADLDHP
125718643        YKIIKQIGRGGMADVYLAKDLILDGEEVAVKVLRTNYQTDPIAVARFQREAKAMADLDHP
24376858         YRILQSIGRGGMADVYLANDLILDNEEVAIKVLRTNYQTDQVAVTRFQREARAMAELNHP
55821411         YRILKSIGRGGMADVYLANDLILDNESVAIKVLRTNYQTDQVAVARFQREARAMTELSHP
76788494         YRILKSIGRGGMADVYLARDLILDNEEVAIKVLRTNYQTDQIAVARFQREARAMAELTHP
12724921         YRIIKEIGRGGMANVYQGEDTFLGDRLVAIKVLRSNFENDDIAIARFQREAFAMAELSHP
29345043         YHIIGSIGSGGMANVYLAHDLILD-RDVAVKVLRFDFQNDQAAIRRFQREALAATELVHP
29377576         YHIIGSIGSGGMANVYLAHDLILD-RDVAVKVLRFDFQNDQAAIRRFQREALAATELVHP
28378319         YRIVRSLGEGGMANVYLAHDLILD-RDVAVKLLRLDLRDDPKTIKRFQREALATTELVHP
104774276        YRIVDTLGEGGMANVYLADDIILK-RQVAVKIIRLDLQKDSQVLARFQREALATSELSHP
116490836        YRIIRPLGEGGMANVYLAVDTKNN-QQVAIKVLRLDLQNNPDFVRRFQREAQAAAQLLHP
10175124         YEILETIGGGGMADVYKAMDVILD-RQVAVKVLQAQFSKDEQFIKRFRREAQAATSLAHQ
13701020         YKIVDKLGGGGMSTVYLAEDTILN-IKVAIKAIFIPPREKEETLKRFEREVHNSSQLSHQ
21282832         YKIVDKLGGGGMSTVYLAEDTILN-IKVAIKAIFIPPREKEETLKRFEREVHNSSQLSHQ
27467813         YTITDKLGGGGMSIVYLAVDSILN-RKVAIKAISIPPSEKEETFKRFEREVHNSSQLSHE
134299570        YEILEQLGGGGMALVWKGKDTFLN-RLVTIKVLRPEYASDQEFVRRFRREAQAVASLSHP
83589763         YEIVSELGGGGMARVYRGQDRLLN-RNVTIKILREQYASDKEFLARFQREAQAVASLSHP
108802877        YRIVRRLGSGGMADVYLAHDDILD-RDVALKVLSSHYAADAEFVERFKREAQSAAALAHP
108803785        YAVLGRLGGGGMAEVFLARDRVLG-REVALKVLREAYAADAEFVERFRREARSAASLSHP
118444123        YELLQKIGEGGMAEVYKAKCHLLN-RFVAVKVLKSQYSDDIEFVNKFKQEASSAASLSHN
28210913         YELLEKVGEGGMAEVYKAKCHFLN-RYVAVKILKEEFSQDAQFVEKFKREATAAASISDN
18145401         YELLQCVGEGGMSFVYKARCRKLN-RFVAVKILKDEFKNNEEIVRRFKKEATAIANLSNP
15024695         YKIEEEIGVGGTAVVYKAMDTLLN-RHVAVKVLKHEFTEDEEFVFKFKREASAAARIANA
125973093        YELIEKIGGGGMADVYKARCKLLN-RFVAIKILKPEFINDEEFLKRFTIEAQAAASLSHP
20807943         YEILEKIGEGGMAKVYKAKCHLLN-RIVAIKILRPEFAADENFVKKFKRESQAAASLSHP
89895431         YEIIEKIGSGGMAIVYKAKDLLLN-RIVAIKILHEQFTADEEFVRRFRREAQSAASLSHA
116626609        YRIISKIGQGGMGTVYRAMDTKLG-REVAIKILPDQFAADSSRMARFSREAKVLAALNHP
116626708        YRIAAKIGEGGMGAVYRAGDTKLN-RDVAIKVLPESFAQDPDRLARFTREAQLLAGLNHP
                 * :   :* ** . *: .         *::* :      .   . :*  *    : : .

2633949          NIVSIYDLG-EEDDIYYIVMEYVEGMTLKEYITANG-PLHPKEALNIMEQIVSAIAHAHQ
52785557         NIVSIYDVG-EEDDIYYIVMEYVEGMTLKEYINRTG-PLHPKEAVQIMEQIVSAIAHAHD
118479084        NIVNMYDVG-EEDGIYYLVMEYVPGQTLKQYIIERG-MLPIGEALDIMEQLTSAMAHAHH
30258509         NIVNMYDVG-EEDGIYYLVMEYVPGQTLKQYIIERG-MLPIGEALDIMEQLTSAMAHAHH
29897506         NIVNMYDVG-EEDGIYYLVMEYVPGQTLKQYIIERG-MLPIGEALDIMEQLTSAMAHAHH
30021950         NIVNMYDVG-EEDGIYYLVMEYVPGQTLKQYIIERG-MLPIGEALDIMEQLTSAMAHAHH
138894697        HIVSIYDIG-EEEGVYYIVMEYVRGATLKQYIQQYA-PLPVEQALRIMDQLTSAIAHAHE
16411274         NIVSVYDVG-EENDLHYIVMEHVDGMDLKQYIHENH-PISYEKAVDIMLQIVSAVAIAHQ
16414435         NIVSVYDVG-EENDLHYIVMEHVDGMDLKQYIQENH-PISYDKAVDIMLQIVSAVAIAHQ
14973227         HIVRITDIG-EEDGQQYLAMEYVAGLDLKRYIKEHY-PLSNEEAVRIMGQILLAMRLAHT
15459243         HIVRITDIG-EEDGQQYLAMEYVAGLDLKRYIKEHY-PLSNEEAVRIMGQILLAMRLAHT
125718643        HIVRITDIG-EEDGQQYLAMEYVAGLDLKRYIKENS-PISNEEAVRIMGQILLAMRLAHT
24376858         NIVAIRDIG-EEDGQQFLAMEYVDGADLKKYIQDHA-PLSNAEVIRIMKEVLSAMTLAHQ
55821411         NIVAIRDIG-EEDGQQFLVMEYVDGSDLKKFIQDHA-PLSNQDVVRIMGEVLSAMTLAHQ
76788494         NIVAIRDIG-EEDGQQFLVMEYVDGFDLKKYIQDNA-PLSNNEVVRIMNEVLSAMSLAHQ
```

```
12724921        NIVGISDVG-EFESQQYIVMEFVDGMTLKQYINQNA-PLANDEAIEIITEILSAMDMAHS
29345043        NIVSVYDVG-EEDGLQYLVMEYVKGMDLKRYIQTHF-PIPYSTVVDITQQILSAVAMAHE
29377576        NIVSVYDVG-EEDGLQYLVMEYVKGMDLKRYIQTHF-PIPYSTVVDITQQILSAVAMAHE
28378319        HIVSLYDIG-EENGMQYLVMEYVKGMDLKNYIKENF-PLPLQQVIDIMEQILSAVATAHA
104774276       NIVSVFDVG-TDHGLPYMVMEYVKGPDLKEYIREKS-PIPLPQVIKIMDQILSAMELAHK
116490836       NIVKVLDAG-SFDGVQYLTMEYVDGMDLKKYISQYY-PVPYAQVVNIMEQVLSAVSMAHN
10175124        NVVSIYDVG-EEENLYYIVMEYVEGPTLKELIQQRG-PLPVDETIDIMSQMMAAISHAHM
13701020        NIVSMIDVD-EEDDCYYLVMEYIEGPTLSEYIESHG-PLSVDTAINFTNQILDGIKHAHD
21282832        NIVSMIDVD-EEDDCYYLVMEYIEGPTLSEYIESHG-PLSVDTAINFTNQILDGIKHAHD
27467813        NIVSMIDVD-EEDDCFYIVMEYIEGPTLAEYIHSHG-PLSVETAIQFTEQILSGIKHAHD
134299570       NIVSIYDVG-QENESHYLVMEYVDGESLKEMIRREA-PLAPFRVIQLGRQIADALEHAHE
83589763        NVVSIYDVG-QEDDLHYLIMEYVEGRSLKDLISERA-PLPPLEAIDISLQICDALEHAHE
108802877       NIVSIYDRGEAEDGTYYIAMEYLPGGTLKDRILRKG-ALPPKTAAAVALQIAEALKAAHQ
108803785       NIVAIYDHGRAEDGTYYIAMEHVPGGTLKERILGGA-PLPPEEAAGIASQVARALEAAHR
118444123       NIVGIYDIG-SENNINYIVMEYIDGKTLKQIINENG-SLGFNASIDIAIQIAKALECAHN
28210913        NIVNIYDVG-SEENINYIVMEYVQGETLKDVIRKKG-KIPYKEAVKLAIQIAKGLECAHR
18145401        NVVNVLDVG-TQDDINYIVMEYVEGKTLKDIIKEKG-ALPYEVAISIGIKVAKALECAHK
15024695        NIVNIYDVG-ADGNVNYIVMEYVAGKTLKKLIKENG-KIEFNKIIDYATQIAKALNFAHK
125973093       NIVSIYDVG-QENDIHYIVMEYVNGQTLKEYLDENG-ALYWKDAVNIAIQICQAIEHAHK
20807943        NIVGIYDVG-QEGDIYYIVMEYVKGRTLKELIRENGGPLEVKRAVEIASQVCRALDHAHK
89895431        NIVSIYDVG-KDGETEYIVMEHIEGQNLKDIIRNYA-PLSTEQTLELGIQIAEAIRHAHE
116626609       NIAAIYGVE-----ECALVMELVAGKPLA-------GPLPVDTALDYARQIADALDYAHD
116626708       NIAAIYGVE-----ERALVLELVEGPTLADRI--AQGPIPLDEALPIARQLAEALEYAHE
                ::. : .          : :* : *  *          :          ::  .:  **

2633949         NQIVHRDIKPHNILIDHMGNIKVTDFGIATALSSTTITHTNS--------------VLGS
52785557        NQIVHRDIKPHNILIDHMGHIKVTDFGIAMALSSTTITHTNS--------------VLGS
118479084       FEIVHRDIKPHNILIRADGVIKVTDFGIATATSATTITHTNS--------------VLGS
30258509        FEIVHRDIKPHNILIRADGVIKVTDFGIATATSATTITHTNS--------------VLGS
29897506        FEIVHRDIKPHNILIRADGIIKVTDFGIATATSATTITHTNS--------------VLGS
30021950        FEIVHRDIKPHNILIRADGIIKVTDFGIATATSATTITHTNS--------------VLGS
138894697       NGIIHRDIKPQNILLDEDGNVKVTDFGIAVAMSGTTITQTNS--------------VLGS
16411274        HHIIHRDLKPQNILIDHDGVVKITDFGIAMALSETSITQTNS--------------LLGS
16414435        HHIIHRDLKPQNILIDHDGVVKITDFGIAMALSETSITQTNS--------------LLGS
14973227        RGIVHRDLKPQNILLTPDGTAKVTDFGIAVAFAETSLTQTNS--------------MLGS
15459243        RGIVHRDLKPQNILLTPDGTAKVTDFGIAVAFAETSLTQTNS--------------MLGS
125718643       RGIVHRDLKPQNVLLTPDGNAKVTDFGIAVAFAETSLTQTNS--------------MLGS
24376858        KGIIHRDLKPQNVLLTKDGTAKVTDFGIAVAFAETSLTQTNS--------------MLGS
55821411        KGIIHRDLKPQNVLLTKDGRAKVTDFGIAVAFAETSLTQTNS--------------MLGS
76788494        KGIVHRDLKPQNILLTKKGTVKVTDFGIAVAFAETSLTQTNS--------------MLGS
12724921        HGIIHRDLKPQNVLVSSSGTVKVTDFGIAKALSETSLTQTNT--------------MFGS
29345043        HRIIHRDLKPQNILIDEHGTVKITDFGIAIALSETSITQTNT--------------MLGS
29377576        HRIIHRDLKPQNILIDEHGTVKITDFGIAIALSETSITQTNT--------------MLGS
28378319        HNIIHRDLKPQNILIDEQGNAKITDFGIAVALSEHTMTQTNT--------------ILGS
104774276       HNVIHRDLKPQNILMDEKGNIKIADFGIAVALNQSAITQTNS--------------VLGS
116490836       HGIVHRDLKPQNILVGKDGSIKIVDFGIAIARSEFGMTQTNA--------------VLGS
10175124        NQIVHRDIKPHNILIGEDGVVKVTDFGIARAMSSATITHTNS--------------VMGS
13701020        MRIVHRDIKPQNILIDSNKTLKIFDFGIAKALSETSLTQTNH--------------VLGT
21282832        MRIVHRDIKPQNILIDSNKTLKIFDFGIAKALSETSLTQTNH--------------VLGT
27467813        MRIVHRDIKPQNILIDKNKKLQIFDFGIAKALSETSLTQTNH--------------VLGT
134299570       NSIIHRDVKPHNILITRSGRAKLTDFGIAQAS-ASTITHTDT--------------IVGS
83589763        NGVIHRDIKPHNILITRNGRVKVTDFGIAQAVSEVTMSQSGT--------------MIGS
108802877       RGVVHRDIKPHNILITESGDLKVTDFGIARAASSSTMTRTGA--------------VLGT
108803785       RGIVHRDIKPQNILLGASGEAKVADFGIARAASEATISGTSL--------------VLGT
118444123       NNIIHRDVKPHNILVTRDRNIKVTDFGIAKATSSVTITNSDK--------------IIGS
28210913        NNIIHRDIKPHNILVTADNLVKVTDFGIAKATNSVTITNTSQ--------------IIGS
18145401        SGIIHRDVKPQNILVTEEGVVKVTDFGIAKSMDSSTIAHTNS--------------VMGS
15024695        NGIVHRDIKPHNIMVTDDDIIKVTDFGIAKASNESTITTTNK--------------VVGS
125973093       NHVVHRDIKPHNILLTKDGMLKVTDFGIARAVSSSTITMAGN--------------AIGS
20807943        NKIIHRDIKPQNILVTDEDVVKVTDFGIARAANGATITYTGD--------------VIGT
89895431        HHIIHRDIKPHNILVTEDGRIKVTDFGIARAVSAATMTHTGD--------------IVGS
116626609       KGIVHRDLKPANIMVTPEGVVKVLDFGLAKATA-EDVRTASESFPTVTLDETEPGVVMGT
116626708       KGVIHRDLKPANIKITHEGRVKVLDFGLAKALAPETVAGDPSSSPTMTMRATALGMIMGT
                  ::***:** *: :      :: ***:* :     :                    .*:
```

```
2633949         VHYLSPEQARGGLATKKSDIYALGIVLFELLTGRIPFDGESAVSIALKHLQAETPSAKR-
52785557        VHYLSPEQARGGLSTKKSDIYSLGIVLFELLTARMPFEGESAVSIALKHLQSETPSVKR-
118479084       VHYLSPEQARGGIANKQSDIYSLGIVMFELLTGRQPFSGESAVAIALKHLQSEIPSPKR-
30258509        VHYLSPEQARGGIANKQSDIYSLGIVMFELLTGRQPFSGESAVAIALKHLQSEIPSPKR-
29897506        VHYLSPEQARGGIANKQSDIYSLGIVMFELLTGRQPFSGESAVAIALKHLQSEIPSPKR-
30021950        VHYLSPEQARGGIANKQSDIYSLGIVMFELLTGRQPFSGESAVAIALKHLQSEIPSPKR-
138894697       VHYLSPEQARGGIATEKSDIYSLGVVMFELVTGRLPFSGESAVSIVLKHLQEETPSPKD-
16411274        VHYLSPEQARGGMATQKSDIYSLGIVLYELLTGKVPFDGESAVSIAIKHLQADIPSARE-
16414435        VHYLSPEQARGGMATQKSDIYSLGIVLYELLTGKVPFDGESAVSIAIKHLQAEIPSARA-
14973227        VHYLSPEQARGSKATVQSDIYAMGIIFYEMLTGHIPYDGDSAVTIALQHFQKPLPSVIA-
15459243        VHYLSPEQARGSKATVQSDIYAMGIIFYEMLTGHIPYDGDSAVTIALQHFQNPLPSVIA-
125718643       VHYLSPEQARGSKATVQSDIYAMGIIFYEMLTGHIPYDGDSAVTIALQHFQKPLPSIIS-
24376858        VHYLSPEQARGSKATVQSDIYAMGIMLFEMLTGHIPYDGDSAVTIALQHFQKPLPSIIA-
55821411        VHYLSPEQARGSKATIQSDIYAMGIMLFEMLTGHIPFDGDSAVTIALQHFQKPLPSILA-
76788494        VHYLSPEQARGSKATVQSDIYAMGIMLFEMLTGHIPYDGDSAVTIALQHFQKPLPSILA-
12724921        VHYLSPEQARGSNATVQSDIYAIGIILFELLTGQIPFDGDSAVAIALKHFQESIPSIIN-
29345043        VHYLSPEQARGSMATNQSDIYAVGIILYEMLTGNVPFDGESAVTIALKHFQEEIPSVKM-
29377576        VHYLSPEQARGSMATNQSDIYAVGIILYEMLTGNVPFDGESAVTIALKHFQEEIPSVKM-
28378319        VHYLSPEQARGSMATKQSDIYSLGIILYEMLTGSVPFKGETAVSIALKHFQNAMPSVRE-
104774276       VHYMSPEQTRGGMVTKQSDIYSLGIILYEALTGHVPFNGETPVAIALKHAEDDIPSLRK-
116490836       VHYLSPEQTRGGMATNKSDIYALGVILFEMLTGQVPYKGETVVSIAMKHSSEQMPSAKD-
10175124        VHYLSPEQARGGLVTFKSDIYSLGIVLFEMVTGQLPFSGDTAVSIALKHLQNDIPSPKE-
13701020        VQYFSPEQAKGEATDECTDIYSIGIVLYEMLVGEPPFNGETAVSIAIKHIQDSVPNVTTD
21282832        VQYFSPEQAKGEATDECTDIYSIGIVLYEMLVGEPPFNGETAVSIAIKHIQDSVPNVTTD
27467813        VQYLSPEQAKGEATDESTDIYSIGIVLYEMLVGEPPFNGETAVSIAIKHIQDSIPNITTD
134299570       VHYISPEQAKGEPAGPKSDIYALGVVLYEMLTGQVPYQADGAIGVALKHIQEEPLSLRE-
83589763        VHYLAPEQARGGVIGATADIYSLGIVLYEMLTGDLPFHGETPVAVALKHLQENPRPVRE-
108802877       AHYISPEQAMGEPVGPPSDLYSLGVVLYEMLTGQLPYDADTPIGIAMKHVNGHLRPPRE-
108803785       VGYMSPEQAMGRRAGPRSDLYSLGAVLYEMLVGNLPYEADTPVAVAVRHIHEPPRHPRA-
118444123       AHYISPEQAKGRFVDCKTDIYSLGIILYEMVTGKVPYDGESPVSVALKHVQEELIPPIK-
28210913        VHYFSPEQAKGGFIDFRTDIYSLGIVLYEMVTGQLPYNGETAVSIALKHIQGDFIPPKT-
18145401        AHYFSPEQAKGTYTDYRTDLYSLGIVLYEMVTGVVPFNGDSPVTVAVKHIQEKAIPPKN-
15024695        AHYLSPEQAQGIPVDCRTDIYSFGIVLYEMATGKVPYDADTPVSIALKHIQDAAVPPNE-
125973093       VHYFSPEQARGGFTDEKSDLYSLGIVLYELLTGRVPFDGESPVAVAIKHIQDEPEEPIN-
20807943        AYYFSPEQAKGSIVDERTDIYSLGIVLFEMLTGKVPFEGDSPISVALKHIQEDILPPSR-
89895431        VHYLSPEQARGIQTNEQSDLYSLGIILYELLTGKVPYDGETPISIALKHLQELAVPPSK-
116626609       ARYMAPEQARGKPVDKRADIWAFGVVLYEILTGKHLFQGDTASDTIVAVLTLEPN-----
116626708       AGYMSPEQARGKRVDRRADIWAFGVVFYEMVTGRALFDGDTVSDLIAQVLTKEPD-----
                . *::***: *      :*:::.* :::*  ..   : .:

2633949         WNPSVPQSVENIILKATAKDPFHRYETAEDMEADI-
52785557        WNPAVPQSIENVVLKAMAKDPFHRYEAAEEMENDL-
118479084       WNENIPQSVENIILKATAKDPFHRYQSANAMKRDI-
30258509        WNENIPQSVENIILKATAKDPFHRYQSANAMKRDI-
29897506        WNENIPQSVENIILKATAKDPFHRYQSANAMKRDI-
30021950        WNENIPQSVENIILKATAKDPFHRYQSANAMKRDI-
138894697       WNPEIPQSVENIILKAMAKDPFYRYPSAQEMNEDI-
16411274        QNPEIPQSLENIIIKATAKDPFLRYQNAEEMEKDL-
16414435        QNPEIPQSLENIIIKATAKDPFLRYQNAEEMEKDL-
14973227        ENPSVPQALENVIIKATAKKLTNRYRSVSEMYV---
15459243        ENSSVPQALENVIIKATAKKLTNRYRSVSEMYV---
125718643       ENPNVPQALENVVIKATAKKLTDRYQSVAEMYVDL-
24376858        ENKNVPQALENVVIKATAKRLTDRYHSTQEMLQDL-
55821411        ENRNVPQALENVVIRATAKKLENRYNSTLEMSRDL-
76788494        ENKSVPQALENIVIKATAKKLTDRYKTTYEMGRDL-
12724921        LNPEVPQALENVVIKATAKDIKNRYTDVEEMMTDV-
29345043        FDPGIPQSLENVVRHATAKDPSDRYKTANEMAEDL-
29377576        FDPGIPQSLENVVRHATAKDPSDRYKTANEMAEDL-
28378319        FDADIPQALENVVLQATTKDPRERYATVEDMAADLL
104774276       QNKAIPQALENVVLKATAKDPRDRYASVAEMKADL-
116490836       IDPNIPQALENVILRATAKNPDNRYLTAEDMANDL-
10175124        IQPSLPQSIENVIVKATAKDPFYRYGSVSEMDEDL-
13701020        VRKDIPQSLSNVILRATEKDKANRYKTIQEMKDDL-
```

```
21282832        VRKDIPQSLSNVILRATEKDKANRYKTIQEMKDDL-
27467813        KRDDVPQSLSNVVLRATEKDKYHRYHTVQEMCDDL-
134299570       INPNVPEDLEKVVLRAMDKRQDRRHKSAKALGEDLV
83589763        LNPNVPPALERVVMRTLEKDPARRYPSAAALRSDLL
108802877       VNPAVPEGLDAITVRLLAKDPKERYQSADELMEDL-
108803785       ANPAVPQALDAVTVRLLAKRPEDRYPDAATLAEDL-
118444123       VNPNIPESLNKLILKAVEKEPYKRYQSAHDMI----
28210913        INPSLSESLNSIILKCMEKEPVKRYQNVGELINDL-
18145401        INQNIPNSLNDLIMKAMEKDPVNRYQTAKEIIGDL-
15024695        LNKDIPIALNKMILRCIEKKPENRYQNANEILDEL-
125973093       IKEDIPTGVNSIVMRAIQKDQALRYQSASELLNDL-
20807943        LNEKVPEELDKIVLKATQKDPNLRYQTASEFLKDL-
89895431        LNARVSPALENLVMRSIAKSPDQRYATAKDLLQDL-
116626609       -WDAVPPRVQGLLRRCLERDPRKRLRDIGDVGLL--
116626708       -LDAAPENLRPLLRRCLERDERKRLRDIGDAF----
                     .  :  :  :   :    *
```

Cluster No. 34 multiple sequence alignment

```
CLUSTAL 2.0.10 multiple sequence alignment


21220714        YRLERLLGEGGMGRVYLGRTPAG------SAVAVKVVHRAYAADPEFRRRFALEVAAARR
21222767        YRLAARLGAGGMGRVYLSHTRGG------RPVAIKVVRSELADDATFRRRFGREITAARR
21221774        YRLESRLGSGGMGVVHLARSTSG------MRVAVKVVHATYARDPEFRGRFRQEVAAARR
29831260        YLLEAALGSGGMGVVHLARSASG------LRLAVKVVHAQYAQDPEFRGRFRQEVAAARR
29830359        FEVLGRLGAGGMGLVYLARSASG------RRVAIKTVRTELAEDQLFRVRFTREVEAARA
21222037        YRLLGRLGTGGMGHVYLARSDRG------RTVAVKLVREELAALEEFRERFRHEVESARR
29831097        YRLLARLGAGGMGQVYLARSDRG------RTVAVKLVRQQLAEQDEFRARFRQEVQAARR
134098455       YRLLARLGRGAMGAVYLARSRGG------RVVAVKLVRPDLADDTEFRERFRREVQMARS
21222873        YRLLGRLGAGGMGRVFLGRSPGG------RLVAVKVVHAELLRRPEFRDRFRREVQAARM
54024334        YRLLGVLGAGGMGRVYLGRSAGG------RTVAVKVIRPDLVADPEFRTRFQREVAAARR
86741561        YRLANRIGAGGMGVVYLGFGTDG------RPAAVKVPSAGLADDPEFRSRFRHEVDAARR
86741562        FTIHNRIGAGGMGTVYLGFNADG------RAAAVKVPDARFADDPEFRERFRREVAAARR
86739363        YRLQNRIGAGGMGTVYLGFAPDR------RPVAVKVAAEDLAEDEEFRSRFEREVRAALR
86739076        YRLHNRIGAGGMGVVYLGFGPDD------QPVAVKVPHEVHASDPEFRARFRSEVSAARH
86739440        FTLLGRLGEGGMGTVFLGRGRPDVAEHAGRLVAVKVIRPDLARVPEFRARFRREADIARR
86741688        YQVIGRLGQGGMGTVFLGR-APD-----GSAVAIKMIRPELAQRPEFRARFAREAESARR
86739244        YQVVGRLGAGGMGTVFLAQDAAE------KFVAIKVIRSDLAADPEFRARFRDEVAAARR
86740152        FTLTARLGSGGMGVVYLGIDDETG-----GPVALKVIRSDFTADPEFRSRFRREVAAARA
86741129        HRVLGRLGAGGMGVVYLAEGPLG-------QVAVKLIRPEYADDPQFRARFHREVQACFR
29833341        FRLHRRLGAGGMGVVYLGSDRRG------QRVALKVIRPDLAEDQEFRFRFAREVSAARR
29830627        YTLLARLGAGGMGQVYLGRSPGG------RLVAIKVIRDEITDHPEALARFRREVETAGA
119717589       YTLLARLGEGGMGVVHLARREAG------ERVALKVLRPHIVGDEEARRRLAREVGSLSR
                . :   :* *.** *.*.              *:*              *:  *

21220714        VQGLYTVPVVA-ADLDADEPWLATAYAPGPSLQQAV-GERGPLPAAEVLALTAGVAEALE
21222767        VKGAYTAELID-ADPDGTPPWLATLYVPGPSLAGAV-ARSGPLPVPAVLWLMAGVAEALQ
21221774        VSGAFTAPVVD-ADPEAGRPWMATLFIPGPTLSEQV-KRNGPMDEPQLRRLMAGLAEALR
29831260        VSGAFTAPVVD-ADPGAGRPWMATLFIPGPTLAEHV-KRNGALPPARLRHLMAGLAEALR
29830359        VSGFYTATVVD-ADPRAAVPWLATAYVPAPSLEEIV-TECGPLPAQAVRWLAAGVAEALQ
21222037        VGGHWTAPVLD-ADTEAAVPWVATGYVAGPSLQQVVGHDHGALPERSVRTLGAGLAHALQ
29831097        VGGHWTAPVLD-ADTEAAIPWVATGYVAGPTLQTVVGSDHGALPERSVRILAAGLAHALQ
134098455       VGGFWTATVVD-ADTEAEQPWLATEYVPGPTLHQAV-ADHGALPEHTVRSLAAGLAEALA
21222873        VSGAFTAPVVD-ADPDAPLPWLVTSYIAGPSLEQAV-AERGPFDPQAVLTLAAGLAEALV
54024334        VGSSCTAPVLD-ADVAANPPWLATGYVAGLALSDAV-ERFGPLGEHSLLVLAHGLAEALI
86741561        VRGSAVAAVLD-ADLTGQRPWMATEYVEGRNLADAV-ATRGQLDDRLVQGLAVGLADALV
86741562        VHGRAVAAVLD-ADPEATSPWLATEYVEGTSLADAV-LRHGRLEERLLHGFSVGLADALI
86739363        VRGTAVAAVLD-ADTEAAAPWMVTEYVEGTSLAEAV-RARGRLEDHLVRGLAVGLADALV
86739076        VRADTVARVIR-AEVDGPKPWLATEYVAGPTLRAAV-QEGGPLTGRPLDGLAIGLAAALE
86739440        VARFCTAEVLGVVDPPDGRPYLVTEYIDGLTLAQTV-AADGPLRSADLERVAVSVAAALT
86741688        VRRFTTAAVLD-ADPYGPQPYLVTEFVEGPTLSRRV-SVRGPLRPADLEQLAVSVTTALS
86739244        VAPFCTAQVLD-ADPDARRPYLVTEYIDGVRLDQAV-TESGPLPLSTLQGVAVGVASALT
86740152        VDGACTARLVD-ADPDAEDPWMATEHIHGQSLAEAI-ADRGALAMPVVMALATGLAEALK
86741129        VGGAHTARLVD-FELEAERPWLATEFVDAPDLAAQV-AAAGPLSTGEQIILAAGLAEALA
29833341        IRGGCTARLVA-ADLDADRPWFATQYVPGPSLHDKV-AEEGPLSAADVAAVGAALSEGLV
29830627        VRSAYTANLID-ASLDAAPYWLATEYVSGPTLGGAV-TERGPFPPDSARRLFAALAEALA
119717589       VRSQWVAEIVD-ADPWAEVPYVATRYVPGLSLHDHV-VEEGAITGADLLWLASCLAEGIA
                :    .. ::   .      :..* .  .  *   :    * :       .   :: .:

21220714        TIHAAGVIHRDLKPSNIVLTADGPKVIDFGIARAAD---VTALTATGMRAGT---PAYM-
21222767        AIHAAGIVHRDLKPANVLLAADGPRVIDFGISLAAD---STAHTATGTTIGT---PQYM-
21221774        DIHRVGVVHRDLKPSNVLLAEDGPKVIDFGISRPKD---SELRTETGKLIGT---PPYM-
29831260        DIHRAGVVHRDLKPSNVLLAEDGPKVIDFGISRPSD---SELRTETGKLIGT---PPFM-
```

```
29830359        SIHGAGLVHRDLKPSNVLVVEDGPRVIDFGIASGVS---NTRLTMTNVAVGT---PAYM-
21222037        DIHAAGIVHRDLKPSNVLVTIDGPRVIDFGIARALQTVADGGLTRTGALVGS---PGFM-
29831097        DIHAAGLIHRDLKPSNVLVTIDGPRVIDFGIARALETVTDVRAQSHRKPARSSVRTGFHG
134098455       AIHRADLVHRDLKPANVLLGPDGPRVIDFGISRAMT---GNALTATGMFLGT---PGFF-
21222873        SIHAAHLVHRDLKPSNVLLAEDGPRVIDFGIVRSVD---ADSLTGSGHMAGS---PGFM-
54024334        AVHAAGVVHRDLKPSNVLLALDGPKVIDFGIARAMD---DTSLTTTGKVIGS---PAFM-
86741561        AIHAAGVVHRDLKPANILLTWDGPKVIDFGIARAGD-NTS--HTRTGMLIGT---LVWM-
86741562        AIHAAGVVHRDLKPSNILLAWDGPKVIDFGIARASG-IPS--HTRTGILIGT---LAWM-
86739363        AIHAAGVVHRDLKPSNILLAWDGPKVIDFGIAHLTD-SAT--LTRTGHVIGT---LAWM-
86739076        AIHAASVVHRDLKPANIVMSWAGPKVIDFGVARSAD-YTG--YTQAGELVGT---VVWM-
86739440        AIHGAGLVHRDLKPSNVLLSALGPRVIDFGIARALD-APTMLSQEIQR-IGT---PAFM-
86741688        AIHAAGIVHRDLTPGNVLLSPVGPKVIDFGLAREFN-ADTDLSHNVRHAIGT---PGYM-
86739244        AIHRAGIVHRDLKPSNVMLSYSGPRVIDFGIARTLD-MTKGRTQ-TGLVLGS---VGWM-
86740152        SIHDAGIVHRDLKPGNVILSEDGPKVIDFGIAAAVD-ATA--ATRTGVLLGS---PGYM-
86741129        SIHAAGLIHRDLKPSNVLWTADGPKVIDFGIAAAAE-ARP--LTAVGGVVGT---PGWL-
29833341        AVHEAGVVHRDLKPSNILLSPKGPRIIDFGIAWATG---ASTLTHVGTAVGS---PGFL-
29830627        SVHGYGVTHRDLKPQNVILSGQGPLLIDFGIARNTT---DTALTQTGLAPGT---PGFT-
119717589       SVHAVGVLHRDVKPSNVLMEGRTPILIDFGLARVAD---DPKLTHTGWLLGT---PGYL-
                 :*   : ***:.* *::     * :****:                    :     :

21220714        APEYIR-GQEVTEAGDVFALGLVAHFAATGRLAFGGGSD--HGVAYRILEASP--DLDGC
21222767        APEQAS-AGAITAATDVFSLGQTAAFAALGKPLYGDGPA--ATVLYRIVHSEP--DLSEL
21221774        APEQFRRPREVGPAADVFTLGSLMVHAATGRGPFDSDSP--YVVAYQVVHDEP--DLTGV
29831260        APEQFRRPREVGPAADVFALGSVIVHAATGSGPFDSDSP--YLVAYQVVHDEP--DLTGV
29830359        SPEQAKDSRSVTGASDVFSLGSMLVFAATGHAPFHGANP--VETVFMLLREGP--DLEGL
21222037        APEQVR-GDRVTPACDVFCLGSVLAYAATGKLPFGSANSGAHALMFRIAQEEP--DLEGV
29831097        HPEQVR-GDRVTPACDVFCLGSVLAYAATGALPFGTASSGVHALMFRIAQEEP--DLTGV
134098455       SPEQTV-GNEVGPPSDVFSLGAVLVFAATGMGPFGNENT--AAMLYRVVHTEP--DLDEV
21222873        SPEQVN-GDEVTWASDVFCLGAVLAFAATGTNPFGAGPT--PALLYRVVHNAP--DVAAV
54024334        SPEQVT-GEPIGPAGDMFALGGVLAYAAAGQGPFGTGDT--VQLLWRVVYEEP--RIEAV
86741561        APEQLRGE-RAGPAADIFAWGACVTFAAAGRPPFRGERA--EAIGLQILTAEP--NLDGL
86741562        APEQLRGE-RAGPPADVFAWGACVTYAATGHPPFASEQS--DVLTRMREDRPP--DIAGV
86739363        SPEQMRGE-PSDASADVFAWASCVTYAATGRHPFHAETP--DLLAVRVQRDSP--DLYAL
86739076        APEQINGQ-QAGSAADVFAWGCCVVFAATGRRPFRGEAP--EIVALHISSTEP--ELDGV
86739440        APEQANGE-PVTAAADVFAWGGLVTYAGTGSFPFGDGPT--PVQLYRVVHREP--LLDGL
86741688        SPEQILDA-PITSAVDIFAWGAVIIFAATGHAPFGTGRI--DAILYRIVNEPP--RLDGV
86739244        APEQMEGA-ALGPAVDVFAWGLLIGYAATGGHPYGHGTY--LEMSEKILTGQP--DLRAM
86740152        APEQVTGRGEIGPPADVFAWGLTVLFAASGRPPFGAGRP--DALLYRVVHDEA--DTGDV
86741129        SPEQATGG-EVTAASDVFGWGALVCFAATGQPPFGSGSA--DAVASRIAAAEFQIDFDRL
29833341        APEQVR-GAAVTPATDVFSLGATLAYAATADSPFGHGSS--EVMLYRVVHEEA--HLNGV
29830627        APEVLTHN-QVTSAADVFALGATIANAATGRPPFGSGPA--HAVSYRAVHEDI--DVDGV
119717589       APEILY-GEDATVASDVHSWAATVAYAGTGHPPFGRGPS--MAIMDRVRRGQF--DLDGL
                 **          . *:.  .     *. .   :

21220714        P-ESVRGVVALCLEKDPARRPTPAEIVRL-
21222767        P-ERLRDLLGRCLATAPEERATPAEIVEW-
21221774        P-DSLAPLVLRCLAKEPEDRPTPDELMREL
29831260        P-EELAELVAGCLAKEPDDRPTPDELMTAL
29830359        P-DELRPLIESCMQMEATGRPNPADLQAQL
21222037        P-EGIADLVRDCLRKDPAARPALADVLER-
29831097        P-EGLADLVRECLRKDPAARPPLERILER-
134098455       P-DGLRPLLASCLAKDPAQRPTPAQLLDGV
21222873        ADPALRSLIADCLAKDPAHRPAPREILARI
54024334        P-PRLRPAVAACLAKNPADRPTPRQVLDQL
86741561        P-ASLVGVVRAALDKEPARRPAATELLARL
86741562        P-VKLAPLVRAALGRRPEERPSAAELVRSL
86739363        P-GYLLNQVARALSKAPRERPDAASLLAAL
86739076        P-ERLLGPVRQALTKNPGHRPSAGELVRLL
86739440        A-PALRPIVEEAMRKDPATRPSAQELFLRL
86741688        G-GELRNLVEIAMAKDPAARPSAEELRTAL
86739244        P-PDLTPIVRSALARDPRDRPSTENLLLTL
86740152        P-PALRPAVRAALLKEPMARPSAHALLRVL
86741129        A-PELHAPVREALDRQPPQRPTALDLCERL
29833341        P-DALAPLVRACLAKDPEERPSTLQLSLRL
```

```
29830627        D-PELAALIQACVAKDPVERPGLETV----
119717589       P-HDLHQVVAAALDPDPRERPSLEQILAWL
                    :   :  .:   .  *.    :
```

Cluster No. 35 multiple sequence alignment

```
CLUSTAL 2.0.10 multiple sequence alignment


21221541        ------------MGQVWTAYDRRLDRRVAVKLLRPDKVAGAEADELRRRFVRECRITAQV
29830083        ------------MGQVWTAYDKRLDRRVAVKLLRPDKVAGQEADELRRRFVRECRVTAQV
134103045       YELDELPLGKGGMGAVHRGHDLHLDRRVAVKFLH--LPGG-PDEELEQRFIREARILARL
86741364        YELEAVPLAKGGMGEVWVGRDVKLDREIAVKFVR--FPDGQPDQELIRRFIRESRITARL
117928194       YLIEDR-IARGGMATVYRARDMRLDRPVAVKVMH---DVFATDPEFVARFIREAKAAAAF
                            *. *  . * :*** :***.::          *:  **:**.:  * .

21221541        DHPGLVTVHDAGSEGEELFLVMQYVDGADLSDHLAEHDPYPWQWAVAVAAQLCAVLSAVH
29830083        DHPGLVTVHDAGSEGEELFLVMQYVDGADLADHLAEHTPYPWQWVVAVAAQLCAVLSAVH
134103045       EHAGAPTLYDFGAYDHRLFQVMQFVDGVTVADLISEHGPVPVPWAAAVAAQACAVLSAAH
86741364        LHPGVPAVYDAGTHEERPYLVMQRVHGLSVADLVAEQGALPVGWAAAIAAQVASVLAVAH
117928194       SHPNVVAVFDQGADGGHVYLVMEYVVGETLRDLLRRRGRLSPAEALGILQPVLAALSAAH
                 *..  ::.* *:   . : **: * *  : * : .:   .   . .:     :.*:..*

21221541        AVPIVHRDLKPRNVMVKQDGTVTVLDLGVASVMD-ADTTRLTHTGTPIGSPAYMAPEQAM
29830083        AVPIVHRDLKPRNVMVKQDGTVTVLDLGVASVMD-TDTTRLTHTGSPIGSPAYMAPEQAM
134103045       ALSICHRDLKPTNLMLCPDGAVKVLDFGLAVLRD-TDVAQFTRAGQILGTPAYMAPEQIQ
86741364        RASLVHRDLKPANLMLEPDGTVKVLDFGLAVALDRTNLSKITVTGQHLGSPAYMAPEQVL
117928194       ASGLIHRDVKPENVLLARDGRVKVADFGLAQAVN----RTASRTATLIGTVAYLAPEQVT
                   : ***:** *:::  ** *.* *:*:*   :       : :.  :*: **:****

21221541        GGAVGPYTDLYALGVLLHELLSGDVPFAGSTALGVLHRHLYEPPLPVRRIRPEVPEALEA
29830083        GGAVGPYTDLYALGVLIHELLSGDVPFTGSTALGVLHRHLYEPPLPVRRLRPEVPEALEA
134103045       QGVAGPRSDLYALGCVLHEMLTGRQLFTGPTAYAVFEKQVKQAPPPVHG----VPAELDD
86741364        AGLSTPATDIYALGATLFEMLTGQRLFAGASSYTVMNKQVEEVPARVRSLRADVPRGLDE
117928194       RGVADARSDVYAAGIMLFEMLTGAPPYQGDSPLAVAYRHAHEDVPPPSSRVQSLPPAIDA
                 *   . :*:** *  :.*:*:*   : * :.  *  ::  :           :*  ::

21221541        LVLRLLAKDPQHRPDSAQEVYEHL
29830083        LVLRLLAKDPQHRPASAQETYEQL
134103045       LVQDLLKKDPEDRPADAGVLYERL
86741364        LVAAMLSKAPEDRPCGVEKV----
117928194       VVLAATARDPDRRPADAHALLALV
                :*     : *: ** ..
```

Cluster No. 36 multiple sequence alignment

```
CLUSTAL 2.0.10 multiple sequence alignment


32471012        YTLREQIGEGGFGLVFVAEQES-PVRRRVALKIVKPGVGSKEVIARFEAERQAVALMNHP
32477437        YQLMERIGEGGFGWVFVAQQRS-PVQRRAALKIIKPGMESREVIARFEAERQAIALMDHP
32476285        YSICELIGEGGMGSVFVAQQEQ-PVRRKVALKIIRAEIATKEALARFSAERQALAMMDHP
94969337        YRLIDLLGEGGMGEVWMAEQVE-PLRRTVALKLIKAGMDTKAVVARFESERQALALMDHP
116620331       YRIVQLIAEGGMGAVYQAVRVDDLYRKVVAVKVIRRGVFGEFALRRFDIERQILAHLDHP
116620490       YQMVREIGHGGMGTVYLAERADGEYRMQVAIKLVSPHLCTEAVLRRFRTERQVEASLDHP
                * :   :..**:* *: * : .   :  .*:*::   :  . .: **  ***  * ::**

32471012        NIAQVFDAGVTADYRPYFVMELVRGLPITEFCDEKQMDVRERLNLMIDVCSAVHHAHQKG
32477437        NIARVFDAGVTETAQPYFVMELVRGVPLTDFCNSNRLRISDRLQLFVTICHAVQHAHQKG
32476285        CIAKVLDGGATESGQPYLVMELVQGTPITEYASHSGLSIEQRLRLFQKVCHAVQHAHRKG
94969337        NIARVFDAGSTPEGRPYFVMEYVPGQPITAYCDRQKLALKQRLELFIQVCEGVQHAHQKA
116620331       NIAKLLDGGATPDGRPFFVMDFIAGTPIDEYCDHHKLGIRERLDFFLKVCSAVHYAHQNL
116620490       NITRLLDGGTTDDGLPYLVMEYVDGVRIDAWCDSRKLSVRDRLKLFRQVCAAVQSAHEKE
                 *::::*.* *    *::**: : *  :  :..   : : :** ::  :* .*: **.:

32471012        VIHRDIKPSNVMVTLHDDRAVAKVIDFGVAKALD-QKLTDKTVYTRFFSMIGTPLYMSPE
32477437        IIHRDLKPSNILVTLQDGRPLAKVIDFGVAKAIG-QSLTVKTIYTRFTSMVGTPAYMSPE
32476285        VIHRDLKPSNILVAEIDGEALPKVIDFGLAKALD-QPLTDITIHTGFAQLMGTPMYMSPE
94969337        IIHRDLKPSNVLVQEIDKRPVPKIIDFGLAKATG-QRLTEATMYTEVGARVGTPAYMSPE
116620331       VIHRDLKPGNILVTEEG---ALKLLDFGIAKLLDPDALAGEDEPTLTTVHAMTPEYASPE
116620490       IVHRDIKPGNILVTADG---TIKLLDFGISKVLN-RELFDTPETTLG-ETPMTPEYASPE
                ::***:**.*::*   .     *::***::*  .   *      *       ** * ***

32471012        QAEMSSLDVDTRSDIYSLGVLMYELLVGATPFDRGRLDSAGLDEMRRIIREEEPPRPSTR
32477437        QAAMSTDDVDTRSDIYSLGVLLYELLTGSTPFAPDRLQTAGFDELRRIIREEEPPRPSTR
32476285        QAEMGTIDIDTRSDVYSLGVLLYELMVGAPPFDRETFKTASFDEVRRIIREIQPPRPSVV
94969337        QADANERNIDTRTDVYSLGVILYELLTGVLPFEP---QDTTADEMLRKIRDEEAPRPSTK
116620331       QLHG--LQVTTASDVYSLGVLLYRLMTGHRPYAGDS---RSIEELWEHIRSRPPRRPSTV
116620490       QIRG--LRVGPATDIYSLGVVLYQLLTGSLPFASQ----REQRQVMRAICEEEPLKPSAA
                *       : . :*:*****::*.*:.*  *:           :: . * .  . :**.

32471012        LSTLT----AERIRSLGLQNHGAGASQSRSLSSDLRGDLDWIVMKALEKDRTRRYDSAAS
32477437        LSTLN----SEESRTSDVERLGP----PTTPSTAMKRDLDWIVMKALHKDRNRRYSTAGA
32476285        SRTLS----ANENVN---HQQGD---VQRKLHPSIRGELDWLIMKTMEKDRRRRYGSASE
94969337        LRSST----QNAVKVAEQRQEQP-----QTLIRHLRGELDWIVMKALEKDRGRRYGTPTE
116620331       IAAN-----DDGVTPETVCSARS--TKPERLQRQLSGDLDNILMMALRKEPERRYCSVEQ
116620490       IHQETVLAGQQGMVAETTSQARG--ESPTGLRRLLSGDLDNIVLKALRKEPERRYPTVRA
                          :                       :  :** ::: ::.*:  *** :

32471012        LANDI----
32477437        LAEDILRFL
32476285        LADDI----
94969337        LAADV----
116620331       FANDLRRHL
116620490       LSEDL----
                :: *:
```

Cluster No. 37 multiple sequence alignment

```
CLUSTAL 2.0.10 multiple sequence alignment


37521672        YRLCRHLG--VNGGRETWLAADGLCAEVSVTLKTLYFG-RSATWQDHERLEREARTLASL
37521696        YRLVERLG--HNVHRQTWLAGDERTAEQ-VVLKALAFD-NEMQWQQLKLAEREAATLQSL
37519991        YLLERQLG--STGARQTWLVQNSATGQA-LTLKALYFG-TGMDWRNLALFEREAQTLKSL
113475351       YQLQKQLGR-AVAGRKTWLAFDSQLEEQ-VIIKMLAYS-PEILGQELDLFETEAKILKAL
17133011        YEIQQLLG--KKAGRRTLLAKDVVTGEL-VIVKLLVFS-SDFEWDDLKLFEREAETLKSL
22298642        YELLRELG--SHPGRRTYLALDRWHYEQ-VVLKLLLFG-GGITWDDFKLFEREVAVLQTL
17131853        YQVIQELGRNQEEGRITYLAHHHQSKQQ-VVIKEFSFAHTYTDWCDFTAYESEAKILQKL
                * : . **      * * *. .    :  : :* : :        :    * *.  *  *

37521672        DYPGIPRHRDAFWVELPEGH---YYCLAQEYIAGITLAERVGSGGRLGEADIVRLAANLL
37521696        THPCIPRLVESFWLELPEGH---YFCLVQSYIPGQSLAAMLRAGRRWSPGEVLNIARQVL
37519991        DHPRIPRYHEFFQWQQPEGD---YFCLVQDYIPGVSLAEQVHSGKRWSEAQIEQAALEIL
113475351       NHPHIPRYRDYFSLEKEANLDLPWFALVQNYIPGFSLQELLDRDKIFTEEDVEKIAKEML
17133011        SHPHIPRYLDYFEVNPPTIKG---FALVQSYISAQTLEQYLQSGRSFSESDIKQIATAIL
22298642        DHPAIPRYLDYFEINTPDLKG---FALVQTYIEAKSLALWQAEGRVFSEGDLRLLADRLL
17131853        NHPRIPRYLDSFATQT-------GFYLVQEYKHALPLS----SKRSFQPEEIKQIAVSVL
                 :* ***  : *  :         : *.* *  . .*             ::   *  :*

37521672        ETLAYLHSQAPPVIHRDIKPSNIVCTAD-----GGYALIDFGSVQAQSS--TATLTVAGT
37521696        EILDYLHSQSPPVVHRDIKPSNIILSDD-----GRLFLIDFGAVQAQSLP-DQTVTVVGT
37519991        EILDYLHSLAPPVVHRDIKPSNVICGED-----GRLYLVDFGSVQAEQVS-GRTVTVVGT
113475351       EILIYLHELKPSVLHRDIKPSNLIMGAD-----QKIYLVDFGAVQSQGNAQGVTFTIIGT
17133011        EILIYLHGLYPPVIHRDIKPSNILLGERSGNNVGQVYLIDFGSVQTALGAEGGTRTVVGT
22298642        DILIYLHSRQPPVIHRDIKPTNILLGDRSGHDLGKVYLVDFGAVQTAI--AGSTRTVVGT
17131853        EILVYLQQRIDPIIHRDIKPENILVDEQ-----LNAYLVDFGLARVQDTKIALTSLLTGT
                : * **:    .::****** *::             *:*** .:        *  : **

37521672        FSYMPPEQFIGRAAP-GSDLYALGATLLFALTGTDPADFP----RRGLHLIFQSRVGVGR
37521696        FGYMAPEQFYGKTSP-ASDLYSLGMLLLCILTGTEATEIP----RRGLQVELPSTSLVEQ
37519991        YGYMAPEQFGGRAVP-GSDLYSLGATLVHLATGMNPADLV----DGGFHIRIPEQLPLSP
113475351       SGYAPLEQFWGRAVP-ASDLYALGATLIHLLTGISPVDLP----QKNYHIQFPENVNINP
17133011        YGYMPPEQFGGRTVT-ASDLYSLGATLIYLVTGTHPADLP----QKDFRIQFESVANLSP
22298642        YGYMPPEQFGGKTLP-ASDLYALGMTLIYLATATPPDELP----QKELRVQFQPLVSLSP
17131853        PGFIPPEEQLGYSLSLASDLYSVGATLICLLANIRSVDIGKLIDSKGYFDFQKLDSQIDL
                 .: . *:  * : . .****::*  *:   :   . ::                  :

37521672        PLVRWLERLLEPALEERLGNA---REALETL
37521696        PMRAWFACMLAPEHQDRFDSA---REALESL
37519991        GLRHWVEKLVDSDPERRFKNA---REAI---
113475351       SLANWINKITEIDLEIRFKNA---REALKCL
17133011        GLAAWLKTMTEPSVERRFSSP---QQALKAL
22298642        PFIRWLEKMVAPALEERFDSATAARHALKQL
17131853        RFRSWLMGMVEPKWQYRYTNA---ADALAAL
                 :  *.  :     : *  ..    .*:
```

Cluster No. 38 multiple sequence alignment

```
CLUSTAL 2.0.10 multiple sequence alignment


13472161        YELDERIASGGMGEVYRGHNIQTGDHVAIKIVLPEFARDQTILSLFRKEASILNHLSHDA
27351864        YEIEAVIGAGGMGEVYKCREIQTGSPVAVKMLLPDMVDNEAALALFRREAAALHNLPHDA
126464610       YRVEARLGQGGMGTTWRGVNLTTQDVVAIKVMTPEIALVPKAVELFRREAQLLRKVQSDA
119385168       WTVEGVLGRGGTGEVYRAKSLVTGRVVAIKALSAGFSGDEGYLELMRREE-AMRDILHDA
                : ::  :. ** * .::  .: *   **:* : . :      : *:*:*   :..:  **

13472161        VVRYHVFTIDPRIGRPYLAMEFVDGQSLFDVMRRGP-MRGEEVRRLCHRLASGLAAVHQA
27351864        IVRYFLFTVEPVLQRPYLAMEFVNGRSLSNMLDDGP-LTFEALIKLMLRVASGLEAAHEH
126464610       VIRYET-TLQDREGRLYLIMEFVDGQPLSHYLERGARLAPGDVLRLGRRMAGALAAIHAL
119385168       VVRYSDCSRTP-EGHVFLVMDFIDGPALSEAMAERR-LGMRELLIVAHRVAEGLVAAHAR
                ::**   :      : :* *:*::* .* . :     :    :  :  *:* .* * *

13472161        GAVHRDLSPDNIILPGGRVESAKIIDFGIARSAAVGGETLIGGKFAGKYNYVSPEQLGLY
27351864        GIIHRDVSPDNIIVPLDDVRRAKIIDFGIARSTQMGDKTIIGSGFAGKDNYASPEQVGLY
126464610       GIVHRDVAPDNIMLPDGDIGHCKLIDFGLASNTSGTEASIIGASYAGKYSYSSPEQFGLH
119385168       GVVHRDLSPDNVILRDGRVEGATVIDFGIAKDTAAGAHTVVGNQFAGKYEYAAPEQF---
                * :***::***:::  . :  ..:****:* .:     :::*  :*** .* :***.

13472161        GGEVSEQSDIYSLGLVLAAALRGKPLDMSGSQYEVIEKRR---TVPDLSDIDPDFQELIA
27351864        GNDVTAKSDVYSLGLVLFHALTGQKLDMGGSQFQLVEKRR---RVPDLGAVDMRIRPLLE
126464610       GARVGPASDIYSLGLVLMKVC-GLKVPGEGMGMAAVEVRRENVTIPRDAGLPPALARLLE
119385168       DGKAEPASDLYALGALLLAAARGEVPFAGATPGEMIRRKA---ETLDVTGLPEPLAGLIL
                .  .   **:*:** :*  .  *      .     :. :          :   :  *:

13472161        AMLQPDPRDRPASMAEIARA-
27351864        RMLQPDPALRPT-MGEVASW-
126464610       AMLRTRPDERPADLQKLF---
119385168       WLSAPRIAERAPSAAAVVARL
                 :  .    *..    :
```

Cluster No. 39 multiple sequence alignment

CLUSTAL 2.0.10 multiple sequence alignment

```
126435516        YVLRGVLGAGGMAEVRDAWDTRLDRPVAIKILHPAM----NAQPETLNRFRDEARSAAML
126438334        YELREVLGLGGMAEVRDGWDTRLDRAVAIKLLHPAM----RAQPDVRSRFEAEARSAAAL
120406349        YELRGLLGCGGMAEVRDGWDIRLDRPVAVKLLHPNF----NADADARARFCDEARSAAAL
145221981        YELRGLLGRGGMAEVRDGWDTRLDRSVAVKLLYPSQ----SSDDSVRRRFEDEARSAARL
111017717        YELRGVLGRGGLADVYDGWDQRLARAVAVKVLRPEM----ASEPQTRRRFESEARLAATL
111018704        ------------------------------------------------------------
120406030        YELRGVLGRGGMAEVRDAWDKRLGRPVAVKLLYPSV----SAHPDTRRRFAIEARAAAAL
145222263        YRLGPVLGRGGMAEVRDAWDQRLGRPVAVKLLYPSV----STQPDTRRRFATEARAAAAL
108805087        YRILRTLGKGGMGRVYLAHDEVLDREVALKVLYRDL----AEHEDFVERFKREARSAASL
21223157         YRLHERLGRGGMGIVWRATDQLLAREVAVKALPLDESLSAAEARRRRERTLREARAVAQL
41406116         YRLQRLIATGGMGQVWEAVDNRLGRRVAVKVLKQEF----SQDPEFIERFRAEARTTAML


126435516        SHPNIVAVHDYG----EQDGT-PFIVMERLPGRTLHDVMA-DGPLAPDQVRSMLDDVLAA
126438334        THPNIVAVHDYG----EDDGT-PFIVMERLPGRTLGDVIA-AGPMSPAQVRSMLGEVLAA
120406349        SHPNIVAVHDFG----EHDGR-PFIVMERLPGRTLADLIA-QGPMAPAQVRAMLDDVLAA
145221981        SHPNIVAIHDFG----EHDSA-PFIVMERLPGRTLADVIE-QGPMPAPQVRRVLDEVLAA
111017717        NHPNVVAVHDSG----DDHGA-PYIVMERLPGRTLLDDLL-AGPLPAPRARTILTQTLDA
111018704        -------MHDCG----EDAGV-AFIVMERLPGRTLADEIA-AGPVPDARVRSILADVLAA
120406030        NHPHVVAVHDSG----VHDGR-HYIVLERLPGQSLADVLAHHGPLPVDHVRAIMRDVLSA
145222263        NHPHVVAVHDTG----VHEGR-HYIVLERLPGQNLADLLARHGPLPVEQVRAILRDVLSA
108805087        SHPNIVSVHDLG---VTGDGS-HFIAMEYVSGGTLEYLLKREGFLPPERARRIAAQIASA
21223157         RHPHVIVVHDV----VEDDGR-AYMVMELVDGGSLADRVLTRGPVDAVEAARIGVALLDA
41406116         NHPGIAAVHDYGESQLDGEGRTAYLVMELVNGEPLNSVLKRTGRLSLRHALDMLEQTGRA
                        :**         .   ::.:* : *  *   :   * :   ..  :      *

126435516        LSVAHSAGVLHRDIKPANILLSVTGDSVKVADFGIAKAGG-AAHTMTGQIIGTMAYMSPE
126438334        LEVAHAAGVLHRDIKPGNILLSADANTLKVADFGIAKTGG-AAYTMTGQIIGTLAYMSPE
120406349        LDVAHTAGVVHRDIKPGNILLSATGDRMQVADFGIAKSGG-AAHTMTGQIVGTLCYMSPE
145221981        LSVAHAAGVLHRDIKPANILLTTAGDSVKVADFGIAKSAG-AAHTATGQIVGTLCYLSPE
111017717        LGAAHAAGILHRDIKPGNILFDAHG-TVKVTDFGIAKSAD-TTHTTAGEVLGTVAYLSPD
111018704        LGAAHGAGILHRDIKPGNVLFTAAG-TVKVADFGIAKSAA-SDHTATGQVLGTVAYLSPD
120406030        LRAAHTSGVLHRDIKPANILFTHFG-GVKIADFGVAKSAD-TPQTLTNRVFGTMAYLPAD
145222263        LSAAHLSGVLHRDIKPANILFTEFG-GVKIADFGVAKSPD-TPQTLTNRVFGTMAYLPSD
108805087        LGAAHERGVIHRDVKPQNVLLTGSG-DAKVADFGIARAAALTTLTGTGFVLGTARYISPE
21223157         LDTAHASGILHRDVKPSNVLVADDG-RVVLTDFGVAQVAGATTLTESGSFVGSPEYTAPE
41406116         LQVAHAAGLVHRDVKPGNILITPTG-QVKITDFGIAKAVDAAPVTQTGMVMGTAQYIAPE
                 * .**  *::***:** *:*.   .    ::***:*:    :  * :. ..*:  * ..:

126435516        RVTGAPASVADDLYAVGVVGYEAIVGHRAFPQDSPVAVARAIMDDPPPPLREVRPDLDPV
126438334        RVSGAPASVADDLYAVGVMAHEALLGRRAFPQDNPAAVARAIMDDPPPPVTAFRTDVDPI
120406349        RVSGAPASVADDLYAVGVMGYEALLARRAFPQESPVALVRAIVDAPPPALAGLTGGADPV
145221981        RVMGAPASVADDLYAVGVMGYEALLGRRAFPQDNPAALARAIIDVPPPPLRALSVTADPA
111017717        RITGKPASVTDDLYAVGVVGYEALAGHRPYTGDTILSLAHAIVHGHHEPLTAVRPDLAPT
111018704        RILGTPATTADDLYAVGVVGYEALAGHRPFAGDNILSLARAITDGAARPLRDARPDADPN
120406030        RIAGRPATPSDDLYALGVVAYEALTARRAYPQENLTALADAIAAGHLAPVATLRPDVDPA
145222263        RIAGRPATPSDDLYALGVVAYEALTGRRAYPQDNLSALADAIAAGRLAPLTSVRPDVDPA
108805087        QARGEPVGPPSDLYSLGIVLYEMLTGEVPFDAETPIGLAMKHMSDVPRPPKEANPAVPDD
21223157         RMSGAGTGPESDLWSLGVLLCAVLSGASPFHRDSLGGVLHAVVTEEIRPPAQAGPLLP--
41406116         QALGHDATPASDVYSLGVVGYEVVSGKRPFSGDGALTVAMKHIKEP--PPPLPAELPPN-
                 :  *  .   .*::::*::    : .  .:  :    :          .

126435516        LAGTIDRAMTRDPAQRFGDAAQMRAAV
126438334        LAGVIERAMTPDPPQRFGSATQMRAAL
120406349        LAAVIDRAMSRDPRRRFGSAAQMRAAL
```

```
145221981       LVAVIDRAMSRDPRLRFGSAPQMRAAL
111017717       LTTVIERALSCDPQQRFATADQMRNDL
111018704       LVHTIERAMARDPQQRYADAQSMRDAV
120406030       LATTIERSMARDPRWRFATADQMLACL
145222263       LAATIERAIARDPRWRFATAEQMRRSL
108805087       LNDLVMRLLSKAPEERPKDASALI---
21223157        ---VVRGLLERDPRRRLDAASAQRML-
41406116        ----VRELIEITL--------------
                    :   :
```

Cluster No. 40 multiple sequence alignment

```
CLUSTAL 2.0.10 multiple sequence alignment


116622280       YEILALIGTGGMGEVWKAR-DTRLKRTVAIKVSKS------NFINRAEHEARAVAALSHP
116622762       YELLTPIGEGGMGQVWKAR-DTRLGRTVAIKVSKT------SFSERFEREARAVAALSHP
116623587       YEIVGLLGTGGMGEVYAAV-DTRLGRKVAIKVSQA------RFSGRFEREARAISALNHP
94971261        YEIVGPIGNGGMGEVYKVR-HTISQRTEAMKVLLSGAARRPEVTDRFVREIRVLANLNHP
116622368       YSITGLIGRGGMGEVYHAVREDDFRMPVAIKLLKRGGG-TEMALRRFRIERQILAGFQHP
                *.:   :* ****:*: .  .       *:*:             *   * : :: :.**

116622280       NICMLY-----DIGPDYLVMEYIEGKPLQGPMPVETA----LRHAIEIAKALDAAHRVGI
116622762       NICTLY-----DVGPDYLVMEYIEGKPLKGPIPVETA----LRHAIEIAKALDAAHRLGI
116623587       NICTLH-----DVGPNYLVTELVDGETLRDWLKRATAGERYLDVARQVLQALCAAHRAGI
94971261        NIAALHTA-FHHEDQLIMVMEFIEGKNLSEMLSTGMVLRDSVAYIRQAVTALAYAHSQGV
116622368       NIGRLLDGGATASGLPYLVMEYIEGVPLLEYAAGLPVRER-LRLFLAVCSAVQYAHQRHV
                **  *        .   :* * ::*  *        .    :        *:  **   :

116622280       IHRDLKPANILVTKSG-IKLLDFGLAKVT---AASAVSDETVTRGLTEEGSILGTLQYMA
116622762       VHRDLKPGNVLVTRTG-IKLLDFGLAKVTT--AAAAASEETVTRALTEEGTILGTLQYMA
116623587       VHRDLKPANIMVRFDGYVKVLDFGLAKWITPGGHSVQLEPPATMTLSQPGQIVGTVAYMS
94971261        IHRDIKPSNIMINSAGQVKLLDFGLALMS-----------TPDPRLTSSGSLLGSVHYIS
116622368       VHRDIKPANILVTVDGIPKLLDFGIAKLLHP------STADRGPSPTAADNAVMTPEYAS
                :***:**.*:::   *  *:****:*                    :  .  : :  * :

116622280       PEQLAGGEADARSDIFAFGCVLYELLSGEPAFAAASRAGIIAAILEREPKA----LPAAP
116622762       PEQLEGKEADARSDIFAFGCVLYEMLTGAPAFTGGSRASIIAAIMDREPKA----LPEAP
116623587       PEQILGQEVDQRSDLFAFGIILYEILSGLHPWPRKSAVDTMHAILHDEPAS----MHAIP
94971261        PEQIRGETMDARSDLYAVGVTLFEVITGRLPIQGHSFSEIINGHLQVIPPSPAVLNACIP
116622368       PEQIRGEPVTIATDIYSLGALLFEVLTGRQAPRPGN----------GEPAA-----HEMD
                ***: *      :*:::.*  *:*:::*  .    .            * :

116622280       AHVAATLQRCLAKDPDDRWQSARDL----
116622762       ASLESAIRQCMAKDPDERWQSARDL----
116623587       ANIARIVRRLLRKNPTDRFASAEEVLDAF
94971261        ANLAAITLKALAKNPSERFQNASEFLQAL
116622368       AELAAILRKALRQQPKERYASVAELSDDV
                * :     : : ::* :*: .. :.
```

Cluster No. 41 multiple sequence alignment

```
CLUSTAL 2.0.10 multiple sequence alignment


116622459       YEILARIGEGGMGEVYRATDTRLHRTVAVKISC------DQFGERFEREARAVAALNHPN
116625320       YTLISPLGEGGMGEVWKARDTRLDRAVALKVSK------AAFTERFEREARAVAALNQPN
116622825       YEILALIGKGGMGEVYRAHDPRLRRDVAIKISS------VRFDQRFEREARAIATLNHPN
116626668       YEILAPLGAGGMGEVHRALDTRLNREVAIKVSQ------ERFSDRFEREARAVAALNHPN
83647568        YKIIRKIGEGGMGWVYKAEDTRLERYVALKCLAPEVATDERVRFRLMQEAKAISRLDHPN
                * ::  :* **** * :* *.** * **:*           .  *: :**:*:: *::**

116622459       ICQIYDVGPN-----YLVMEYIEGLPLR-----GPLPLDQALRYAAQICDALDAAHTKSI
116625320       ICTLHDVGPN-----YLVMEFVEGVPLK-----GPLPVEKAVEYAGQILEALDAAHRKGI
116622825       ICQVYDVGPD-----YLVMELVEGITLKERIKEGAIPLEEALRIAGQIAEALSAAHERFI
116626668       ICHLYDVGPN-----YLVMELVEGESPK-----GPLSLEKALDYARQIADALEAAHEKGI
83647568        VCVIHDVGAMSDGGMYIAMQYYSGETLENALTKGALAPDQVVRIAKQIAYGLQAAHQRDI
                :* ::***.      *:.*:  .* . .     *.:. ::.:  * **  .*.*** : *

116622459       VHRDLKPANILVTRSG-VKLLDFGLAKIEQAVAA-----GSDTMTLALTGKGQILGTLHY
116625320       THRDLKPANILVTRQG-IKLLDFGLAKQRTPLPQ-----DGATLTRALTSQGEIVGTLQY
116622825       VHRDLKPDNIKIKPDGTVKVLDFGLAT-VGGAPAPLDSEDSPTSSMSLTQAGAILGTAGY
116626668       VHRDLKPANIKITPEGKVKVLDFGLAKRIQSDGTSYNSENSPTLTNTATQAGAILGTAAY
83647568        IHRDIKPANILLSEDGIVKILDFGIAK---------------LSGVDITLGGARMGTLRY
                 ***:** ** :. .* :*:****:*.                     *  *  :**  *

116622459       MSPEQLQGKDAGARSDIFAAGLVLYEMLTGKRAFDGDSPASVIGAILERQAPSIADVAPA
116625320       MAPEQLHGKDADARSDLFAFGCVFYEMLTGKRAFEGESAASVIAAILERE-PAPLTVAPP
116622825       MSPEQARGKPVDARSDIWAFGVVLYEMLTARRLFQGDDLTETLAAIVKEE-PNLASVPPQ
116626668       MSPEQARGKNVDKRADIWAFGVVLYELLVGERPFHGDDLTEILASVVKES-PDLSRVPLK
83647568        MSPEQLQGRDMGLQTDVWSVATLIYEMLTGREMFDGEKSSTVLHAVMHEEPDYSLAVFQE
                *:*** :*:  . ::*::: . ::**:*.... *.*:. :  : :::...      *

116622459       A--LDRILKRCLAKDSGDRWHSAADLKAAL
116625320       ---LDRIVRRALAKDPDQRFQTALDLKAAM
116622825       ---VRRLLEACLQKDPTRRLQAIGDRRLL-
116626668       ---VRRLLGACLEKDPQRRLRDIGDAW---
83647568        YPLFKRLLTAMLHKKPEKRLDSMGKVLQWL
                   . *::   * *..  *     .
```

Cluster No. 42 multiple sequence alignment

```
CLUSTAL 2.0.10 multiple sequence alignment


9945898         FDIERELGEGAMATVYLATQRSLQRKVALKVMAAALAADPSFAERFLREGRTLARLSHPN
90022079        YKILRTLGKGGMATVYLAEQEIFEREVALKVMSKSLAEDPSFGQRFMREAKIVSQLVHPN
51246425        YCIIEKIGVGGMADVYKAEDTELGRQVALKLLPPEFARDAERVTRFDKEVCAAAALMHQN
83647361        YEILDEVAEGGMAKVFKARQTLADRIVAIKIISPAICLNKEFRQRFIQEAQITAKLNHPN
                : *   :. *.** *: * :    * **:*::.  :. : .   ** :*    : * * *

9945898         TVTIHDIGNVGSCYYMAMEYLPNGTLKERIQQG-LDPEQGLAYVRQVAAALGYAHSQGLV
90022079        IVTVHDVGVHEGYYYLSMQVIDGLDLKQ-SRRS-LSLRQKVTAIRDIAKALDYAGSKGYV
51246425        IVTLYDVTHVDGYHFYTMDLITGGDLKEKIKQG-LSPEAAVNILLELGKALSHAHAKEFV
83647361        IIQIYDIAPFEDTFFISMEFMEGGSLTERIRSGRLTRDESTRIILQLAEAMNYAHKLGYI
                 : ::*:    . .: :*: : .  *.:  : . *        : ::. *:.:*     :

9945898         HRDVKPANILFR-ADGTAVLSDFGIAKSIEDNTQFTQVGFAVGTPSYMSPEQARGQEIDG
90022079        HRDIKPENILFHTSDGRAVLTDFGIARAAESDLTMTQTGTAIGTPHYMSPEQAKGLAVDH
51246425        HRDIKPENVLFR-EDGTAVLTDFGIAKAVGTATQMTKTGMSIGTPHYMSPEQARGIGVGY
83647361        HRDIKPGNILFR-ADDTPVLSDYGIAKSLYHRGDLTTTGSILGSPPYMSPEQAMGRQLDH
                ***:** *:**:  *. .**:*:***::      :* .*  :*:* ******* *  :.

9945898         RADLYALGVVLYEILTGKLPYNGKDSLSTALAHLTEPLPELPIEQGRYQDILRQLLAKDP
90022079        RADLYSLGVVFYLLLANRLPFDAESAVAIGIKHITQAIPLLPAEYNQLQPLIDKLMAKKV
51246425        RSDLYSLGILFYEMLVGSVPYDSEDTIAIAYSHVNDPVPELPSQFRKYQPIVDHLLAKSS
83647361        RADWYSLGIVFYQMLTGAVPFEATDPISLGIKHKQEPPPPLPSHLRMYQQLMDLLLAKEP
                *:* *:**:::* :*.. :*::. ..:: .  *  :. * ** .    * ::  *:**.

9945898         AERFPTAGALIAAL
90022079        EDRFQSAKELIDAL
51246425        ADRYVDAATMVRAI
83647361        ERRLSSLDEFRAHL
                  *      :   :
```

Cluster No. 43 multiple sequence alignment

```
CLUSTAL 2.0.10 multiple sequence alignment


86739661        YTLLGKLGQGGMGMVYLGRSGKGRLVAIKVIRNEAVGNPEFRARFRLEAETARRVARVCT
86742078        YRLRGRLGVGGMGVVYLAEDPHHHPVAVKVIRVEFAADPEFRARFRHEAEAARRVPRFCT
86741942        YQLVARLGQGGMGIVYLGRSRDGRPVAVKVVRTDLARQPEFLARFRREAEVAQRVARFCT
86742102        YRLVRRLGAGGMGTVYLGENAAGGLVAVKLIRADLARLAEFRSRLKQEADNARRVARFCT
86743100        YVLLSRLGVGGMGTVYLARNRAGRRVAIKVIRPDLAADEEFRRRFRAEVEAARKVAPFCT
86742726        YTVERKLVDARTGPVFLARNGEARPVLVKTITAPFGRDAEFRRRLRVDLDNIRRLAPSCL
                * :  :*  .  * *:*...     * :* :        **  *:: : :  :::.  *

86739661        AEVLDADPDAEWPYLVTEFIEGETLARYVQRNGPLADANLEQLAVGVAAALTAIHSAGIV
86742078        AAFLDADPNAERPYLVTDYVPGPTLAQAARR--PLRGAELEQVAVHIAVALTVIHGAGVV
86741942        AEVLEVDVEADRPYLVTEFIDGPTLADAIAANGPMAEADLERLAISVAAALTAIHAAGMI
86742102        AAVLDVDITADPPYLVTEYVDGPTLSEAVGTRGPLTPAELHQLAVSMTTALMAIHRAGLV
86743100        AEVLDADPNAPAPYLVTEFIDGVRLDMQVDK-GPLTSSTLTGLAVGVATALTAIHSAGLV
86742726        AAILDLDTGARPPYVVAEFIDAPTLAATVAGGAALSGPDTYRLAVGLATALAALHELEIF
                * .*: *  *  **:*:::: .  *        .:  .    :*: ::.** .:*   :.

86739661        HRDLKPANVILSPFGPRVIDFGIARAVDAGSNLT--GDLQQLGTPAFMSPEQIESRPITS
86742078        HRDLKPSNVILSPTGARVIDFGIARAH--GMTMF--HIDEQIGTPAYTAPENIDGAPPDP
86741942        HRDLKPSNVLLSRLGPRVIDFGIARAMDSTTSLT--TSTGLIGTPAFMAPEQARGALVTA
86742102        HRDLKPSNILLSRLGPKVIDFGIARALDSATVLS--GDR-QLGTPAFMAPEQALGEQVTS
86743100        HRDLKPSNVMLSLSGPRVIDFGIAQALEGAKAK---PTAWGFGSAGWMAPEQVHGQPIGP
86742726        LGDLKPINVVLSGQGVRLVDFGLFRAMNAVSINNPGGPPSGIGTLAFITPEQALGQTATV
                  **** *::**  * :::***: :*               :*: .: :**:  .

86739661        AVDIWAWGGLVAFAATGHYPFGEASAQVLLYRALHEEP-QLDDVDPALRPIVWHAMRKDP
86742078        AADIFAWGGVVLYAATGQPPFGDRSSELLLHRIRYDHPTHLNQLHGRLHDTVTAAMAKAP
86741942        AADIFAWGGVVTFAGTGIGPFGKATTPVLLYRAVHEAP-KIDGLPDGLRSIVAKTMSKEP
86742102        AADVFAWGGVLIFAGTGRYPFGNGPAPSVLYRTVNDPP-TLDGFEDSLRPLVSDAMRKAA
86743100        EADVFAWGILIAYAGTGRHPFGDGTDIDLGMRIVGSAP-DLRGLPQPLVGLVSAALAKHP
86742726        ASDVFTWGGMLLFAATGRPPFGAGTPRVLLQRAVYAEP-DLSVFCPELRELVAAAMRKDP
                  *:::** :: :*.**  ***  .   :  *     *  :  .   *   *  :: * .

86739661        STRPSAQQLMLRL
86742078        EQRPTAEQLYRML
86741942        ADRPTASDLYQSL
86742102        AERPTAEKLYARL
86743100        DDRPSARDLLLRL
86742726        KRRPAAAELLEQL
                  **:* .*   *
```

Cluster No. 44 multiple sequence alignment

```
CLUSTAL 2.0.10 multiple sequence alignment


24983617        --------HSRQSLLYKVRDSQNQPWLLKTLPTAREQEPGAAQGLLLEEWFLRRIAGRHF
26988816        --------HSRQSLLYKVRDSQNQPWLLKTLPTAREQEPGAAQGLLLEEWFLRRIAGRHF
104780903       WKVDALLGQSRQSQLYRVRDEQGQAWLLKTLPQVDEVEPQAIQSLLLEEWFLRRVAGRHF
28852745        WNVGDIVAQSRQSILYRVKDAHGQPWLLKTLPTSRHDEAGAGQGLLLEEWFLRRVAGRFF
28869503        WNVGDIVAQSRQSILYRVKDAHGQPWLLKTLPTSRHDEAGAGQGLLLEEWFLRRVAGRFF
9947763         WRVRQLLGESRQSLLYRVEDGQGQPWLLKTLPAARADDPLATQALLLEEWFLRRVQGRHF
119897580       LTVEEVLHHSLVTLLYRVADAQGRQRVLKTLRNG-AGDVENCQALVYEEWLARRVVDSRF
                        .*  : **:* * :.:  :****      :    *.*: ***: **: .  *

24983617        PELHAASQRQHLYYVMREYPGQNLAALLAEQGPLPLPQWLEVARQLLQAVGVLHRRNLLH
26988816        PELHAASQRQHLYYVMREYPGQNLAALLAEQGPLPLPQWLEVARQLLQAVGVLHRRNLLH
104780903       PETHPANQRQHLYYLMREYPGETLATLFATHGPLPLPQWLEIARQLLHAVGALHRRNLLH
28852745        PEVHSLAQRQHLYYVMREYPGSTLAEVFRRNGPLPLVQWQDLATRVLRATGLLHRRNIIH
28869503        PEVHSLAQRQHLYYVMREYPGSTLAEVFRRNGPLPLVQWQDLATRVLRATGLLHRRNIIH
9947763         PELHGLAQRQHLYYLMREHPGETLAERLRNHGPLSLPEWLGLANQLLRGLGQLHRRNLLH
119897580       PEVIDWPGRHHLYYLMSWHEGATLKARLAAGHRFSPAQVVELGGEILRGVGALHRLAIVH
                **      *:****:*  : * .*   :     :.  :   :. .:*:. * ***  ::*

24983617        RDIKPDNLHLGRDGQLRLLDFGLAYCPGLSEDPLHELPGTPSYIAPEAFDGHPPSPRQDL
26988816        RDIKPDNLHLGRDGQLRLLDFGLAYCPGLSEDPLHELPGTPSYIAPEAFDGHPPSPRQDL
104780903       RDIKPENLHLGSDGQLRVLDFGLAYCPGLSEDLPHTLPGTPSYIAPEAFDGQPPSPRHDL
28852745        RDIKPENLLLGNDGELRLLDFGLAFCPGLSATNAEDLPGTPSYIAPEAFNGAEAHPRQDL
28869503        RDIKPENLLLGNDGELRLLDFGLAFCPGLSATNAEDLPGTPSYIAPEAFNGAEAHPRQDL
9947763         RDLKPENLHLGRDGELRLLDFGLAWCPGLSREDPHLLPGTPSYLAPECFSGTSPSVRQDL
119897580       RDIKPDNLHLDAHGRLRILDLGVAASDGHAFNEINN-PGTPSYMAPELFAGERANEASDL
                **:**:** *. .*.**:**:*:* . * :    .  ******:*** * *  .    **

24983617        YAVGVTLYHLLTGHYPYGEVEAFQRPRFGQPVNAARYRPDLPEWLQHNLLQAVASDPAQR
26988816        YAVGVTLYHLLTGHYPYGEVEAFQRPRFGQPVNAARYRPDLPEWLQHNLLQAVASDPAQR
104780903       YAVGVTLYHLLTGHYPYGEIEAFQRPRFGAPVSAARYRPDLPEWLQRNLEQAVAANPAHR
28852745        YAVGVTLYYLLTGHYPYGEIEAFQHRRFGTPIPASRYRPDLPQWLSQSLDKALQADPDQR
28869503        YAVGVTLYYLLTGHYPYGEIEAFQHRRFGTPIPASRYRPDLPQWLSQSLDKALQADPDQR
9947763         YATGVCLYQALTGRYPYGEIEAFQHPRFGRPVPPSRYRPDLPAWIDDLLLRAVACDPAQR
119897580       YAVGVTLYELLARRYPYGEIEPFQKPRFGDPVAVTRYRPDTPDWLEAILLKAVAPTPAER
                **.** **  *: :*****:*.**: *** *:  :***** * *:.  * :*:   * .*

24983617        FETAEHWL---
26988816        FETAEHWL---
104780903       FETAEQWLLLL
28852745        YETPEQWLLEL
28869503        YETPEQWLLEL
9947763         FETAEEWLLSL
119897580       FETAEEFLLAL
                :**.*.:*
```

Cluster No. 45 multiple sequence alignment

```
CLUSTAL 2.0.10 multiple sequence alignment


118477730       YKIESVIGMGSYGVTYVVNDLQINRYKVLKQLRQSKQRYVSGRKSFEQEKMILQTLNHHA
30256946        YKIESVIGMGSYGVTYVVNDLQINRYKVLKQLRQSKQRYVSGRKSFEQEKMILQTLNHHA
29895941        YKIESVIGMGSYGVTYVVNDLQINRYKVLKQLRQSKQRYESGRKSFEQEKMILQTLNHHA
30020390        YKIESVIGMGSYGVTYVVNDLQINRYKVLKQLRQSKQRYESGRKSFEQEKMILQTLNHHA
138895192       YEIIEELGMGSYGIAYKGRDRDTGRLVVIKQARRTKG--EDGRRLLQREADVLSRLRHPQ
52784213        YRINRVLGMGNYGITYLAEELGRSGFRVIKQQRKTKAWTSAGRRSFNREAEILRELDLPA
                *.*   :***.**::*  .:   .   *:** *::*     **: :::*  :*  *

118477730       IPSLYDHFVWEKKSFFVMEYMPGKNFEDYIFLDGHVYTEREVLKILYEILEIVSVFHSKG
30256946        IPSLYDHFVWEKKSFFVMEYMPGKNFEDYIFLDGHVYTEREVLKILYEILEIVSVFHSKD
29895941        IPSLYDHFLWEKKSFFVMEYMPGKNFEDYIFIDGYVYTEREVFEILYEILEIVSVFHSEG
30020390        IPSLYDHFLWEKKSFFVMEYMPGKNFEDYIFIDGYVYTEREVFEILYEILEIVSVFHSEG
138895192       IPARYDTFIEGGQPHLVMDYIDGQTVEDQIFGLGITYTEQAAFRLLLDVLDVVRYIHASG
52784213        APRLYEVLRAAGERFIVMEYIEGKTFEDLLFYDGRVFDEQQTISILKEVLHSVSLLHSRG
                 *  *: :    : .:**:*: *:..** :*  * .: *: .: :* ::*. *  :*: .

118477730       IIHRDLRIPNILMKENQISIIDFGLAKLKGEGDERATTYEGEQALMREVHFRSDFYALGH
30256946        IIHRDLRIPNILMKENQISIIDFGLAKWKGEGDERATTYEGEQALMREVHFRSDFYALGH
29895941        IIHRDLRIPNILMKENQISIIDFGLAKWKGEDDERATTYEGEQALMREVHFRSDFYALGH
30020390        IIHRDLRIPNILMKENQISIIDFGLAKWKGEDDERATTYEGEQALMREVHFRSDFYALGH
138895192       IVHRDLRIPNIIWRHGTVAIIDFGLACRMGERVDLRDDDPLEKRLRREPHPRSDFYALGH
52784213        IIHRDLRIPNLIVNRGTIRIIDFGLACRLTERERVDNRHP-EKKLMRAVSVKSDFYALGH
                *:********:: ... : *******    *          *: * *    :********

118477730       FSLFLLYAGYESNEKYEKPWYDELTLENYNREMLMRMLQMKTPYYENVRDLKKDVAFALE
30256946        FSLFLLYAGYESNEKYEKPWYDELTLENYNREMLMRMLQMKTPYYENVRDLKKDVAFALE
29895941        FSLFLLYAGYESNEKQEKPWYEELTLEHYNREMLMRMLQIKTPYYENVQDLKQDVASALE
30020390        FSLFLLYAGYESNEKQEKPWYEELTLEHYNREMLMRMLQIKTPYYENVQDLKQDVASALE
138895192       FTLFLLYSAYEPTSEDEKSWEEELDLSPKARAMLRKMLQLDAP-YDHVDELIADVKQLL-
52784213        FALFLLYSGFTPSDEEEKCWEEELSISSGLKSVLRKMLQIDLP-YERAEDIIGDL-----
                *:*****:.: ...: ** * :** :.   : :* :***:. * *:.. ::  *:

118477730       RMEVPCFKSF
30256946        RMEVPCFKSF
29895941        RMETPCF---
30020390        RMETPCF---
138895192       ----------
52784213        ----------
```

Cluster No. 46 multiple sequence alignment

```
CLUSTAL 2.0.10 multiple sequence alignment


120401718       YEIKGCIAHGGLGWVYLAFDKNVNDRPVVLKGLVHSGDAEAQAIAMAERQFLAQVTHPGI
126433142       YEIKGCIAHGGLGWVYLAFDHNVNERPVVLKGLVHSGDAEAQAIAMAERQFLAEVTHPGI
1817676         YEVKGCIAHGGLGWIYLALDRNVNGRPVVLKGLVHSGDAEAQAMAMAERQFLAEVVHPSI
31791588        YEVKGCIAHGGLGWIYLALDRNVNGRPVVLKGLVHSGDAEAQAMAMAERQFLAEVVHPSI
41409991        YEVKGCIAHGGLGWVYLAVDHNVNDRPVVLKGLVHSGDAEAQAIAMAERQFLAEVVHPQI
19553944        YEVLGVIAHGGMGWIYLANDRNVSGRIVVLKGMMAQSSVQDQGTAEAEREFLADITHPGI
23494432        YEVLGVIAHGGMGWIYLANDLNVANRVVVLKGMMAQTSAQDQGTAEAERAFLADITHPGI
38234610        YEVAGVIAHGGMGWIYLAHDRNVSGRMVVLKGLRDKAKPQDYGAAVAEKEFLADITHPGI
68535317        YEILGPIAHGGLGWVYIAIDHNVADRYVVLKGMMATENEQERAVAESERAFLADITHPGI
                **: * *****:**:*:* * **  * *****:    . :  . * :*: ***::.** *

120401718       VKIYNFVEHEDKHGNPVGYIVMEYVGGTSLK-QATAQKGTRLPVAEAIGFMLEILPALGH
126433142       VKIYNFVEHEDKHGNPVGYIVMEYVGGTSLK-QARGQ---RLPVAQAIGYMLEILPALGY
1817676         VQIFNFVEHTDRHGDPVGYIVMEYVGGQSLK-RSKGQ---KLPVAEAIAYLLEILPALSY
31791588        VQIFNFVEHTDRHGDPVGYIVMEYVGGQSLK-RSKGQ---KLPVAEAIAYLLEILPALSY
41409991        VQIFNFVEHVDRHGNPVGYIVMEYVGGQPLR-HGKGE---KLPVSEAIAYVLEILPALGY
19553944        VKAYNFIDDPRVPG---GFIVMEYVNGPSLKDRCKAQPDGVLRVDLAIGYILELLPAMDY
23494432        VKAYNFIDDPRVPG---GFIVMEYVNGPSLNDRRKQQKDGVLSFDLAIGYILEVLPAMDY
38234610        VKSYNFIDD---------LIVMEYVEGPALR--------GPMAIDLAIGYILEVLPALDY
68535317        VKIFNFIDDPRSPG---GFIVMEYVGGPSLRGQRRKLAAGVLDLDVAIGYILEILPALDY
                *: :**::.          ****** * .*.          : .  **.::**:***:.:

120401718       LHSIGLVYNDLKPENVMVTEDQLKLIDLGAVSRINSFGYLYGTPGYQAPEIVRTGPTVAT
126433142       LHSLGLTYNDLKPENIMLTEDQVKLIDLGAVSTINSFGYLYGTPGYQAPEIVRTGPTVQS
1817676         LHSIGLVYNDLKPENIMLTEEQLKLIDLGAVSRINSFGYLYGTPGFQAPEIVRTGPTVAT
31791588        LHSIGLVYNDLKPENIMLTEEQLKLIDLGAVSRINSFGYLYGTPGFQAPEIVRTGPTVAT
41409991        LHSIGLVYNDLKPENIMLTEEQLKLIDLGAVSRINSFGYLYGTPGFQAPEIVRTGPTVAT
19553944        LHQRGVVYNDLKPENVIATEDQVKLIDLGAVTGIGAFGYIYGTKGFQAPEVATHGPSISS
23494432        LHSRGVVYNDLKPENIIATEDQVKLIDLGAVSGIGAYGYIYGTKGYQAPEVATQGPSVAS
38234610        LHSRGVVYNDLKPDNIIISEDQVKLIDLGAVSGIGAFGYIYGTKGYQAPEVASDGPSIAS
68535317        LHSRGVVYNDLKPDNILITEDQVKLIDLGAVTGIGAFGHIFGTKGFQAPEIAKTGPTVAS
                **. *:.******:*:: :*:*:********: *.::*:::** *:****:.  **:: :

120401718       DMYTVGRTLAALTLKLRTRKGRYVDGLPEDD--PVLATYDSYGRLLRRAIDPDPRRRFQS
126433142       DIYTVGRTLAALTLELRTRKGRYVEGLPEDD--PVLAQYDSFGRLLRRAIDPDPRRRFAS
1817676         DIYTVGRTLAALTLDLPTRNGRYVDGLPEDD--PVLKTYDSYGRLLRRAIDPDPRQRFTT
31791588        DIYTVGRTLAALTLDLPTRNGRYVDGLPEDD--PVLKTYDSYGRLLRRAIDPDPRQRFTT
41409991        DIYTVGRTLAALTLNLPTRNGRYVDGIPDND--PVLGTYDSFRRLLRRATDPDPRRRFSS
19553944        DIFTIGRTLAALTMPLPVEDGVLAPGIPSPKNSPLLRRHLSFYRLLQRATADDPQHRFRN
23494432        DIYTIGRTLAALTLKLPVEDGVLAPGIPSPNDQPQLRRYLSFYRLLLRATAEKPEDRFTS
38234610        DIYTIGRTLADLTIDLNDGAGGKKD-LPTADEEPLFAQNLSFYRLVRRCTRSDPAKRFSS
68535317        DIYTVGRTLAALIVKLPVEDGVYAPGLPTPDEEPLFREYLSLYRLLLRATDEDPEARFSS
                *::*:***** * : *    *     :*  .  * :    *  **: *.   .*  ** .

120401718       AEEMSSQLM-
126433142       SEEMSSQLL-
1817676         AEEMSAQL--
31791588        AEEMSAQL--
41409991        TEEMSAQLM-
19553944        VSELRTQLYG
23494432        VAELRTQLFG
38234610        VRELETQLYG
68535317        ATAMANQLI-
                   :  **
```

Cluster No. 47 multiple sequence alignment

```
CLUSTAL 2.0.10 multiple sequence alignment


108760330       LELLELLGRGGMGEVWLARQQSLGRTVAVKLLPPRLAKDPEFVTRFEKEATALAALNHPH
32475155        LQITELLGAGGMGAVYKARQEGLDRVVALKILPEEFGHDVKFALRFTREARTLAKLNHPN
116620001       YEILAPIGAGGMGAVYKALDTRLNREVAIKTV------SEQHMLRFDREARAIAALNHPH
116621582       YRIETLIGSGGMGSVYRAVDTRLDRAVAIKIP------AQPFDARFEREGRSIAALNHPN
                 .:   :* **** *: * :  *.* **:*           .  ** :*. ::* ****:

108760330       IIQIIDRGVAGEHYYFVMEYVEGRSLREAMSAG-LPPEKGIKLLLAVARAIECAHDKGII
32475155        IVSVYEFGHVDDTYYFLMEYVDGSTLRDVVAAGQLAPAHALAIVPHLCDALQYAHDNGVI
116620001       ICALYDVGDS----YLVMEYVEGSPL-----KGPVPVPEAVRLATQIVEALAAAHAKGII
116621582       ICTVHDVGPN----YLVMELIEGPTLAERIRKGPVPLEEALAIARQIAAALDTAHQRGLI
                *  : : *      *::** ::* .*      * :.  ..: :   :  *:  ** .*:*

108760330       HRDLKPENILLDGRGHVKVADFGLAG-------IRAP-DSRLQLTATSVAMGTLNYMAPE
32475155        HRDIKPENILMAVDGSVKIADFGLSR-------ILGDQDQPSALTGTHQIMGTPRYMAPE
116620001       HRDLKPGNVLLTASG-VKLLDFGLAKFAEQ-PQLDADST--LTQTGIGTVLGTASYMSPE
116621582       HRDLKPANVKVKPDGTVKVLDFGLARFAITDPPGDDDPTHSLAVTQAGAILGTPHYMAPE
                ***:** *: :   * **: ****:                   *     :**  **:**

108760330       QRRDAKNVDGRADLFSLGIILYEVLTGELPLGRFKLP---SSKVPGLDPRVDP------V
32475155        QLEGARGVDHRADIYSLGVVFYEMLTGELPIGRFAVP---SKKVQ-IDVRLDD------V
116620001       QAQ-GRPADSRSDVFSFGAVLYELLCGKQAFHEDTAIGTIAAVVHKEPAPLQAPPDLTRI
116621582       QAL-GKSADKRADIWAFGVILYEMVTGARPFPGD------VPVWDRVPAAIRP------L
                *   .: .* *:*::::* ::**:: *  .:                   :        :

108760330       VDRLLEQDPAARYEKASELCAAL
32475155        VLRTLEKEPQRRYQRASQIKSDV
116620001       ISRCLSKAPCDRFQTMSDL----
116621582       LTRCLQKDPAHRLRDIGDAPFL-
                : * *.: *  * .  .:
```

Cluster No. 48 multiple sequence alignment

CLUSTAL 2.0.10 multiple sequence alignment

```
4377006         YHVKKILSKKLRSRVVHGLHPETRHSTVIKVFSPSPSFTSRSVYNFLKEAQSLHQITHPN
8163351         YHVKKILSKKLRSRVVHGLHPETRHSTVIKVFSPSPSFTSRSVYNFLKEAQSLHQITHPN
8979075         YHVKKILSKKLRSRVVHGLHPETRHSTVIKVFSPSPSFTSRSVYNFLKEAQSLHQITHPN
33242061        YHVKKILSKKLRSRVVHGLHPETRHSTVIKVFSPSPSFTSRSVYNFLKEAQSLHQITHPN
29834154        YHIKKLLSKKEGSTVYQGVHPDTLQPAAIKVLATPLVTDTPRVHNFLKEARIIEQISHPN
29839807        YHIKKLLSKKEGSTVYQGVHPDTLQPAAIKVLATPLVTDTPRVHNFLKEARIIEQISHPN
89898773        YCIKRIMSKKEGSTVYHGVHPVTLQPTVIKVLVTPLVADTLRVHNFLKEARIIEQISHPN
3329124         ----RELSRKVGLTVYQGVDEHSSRPVVIKTLVSPGIHDRRFLRAFEEEARIMQLVTHPA
                    : :*:*    * :*:.  : :...**.: ..       :  * :**: :. ::**

4377006         IVKFHRYGKWQDCLYIAMEYIEGISLREYILAQFISLPQAIDIIFDIAQALEHLHSRNIL
8163351         IVKFHRYGKWQDCLYIAMEYIEGISLREYILAQFISLPQAIDIIFDIAQALEHLHSRNIL
8979075         IVKFHRYGKWQDCLYIAMEYIEGISLREYILAQFISLPQAIDIIFDIAQALEHLHSRNIL
33242061        IVKFHRYGKWQDCLYIAMEYIEGISLREYILAQFISLPQAIDIIFDIAQALEHLHSRNIL
29834154        IVKLYQYGQCREGLYIAMEYIQGVSLRHYILSQLIPLSRAIDIILHIAQAIEYLHSRGIL
29839807        IVKLYQYGQCREGLYIAMEYIQGVSLRHYILSQLIPLSRAIDIILHIAQAIEYLHSRGIL
89898773        IVKLYQYGQCREGLYMAMEHIQGVSLRKYILSQLIPLSRAVDIILQIAQALEYLHSRGIL
3329124         FVRLEDRGECEQGRYLVSEYILGSSLRDSILSSQISLDKAISIVLQVAQAITTLHRHGVL
                :*::    *: .:  *:. *:* * ***. **:. *.* :*:.*::.:***:  ** :.:*

4377006         HKDIKPENILITPQGKIKLIDFGLADWDTEIQRAHPSVIGTPYYMSPEQRQGESHSPASD
8163351         HKDIKPENILITPQGKIKLIDFGLADWDTEIQRAHPSVIGTPYYMSPEQRQGESHSPASD
8979075         HKDIKPENILITPQGKIKLIDFGLADWDTEIQRAHPSVIGTPYYMSPEQRQGESHSPASD
33242061        HKDIKPENILITPQGKIKLIDFGLADWDTEIQRAHPSVIGTPYYMSPEQRQGESHSPASD
29834154        HRDIKPENILITSQGNIKLIDFGLAISS-IDKDARPLYLGTPSYMSPEQRQGDKISELSE
29839807        HRDIKPENILITSQGNIKLIDFGLAISS-IDKDARPLYLGTPSYMSPEQRQGDKISELSE
89898773        HRDIKPENILITPQGEIKLIDFGLAASSSMANDPYPVCLGTPSYMSPEQRQGDKISENSE
3329124         HLDIKPENIVLSQSGEIKLIDYGLSAWQ-------FNHWGSPAYMSPEQSRQEPPSPASD
                * *******::: .*:*****:**:  .           *:* ****** : :  *  *:

4377006         IYALGLLAYELILGHLSLGRVFLSLVPERISKILAKALQPSPNNRYSSTREFIQDI----
8163351         IYALGLLAYELILGHLSLGRVFLSLVPERISKILAKALQPSPNNRYSSTREFIQDI----
8979075         IYALGLLAYELILGHLSLGRVFLSLVPERISKILAKALQPSPNNRYSSTREFIQDI----
33242061        IYALGLLAYELILGHLSLGRVFLSLVPERISKILAKALQPSPNNRYSSTREFIQDI----
29834154        IYSLGLIAYELILGNLALGKVVLSLIPDRVSKILAKALQPSPTDRYASMKEFI-------
29839807        IYSLGLIAYELILGNLALGKVVLSLIPDRVSKILAKALQPSPTDRYASMKEFI-------
89898773        IYSLGLIAYELILGNLALGKVILSLVPERMSKILAKALQPSPKDRYASMKEFLIDL----
3329124         VYSLALLAYELIMGQLALGKVYVSLLPSKISKILIQALQPSPAARFSSMQEFAEALQDYL
                :*:*.*:*****:*:*:**:* :**:*.::**** :******  *::* :**
```

Cluster No. 49 multiple sequence alignment

```
CLUSTAL 2.0.10 multiple sequence alignment


4376355         -----------MGEVYLAYDPVCSRKVALKKIREDLAENPLLKRRFLREARIAADLIHPG
8978468         -----------MGEVYLAYDPVCSRKVALKKIREDLAENPLLKRRFLREARIAADLIHPG
8163479         YDIVRIIGKGGMGEVYLAYDPVCSRKVALKKIREDLAENPLLKRRFLREARIAADLIHPG
33241430        YDIVRIIGKGGMGEVYLAYDPVCSRKVALKKIREDLAENPLLKRRFLREARIAADLIHPG
29834784        YDIIRMIGKGGMGEVYLAYDPVCSRKVALKRIREDLSDNELLKKRFLREAKIAADLVHPG
29840435        YDIIRMIGKGGMGEVYLAYDPVCSRKVALKRIREDLSDNELLKKRFLREAKIAADLVHPG
89898141        ------IGKGGMGEVYLAYDPVCSRKVALKRIREDLSDNELLKKRFLREAKIAADLVHPG
3328716         YELIRLIGKGGMGEVYLAHDKACSRRVALKRIREDLSGNALLRQRFLREAKIAADLIHPG
                           *******:* .***:****:*****: * **::******:*****:***

4376355         VVPVYTIYSEKDPVYYTMPYIEGYTLKTLLKSVWQKESLSKELAEKTSVGAFLSIFHKIC
8978468         VVPVYTIYSEKDPVYYTMPYIEGYTLKTLLKSVWQKESLSKELAEKTSVGAFLSIFHKIC
8163479         VVPVYTIYSEKDPVYYTMPYIEGYTLKTLLKSVWQKESLSKELAEKTSVGAFLSIFHKIC
33241430        VVPVYTIYSEKDPVYYTMPYIEGYTLKTLLKSVWQKESLSKELAEKTSVGAFLSIFHKIC
29834784        VVPVFTICSDSDPVYYTMPYIEGYTLKSLLKSVWQCDSLPKDLAEQTSVATFLSIFHKIC
29840435        VVPVFTICSDSDPVYYTMPYIEGYTLKSLLKSVWQCDSLPKDLAEQTSVATFLSIFHKIC
89898141        VVPVFTICSDSDPVYYTMPYIEGYTLKSLLKSVWQCDSLPKDLAEQTSVGTFLSIFHKIC
3328716         IVPVYSICSDGEAVYYTMPYIEGFSLKSLLKSVWQKEVLSKELEEKTSVKSFLPIFDKIC
                :***::* *: :.**********::**:******* : *.*:* *:*** :**.**.***

4376355         CTIEYVHSRGILHRDLKPDNILLGLFSEAVILDWGAAVACGE-----EED-LLDIDVSKE
8978468         CTIEYVHSRGILHRDLKPDNILLGLFSEAVILDWGAAVACGE-----EED-LLDIDVSKE
8163479         CTIEYVHSRGILHRDLKPDNILLGLFSEAVILDWGAAVACGE-----EED-LLDIDVSKE
33241430        CTIEYVHSRGILHRDLKPDNILLGLFSEAVILDWGAAVACGE-----EED-LLDIDVSKE
29834784        STVEYVHSRGILHRDLKPDNILLGLFSEVVILDWGAALSKEM-----EEDFLSDIDVRIP
29840435        STVEYVHSRGILHRDLKPDNILLGLFSEVVILDWGAALSKEM-----EEDFLSDIDVRIP
89898141        STVEYVHSRGILHRDLKPDNILLGLFSEVVILDWGAALSKEM-----QEEVLLDIDIPVT
3328716         ATVEYIHSKGVLHRDLKPDNILLGLFGEVVIIDWGAAIFKHAKELKLEQDDEAAVSFDER
                .*:**:**:*:**************.*.**:*****:          :::    :..

4376355         EVLSSRMTIPGRIVGTPDYMAPERLLGHPASKSTDIYALGVVLYQMLTLSFPYRRKKGKK
8978468         EVLSSRMTIPGRIVGTPDYMAPERLLGHPASKSTDIYALGVVLYQMLTLSFPYRRKKGKK
8163479         EVLSSRMTIPGRIVGTPDYMAPERLLGHPASKSTDIYALGVVLYQMLTLSFPYRRKKGKK
33241430        EVLSSRMTIPGRIVGTPDYMAPERLLGHPASKSTDIYALGVVLYQMLTLSFPYRRKKGKK
29834784        GSLFSNMTIPGKIVGTPDYMAPERLRGTPASESTDIYALGVILYQMLTLSFPYRKKKGQK
29840435        GSLFSNMTIPGKIVGTPDYMAPERLRGTPASESTDIYALGVILYQMLTLSFPYRKKKGQK
89898141        GSMFSNMTIPGKIVGTPDYMAPERLRGTPASESTDIYALGVILYQMLTLSFPYRNKKGKK
3328716         NICYSSMTIPGKIVGTPDYMAPESLLGVEASEKTDIYALGLILYQMLTLAFPYRRKKGRK
                    * *****:*********** * *  **:.*******::*******:****.***:*

4376355         IVLDGQRIPSPQEVAPYREIPPFLSAVVMRMLAVDPQERYSSVTELKEDI
8978468         IVLDGQRIPSPQEVAPYREIPPFLSAVVMRMLAVDPQERYSSVTELKEDI
8163479         IVLDGQRIPSPQEVAPYREIPPFLSAVVMRMLAVDPQERYSSVTELKEDI
33241430        IVLDGQRIPSPQEVAPYREIPPFLSAVVMRMLAVDPQERYSSVTELKEDI
29834784        ISLR-HQISFPEEIAPHREIPPFLSQVVMRALAADPRERYRSVSALKADI
29840435        ISLR-HQISFPEEIAPHREIPPFLSQVVMRALAADPRERYRSVSALKADI
89898141        ISFR-HQISSPEEIAPHREIPPFLSQVAMKALAADPKVRYASVKELKDDI
3328716         LSYR-DVVLPPIEMSPYREIPPSLSQIAMKAIAINPADRFSSIQELRQAL
                :    . :  * *::*:***** ** :.*: :* :*  *: *:  *:  :
```

Cluster No. 50 multiple sequence alignment

```
CLUSTAL 2.0.10 multiple sequence alignment


37520154        YRVVRELGRGGMGVVFLAERADGQFQKQVCIKVLQTGWAAALQVGRFLSERQILANLEHP
37523641        YRLVGELGRGGMGVVYLAERADGLFSKRVAIKVLQPGRGAPLLLERFVQERQILANLEHP
37520484        YRIIRPVGQGGMGAVYLAERDDGQFDKRAAVKILQPQLHGPGLRERFIGERQILASLDHP
116620747       YTIQALIGRGGMGAVYRAVREDD-FHLEVAIKLLKRGTDTDAALARFRGERRILAALQHS
116626655       YTIQALIGRGGMGAVYRAVREDD-FHLEVAIKLLKRGTDTDAALARFRGERRILAALQHS
116626939       YSIAGLIGRGGMGTVYRAEREDD-FHTQVALKVLKRGTDTEKALSRFRSERQILAGLQHP
94971075        YRLVKEIGRGGMGSVFLAERDDEHFHQTVAIKIVKRGMDSAEVLARFRHERQILAGLEHP
116622939       YRLVRTIGAGGMGTVFEAVREQD-YHKRVALKVAASAIGTPAWVERFKQERQILSGLDHP
116620251       YRVLRQIGRGGMGVVYLGARADGEYQKQVAIKLITSGWRDAGLERRFRRERQILAQLDHP
85375834        YRLEGLIGSGGMGSVYAARRDRGDFEHEVAIKLIKPGLLSEQLTERFGRERQLLASFSHP
21244844        WAIDRLIGAGGMGQVYLGHRADGAYEREVAIKLVAADALDAQGRALFEFECRLLAQMVHP
                : :   :* **** *: . *    :   ..:*:             *  * ::*: : *.

37520154        YIARLIDGGSTEAGVPYLVMEFVDGMPIDRYCEAQQLGLRPRLELFSKVCQAVQYAHTCR
37523641        HIARLIDGGTSEEGLPYLVMEYIDGEPIDCYCRKQQLPVRERLALIEKVCRAVHHAHTLQ
37520484        YITRLLDGGTTEQGLPYLVMDYVEGQPINLYCNERKLDVDERLRLFLKVCEAVHYAHAHR
116620747       NIARLLDGGATEFGLPYLVMEYVHGTPLLEYAAT--LDTRRRLELFRSVCSAVQYAHQNL
116626655       NIARLLDGGATEFGLPYLVMEYVHGTPLLEYAAT--LDTRRRLELFRSVCSAVQYAHQNL
116626939       NIARLLDGGATGAGLPYFVMEYVEGAPLLEYAAP--LPVRERVKLFRSLCAAVQYAHEKR
94971075        YIARLIDGGTTDDGRPFFVMERVEGRPIDVYCREQNLSVEARLRLFVRVCEAISYAHRAL
116622939       NIARFLDGGASSDGLPYFAMELVEGEPITDFVIDRNCGLRERIELFRKVCAAVSFAHQSL
116620251       GIARLLDGGSTADGQPYFVMEYIEGLGLLEYCARHELDIKQRLTLFLAVCDAVGYAHQRL
85375834        NIARLYDGGETDDGLPFIVMEKVEGIPLGDWIDQTRPSLPERLALFLKVCDAVSYAHGHL
21244844        AIAQIHDVGTDAHGQPYLVMEYLRGEPITWWCDEHRLSLHARVLLMLRVGEAVQHAHQKG
                 *::: * *    * *::.*: : *  :  :          *: *:  :  *: .**

37520154        VIHRDLKPSNILVNCEG---EPRLLDFGIAKLLDPQ-GRSS--EPTRTDLRVLTPRYASP
37523641        VLHRDLKASNILVDGAG---EPKLLDFGIAKLLDEQ-APEA--EQTATEWRMLTPSYASP
37520484        VIHRDLKPANILIDPEG---NPRLLDFGIAKLLPADPEATA--VFSQSMTRLLTPGYASP
116620747       IVHRDIKPSNILVTPEG---IPKLLDFGIAKLLGPGMDDFTG-AVTIAGERPMTPDYASP
116626655       IVHRDIKPSNILVTPEG---IPKLLDFGIAKLLGPGMDDFTG-AVTIAGERPMTPDYASP
116626939       IVHRDIKPSNILVNRDG---VPKLLDFGIAKLLDAEAEDGT--ASTATGARPMTPDYASP
94971075        VVHRDLKPSNILVTSEG---IPKLLDFGVAKLLGPSLDPGL--TSTWSAMGPLTPEYASP
116622939       VVHRDLKPGNILVTAGG---EPKLLDFGLAKLQSP-IDPCE--GFTRTALPFLTPAYCSP
116620251       IVHRDLKPGNILVTTEG---APRLLDFGLARVLER--DAAS--EEATQGIPLMTPAYASP
85375834        VVHRDLTPSNILVELTN---EPKLIDFGIAQPDGTERRDADGGAPLVLRHLTLTPGYAAP
21244844        VIHRDLKPSNVLVSEIDGRPMPGVIDFGIAV------DATN--PGMTYAHDRGTPGYMSP
                ::***:...*:*:   .    * ::***:*                       ** * :*

37520154        EQIAGAE-LTPASDVYALGVVLYELITGQRPAGAQAAASYELAWTLSDQTALLPSRAVGE
37523641        EQIRGEA-AGPSSDVFSLGVVLYELLTARRPAGLNVGPLDEMLWTLGEQAAVPPSRAVAA
37520484        EQIKGEA-ITPASDEYSLAVVLCELLSGRRPG------------------EATPQQLTG-
116620747       EQVRGEP-VTTATDIYSLGAVLYELLTGQRAHHIEAYSREAIEKEICAREPKPPSTITR-
116626655       EQVRGEP-VTTATDIYSLGAVLYELLTGQRAHHIEAYSREAIEKEICAREPKPPSTITR-
116626939       EQLRGEQ-VTTASDIYSLGAVLYELVTGRRARLAAKYTAAEIE-AICKSDPKRPGAVCA-
94971075        EQIQGLP-ITTAADTYALGAILFELLTGRRAQKIAGHSPAEIERVVCHVEIPAPSAVEKT
116622939       EQVLGEK-ITTRVDVYLLGLILFELLTGSQAHQLTGSSPAELQQIVSEEHTPVPSARATQ
116620251       EQVRGEP-DAVPGDVYSLGVILYELLAGRRPYEVKTGSLLEMARAICEQEPAPLSQGASG
85375834        ERLRGET-ATTLSDIYSAGRLLNFLVPKPRPA----------------------------
21244844        EQARGAQDVDARSDIYALGAMFYELSCGLAPVAGRDGVPQPPSQRVAAVPADARARICAA
                *:  *        * :  . ::  *     .

37520154        QPTRAFAPAAAEGGAQGLSRQLEGALDRIVLRSLSKPLDRRYATVGELLTEVRRYL---
```

```
37523641        GTDAGLLADPQK-------VQIDGSIDPLVLCALAKAPADRYTSALAMAEAIRGYL---
37520484        --------------------KLAGPLASLVLKALCEDPLDRFGSVAAFSLAIERHL---
116620747       --------------------EVDSDLNNITLLALRKEPQRRYGSVEQFSEDI-------
116626655       --------------------EVDSDLNNITLLALRKEPQRRYGSVEQFSEDI-------
116626939       --------------------EVGPDLDNIILMALRNEPERRYASVEQFAGDIDRFLQRL
94971075        SG---------------LSLKIDSDLDNIVLMALRKEPERRYRSVNQFAEDIAKYL---
116622939       AGNLA------------LAKSLRGDLDKIVLKAIQKDPARRYQSVDEF-----------
116620251       S----------------LARRLVGDLENIAAKALAKDACRRYPSVGELAADLRRHL---
85375834        ------------------------ELQAIANKATASETDDRYQSIEALAADVRRHL---
21244844        RATTYQK----------LHEQLRDGLDAIVLRALEPQPGARYASVSAL-----------
                                         :  :   :       *: :   :
```

Cluster No. 51 multiple sequence alignment

CLUSTAL 2.0.10 multiple sequence alignment

```
37519623        YRLLGRLDGGSMGSVYEAADTKLAGKVVALKVMHRSLAGDT-EVVKLLRQRFEEEARLSA
37520234        YRLTRYIDGGGMGKVYEAVDTRLGDKAVAVKLLQQNLNVDD-RLFEQLRRRFEQEAQLCA
86605092        YRLTHHISEGGYGNVFEAVDTQLNDEPVAVKLLRPPPPDMEPEYYQQLQQRFLDEARVSA
86607459        YQLVQSIAAGGMGQVFKAVDTRLFNRPVAVKLLHQNLAGDE-NTRRHLLKRFQQEIRIST
86606900        YRLIQEIGGGGMGSVFRALDLHNNHQEVAVKILHAPLLLSRGDAQIDLRRRFAEEIRISI
                *:*   :  *. *.*:.* * :   . **:*:::             * :** :* ::.

37519623        ILGSHPRIIQVTDYGVEGPQPYLVMELL-----KGRSLKEVL-AQGPMPPGRAVRLAVQL
37520234        LLGGQHGIIAVSDYGLDGPQPYLVMEYLGAAP-RGRSLKELVSAEGPLSPERTVRLAVQI
86605092        LLGEHPNIVQVRSYGLYQNQPYLVMEYLKAKPYTGQGLDYVLAREGPLHPERVVNLALQI
86607459        LLGEHPAIVKVLDYGLENNQPYLVMEYL-----TGRSLGELLLKQPLLPPQQVVKIARQV
86606900        LLGQHPRIVKVLDHGQEGEQAYLVMEYL-----KGQDLGKLMREKGALPLRQVIRLALQA
                :** :  *: * .:*    *.***** *      *:.*  ::  :  :   :.:.:* *

37519623        CDGLQHAHAAQATVEGRTIRGIIHRDIKPGNLF---LIEDESLGETVKILDFGIAKANSD
37520234        CESLQYAHGVRTHLGGRQITGVVHRDIKPSNIF---VIDRALVGETTKVLDFGIAKAVSD
86605092        CSALHHAHNFHMDLGKHSIRGVIHRDIKPSNIF---VQKGPDGKERVKLLDFGISKLMGE
86607459        CAGLYYAHNLETEQDGHLIKGVIHRDIKPSNLF---VLKDETLGETVKILDFGIAKLLSD
86606900        CEGLHFAHTFRAKVDGREIRGVIHRDIKPSNLFLEQVLQEGQLTSQLKILDFGVAKTLAD
                * .* .**  .     : * *::******.*:*   : .     .  *:****::*  .:

37519623        ISLALGTQAG--FVGTSGYASPEQLRGEALDARSDIYSLGVVLYQMLTGQMPLKPKTETF
37520234        VTIAMGTNMG--FVGTCDYASPEQLRGEELDARSDIYSLGIVLYQMLTGQLPLQPKTHSF
86605092        TSRGL-TQTGY-FLGTMVYASPEQMRGEKLDGRSDIYSLGVVLYELLTGALPFEPETDTL
86607459        VSLALGTQTTG-FLGTVRYASPEQVRGEELDPRSDIYSFGVVLYRMLTGQHPLKPKTDSF
86606900        HTLSLGTHKGGGFIGTARYASPEQIRGKSLDARSDIYSFGVVLYEMLTGSMPFRLETDSL
                 : .: *:    *:**  ******:**: ** ******:*:***.:***  *:. :*.::

37519623        AGWYHAHNYTTPTGFQAHRLPYMLPPALAAVVLSCLEKDPANRPQSMQMLGMQL
37520234        AGWYQAHNHESPVPLPRLAVGQAIPPRVAAVVMACLEKEPARRPASMQELSQRL
86605092        QGWYHVHNFQKPRPFQDHPLPHPIPEALEKVVLRCLEKDPALRPATMEELAQQL
86607459        PGWYEAHNYQQPRPFDRSQLPHEIPPALEEVVLACLAKDPGQRPPNMKLLSDQL
86606900        HSWIHAHCYESPLEINPETTPQPIPPALAAVVMDCLKKNPDERPQTMQELGERL
                 .* ..* .  *  :        :*  :  **: ** *:*  ** .*: *. :*
```

Cluster No. 52 multiple sequence alignment

```
CLUSTAL 2.0.10 multiple sequence alignment


15921045        YKVEKVIGTGGMGFVLLVEKNMKKFAMKVIKKEFRYDELLYEIAKMQEISKGSKYLVKIM
70606963        YKVEKVIGSGGMGYVLLVEKGMRKFAMKIVKKEFRYDEMLYEIAKLQEISKGSKYIVRIF
13816645        YEVKQLLGNGGMGYVLLVERNGKKYAMKVMKKEYTFIEMLYEVAKMQEISKRSEYLVKIF
146303616       YEIRSVLGRGGMGYVLLAEKNGKQFAMKVMKKEYRMDEFLYEVAKMQEISKGSRNMVRIL
                *::..::* ****:***.*:. :::***::***:   *:***:**:***** *. :*:*:

15921045        ASFVDENFSDYYSSPPAVVMEYMEGGDLREILVNQEYSTLRHSSKWPQIVSVIYSKLADA
70606963        ASFVDENFTDYYSSPPAIVMEYMEGGDLRQIITNNEYSTLRHSSRWPEIVSIMFSKIADA
13816645        ASFLDENWTDYFSSPPAIIMEYMEGGDLRSILVDQEYSALRHSVKWPQVVALIFSKIAKA
146303616       ANFIDENWTDYYGSPPAIVMEYMAGGDLRRILADEEYSSLRHSVRWGEVVSLIYSQIADA
                *.*:***::**:.****::**** ***** *:.::***:**** :* ::*::::*::*.*

15921045        IIHIHKNGYVHCDIKPSNILFNRKLPKYGEEALEALLNEEVVPKLSDLGSAVKVGVPVIH
70606963        IIHVNKNGYVHCDIKPSNILLNGKLPKYGEQAVQALLEEKITPKISDLGSSVKVGTPVIH
13816645        VIEVHKEGYTHCDIKPSNILFNKKLPRYGEDALNSLLNFEVVPKLSDLGSSVKIGTPVMH
146303616       VIHLHKQGYVHADIKPSNILFDRALSKYGEEAEQQLLKGDVIPRLSDFGSSIRVGSPVIH
                :*.::*:**.*.********::  *.:***:* : **: .: *::**:**::::* **:*

15921045        YTPYYAHPLQRFGGKAEYNFDVYSFTVSLYVTLTNNFPFSEWLERELEEAVTDPSKREIA
70606963        YTPYYAHPLQRFGGRAEYNFDVYSLTISLYVALTNNFPFPEWLEREIEEAVIDPSKRENV
13816645        YTPYYAHPLQRFGNRAETMFDVYSFSVSLYVSLTNNFPFPEWLENEIEEAVKNPEKRKQA
146303616       YTPYYAHPKQRFGGKAETSMDVYSFVVSLYVTLTNNFPYPEWLERELEEAIISPEKRADA
                ******** ****.:**  :****: :****:******:.****.*:***: .*.**  .

15921045        LKDFYLAEPRLDQIPDEFRDLVERGLRGEITLEEMSREL---------
70606963        IKDFYNMDPRLDYVPSEFHDLILRGLRGEITIEGISRELKQITRTEYG
13816645        LDDFHNATPRLDYVPAEFKDLITMGLKGEISMLEINKRL---------
146303616       LKDFYSVEPRMDYVPQEFREIILSGLRGDPTMEQIRREL---------
                :.**:   **:* :* **::::  **:*: ::  : :.*
```

Cluster No. 53 multiple sequence alignment

CLUSTAL 2.0.10 multiple sequence alignment

```
116622793       YEILSLLGSGGMGEVYRARDQRLARTVALKVLPPEFAADSIRRQRFEQEARAASALNHPN
116623625       YEIVAAIGAGGMGEVYRARDARLGREVAIKVLPEAFARDAERMLRFEQEARAVAALNHPN
94967294        YEIQSQLGAGGMGEVYRAKDLRLDRSVAIKVLPGHLSSNPELKERFVREARAISSLNHPR
94971144        YEIQSPLGAGGMGEVYRAKDLRLDRTVAVKILPGHLSDNPDAKQRFDREARTISSLNHPN
94971417        YEIQSPLGAGGMGEVYRATDTRLDRIVAIKILPAHLSANPEARQRFEREARSISALNHPN
116622781       YRIDSKLGEGGMGVVYRAFDTHLDRPVAIKLLRPDAIGSAERRRRFVQEAKSASALNHPG
116625466       YRIIGELGAGGMGIVYKAQDIRLERFLALKFLKPERVN-DDFRRRFLQEARASSALAHPS
116624547       YEIREKLGSGGMGSVYLAFDTRLNRSVALKILSIENWEGTDGASRLLREAQAASALNHPN
94969605        YQVDALLGKGGMGEVYTARDTRLQRTVAIKILPSHLSYNPDLRARFEQEAKSISALQHPN
94970396        YRIVEKIGGGGMGVVYKAEDTRLHRFVALKFLPDTVSTDPQALARFQREAQAASALNHPN
94971006        YRIVEKLGGGGMGVVYKAEDTRLHRFIALKFLPDNVAADPQALARFQREAQAASALNHPN
94971419        YRITEKLGGGGMGVVYKAEDTRLHRFVALKFLPPELARDPQALARFQREAQAASALNHSN
94968483        YRIVERLGGGGMGVVYKAEDTRLHRFVALKFLPDELARDSQALSRFQREAQAASALNHPN
94971143        YQIVEKIGGGGMGVVYKAEDTRLHRFVALKFLPANVAGDAQALARFRREAQAASALNHPN
94971416        YRILEKLGGGGMGVVYKAEDTRLHRFVALKFLPPELARDPQALARFQREAQAASALNHPN
94968081        YRIVEQIGGGGMGVVYKAEDTRLHRLVALKFLPEDVAEDTATLARFQREARAASALNHPN
116622913       YRIVGKLGSGGMGVVYKAEDILLHRFVALKFLPEHIVHDARALARFQREARAASALNHSN
94967801        YVVAERLGGGGMGVVYKATDSRLGRSVALKFLPDDISHDPQAIERFRREARAASALNHPN
94971418        YHILEKLGGGGMGVVYKAEDTRLRRFVALKFLPDPVANDPQTLSRFQREAQAASALNHPN
94969422        YRVLEELGGGGMGVVYKAEDSKLGRLVALKFLPADVTPDRGTLERFQREARASAALNHPN
94970962        YVVSRKLGSGGMGVVYQAEDTTLGRHVALKFLPDSLAQDSHALERFQREARASSALNHPN
94970115        YQVLEEIGSGGMGVVYKAQDTRLGRFVALKFLPEEFANNPEVLARFRREAQASSALNDPN
                * :   :* **** ** * *  * * :*:*.*            *: :**:: ::* ..

116622793       ILSVFDMG----SQDGLVYIVSELIEGESLRDLIRRG-PLPQSRAVEIAGQVADALAAAH
116623625       VLSVFDIG----TQDGVPYLVSELLEGESLRDVLRTG-PITSRKAVEYARQIADGLAAAH
94967294        ICTLHDVG----QQEGVDFLVMEFLEGESLAQRLQKG-ALPIKEVLKIGVEVSEALEVAH
94971144        ICTLYDVG----QQEGTDFLVMEFLEGETLADRLRKG-PMPIAQLLKCAIEVCDGLDRAH
94971417        ICALYDIG----TQDGTSFLVMEYVQGETLEARRQKG-PLPLKQVTEIGIQVCDALEKAH
116622781       IIHIYDIDKAILPEGPVDFIAMEFVPGRTLEQCIGKV-GLSLKDTLKFGIQVADALARAH
116625466       IIHIYDIG---VFDG-MDYIAMEFVEGRSLRDVLRDA-RLSVEDTVKFGIQIADAMSMAH
116624547       IITIYEVG----REHEIDFIAMEHISGKTLAK-LATR-RLSPRELIPLLIQIADALAAAH
94969605        ICVIHDVG----SQDDIEFMVMEYVQGDTLDKLIPKG-GLPAEIAIRYAIQIADAIGCAH
94970396        ICTIYDIG----EENGKAFIAMEYLDGQTLKHRVDNR-PLAMDELLPIAIDVADALDAAH
94971006        ICTIHDIG----EENGKAFIAMEYLDGVTLKHLIEGR-PLDMERLLPIAIDVADALDAAH
94971419        ICTIYDIG----EDNGQAFIAMEYLDGVTLKHRIEGR-ALDLEVLLPIAIEIADALDAAH
94968483        ICTIHDIG----EENGRAFIAMEFLDGLTLKHTIEER-SMEMDRILALAIEIADALDAAH
94971143        ICTIYDIG----EENGQAFIAMEFLDGMTLKHKASGL-AMDMDTILPFAIEIADALDAAH
94971416        ICTIYDIG----DDNGQGFIAMEFLEGMTLKHRINSQ-PVDLETVLTLAIDIADALDAAH
94968081        ICSIFDIG----EQEGRAFIAMEYLDGVTLKHLIGRS-PVEMSKLLGLAIEIADALDAAH
116622913       ICTIHEVT----EYNHQPVIVMELLEGDTLRERIRGG-ALPLEEILNLAVQATDALDAAH
94967801        ICTIYDIG----EYEGRPFIAMELLEGQTLKHRIGTR-PMDITEILDAGIQIANGLDAAH
94971418        ICTIYDVG----EDDGRVFIAMEYLEGFTLKHLIEGT-PLKTAQFLDLGIQIADALDAAH
94969422        ICTIHEIG----EHQGRPFIVMELMEGATLKHLIAGR-PMRMDRMLELGIQISDALDAAH
94970962        ICTVYAIE----QHERQHFIVMELLEGQSLAAMIGKS-AFEIEQLLALAIQIADALESAH
94970115        ICTVHDIV----DYEGRTFIVMEYLEGANVRERIKERGPFAIEEFFRIAISITEGLADAH
                :  :. :            :. * : * .:         .          .  :.:  **

116622793       AANIVHRDLKPENIMLTRDGRAKILDFGLAKQ--VQPRVPGSDET--QFLTRTS------
116623625       EKKIVHRDLKPENLFLSGGGRLKILDFGLAKMPVTESAAAGVDAATATIVAVTS------
94967294        RAGIVHRDLKPGNIMLTKTG-AKLMDFGLAKAVESTMAAGTSSAPLLSGAPTMSG---LS
94971144        RSGVVHRDLKPGNIMLTKGG-AKLMDFGLAKAAVASASAVSSLSMTLS-TPAQS------
94971417        RAGIIHRDLKPGNIMLTASG-AKLLDFGLAKAVGVLGAQAATAGTHTPDTPTMNVSALRA
116622781       AAGIVHRDLKPANIIVSDDGRVKLLDFGLAKLTEKIDGDPDGVTATMG----TG------
```

```
116625466           AAGIVHRDLKPGNLMITPARLVKILDFGLAKVANPNTTSVMSVSAESTNSVTTL------
116624547           TAGIVHRDLKPSNIMVTDRGLVKVLDFGIAKIK------PGGNPADDP------------
94969605            SAGIVHRDLKPSNVIVDKSGLVKVLDFGLAKTS----ALAAQAGAMET------------
94970396            TAGIVHRDIKPANIFVTKRGAAKVLDFGLAKVSGRNPSSSQIASANTMTMA-AED-----
94971006            AVGVVHRDIKPANIFVTKRGHAKILDFGLAMVSPRSESATVIASANTMSAA-IAK-----
94971419            AAGIVHRDIKPANIFITKREHAKILDFGLAKVEVLAST-----SAATMTAG-VDE-----
94968483            AAGIVHRDIKPANIFVTKRGHAKVLDFGLAKVENVAGSQTAIASANTMTAAGVQE-----
94971143            TAGIVHRDIKPANLFLTKRGHAKVLDFGLAKVGGAAAASATSKPADNTISV-VRD-----
94971416            SKGIVHRDIKPANIFVIERGHAKVLDFGLAKVTPRSASSVPSANTMTATELAVED-----
94968081            TQGIVHRDIKPPNIFVTKRGHAKILDFGLAKMSQEQETES---RLVAVTSDGLTG-----
116622913           EKGIVHRDVKPANIFVSKRGHLKMLDFGLAKVDSPLAS------TETDAPTATIE-----
94967801            TKSIVHRDIKPANIFLVEPGEAKILDFGLAKVTLKNAVSA-AETMATMPAPVVSE-----
94971418            SAGIVHRDIKPANIFVTPRGQAKVLDFGLAKLGQVASRGG-ASDVTVGNSAVVGT-----
94969422            AKGITHRDIKPANIFVTNHGQAKILDFGLAKLDGRGIRSR-AAAGADLG-VTISE-----
94970962            AKGIVHRDIKPANIFVTPRGQVKILDFGLAKMELLKPSVG--VATVSQMETVGQR-----
94970115            RHGILHRDIKPANIFITDRGRVKILDFGLAKMGIQQLG----------TNTGDDD-----
                       : ***:** *:::      *::***:*

116622793           -------PGAILGTAGYMSPEQVRGEPVDPRSDIFSFGLVLYECLSGRAPFERQTGAEMM
116623625           -------PGVVMGTTGYMAPEQVRGDAVDHRADIFSFGATLYEMLTGKRAFQGDSSIETL
94967294            PLSPLTMAGAVVGTVQYMAPEQVEGKLADARSDIFALGATLYEAATGKRAFDGKSQIAVA
94971144            --HPLTAEGTVVGTFQYMAPEQVEGREADARSDIFSLGAVLYEMATGKRAFEGKSAASTM
94971417            PAAGLTQQGTIVGTFQYMAPEAAEGLATDARSDIFSLGCVLYEMVTGRRAFEGKSQLSVL
116622781           -ESPETDEGTIVGTVAYMSPEQAEGRKVDGRSDIFSFGSVLYEMATGRRAFEGINKISTL
116625466           -TAEQTQFGSALGSPAYMSPEQATGKLVDGRSDIFAFGAMLYEMLAGQRAFTGDATIEVI
116624547           -TQTITQLGQVHGTVAYMSPEQAEGREVDHRSDIFSFGCVFYELITGQRAFHADTGLATL
94969605            -ITVGTSPGTIVGTVAYMSPEQAEGKAVDTRSDVFSFGAVFYEMLSGHRAFEGESSAALL
94970396            --PHLTSPGSTLGTVAYMSPEQARAKELDARSDLFSFGSVIYEMATGQLPFRGDSTASIF
94971006            --EQLTSPGSTLGTVAYMSPEQARAKELDARTDLFSFGAVLYEMATGTLPFRGDSTATIF
94971419            --KQLTSPGSTLGTVAYMSPEQARAKELDARTDLFSFGAVLYEMATGQLAFRGDSTATVF
94968483            --QHLTSPGSTLGTIAYMSPEQARAKDLDARTDLFSFGAVLYEMATGTLPFRGGSTAEVF
94971143            --EDLTSPGSTLGTVAYMSPEQVAAKDLDARTDLFSFGVVLYEIATGRLPFPGESTGLIF
94971416            --EHLTSPGSTLGTIAYMSPEQAKGKELDARSDLFSFGSVLYEMVTGALPFQGETSALMF
94968081            --EHRTSPGTTMGTIAYMSPEQARGKELDARTDLFSFGVVLYEMSTGTLPFRGESSVETF
116622913           --ERLTDTGSTMGTAWYMSPEQVRAKELDGRTDLFSFGVVLYEMATGTLPFRGESQGVVF
94967801            --DQLTSPGSSLGTVAYMSPEQARGEELDARSDLFSLGVVLYEMATGQLPFAAATAALMF
94971418            --DQLTSPGSTIGTIGYMSPEQARGEVLDHRSDLFSFGVVLYEMATGQQPFSGATSAVVF
94969422            --DDLTSPGATLGTVAYMSPEQARGETLDARTDLFSFGAVIYEMTSGNIPFSGNTTAVIF
94970962            --DDLTIPGTALGTVAYMSPEQARGQFTDSRTDLFSLGTVLYQMGTGVLPFQGDTTAVVY
94970115            --DATKTRGWAFGTVAYMSPEQALGKPLDQRSDIFSLGTVFFEMLAGITPFEGETTGTVF
                            *   *:  **:** . .   * *:*:*::*  :::  :*  .*

116622793           TAILREDPPELPET---VFPALRQIVHHCLEKDAVRRFHSANDLAFALRTV-----
116623625           NAILKEDPPEFDSEKLHVSPGLERIVRHCLEKRPGERFQSARDLTFALSALSDPSG
94967294            NSILEKDPEPASTLNPQVPRGVDHVIARCLAKDPEQRWQTARDLGLEL--------
94971144            AAILERDPAPISTIQPTTPPALERLVKTCLAKDPDERWQTAHDVKLQL--------
94971417            TAILERDPEPISTIQPLTPLALEYTVHTCLEKNPDQRFQTAHDVKLQLVWI-----
116622781           SAILHKEPKAASEISQAVPVELEKIIARCLRKDLERRAQGIADI------------
116625466           SAVLRHDPPVPSASNSNAGPELDAVVMKCLKKSPHDRYQSMEEVKLAL--------
116624547           AAVVAKDPRPVRELVPDLPRSVERILENCLRKRRNDRWQSIEDVKLVLEAALADL-
94969605            ASVLRDEPKPLTEVRRDLDPEIRRIVTRCLKKDPAARYADGNDLARDLKRCRETL-
94970396            DAILNRQPTAPVRLNPDIPAELERIINKALEKDRDLRYQVASEMRADL--------
94971006            DAILNRAPVAPVRLNPDLPAKLEDIINKALEKDRNLRYQSAAEMRADL--------
94971419            EAILNRAPVPPVRLNPDLPPKLEDIINKALEKDRNLRYQHAADIRADL--------
94968483            KAILDTAPTPMVRMNPDVPPELERIVSKALEKDRNLRYQSANDMRADL--------
94971143            KAILDNAPEPMTKLNPAIPAEFERVVFKALEKDRDLRYQSAAELRADL--------
94971416            DAILNRDPLPPLRFNPKVPAKLEEIIQKALEKDRDLRYQHASEMRSDL--------
94968081            EAILGRVPVAPVRLNPDVPHELERIISKALEKDRNLRYQSAAEMQADL--------
116622913           DAILNQTPVPPVRLNPDLPAELERIIAKCLEKDRNLRYQHASEIRSDL--------
94967801            DGILHSEPKSASEVNHRIPLAFDTLLNKAMEKDRDLRCQSAAELRADL--------
94971418            EAILNKVPTPANDVNPTVPKQIEEVLSKALEKDRELRCQSAAELRADL--------
94969422            NNILNISPKPLPQSIPDVPLELDRIVSKALEKDRELRYQTAAELRGDL--------
94970962            EAILNRDPVPVAEVNGALPQAFARIVEKALEKDRTLRYQTANDIKTDL--------
94970115            LAVVQNTPVIPVQEIPNTPAGLKRIVGKCLEKDREKRYQSMAELRDDL--------
```

```
                  ::   *             .   :  .: *    *     ::
```

Cluster No. 54 multiple sequence alignment

```
CLUSTAL 2.0.10 multiple sequence alignment


1653478         YQTLGLLGKGGFGATFAAADVA-LPGTPICVVKQLRPQTDDPNV-FRMAKELFEREAQTL
22299083        YRILSQLGQGGFGRTFLAADLH-LPDHPICVVKQLVPSRKDERF-LAIARRLFQREAETL
113477484       YKIIKIIESEGLAHTYLAQDIR-RPGAAECFIKHLQPSISDQKF-LEIIRRRFQQEAQIL
113478146       YRVVQILNSGAFGQTYLAADTR-RPGHPQCVVKVLR--TPSNSL-LKTAHRLFKQEAEIL
17132866        YQIVQNLGSGVFGQTYIAVDIN-YPHQPKCVVKQLKVNSFHSSH-LDTIRLRFLTETETL
113476415       YKVIKSLGTGGFGYTYLAEDLD-LPGYPKCVVKHLKPKSPDSTV-LNIARKLFLREADIL
113476417       YKIIKSINNGGFGDTYLAQDID-LPGYPKCVVKHLKPKNLDPRV-LNVARKLFEREADTL
113474333       YKIIKPLGSGGFGDTYLAQDSD-LPGKPKCVVKHLSPKSSDPMV-LSIARKLFEREAEAL
113476216       YEIIKTLTAGSFGQTYIAINKHSQPPNQEVVIKKLKPQQNDPYT-LQNAERLFKKEVESL
113475689       YQGIKPIGNGSFGRTLLAVDLD--RFNTPCVVKQLAPKNNNLLS-NNKVLELFKREAKQL
1653955         YRIIETLGRGGFGETFLAQDTH-MPSARKCVIKHLKPVLENPEIPSWLRER-FHREAATL
1006577         YEIVKSLGSGGFGDTFLAKDTQ-IPSQKLVVIKRLKPANANSNTSTELIQKLFEKEASVL
                *. :  :    :. *  * :          .:* *                 *  *.  *

1653478         GRVG-NHPQVPRLLDYFEDDHQFYLVQEYVKGHNLHQEVKKNG----TFTEGSVKQFLTE
22299083        AQLG-QHQRIPRLLAYFEEGGYFYLTQEYVDGESLKEEFEKKI----TLSQGEAIAILKS
113477484       EKLS-QNNQIPKLLAYFEENQEFYLVQSFIPGKSLDNEILPGR----ALSEIQIIRILIE
113478146       EKLG-RHSQIPLLLAYFEENNQFYLVEEFIYGTPLEKEIIPGK----PWSEKQVIKLLLE
17132866        KHLG-QHPQIPNFIACFEENERFYLVQEYIGGHALTAELPIAQNWGSVWREDEVITFLED
113476415       YKLGNDSDQIPRLFAYFQEQREFYLVQEYIEGQDISRELTPGK----KLSESDTIALLKG
113476417       YKLGNDSDQIPKLLAHFQEQREFYLVQEHIEGQDISREMTPGK----KLSESDTIALLKG
113474333       YRLGTDSDQVPKLLAHFQEEKEFYLVQEYIEGQDISRELTPGK----KLSESDTIALLKG
113476216       KKLG-YHKQIPTYIDNFEENNEFYLVQEYIKGKDLTEELKPGN----KLSESEVIHLLID
113475689       LKLG-KHPQIPTFYAYFEEDENLYLVEELITGKNLLQELESEG----AFSEKKIWKILYE
1653955         EELGENHPQIPQLYAYFSEGEDFYLVQEWIPGLTLTQAHAQKG----NFSSTAVEELLLG
1006577         EDLGEHNSQIPKLYSYFSNDNEFYLVQEYIQGVSLNEIAP--------ISSEQAKTILSS
                  :.    ::*     *.:   :**.:. : *  :               .     :*

1653478         ILPILDYIHSQKVIHRDIKPANLIRRQ----------TDQKLVLIDFGAVKNQIDSVLSS
22299083        ILEILQYVHQFGVVHRDIKPANIIRRR----------SDQQLFLIDFGAVR-----HVQP
113477484       VLEILVFVHRNNVIHRDIKPANLIRRK----------LDNKLVLIDFGAIK---EIN--I
113478146       ILEILGFVHRYEVIHRDVNPSNLIRRQ----------LDRKLVLIDFGSIK---EMSHQI
17132866        ALSILQFVHSQGVIHCDVKPENLIRRA----------VNGKLVLIDFGSIQ---SVNFGI
113476415       ILEALTVAHQNNVIHRDIKPQNLMRRR----------SDNKIVLIDFGAVK---EIDVLT
113476417       ILEALTVAHQNNVIHRDIKPQNLMRRR----------SDNKIVLIDFGAVK---EIDVLS
113474333       ILEALVVAHENNIIHRDIKPQNLMRRG----------SDNKVILIDFGAVK---EISILT
113476216       ILEVLDFVHKNGVIHRDIKPSNLMRRT----------EDNQIVLIDFGAVK---EVGTVL
113475689       ILPLLKFIHENKVIHRDIKPENILRVDGVNSLKVGENQKDQLVLIDFGVSK---FSSEVD
1653955         ILPVLEFIHQRRIIHRDIKPDNIILRE----------ADGKPILIDFGIIK---ETMGTL
1006577         LLTTLKYIHSKGIIHRDIKPENIILRD----------SDHLPVLIDFGAVK---ETMGAV
                 *  *   *  ::*  *::* *::              .   .*****  :

1653478         NTSAQTALTAFAVGTAGFAPPEQMAM-RPVYASDIYATGVTCLYLLTGKTP-----KEID
22299083        EDLLRHGKYTISIGTRGYAPSEQMAG-RPVIASDIYSLGMVIVEGLTGLAP-----MDLP
113477484       AQPS----RTARIGTMEYMPIEQFEY-NPQLNSDIYALGMMGIQGITGLPSSELSKLKYQ
113478146       AQENGARQRTIATGTPSYMPIEQFQG-IPQYNSDIYAVGMIGIQALTGIPSGELPKLQDM
17132866        DEQVS--IYQVPATSLGYIPPEQFIG-KTQINSDIYALGMIAIQALTGLEP-----LQLK
113476415       INQQGATTLTVAVGTPGYMPSEQSNG-KPKLSSDIYAVGMVGIRALTGKEP-----QSLL
113476417       TDQNGQTILTVAVGTPGYMPSEQSNG-KPKLSSDIYAVGMVGIRALTGKKP-----QSLL
113474333       IAPQGATTLTVAVGTPGYIPSEQSNG-KPKLSSDIYAVGMVGIKALTGKDP-----QSLP
113476216       VNQQGQKTITVIIGTPGYMAGEQGQG-HPECASDVYAVGKIAIQALMGVSPNLLAPNLLL
113475689       YDKIG-----TITGTLGYSPIEQIRGGKAYPASDLYSLGMTCIHLLTEVPP------NQL
1653955         VNPDGRSAYSVALGTPGYMASEQAAG-RPVFSSDLYSLGLTAIFLLTGKTP-----QYLT
1006577         TLGSGSTVSSVVIGTRGFMAPEQSSG-RSVFSTDLYALGLTIIYTLTKKLP-----VEFS
                              :  : . **     .   :*:*: *   :  :    .
```

```
1653478         CNSQTGEMDWE-----KHVTVSSKFAEVIRKMLELSVRHRYKSAQQVLDAL---------
22299083        SDPDSGDLIWQ-----PGRHLSPQFVAIINKMIKYNFRDRYQSAREVLTDL---------
113477484       ENGNKKEIFWR-----NRINCSTALADVLDKMVRYNYRERYQSATEVIEDL---------
113478146       SKSNSSGILWR-----SQAQCSSNLADILEKMVHCHYAQRYQSIEKVVI-----------
17132866        IDPDSNEIIWRF----ADTPVSDYLAAILSQMIRYNFQERFQSAAEVLRVL---------
113476415       TDPKTGNVIWR-----NEAQVSNRLADILDKMVHEYFPQRYENAMEVLDVL---------
113476417       TDPKTGNVIWR-----NEAQVSNHLANILDKMVHEYFPQRYENAMEVLDVLLGAKGKINP
113474333       TDPNTGNIIWR-----NEAEVSNDLANVLDRMVRDHFSERYKNAMETM------------
113476216       RNQNNPELLWR-----QGVLISQELGNIIDKMVEQNCLLRYHNAGEALQEI---------
113475689       FDSFSGELIWRSHLIKKGKNISDRLGKILDKLVKDLVKERYQSATEVLIEI---------
1653955         SDSRTGEILWRQ----GAPQVSPTLAKVIDQAVRYHPRERFNSATAMAQTL---------
1006577         SDQQTGQLDWQS----HVSKIDSVLAKVINKAIEMEPSRRYSSAEAMYQAL---------
                 .  .  : *.          .  :  :: : :.     *: .

1653478         -------------
22299083        -------------
113477484       -------------
113478146       -------------
17132866        -------------
113476415       -------------
113476417       PKPKQRPTAKTTV
113474333       -------------
113476216       -------------
113475689       -------------
1653955         -------------
1006577         -------------
```

Cluster No. 55 multiple sequence alignment

```
CLUSTAL 2.0.10 multiple sequence alignment


116333600       YRIVRALGEGGMANVYLAR-DLILNR-DVSVKLLRLDLR--DDPG--TIRRFQREALAAT
116492599       YKIIRPLGEGGMANVYLAH-DLILDR-DVAVKLVRFDMQ--DDVS--AIKRFQREALSTT
116618605       YRIIKSLGDGGMANVYLAH-DEFLNR-DVTFKMMRLDMK--NDVD--LAKRFQREALSVT
13622698        YRILKSIGRGGMADVYLAN-DLILDNEDVAIKVLRTNYQ--TDQV--AVARFQREARAMA
28895405        YRILKSIGRGGMADVYLAN-DLILDNEDVAIKVLRTNYQ--TDQV--AVARFQREARAMA
19551291        YELDAVIGSGGMSEVFAAT-DTLIGR-EVAVKMLRIDLA--KDPN--FRERFRREAQNSG
23491868        YELGASIGSGGMSEVFAAT-DLLIGR-EVAVKMLRTDLA--KDVN--FRERFRREAQNAG
68535099        YRLGAKIGTGGMSDVYAAT-DELLGR-DVAVKMMRPDLA--RDTT--FLERFRREAQNAA
116668588       YELGELIGRGGMADVHRGL-DTRLGR-TVAIKLLRPDLA--RDPQ--FQARFKREAQAVA
29830914        YQLRDLLGEGGMASVHLAY-DSVLDR-QVAIKTLHTELG--REQA--FRERFRREAQSVA
83646939        YKAQRELGRGAMGVVYQCF-DPDFER-IVAVKVLLPELLG-ADVTGEFRERFRNEMRAAG
124265262       YVVERDIGHGAMGAVLLCR-DERTGE-AVALKTMALGREFHGDALEDARERFFREAQMAG
116622546       YTITGLIGHGGMGSVYRATRVDDFRM-QVAIKLLKRGTD--TDLA---LGRFRAERQILA
32473469        YRVVRRIGSGGFGSVFHAK-DESLNR-DVAIKVPLRSLD---DVN--DEFQWSSEARMVA
29833662        YLLLNRLGSGGMGHVWLAH-DQRLACEVALKEIVFRSPAEAGQERTARVARARAEARHAA
                *     :* *.:. *             .  :          :       :   *

116333600       ELV-SPNIVQVYDVG-------EENG--MQYLVMEYVEGT----DLKAYIKQHFPIPYQE
116492599       ELV-HPNIVGVYDIG-------EEHG--MNYLIMEYVEGQ----NLKQYIRDQFPIPLGQ
116618605       ELI-NDNIVQVYDVG-------EYQG--SQYIVMEYVDGT----NLKSYIGEHFPIAYQQ
13622698        ELN-HPNIVAIRDIG-------EEDG--QQFLVMEYVDGA----DLKRYIQNHAPLSNNE
28895405        ELN-HPNIVAIRDIG-------EEDG--QQFLVMEYVDGA----DLKRYIQNHAPLSNNE
19551291        RLS-HSSIVAVFDTG-----EVDKDGTSVPYIVMERVQGR----NLREVVTEDGVFTPVE
23491868        KLS-HPSIVAVFDTG-----EVDRDGISVPYIVMERVHGR----DLRDIVREDGPYSPSQ
68535099        KLN-HPAIVAVYDTG-----QTPDEDGAVPYIVMERVHGE----TLRDIIQDSGKMSLND
116668588       ALN-HPSIVAIYDTGDHAVPGGPEDTVRVPYIVMEFVSGK----TLRDLIR-AKEVSIDH
29830914        KLT-HTNIVSVFDTG-----EDSLDGLTTPYIVMEYIEGKPLGSVLDADIQQHGAMPADK
83646939        KIS-HPNVINVFDAG-------DQDG--APYFVMEYVEGY----ELKSALDDGQRFPIDK
124265262       RLQ-HPDIVSVREAG-------EQDG--TAYIAMELLSGR----DLSEFTQPGRLLPVPA
116622546       RLQ-HPNIARLLDGG--------ANGTGSPYFVMEYVEGL-------PFLQFVAPLSLRS
32473469        KLD-HPNIVPVYDVG--------KSDQFPFFVVSRFIQGV----DLRERILKGKPS-LEE
29833662        GLRGHPHVVTVHDVL-------EHEG--LPWIVMEYVAGA---LDLRDLVRRRGPLAPAE
                 :     :  : :                 :.  . : *

116333600       VIQIMEQILSAVKTAHEHNIIHRDLKPQNILID------RNQVAKITDFGIAVALSEH--
116492599       VVSLMSQILNGVQTAHYHGIIHRDLKPQNILID------KHGKAKITDFGIAIANQQS--
116618605       VVDIMMQILNAVQAAHNAGIIHRDLKPQNILID------RNDQVKITDFGIAIAKSEQ--
13622698        VVRIMEEVLSAMTLAHQKGIVHRDLKPQNILLT------KEGVVKVTDFGIAVAFAET--
28895405        VVRIMEEVLSAMTLAHQKGIVHRDLKPQNILLT------KEGVVKVTDFGIAVAFAET--
19551291        AANILIPVCEALQASHDAGIIHRDVKPANIMIT------NTGGVKVMDFGIARAVNDS--
23491868        AATIMIPVCHALQSSHEAGIIHRDVKPANIMIN------NTGGVKVMDFGIARALDDS--
68535099        AAAVMSQVCSALFFSHEAGIIHRDIKPANVMIT------NTGAVKVMDFGIARALSDS--
116668588       AIDFTLGVLSALEYSHRAGIVHRDIKPANVMFCE-----DSDTIKVMDFGIARAMADS--
29830914        ALKITADVLAALEISHEMGLVHRDIKPGNVMMT------KRNVVKVMDFGIARAMQSG--
83646939        TLKIMTDVLSGLGHIHEHGIIHRDLKPANIFIT------KNGVAKIADFGVAKLE-----
124265262       VLGIIARVAQALAYAHRQGVTHRDIKPANIMVEL-----KSGMVKVTDFGIARIID----
116622546       RLELFRSVCSAVQYAHRNLIVHRDIKPGNILVT------PEGIPKLLDFGIAKLLDPTSD
32473469        GLIWTASIADALDHAHSNGLVHRDVKPSNILID------TQDRAWLTDFGLAMSDDAP--
29833662        CARIGLAVLDALTAGHERGIMHRDVKPANILLAPDRTGAAYARVLLTDYGISVQPDAG--
                       :  .:   *   : ***:** *::.             : *:*::

116333600       ----NLTQTNTVLGSVHYLSPEQARGG--MVTKQSDIYSLGIILYELLTGT--VPFEGET
116492599       ----SFTRTNTVIGSVQYLSPEQVRGH--IATQQSDIYSLGIILFEMLTGK--VPFEGES
```

```
116618605       ----DLTQTHTVIGSVHYLSPEQTRGG--MASAKSDIYALGVMLYEMLTKQ--VPYEGDT
13622698        ----SLTQTNSMLGSVHYLSPEQARGS--KATIQSDIYAMGIMLFEMLTGH--IPYDGDS
28895405        ----SLTQTNSMLGSVHYLSPEQARGS--KATIQSDIYAMGIMLFEMLTGH--IPYDGDS
19551291        --TSAMTQTSAVIGTAQYLSPEQARGK--PADARSDIYATGCVMYELVTGK--PPFEGES
23491868        --TSAMTQTAAVIGTAQYLSPEQARGK--PADARSDVYAAGCVLYELVTGR--PPFEGES
68535099        --SSAMTQTAAVIGTAQYLSPEQARGQ--SADARSDIYAAGCVFYELATGK--APFHGES
116668588       --SATMTQTQAVVGTAQYLSPEQARGE--TVDARSDLYSAACLLYEMLTGR--PPFIGDS
29830914        --VTSMTQTGMVVGTPQYLSPEQALGR--GVDARSDLYSVGIMLFQLVTGR--LPFEADS
83646939        --SSELTRVGTIIGSPRYMSPEQCQGL--PVDARSDLFAAGIIFYQLLTNE--HCFNANS
124265262       ---STQTRTGIVLGTPSFMSPEQMAGA--RVDGRSDLYALGVTLFQLLTGV--LPHRPES
116622546       GGTAAYTATGARLMTPDYASPEQVRGE--PVTTATDVYSLGAVLYELLTGRRAHKFESYS
32473469        ----RPTRAGLLIGTYSYMSPEQARGEGHLVDGRADIFALGIVLYELLVGR--RPFGGGS
29833662        --ETRYTLTSALVGTPGYLAPERATGG--PPTATADLFSLGCTLYFGVEGCG--PFERDT
                      * .   : :  : :**:  *        :*::: .  ::          .   :

116333600       AVSIVLKHFQSEIPSVRDLD-----PRIPQALENVVLKATAKRPSDRYTDSLSMADDLRT
116492599       AVSIAVKHYQDQLPLVKDFN-----TKIPQSLENVVLKATAKNPIDRYKNVAEMAADLKT
116618605       PVAVALKHATDDMPSVRDFD-----PRIPQALENVILKATAKNPQDRYLDVSVMAEDLKT
13622698        AVTIALQHFQKPLPSIIEEN-----HNVPQALENVVIRATAKKLSDRYGSTFEMSRDLMT
28895405        AVTIALQHFQKPLPSIIEEN-----HNVPQALENVVIRATAKKLSDRYGSTFEMSRDLMT
19551291        PFAVAYQHVQEDPTPPSDFI-ADLTPTSAVNVDAVVLTAMAKHPADRYQTASEMAADL--
23491868        PFAVAYQHVQEEPTPPSEYI-SDLSPTAALNVDAVVLTAMAKHPADRYQTAAEMAADLEL
68535099        PLSVAFQHVQENPEAPSQVLGMHLSKREALSLDSITLTAMAKSPSDRYDDAQEMATDL--
116668588       PVSVAYQHVREIPEPASSLN-----PEVSEALDSVLSKALQKNRADRFQDAAAFQRAL--
29830914        PLAIAYAHVQEEPVAPSSVN-----RSLPPAVDALVARALKKNPNERFPSAEIMRDECL-
83646939        PTAIMQKILHAKPELPSMLI-----PTLSKYYDAVVVKALEKSPEKRYQSAEEFIQAL--
124265262       MAELIRSIANDVAPDVRTLR-----PELPAALADIVALALEKRPEIRYADGEAMAADLHA
116622546       PVEVEKEVCEREPTIPSAVA-----RHLDPDLDNIVLMALRKEPLRRYSSVEQFSEDI--
32473469        SQQLLQDIVRAEPTPLRQFN-----PQLPIELERICLKALAQRVSDRYSNAAAMAADL--
29833662        HLAEVTAVVLEEPRPPVRAG----------ALEPVLAAMLAKDPGQRITAEDTEAALSAI
                                                  :      :    *

116333600       AL------------
116492599       VLQPERANESKFV-
116618605       V-------------
13622698        AL------------
28895405        AL------------
19551291        --------------
23491868        LSRNAVSRAARAHV
68535099        --------------
116668588       --------------
29830914        --------------
83646939        --------------
124265262       VL------------
116622546       --------------
32473469        --------------
29833662        --------------
```

Cluster No. 56 multiple sequence alignment

```
CLUSTAL 2.0.10 multiple sequence alignment


146303114       YDVESILGIGGTSYVLLGRRENKKYAIKIANVSPSSSSSSTRLGISTFSDLSKESSKLQE
146304560       YQVKRVVGKGGTAYVLLGERGGQAYAIKIPFISPASAGERTRLSKTTFADMAGESSKLQE
70607598        YKVESVLGEGGTSYVLSATS-GERFAIKVPKLSQPSSS-ATKLSKVTFEELYKESSNLQR
15921012        YRIIDYLGKGGSAYVLIGEKDNKKYAIKIPILIPLSNV------VESYYDFINEYSQLRE
                * :   :* **::*** .   .: :***:. :   *          :: ::  * *:*:.

146303114       ISERSNDIVKLYGVYADVNTIREILEGKSFLYLTNPPAIVMELMTGGTAEDLVKKEAVFL
146304560       ISTKTEDMVTLYGIFVDRTAITEILSGKVEVYLKSPPAMVMEFMGGGDVDSLLKEQAVFY
70607598        LSEGVENVVKIYGIFLDINLLRRIERGEVKYYLTNPPAIVMEYMEGGTLADLMRNDIVVN
15921012        LSLSSDSIVRFVDAIIDVSAIKRIKDGDVLAYLNEPPILVMEFMEGKSVKELIQNDNVYY
                :*   :.:* : .   * . : .*  *.   **..** :*** * *    .*:::: *

146303114       SSNWPSVVRIIFMSTARALSVVHRENYVHLDVKPRNIFFSEPPGNTGGEVLNNLVNGKTA
146304560       SEKWERIVTFILMRVARALNMVHTEGYVHLDVKTKNIFFSSFPGRSGDEVFENLVTGRVK
70607598        SSHWGDIVKVVARKVAKALDYIHSSGYVHLDVKPQNIFFSAPVGKTGGEVFDSLVKGRNE
15921012        SDEWEKIVLLIALEVVKSLEDIHKAGFVHLDIKPSNILFSSLPGKTGKEVLDNLTQKKVK
                *..*  :* .:   ..::*. :*  .:****:*. **:**   *.:* **::.*.  :

146303114       VKLGDLGSARRRGERITEYTAEYCPVDQVEDMLLGRGARPDMDVFALGATIYRLINGTSL
146304560       AKLGDLGASKKVGGVLDQYTAEYCPVDQVQALLMRSGAHPRMDIYALGATGYKMLTGQIL
70607598        VKLGDLGSAKRVGERVSQYTPNYCPVDQVEALIVGKGAETTMDVYAFGATIYTALTGKAF
15921012        IKISDLGSARKIGEKFSQYTPEYCSVDQVEAIVEGKGADPSMDIYSLGATIYKMLTRKDF
                 *:.***:::: *  . :**.:**.****: ::   ** . **:::*** *  :.   :

146303114       NPPEVVREMDGAVDAFLRRGDFRANLDRARRAYSAVHSSLR---LSRFH-EVEGLVKKMT
146304560       NPAEVVKLMDGAVDEYLNRGNYSVLIDQAFREYQKFYAGLS---LPGVDPELANVIKAMV
70607598        SPPEVVGLVEEAVEDYTRGGSPLGKLKLARDRYEEYYFNVLPSTLTSAPREISEVVIKAT
15921012        NPPELIRLFNEASIIYSNGGDPKPILQKAKEVYKEYYMKLE---IPDVDQRFKDLVKELV
                .*.*::  .: *   : . *.    :. *   *.  :  :    :.    ..  ::   .

146303114       DPDPTRRPSIQEVLNEL-------
146304560       NPDPVRRPTAGQVATNLERILNRM
70607598        HPDPSKRAKVSELLKVL-------
15921012        NPE--KRPTASEVYNKL-------
                .*:  :*..  :: . *
```

Cluster No. 57 multiple sequence alignment

```
CLUSTAL 2.0.10 multiple sequence alignment


108756902       YVVRRKLAEGGMAEIYLCTARGAEG-FEKEVVIKRVRAFLASDPEFVGMFIAEARLASRL
86158893        YRLVELLASGGMADVWRAEVAGAAG-VVKEVALKRVRGEHGARSDFVRMFIEEARLASRL
108759111       YRLSARIATGGMAEVYLGRRIEDDGSRGPSVAVKRLMPHLASDRRVVQMFLNEARITAQV
108763617       YELQERVGQGGMAETWRARLLGAAG-VTKSVLIKKVLPEYANDEAFVSMFISEARISATL
108762650       YEILTRLSIGGMAELFLAYTSGPGG-FRKFVAVKQILPDIKKDEQFVQMFLDEARITAAF
108762968       YEVLCRLSTGGMAEIFLASQRGLAG-FHKLVVLKQILPDIRGEEEFVRMFLDEAKVTAAF
108762958       YELVHPLGQGGMGEVYLAKISGAAG-FEKPCIVKTILPALLKDRQFLDRFHHEAKVLVHL
                * :   :. ***.: :        *       :* :         .:  *  **::   .

108756902       NHANVVQIFDFDKHEDTYYLAMEYVRGCSLWELRKRGKELM-EPVPPMLVAHIGAEVARG
86158893        THANVVQVFEFDQVDGRYYIAMELVRGRHLGQVVERAREAG-VRLGLARAVHACAEVARG
108759111       RHPNVVTIIELGMEGTEPFIAMELLEGRSFAELRQEAAEHG-HRVPLGITLRVLVEACRG
108763617       SHGAIAQVFDFGKVDGQYFLAMELVEGQPLNRILKRALRSGYHSLPVPIAVFIAMEMCRG
108762650       SHANIGQVFDLGEDGGELYLAMEFLPGQNLEQVMKASTRQG-YALPLGFVGRVIRDTCLG
108762968       NHPHIAQVYDLDIADGELFLSMEFVPGATLVEVARACRQAN-TPIPMGYSLMAVRDTAVA
108762958       VHSSIAQVYDMGEADGTYFMALEYVAGVDLAYLLEQARSQG-VAVPVPVALFLGQRIAEG
                 *  :  : ::.      ::::* : *  :  : .         :            . .

108756902       LHYAHR-VRVNGQPLDLVHRDVTPHNVLLSFDGAVKLTDFGIAKA-GNK--LTQPGVLKG
86158893        LSYAHR-LADGGRPLGLVHRDVSPHNVLVSFEGEVKLADFGIARA-MSQGGLTDPGTVKG
108759111       LDAAHRAVDEAGRPLRIVHRDFTPDNIHVGVNGAVKVIDFGIAKADALGSG-TEPGILKG
108763617       LHYAHTRSDEKGEPLGIVHRDISPDNVLIGYEGQVKIVDFGIAKARSLRSFNTEPGVVKG
108762650       LHYAHHFTDPSGRPAVVVHRDVSPKNVMLTYDGVVKVIDFGIAKA-RGRLGRTQVGTVKG
108762968       LHYAHTFTDPLGRPSPVIHRDVAEKNIMVTYEGVTKLLDFGIAKS-LARASRTAVGMVKG
108762958       LGYAHRKTGPDGSPLGIVHRDVSPHNVMVSYEGEVKVIDFGLAKS-AARSKYTLPSTVMG
                *  **       * *  ::***.: .*: :  :* .*: ***:*::       *  . : *

108756902       KFAYMSPEQARGEA-VDARTDIFALGVVLWEMLTGGRLFDGDSEVAVLRAVQQSTIPPP-
86158893        KLAYMAPEQARGAP-VDARADVFALGVVLWELCAGRRLFARDSEAATLAAVLEGAPPPPP
108759111       KFFYMSPEMIAGKP-VDHRADLFAAGVMLYEQLCGRRPFTGLSADEVLGRIAEGRPKP-P
108763617       KYLFFSPEQARGEA-VDARTDVWATGVVLFQMLCGRLPLEG-QVHTVLRRLNSGQPLPSP
108762650       TSGYMSPEQVRGVATLDGRSDLFSVGVMLHELLSGQRLFSGPHEAAVMMQIVEADVPP-L
108762968       TSGYMSPEQIMGEP-LDARSDLFSLGVVLHECLTGMRLFYAKQAEAMMNAVLRCEVTP-P
108762958       KLGYMSPEQVRAEP-LDHRSDIYSCGVVVWEMLAGRSLIPHGTVGEMMAAMSQPVVPS-L
                .  :::**    . . :* *:*::: **:: :   *   :        :  :      .

108756902       ARLNPDVPADLDAAVVRALDRDPALRFQTAAEFERALAQCVLTH--
86158893        SAWNDEVPPELDALVLAALEHEPARRTASAGELATALSRVLL----
108759111       TAFDPSVPTALELVCLTALAREPAARFDSLEDF-------------
108763617       RQVRSDIPVALDSIIQRALALQKDSRFESAHALGD-----------
108762650       RGINPDVPEALEAVATRALSRDVSQRFTNCREMARAIEAALGSELF
108762968       SRTNKHVPPELDAIVMRALSKRREDRYASTLEFARAIERAV-----
108762958       SELRPDVDAALDAVVRRALTARPDDRYMRSDELARALNTELV----
                      :   *:     **      *      :
```

Cluster No. 58 multiple sequence alignment

```
CLUSTAL 2.0.10 multiple sequence alignment


17131775        YRLDEELYNGSRTLVYRGYREIDSLPVVVKLLKNPYPSFNELIHFRNQYTITKNLHYPGI
17132825        YFILEEIYHGSKTVVYRAVREADQQPVVIKLLKREYPTFSELLQFRNQYAIAKNLKIPGI
113476169       YHITEIIYQGTRTEVYRGTRNSDNKPVIIKVLANVNPKFNELVQFHNQYMLSRHLQHPNI
17129668        YRILELTHSGTNTNIYRATKVDGNTPTILKVLIDNYFSLEAIVRFKHEYSISTNLDHPNI
                * : *  : *:.* :**. :  .. *.::*:*     .:. :::*:::* :: :*. *.*

17131775        IQTYSLEPYKNGYALVMEDFGGVSLKDYFG------KDHHVASLQEFLEIAIALCNTLDV
17132825        VNLYSLEPYRNSYAMVMEDFGGISLRDYA--------QQQSLSLTEILTITIQLADILHH
113476169       LQPLALERYGNGYALVMPDNGAIALSSYWHGGLKSVDTELEQNLGEFLRIAIQLTESLHY
17129668        VKVISLETHHKRLVLVFEDFGGISLKQYLY--------THQPSLQLTLQVAIAITRALVH
                ::  :** : :  .:*: * *.::* .*              .*   * ::* :   *

17131775        LYRECIIHKDIKPANILIN---------PETKQ---IKLIDFSIASLLPRETQIVINKNI
17132825        IYQQRIIHKDIKPANILIN---------PETKQ---VKLIDFSIASLLPRETQNIISPNI
113476169       LNQKRIIHKDIKPANILIY---------PKTQQ---IQLIDFNIASLLPKAQQEVTHPNV
17129668        IHDNKIIHKDIKPGNIIIKNLDNELQKTPEVEPSLIIKLTDFSIASRLKKETPQLINPNQ
                :  : ********.**:*          *:.:    ::* **.*** * :    :   *

17131775        LEGTLAYISPEQTGRMNRGIDYRTDFYSLGVTFYELLTAELPFVSRDPMELIHSHIAKLP
17132825        LEGTLAYLSPEQTGRMNRGIDYRSDFYSLGVSLFELLTGELPFTTDDPMELVHCHIAKQP
113476169       LQGTLAYISPEQTGRMNRGIDYRVDFYSLGVTFFELLTGELPFTTDDPMELVHCHIAKLP
17129668        LEGTLAYMSPEQTGRMNRNLDYRSDFYSLGITLYEMLTGQLPFNSSEPLELVHAHIAKEA
                *:*****:**********.:*** *****:::*:**.:*** : :*:**:*.**** .

17131775        EKLRS---------------------GREREIPKVICDIVMKLMAKNAENRYQSALGLKF
17132825        DKFTIPFSCQRRYANRFSNGGESTKLPNEETIPQVVGEIVMKLMAKNAEDRYQNALGLKY
113476169       -IFENK---------------ELRNGVCIPQIPQMVINIVMKLMGKNPEDRYQSALGLKY
17129668        TPIQQL----------------------AANIPNAVVGIVHKLMAKNAEDRYYSAQGLLV
                  :                            **: :  ** ***.**.*:** .* **

17131775        DLENCLNQL
17132825        D--------
113476169       DLENCLQQW
17129668        DLEQCLEQL
                *
```

# Cluster No. 59 multiple sequence alignment

```
CLUSTAL 2.0.10 multiple sequence alignment


119900177       FEIIGLVGEGGFGIVYLAQDHSLERKVALKEYMPASLASRTAAATVAVRSERHRETFEIG
120610153       YRLNDVVGEGGFGIVYKARDLSLDRIVAIKEYMPATLAGRKDGNEVHVRS-QHRGAFDAG
124266686       MEIVRVLAVGGFGIVYLARDHALDRDVAIKEFLPSHLVCRGEGCEVTVRSLTHATTFALA
120612691       FEVVSLLGVGGFGMVYKAFDHSLLRFVAIKEYMPTALAARAHGQSLWVRSSSHEQSFQAG
121593051       FEIVALLGVGGFGMVYQAFDHSLLRFVAIKEYMPAALAGRADGHSLWVRSSSDEQSFQAG
                 .:  ::. ****:** * * :* * **:**::*: *. *  .  : ***  .  :*  .

119900177       RRSFVNEARLLAQFDHPALVKVYRFWEANGTAYMAMPYYDGRTLREVLQ-QRPVPDESWI
120610153       LRSFINEARLLAKFSHPALVHVYRFFEANGTAYMVMQYYEGQTFRSFLA-QQHTVDEAWL
124266686       LESFVDEAKLLARFSHPAMVKVHSSWKANGTAYMAMPYLRGPTLWELRRSMTQAPTEIWL
120612691       LASFVAEARLLAQFDHPSLVKVFRFWEANQTAYMVMPLYAGMTLKQARAHMRTPPPEAWL
121593051       LASFVDEARLLAQFDHPSLVKVFRFWEANHTAYMVMPLYSGLTLKQARAHMRTPPPEEWL
                  **: **:***:*.**::*:*.  ::** ****.*    * *: .          * *:

119900177       RKVLAPVIDALELIHRENCFHRDVAPDNIMLIGDDRPVLLDFGAARRVIGDMTQALTVIL
120610153       SAVLVPILDVLEMLHAADCYHRDIAPDNIFLQESGMPVLLDFGAARRIIGDMTQALTMVL
124266686       RSIVDPLLEALQLLHAERIYHRDVAPDNVILTGAGLPVLLDFGAARRLIGDRTQLLTAVV
120612691       RKVLWSVLGALRVLHEGQTLHRDISPDNIFLQDSGLPVLLDLGAARHAITDHSHRLTAVL
121593051       RKVLWSVTSALRVLHEGQTLHRDISPDNIFLQDHGPPVLLDLGAARHAISDRDQKHTAVL
                  :: .:  .*.::*     ***::***::*   . *****:****: * *  :  * ::

119900177       KPGYAPIEQYAEMPG-MQQGPWTDVYALAAVIYFMITGKTPPPSVGRMMQDSYQPLATLA
120610153       KPGFAPIEQYVDDGA-MPQGAWTDIYQLGAVLYQAITGRPPATSVARMINDPLARLTPEN
124266686       KPHYAPIEQYAEATR-LRQGPWTDLYALSALVTFLLEGKPPPASTARSIHDDMHRLAERP
120612691       KVNYAPIEQYNDEGNDLRQGPWSDLYSVGAVVHGCLCNDTPLPATLRAIRDRMVPFSRVA
121593051       KVNYAPIEQYTDAASHLHQGPWSDLYSLGAVVHGCLCNDTPLPATLRAIRDRMVSFSRVA
                *  :****** :    : **.*:*:* :.*::   : . .* .:. * :.*    ::

119900177       AGR-------YGDAFLRGVDRCLAVKAEDRPQNMAEMREAL
120610153       CPG-------FSARFLHGVQSALAVKPQDRPQGIAELRQLL
124266686       FPG-------LSHGFLSAIDWGLSVRPEDRPQDVATF----
120612691       KTVKRQFGVEYSPPFVAAVSQTLALQPQDRPQSIDDFLRTL
121593051       RTVRKQFGVEYSRPFVDAVSQCLALQPQDRPQSIDAFLQTM
                           .  *: .:.  *:::.:****.:  :
```

Cluster No. 60 multiple sequence alignment

```
CLUSTAL 2.0.10 multiple sequence alignment


17133654        YQLQELIGTGAMGRVYRAKDVLLGGVPVAVKFLALSMQNEKMRLQERFEREAKTCALLGQ
22297988        YQLTELIGKGSMGRVYRAEDILLGGVPVAVKFLSQTLLND--RMKTRFAQEARAGALLGQ
86604933        YRLTHLLGQGGMGRVYLAQDLLFGGVEVAVKLLSQPAMDE--QARLRFEREAKACAALGQ
17132301        YQLQKLIGIGGMGEVFLATDILLGGAPVAIKFLTQTVCDP--KIQKDFAREALMSAALSQ
17132302        YLIIDLIGKGGMGRVYLAEDAAKGGKKVALKILMLNLVNQ--HISQRFAREIFIGAQLGR
                * : .*:* *.**.*: * *   **  **:*:*     :   :    * :*    * *.:

17133654        KSIHIVRVMDYGVDENKIPFYVMEYLQGQSLNQIIRQQNISLSRFVSMARQICLGLQCAH
22297988        KSMHVVRVLDYGMNNEEIPFYVMEFLEGENLSDLLLEEPLPLSRFLRIARHMCLGLQVAH
86604933        KSLHIVKVSDYGITADGVPFYVMEYLNGQTLKDILATGSLPLERFFRLARQIGLGLKAAH
17132301        KSLHIVRAYDYGVSDTGKPFYVMEYLNGKSLK---DLIPLPLNQFVHLTRQICLGLQCAH
17132302        KSKNIVRVLSYGVTDEKTPFYVMEYLQGKNLKQILKLKPLTIEKFLDICYQICVGLKCAH
                ** ::*:. .**:     ******:*:*:.*.       :.:.:*. :  :: :**: **

17133654        NGIPFDGTVYPIIHRDIKPSNILVIQDPSFGELVKVLDFGIAKLLQSNSD--QTKFYLGT
22297988        EGIIIEGQKCPIIHRDIKPSNVLVIQDGTMGELAKLLDFGIAKFLGDVPEKGQTSSFMGT
86604933        EGILLEGQRIQVIHRDLKPANVIVLADESLGELAKLVDFGIAKLLNTRESLSLTHAYLGT
17132301        QGIQIEGKVYPLVHRDIKPANILVIPDPILGQLVKILDFGIAKFLNHTVTLSTNRGFHGT
17132302        QGILLKGEIYPIVHRDIKPENIFITENNKQNENVKILDFGIAKFLTERSGMTLTDSFIGS
                :** :.*    ::***:** *:::  :   .: .*::******:*        .  : *:

17133654        LAYSSPEQMEGKE-LNNRSDIYSLGVMMFEMLTGKMPLVAPTHSFGSWYKTHHHQPPRTF
22297988        LAYCSPEQIEGRE-LDHRSDIYSLGITMYELLTGKMPIQAESHSIGSWFKAHHFQKPIPF
86604933        LAYSSPEQLEGLP-LDARSDIYSFGIMLYQMVSGQMPLQPTTESFPGWYQAHHKQRPLPL
17132301        LPYCSPEQLDGEK-LDGRSDIYSLGVIMFEMLTGAKPWQPETDLFGAWYKAHNFEQPRAI
17132302        LPYCSPEHMEGRKLLDVRSDIYSLGVLMFEMLTAKHPFQTQSNSFGNWYQAHRFQTPPTL
                *.*.***:::*   *: ******:*: ::::::.  *  . :. :  *:::*. : * .:

17133654        SEVAPDLAIPKEIENLVMNCLAKSAKDRPSSISEILQVLESV
22297988        NVASPGLHLPPALEELIMACMAKSPSDRPQNVAEIIKVL---
86604933        EELGLPLALPAGLSDLILSCLAKDPAARPPTVANVLAEL---
17132301        SEVKPQLKIPQQLNDLIMACLEKKASDRPQNVGEILQII---
17132302        GEVNNQLKIPEELQDLVICCLAKEVGDRPQNVQEIIQVL---
                      * :*  :.:*:: *: *.   ** .: :::  :
```

Cluster No. 61 multiple sequence alignment

CLUSTAL 2.0.10 multiple sequence alignment

```
2131007         YRLRRLVGRGGMGDVYEAEDTVRERIVALKLMSETLSSDPVFRTRMQREARTAGRLQEPH
13881430        YRLRRLVGRGGMGDVYEAEDTVRERIVALKLMSETLSSDPVFRTRMQREARTAGRLQEPH
31792931        YRLRRLVGRGGMGDVYEAEDTVRERIVALKLMSETLSSDPDFRTRMQREARTAGRLQEPH
145225785       YELHSVIGVGGMGEVYRAYDTARERMVAIKLLRPEMAADHSFQERFRRESRVAARLQEPH
41408129        YELRSLIGTGTLGEVYRAYDTVKDRLVALKLLRGELDAG--FRQRLWRDCRAVTRLQEPH
41408124        YEIRRLIGKGGMGEVYEAYDTKKGRAVALKLLTDNYADDEKFRERFLRESRAAAILQEPH
120402048       YRLQKLIGRGGMGEVYQAYDTKTDRVVALKVLPHSMAQDETFQARFRRESQAAAGINDPH
145225811       YRLQKLIGQGGMGEVYQAYDTKTDRVVALKVLPHHMAQDETFQARFRRESQAAAGINDPH
134097137       YKIDAVLGRGGMSVMYRATDTRLGRKVALKVMGEHITGDAEFRERFVDEARNTSAIDHAN
29827698        YRIEREIARGGMAVVYRARDLRLDRTVALKLLAPELALNDTFRRRFTHESRVAAAIDHPN
108758719       YHLDKLVGSGGMGEVHKATQLSLGRTVAVKLLNPELAKDPSFIARFQKEAAALAALSHPH
                *.:   :. * :. ::.* :    * **:*::      .  *  *: :.      :...:

2131007         VVPIHDFGEIDGQLYVDMRLI-NGVDLAAMLRRQ-GPLAPPRAVAIVRQIGSALDAAHAA
13881430        VVPIHDFGEIDGQLYVDMRLI-NGVDLAAMLRRQ-GPLAPPRAVAIVRQIGSALDAAHAA
31792931        VVPIHDFGEIDGQLYVDMRLI-NGVDLAAMLRRQ-GPLAPPRAVAIVRQIGSALDAAHAA
145225785       VIPVHNFGEIDGVLYIDMRLV-EGASLKEVLRSD-GPLQPARAVSIIRQVAAALDDAHAN
41408129        VLPLHDFGEMDGVPFIDMQLVDDGGSLKELLREQ-GGLEPSRAASITGQVARALDAAHAA
41408124        VIPIHDWGEINGVLYIDMRLV-QGQTLHEMLKT--GSLEPRRATDIIRQVASALDAAHAA
120402048       VVPIHGYGEIDGRLYLDMRLI-EGRNLGTMLQETEKPLGAAFAVTVVEQVANGLDAAHKL
145225811       VVPIHGFGEIDGRLYLDMRLI-EGRNLGTMLQETDKPLGAAFAVMIVDQVANGLDAAHRL
134097137       IVPLYDFGEVDGMLYIAMRLV-DGSDLASKIK--DGPISAQRTLELLGQVAEALDMLHER
29827698        IVPVFEAGETEGVLYIAMRFV-SGRDLRHLLDR-EGPLPFSAGVRIALQVASALDAAHDH
108758719       IVSIVDKGKTDTTYYLVMEFV-DGPSLRELIREP--QLDVMGALRRMLQICRAIEYAHGR
                ::.:   *: :   :: *.:: .*  *   :      :          *:  .::  *

2131007         GATHRDVKPENILVSA------DDFAYLVDFGIASATTDEK------LTQLGNTVGTLYY
13881430        GATHRDVKPENILVSA------DDFAYLVDFGIASATTDEK------LTQLGNTVGTLYY
31792931        GATHRDVKPENILVSA------DDFAYLVDFGIASATTDEK------LTQLGNTVGTLYY
145225785       GLVHRDIKPENVLLTG------DDFAYLVDFGIAHGGGEAS------VTSTGLVVGSSAY
41408129        GLMHLDVKPENILLTH------DHFTYLADFGLAQAAGDDK------LSRT--------Y
41408124        GLIHRDVKPQNIIVTP------DDFAYLVDFGIAEARGDTH------LTMAGHTVGTFDY
120402048       DLIHRDIKPSNILITG------RDFVYLIDFGLARTAGEKG------LTTAGSTLGTLAY
145225811       GLIHRDIKPSNILITD------KDFVYLIDFGLVRTAGEQG------LTTAGSTLGTLAY
134097137       NLVHLDLKPANVLVTSRESS--SEHVYLADFGLTRRGATGH------RTSSGDFLGSPTY
29827698        GLVHRDVKPGNILVARGTDSDHPEHVYLTDFGLTKRSLTVTG-----FTIPGQFVGTLDY
108758719       GVIHRDLKPENILLDQQ----AGGIAKVSDFGLASFLEDASPSSRYALTSTHVSMGTLSY
                .  * *:** *:::           . : ***:.              :          *

2131007         MAPER-FSESHATYRADIYALTCVLYECLTGSPPYQGDQLS-VMGAHINQAIPRPSTVRP
13881430        MAPER-FSESHATYRADIYALTCVLYECLTGSPPYQGDQLS-VMGAHINQAIPRPSTVRP
31792931        MAPER-FSESHATYRADIYALTCVLYECLTGSPPYQGDQLS-VMGAHINQAIPRPSTVRP
145225785       MAPER-FSGERGGPASDIYSLACLLYESLTGRAPFEAADVRQVWSAHMFAAPPRPSIMRR
41408129        MAPER-FTTGSLGPQTDIYSLACVLYECLTGQPPFEGADPGELRSAHLLSPAPRPSIMRR
41408124        MAPER-FGDEETTSAVDVYALACVLYEALTGAKPFPVHSAEQAIRAHLSSPPPRPSAVNP
120402048       MAPER-FEGGEVDARSDIYALTCVLYECLTGSRPYPADSLEQQIAGHMVSEIPRPSDTDP
145225811       MAPER-FEGGEVDARSDIYALTCVLYECLTGARPYPAESLEQQIAGHMVSPIPRPSDVDP
134097137       AAPEH-LRGEPVDGRTDLYALACMLFACLTGRPPFQG-QVQEVIQGHLHGEPPKVTS-LV
29827698        VAPEQ-ICGKPVDGRCDVYGFGCVVYEILAGTPPFCRDDEMALLWAHQHDEPPRLSRRRP
108758719       MAPEQRVDAKSADARADIFSLGVILYEWLTGEVPLGTFDPP--------------SRRKP
                 ***: .         *::.:  :::  *:*  *    .                :

2131007         GIPVAFDAVIARGMAKNPEDRYVTCGDLSAAAHAALATADQDRATDILRRSQV
13881430        GIPVAFDAVIARGMAKNPEDRYVTCGDLSAAAHAALATADQDRATDILRRSQV
```

```
31792931        GIPVAFDAVIARGMAKNPEDRYVTCGDLSAAAHAALATADQDRATDILRRSQV
145225785       GVSRAFDDVIARGMAKNPTERYATAGELARAAAAAASAAPVA-----------
41408129        GVGRAFDDIITRGMAKQRSARFGSAGELARAASEAV-----------------
41408124        HVPASFDDVIARGMAKHPDDRYGSAGALGRAAKRAL-----------------
120402048       RL-AAFDEVIAKGMAKKPDKRYQSAGELAQAAKRALNAPVR------------
145225811       RL-AAFDEVIAKGMAKKPAKRYQSAGELAEAAKRAL-----------------
134097137       VLPADIDDVLRRGMAKKADQRYSTCKGLITAAKAAL-----------------
29827698        DLGPQVDEVMAKALAKSPEDRYDSCLAFV------------------------
108758719       GLDSRLDAIVTRCLKPDPEDRYPSVTTLIADL---------------------
                 :   .* :: : :      *: :   :
```

Cluster No. 62 multiple sequence alignment

```
CLUSTAL 2.0.10 multiple sequence alignment


108757504       YQLLSRLATGGMAQIYLARPQGAE-PDKRVVVKRILPHLAENDDFVKMFLDEARIAARLN
108763056       YQLLKKLATGGMAEVWLARQTGIEGFHKNLVVKRILPHLAEDREFVEMFRNEALIAARFN
108758235       YELLRKIASGGMGQVFLAREHG-TGFERLVVLKLILPHLAEDDEFLSMFLDEAGLVARLT
108762112       YELLRKLAIGGMGAVYLARQKGPVGFQKLLVVKRLLPHLSEDDEFIDMFLDEGRIAAHLN
108760861       YRLVQQLATGGMAQLYLASIDGPDGFSKSCVIKRVLPEYASLESFSRMFADEAKVAALLT
108761425       YEVVTQLSVGGMAELFLGFTSGPGGFRKYVVIKRVLPDARDNAQFERMFLDEARITAAFN
108760985       LNIIRRLGQGGMAEVFLAKQVGVKGFEKFVVMKKILPQFAENPEFVDMLFAEARANARLT
108763014       YEVVTPLGTGGMAQVLVARTRGPEGLGRLVALKRILPHLSSDPSIVQQFLDEARIGLRLS
108759758       YRLIDRIAVGGMAEIFLAHQQQEDGRESPVVIKRIRPHLSKHTAFVKMFLNEARLAAQLN
108763631       YLLIKRLAVGGMAELFLSQRPPD---PELVVLKRILPYLSEEPEFVQMFLDEARIAAQLH
108760907       YQLVRKLASGGMAEVFLAKAAGPRGFEKTLVLKRILPHLAEDAAFVEMFLGEARLAAQLE
108761736       YVLVRKLAEGGMAEIFLAKLLGADGFERNVVIKRMLPHLTNNPDFVEMFRDEARLAAKLA
108759765       YVLLSKIAAGGMAVTYRARMTGAAGVTKPCVIKQILPHFVDDADFVEMFIGEARVVASMS
108762187       YTLLSQLATGGMGEIYLARLEGAQGFEKLCVIKKILPQLAADTDFVERFVGEARTLVRLS
108761001       YLLLDRINIGGMAEVWRGKQFGASGFERLVAIKRILPNIAEDDEFISMFIDEAKISVQLT
                  ::  :  ***.    .            .:* : *       :   :  *.     :

108757504       HPNVVQIFDLGAQDDSFFIAMEYIHGDDLRRLWRQSELMGQP-LPVPLVCRILIEACAGL
108763056       HPNIAQVYEFGEANGTYYIAMEFIHGEDLGRVMRKAASTGQW-VARPLAIRIVAAACEGL
108758235       HPNLITILDLTEIEGRHCLAMEYVQGDDVRRLEKTSRAQGKA-LPVGLILRIIADAAAGL
108762112       HPNIAQIYDLGDVDGQYFIAMEYVHGEAVGPLGARAQQHGIT-IPLGLKCRIIADAAAGL
108760861       HPNIVQVFDFGRVDGQYYLAMEWIQGHSLDRLLRQALRSGRV-VGTRVAVDVGLAVADAL
108761425       HPNIAQVFDLGREDDGLYLAMEFIAGQNLNQITGACLRRQEQ-LPLGFTLSVARDVCMAL
108760985       HPNVVQTFDVGVSDGVAYILMEYVRGPDLKKLVIELRRKGLA-LPLEHALRIVADVAAGL
108763014       HPNLVHFYDFGEAEGAYYIAMELVRGVDLERLLRAAKG---P-LETAHAVAMVCQGLAGL
108759758       HPNVVQIHDLGKIADSYFIAMEYVSGRDMRRVVPKAEALGIP-FPLVYAVKIASCVCAGL
108763631       HPNIVQVHELGKEGDNIFIAMEYVEGVDLRRVMAEESKFGAT-VPYGVAARICAQVAAGL
108760907       HPNIVQIFDFGEAEGSFFLAMEFIDGPNLRKLVKRAAEEA---LPPAFCAKVVAAAAEGL
108761736       HPNIVQIQELGFAEGCYYICMEYLAGEDFSTTLRLAGRKRHY-VPLPVVLRVLIDAARGL
108759765       HSNIAQIFDFGEVDGQYFIAMELVQGQPLSKVLRRAQRMGMASFPEPLALHVASKLCDGL
108762187       HGSIAQVLDMGLHEDEAYMALEHVDGKDLRKVAARVRDRQMP-LPVTFILYTMGRVLDAL
108761001       HANVASIYELGNILGSYFISMEYIPGKDMRAIFDRCRKKGEP-APVPLVAFCVSKMCEGL
                * .:    :.    .   : :* : *  .                             .*

108757504       DYAHKRTDPTTGRPLGIVHRDVSPQNILVTFEGGVKVVDFGIAKAADQATVTR-SGVLKG
108763056       HYAHSRTD-DAGRPLRVVHRDISPQNILISFDGSVKLVDFGIAKAADQASLTK-SGAIKG
108758235       DYAHQARD-AQGKPLRLVHRDVSPQNILVGFDGGVKVIDFGVAKAATSSQNTA-TGVLKG
108762112       DAAHNARS-PSGRKLALIHRDVSPQNVLVGFNGGVKLIDFGVAKASGKLSQTI-VGTIKG
108760861       AYAHAKTL-PDGTPLMLVHRDVTPGNVLVSRDGIIKLADFGIVKSAVNAERTV-AGVVKG
108761425       HYAHTYTA-PSGAPSPVIHRDVAQKNIMVTYDGVVKLLDFGIAKAKDSLERTN-VGTVKG
108760985       HYAHAYVD-PAGTPHPVVHRDVSPHNVLISLDGAIKLSDFGIAKVAG-EEHTQ-AGVLKG
108763014       HAAHVLQG-EDGAPLELVHRDLSPHNLMVGFDGRVKVLDFGVAKARAQRTVTL-PGIVKG
108759758       HHAHTKGD-LYGNPLNIVHRDVSPENIVVAFDGSVKILDFGIAKAANQMEQTR-NGEIKG
108763631       DYAHHSRG-VDGRPLELIHRDVSPQNVMIGYDGRVKLVDFGIAKAGAFMERSK-PGVIKG
108760907       AYAHEFRDVETGEPLGLIHRDVSPDNILVSRQGAVKVVDFGIAKVAGQGHRTL-TGVVKG
108761736       HFAHEFTN-EAGQPLNVVHRDISPSNLYLTYQGQVKVLDFGIAKAESRLVNTR-TGVVKG
108759765       DYAHRHVG-EDGLALGLVHRDVSPDNVLISYEGEVKVIDFGIAKVTSAVEAKTSPGTLKG
108762187       AYAHRKKD-DDGEDLKLVHRDISPQNILISYEGEVKVIDFGLAKSR-LSAAKTNPSIILG
108761001       DYAHRKKD-GMGREMNIVHRDISPQNVLISYEGEVKVIDFGIAKAA-GKATKTQAGILKG
                  **       *    ::***::  *: :  :* :*: ***:.*       .   . : *

108757504       KYSYMSPEQAA-GMRVDCRADIFALGIVLYELLTSTRLFKRPNDIQTLTAVSECRVLPPS
108763056       KFAYMAPEQAA-GKPLDGRADIFAIGLVLYELLTGVRPLKRDSELATLQAAMECAIAAPS
108758235       KYPYMSPEQAS-GLAIDARSDLFALGVVMWELLTGKRLFKGESDMMTLRLVKDCQVPRPS
```

```
108762112       KHAYMSPEQAR-GEPLDSRSDVFGLGTVFYELLTQQRLFKRETELATLKAVVGTKIVPPS
108760861       KYPYMSPEQIT-SQELDHRSDLYSLGIVLYEAATGRRLFKRDTLEATIMAASQANVPPPS
108761425       TTGYMSPEQVR-GDTLDGRSDLFSVGVMMHELITGARLFAGKNERDEMMKILEAPVPWPS
108760985       KISYISPEAAS-GRTLDARNDVFALGVVLFELLTGSLPFRRDHDAATLQAIVRDPAPVPS
108763014       KPLYMSPEQAR-GMRLDARSDLFAMGLVLYQSLIGARAFEKPEELATMHAICDEALPPRP
108759758       KLSYMSPEQCL-GKPLDCRSDVFSLGVVLYEWLTGFKLFTGESEAAVMRSITDGKIYAPS
108763631       KFLYLAPEQVS-QERLDHRADIFALGTMLYEITTGRQPFAKPTTEGILYAIRYEDPSPPH
108760907       KVAYMPPEQLQ-AKAMDRRVDVYALGVVLYELLTGKRPFDATTDVSVMQAILFESFIPVS
108761736       KYMYMAPEQAR-GKEVDRRADIFALGVSLYEALTHVRPFSRENDLAVLNALLQGELKPPR
108759765       KYPYFSPEQAQGRQDLDARTDVYAAGVVLYEMVCGKRPYEGEFVTVLPRILVADRLPP-S
108762187       KFLYMSPEQAR-HQPVDRRSDLYAVGLCLYELICGKNPFDGVHPGELMSLVANPRIAPLD
108761001       KFGYMSPEQIR-GLPLDRRSDVFAIGVCLYEMLTGERLFVGDSDFSVLEKVRKAEVPSPT
                .  *:.**       :* * *::. *  : :

108757504       HVTSRVPADLDAIVLKALAKELPDRYQEAAHLQHAL-----
108763056       QVAD-VPEEMDPVVMRAIAKNSDDRYRDARQFQMALEEILV
108758235       QLNPRLPPGLDEVVLKALAPSPDQRYPDCGAFRLA------
108762112       EVVPEIPKALDAIVFKALARKRDERFSTAGELQLAL-----
108760861       EGTPDFPAELERIILRLLQKAPSARYQSARELRDDL-----
108761425       HVAPHVPEEVSKVVMRALERSREKRFANGRDMARAI-----
108760985       QLKPNIPQDVSDLVLRALVKDPARRTPSAAALREEI-----
108763014       ---SHISRALWDVLETALAKRPEARFGSAREMADRL-----
108759758       YFREDLPERLETILMRALERDRDKRYQTAAQMQKDL-----
108763631       LIRDDYPPALSRIVMRCLTKDRALRYQRASEVHDALESF--
108760907       ARRPDVPVALQQVLDKALAKDRERRYADCRALQDDL-----
108761736       ELRPDLPEELEAILLKAMAFKPEDRYPTAEAFADALETF--
108759765       ALNPTVSEDMETVISHAMALDREARYQTAKDLSESL-----
108762187       QVEPLTPPAVTALVAKALAVDPSQRFQTAEEFRGRL-----
108761001       TYNRRIPETLERIVLKALAKDVDERYQYASELGDDL-----
                      .  :  ::   :      *      .
```

Cluster No. 63 multiple sequence alignment

```
CLUSTAL 2.0.10 multiple sequence alignment


119900250       YRIERQLSLGGFSIVYLAYDDDDGTAVAIKEYLPNSLALRKEGQIEPLVPDENLPAFRYG
71909460        YIIDRQLSLGGFSIVYLATDPE-GRQVAIKEYLPNSLALRSKGEIKPVITAEHLGAFRYG
91774401        YEIKKLLSAGGFSFVYIARDLENNTTVAIKEYLPNMLALRKEGDQVRIPTNEAAAGFRFG
113866975       YRIVKKLASGGFSFVYLATDDT-GAPVAIKEYLPASLARRNPGELIPVVPEENAAAFRLG
17429176        YRIVKKLASGGFSFVYLATDEH-GTPVAVKEYLPSSLARRSPGELIPVVPEESASAFRLG
120612228       YRVVRRLSSGGFGVVYLAVDAE-GQQVAIKEYLPSSLATRSPGELLPKVPPEKLSLYRLG
121593345       YRVVRRLSSGGFGVVYLAIDAE-GQQVAIKEYLPASLATRAPGELLPKVPPEKLSLYRLG
121611074       YRVVRRLSSGGFGVVYLALDPE-GQQVAIKEYLPAALATRAPGELLPKVPSDKLSLYRLG
121603724       YRVVRKLSAGGFGVVYLAVDNE-GQQFAIKEYLPSSLASRAVGELLPQVLPEKLSLYRLG
91787233        YRVVRKLSAGGFGVVYLAVDNE-GQQVAIKEYLPSSLATRSPGELLPQVQPEKLSLYRLG
89900417        YRVVRKVAAGGFGVVYLAEDSE-GQQVAIKEYLPSALATRVAGELLPQVQPERLSLYRLG
124267892       YQVIKKLAAGGFGVVYLCEDSE-RRLIALKEYLPASLAERSVGELTPRVKPEKQPLYRLG
134095313       YRIVKKIASGGFSIVYLAYDED-GNAVAIKEYLPSSLALRQPGELVPAISPENLPVFRIG
34499304        ---------GGFSVVYLALDDD-DRKFAIKEYLPRNLAERKDDGGVTVPHDLDRDAFNLG
                         ***..**:. *      .*:*****  ** *  .             :. *

119900250       MKCFFEEGRSLAKLMHPNVVRVLNFFRANNTVYMVMQFERGRTLHDYIQKHRG-----DV
71909460        MKCFFEEGRALARLSHPNVIRVLNFFRANDTVYMVMEYEHGRTLQEFIQKHQG-----HI
91774401        LKCFFEEGRALANIEHKNIVRVQNFFRANDTVYMVMRYERGKSLQEYVLARDT-----PL
113866975       LKYFFEEGRSLARISHPSVVRVVNFFRENSTVYMVMNYELGKTLQEHVLAARQQGGPKVL
17429176        LKYFFEEGRSLAKISHPSIVRVLNFFRENGTVYMVMTYEQGKTLQEHVLGARQQGKLKVL
120612228       LKSFFEEGRALAQISHASVVSVLNFFRENETVYMVMNYLEGATLQDFIITARDLKTQKVF
121593345       LKSFFEEGRALAQISHASVVSVLNFFRENETVYMVMNYLEGATLQDFIITARDLKTQKVF
121611074       LKSFFEEGRALAQISHASVVSVLNFFRENETVYMVMNYLEGATLQDFIVTARDLKTQKVF
121603724       LKSFFEEGRSLAQISHGSVVSVLNFFRENETVYMVMNYLEGGTLQDFIITARDLKKPKVF
91787233        LKSFFEEGRALAQIAHGSVVSVLNFFRENETVYMVMNYLEGGTLQDFIITARELKKQKVF
89900417        LKSFFEEGRSLAQISHPSVVSVLNFFRENETVYMVMNYLEGASLQDFIVTARDLKQAKVF
124267892       LKSFFEEGRSLAQISHPSVVSVLNFFRENETVYMVMNYLQGDTLQDFIVTARDLKRDKVF
134095313       LKCFFEEGRALARIAHPNVVSVTNFFRANETVYMVMGYESGRSLQDHILRRRDKGEKPLV
34499304        LKCFFEEGRVLSGISHPAVVRVSNFFRANQTVYMVMEYADGRSLGRELELAGG-----RM
                :* ****** *: : *  :: * **** * ****** :  * :*   :           .

119900250       GERFIRGVFTRMLNGLREVHAHKLLHLDIKPSNIYLRTDGTPVLLDFGAARQTLALDQPM
71909460        SERFIRGVFTRLLNGLREVHSNKLLHLDLKPSNIYLRGDNTPVLIDFGAARQTLVTDQPI
91774401        PETMLRSVFGQLLNGLREVHAQKLLHLDIKPANIYIRLDGSPVLLDFGSARMALNEASNA
113866975       REHFMRKVFHDLMSGLREVHIHKLLHLDIKPGNIYLREDESPILLDFGAARQTLTMEAAR
17429176        RERFIRQVFHDLMSGLREVHIHKLLHLDIKPGNIYLREDSSPILLDFGAARQTLTAEASR
120612228       RESTIRSLFDEVLRGLRIVHQHKMLHLDIKPANIFITDDNKAVMIDFGAAREVLSKEGNF
121593345       RESTIRSLFDEVLRGLRIVHQHKMLHLDIKPANIFITDDNRAVMIDFGAAREVLSKEGNF
121611074       RESTIRSLFDEVLRGLRIVHQHKMLHLDIKPANIFITDSNKAVMIDFGAAREVLSKEGNF
121603724       RESTIRSLFDEILRGLRIVHQHKMLHLDIKPANIFITDDNRAVMIDFGAAREVLSKEGNF
91787233        RESTIRSLFDEILRGLRIVHQHKMLHLDIKPANIFITDDNRAVMIDFGAAREVLSKEGNF
89900417        RESTIRSLFDEILRGLRIVHQHKMLHLDIKPANVFITDDNKSVLIDFGAAREVLSKEGNF
124267892       RESTIRSLFDEILRGLRIVHQHKMLHLDIKPANIFITNDNKAVMLDFGAAREVLSKEGNF
134095313       SERFIRKMFSHVMNGLREVHTNKLLHLDLKPANIYLRVDGTPILLDFGAARQTLKADLPK
34499304        EERLIRKWFAALLSGLREVHSRRLLHLDIKPANIYLRRNGVPLLLDFGASRQTLARQDKH
                 *  :*  *  :: *** ** .::****:**.*:::  .  .:::***::* .*

119900250       LKPMYTPGFASPEQLGQREALGPWSDIYSVGASIYACIVGAAPPRSDERLKHDTIMPISR
71909460        LKPMYTPGFASPEHYGNRKDLGPWSDIYSVGASMYACLAGVAPQAADARMKKDTLPPAMM
91774401        LPPSYTPGFASPEQYGDRKQLGPWSDVYSIGASMYACLLQAAPQAADQRVKKDQLVPAAR
113866975       FQPMYTPGFAAPELYGKHSDLGPWTDVYSLGATLYACMAGMPPQEANQREKEDRMGDALA
17429176        FQPMYTPGFAAPELYRKHNELGPWTDIYSIGATIYACMAGAPPQEATQREKEDKLGENLE
```

```
120612228       IRPMYTPGFAAPEMYRRDSSMGPWTDIYAIGACIYACMQGFPPNEAPQRAEKDRLSLALS
121593345       IRPMYTPGFAAPEMYRRDSQMGPWTDIYAVGACIYACMQGFPPNEAPQRQEKDRLSLALN
121611074       IRPMYTPGFAAPEMYRRDSSMGPWTDIYAIGACIYASMQGFPPNEVPQRIEKDRLALALT
121603724       IRPMYTPGFAAPEMYRRDSSMGPWTDIYAIGACMYASMQGYPPNDAPQRLEKDRLSLALS
91787233        IRPMYTPGFAAPEMYRRDSSMGPWTDIYAIGACIYASMQGYPPNDAPQRLEKDRLSLALS
89900417        VRPMYTPGFAAPEMYRRDAAMGPWTDIYAIGACIYACMLGYPPNEAPQRLEKDRIAMALT
124267892       IRPMYTPGFAAPEMYRRDGTLGPWTDIYAIGACIYACMQGYPPNDAPQRIEKDRLGLSLS
134095313       LYPMYTPGFAPPELYVKNGNLGPWTDIYSIGASMFACMVGAPPQPADQRKLNDKMDGHFN
34499304        FAAMYTPGFAAPEQYDKSQPLGPWTDIYAIGACLYVCMGGATPQPSRERCEQDLLAPASQ
                . . ******.**       :***:*:*::** ::..:   .*     *  .* :

119900250       THAGRYSGQLLELIEWSLRLDPLARPQSVYTLQKALM
71909460        RWDGQYSDRLLEIIDWCLNLNHLYRPQSVFALQKALV
91774401        LGKGRYSPNLLEIIDNCMALDYMDRPPSVFALQKSLL
113866975       RLRSSYTNGLVDLVEWCLRLTPAERPQSV--------
17429176        RLRTVYTGSLIDLVAWCLKMKPEERPQSV--------
120612228       KLRGVYSDNLIEVVEWCMALDPLSRPQSV--------
121593345       KLRGVYSDNLIEVVEWCMALDPLSRPQSV--------
121611074       KLRGVYSDNLIEMVQWCMALDPLSRPQSV--------
121603724       RLRGVYSDNLIEVVEWCMSLDPLSRPQSV--------
91787233        RLRGVYSDNLIEVVEWCMSLDPLSRPQSV--------
89900417        RMRGVYSDNLIEIVEWCMSLDPLSRPQSV--------
124267892       RLRNVYSDNLIEVTEWCMSLDPLSRPQSVFALQKELA
134095313       KLEGVYSSDLIKVIRWSLMIDPLERPQSVFALQKAL-
34499304        VFRHRYSRELTGLCDRCLSLEPNLRPASVLEIQKSL-
                     *:  *  :   .: :    ** **
```

Cluster No. 64 multiple sequence alignment

```
CLUSTAL 2.0.10 multiple sequence alignment


145595768       YRVRSRVARGGVATVYTATDERLDRIVAVKIIHPASGPEAQGQTA---GFVERFADEAKT
50842217        YEIVSKIARGGMATVYRAQDLCLPRIVAVKVMH-------EGLDA---ELAERFDSEAKA
126435861       YRVDAPIATGGMSTVYRGLDVRLDRPVALKVMDP-----RYSGDQ---QFLTRFQREARA
145223559       YQVDTLIAAGGTSDVYRGLDLRLDRPVALKIMDS-----RYAGDE---HFMTRFQREARA
41408012        YLIESKIASGGTSTVYRGVDTRLDRPVAVKVMDP-----RYAGDD---QFLTRFQREARA
68535808        YRIGAQIARGGMSTVYAAIDTRLDREVAVKVMDP-----ALAREP---AFRTRFEREARA
134098292       YRVDSLIARGGMSAVYRGLDTRLDRPVALKVMDP-----QYSGDR---SFVARFELEARS
21220588        YRVEARIAVGGMATVYRAVDTRLDRVLALKVMHP-----TLATDA---TFVERFIREAKS
29832634        YRVDARIAVGGMATVYRAVDTRLDRVLALKVMHP-----TLAADG---SFVERFIREAKS
119026032       YRVVSRIADGGMATVYQAVDERLGRTVAIKIMHT-----QLAQGPQRDQFVERFHREARS
23326698        YRIVRNIAEGGMATVYEAIDERLGRTVAIKVMHT-----QLAKGPHREQFVERFRREANS
28493183        YLITEKIASGGMATVYKGKDIRLKRDVSIKIMHD-----HLVDDP---KFTEKFIAEAQL
119025056       YQLGQLVGRGGMAEVHVATDTRLGRTVAVKIMRA-----DFATDS---IFLERFRREAHS
23325797        YQLGQLIGRGGMAEVHVALDTRLGRTVAVKIMRA-----DLANDD---IFLARFRREAHA
21222231        YQLRDLLGEGGMASVHLAYDSVLDRQVAIKTLHT-----ELGREQ---AFRERFRREAQA
                * :   :. ** : *: . *  * * :::* :                 :  :*  **.

145595768       IARLTHPNVVAVYDQGIH---------ADRPYLVMEYVRGRTLRDVLTER----HRLNPD
50842217        AARLVNPHVVSVFDQGMD---------GDRPFIVMEYVPGCTLRHIITQE----APLTPV
126435861       VARLKDPGLVAVYDQGLD---------GQYPFLVMELVEGGTLRELLRER----GPMPPH
145223559       VARLKDPGLVAVYDQGLD---------GRHPFLVMELIEGGTLRELLRER----GPMPPH
41408012        VARLKDPGLVAVYDQGLD---------ARHPFLVMELIEGGTLRELLGER----GPMPPY
68535808        VAKLSDPSLVNVFDQGVD---------DDYVFLVMELVEGGSLRELLKER----GPMPPH
134098292       AAKLHHPDVVAVYDQGVDRE-----IDGDQVYLVMQLVEGCTLRDLMRDQ----GRLSLP
21220588        VARLDHPNVVQVFDQGAE---------GAYVYLAMEYIAGCTLRDVLRER----GALRPR
29832634        VARLAHPNVVQVFDQGAD---------GSYVYLAMEYVAGCTLRDVLRDR----GALQPR
119026032       AAAIANPHIVQVYDTGEF---------DGLDFLVMEYVHGVNLRYEMNQQ----VTFSVR
23326698        AASIANPHIVQVYDTGEF---------NGLDFLVMEYVHGVNLRHEMNAQ----GTFSVR
28493183        AAGISNANIVNVFDQGRE---------NRIAYMVMEYVPGITLRKLLREK----HVLTVK
119025056       VAQMNNPNIVNIYDSGEETVTTETGEIEHLPYLVMEYVKGQTLRDILKVN----GALSQR
23325797        VAQMNNPNIVNIYDSGEELVSSESGDAERLPYIVMEYVKGQTLRDIIKVN----GALSQR
21222231        VAKLTHTNIVSVFDTGEDDLDGMT-----TPYIVMEYVEGRPLGSVLDEDVRQQGAMPAD
                 * : .. :* ::* *               ::.*: : *  *   :         :

145595768       EALAITEQMLAAIAAAHRAGLVHRDVKPENVLVAEAPTGGAANLVDSVVKVADFGLAQAV
50842217        RALDLLEPVVSALASAHEDGLVHRDVKPENVLISDRGQ----------IKVADFGLARAV
126435861       AAAAVLAPVLSGLAVAHRAGLVHRDVKPENVLISDDGE----------VKIADFGLVRAV
145223559       AVAAVLRPVLGGLAAAHAAGLVHRDIKPENVLISDDGD----------VKIADFGLVRAV
41408012        AVAAVLRPVLGGLAAAHRAGLVHRDVKPENVLISDDGE----------VKIADFGLVRAV
68535808        AAIAVMKPVLTALSIAHAKGMIHRDIKPDNVLISDHHQ----------VKLADFGLVRAI
134098292       MALSVLEPMLSALGAAHRAAMVHRDIKPENVLIGLDGS----------VKVADFGLVRAA
21220588        AALDILEPVLAALGAAHRAGFVHRDMKPENVLIGDDGR----------VKVADFGLVRAV
29832634        AALDILEPVLAALGAAHRAGFVHRDMKPENVLIGDDGR----------VKVADFGLVRAV
119026032       ETLRIVGETLDGLASAHRAGVVHRDIKPENILLNDRGH----------VQITDFGLAKAV
23326698        ETLRVVAETLDGLASAHRAGVVHRDIKPENILINDRGH----------VQITDFGLAKAA
28493183        QTLEIITCVLEGLSSAHKSGLIHRDIKPENILIGNNGQ----------IKLGDFGLSRLA
119025056       DAEQVMMGVLNALEYSHRMGIIHRDIKPGNIMISEQGV----------VKVMDFGIARAI
23325797        DCEQVMLGVLNALDYSHRMGIIHRDIKPGNIMISEQGV----------VKVMDFGIARAL
21222231        KALKITADVLAALEISHEMGLVHRDIKPGNVMMTKRGV----------VKVMDFGIARAM
                    :    : .:   :*  ..:***:** *:::               ::: ***: :

145595768       E---ASADDAQANQ---LMATAAYVAPELVTDGHADPRTDVYSAGIVLFEMLTGRVPYDG
50842217        G---NHTMSATSGQ---LIGTVSYIPPERVTRGSSDERSDIYSAGIVLFEMLTGHKPHTG
```

```
126435861       ----AEAKITSTSV---ILGTAAYLSPEQVGTGNAGPRSDVYSVGVMAFELLTGVTPFNG
145223559       ----AEAKITSTSV---ILGTAAYLSPEQVATGETDSRGDVYSVGVMVYELLTGRTPFTG
41408012        ----AAAGITSASV---ILGTAAYLSPEQVRDGAATPRSDVYAAGIVAYELLTGRTPFTG
68535808        NNAMSDATNATTSVNGQVIGTVGYLSPEQVRGENLTQASDVYSAGILLFELLTGRTPFKG
134098292       ----ASAGTTSGSV---ILGTVAYLSPEQVTTGAADSRTDIYSAGVVLYEMLTGEPPYVG
21220588        ----DTVTSTTGAV----LGTVSYLAPEQIEHGTADPRVDVYACGVMLYEMLTGAKPHDG
29832634        ----DTVTNTTGSV----LGTVSYLAPEQIEHGTADTRVDVYACGVVLYEMLTGGKPHAG
119026032       ----SQATLSSTGM---LLGTAAYLAPEMIQHNQATPQGDLYSVGIMAWEMLAGKVPFTA
23326698        ----SQATLSSTGM---LLGTAAYLAPEMIENNQATAQGDLYSVGIMAWEMLTGKVPFDS
28493183        ----SNNTTTGTG----LLGTIAYLSPELITRSEADTRSDVYAIGIMLFELLTGQQPFHG
119025056       DD--SAATMTQSQG---VVGTAQYLSPEQARGESVDMRSDLYSAGCVLYEMLTGRPPFTG
23325797        DD--SAATMTQSQG---VVGTAQYLSPEQARGETVDMRSDLYSAGCVLYEMLTGRPPFTG
21222231        QS--GVTSMTQTGM---VVGTPQYLSPEQALGRGVDARSDLYSVGIMLFQLVTGRLPFDA
                         :        :.*  *:.**           *:*: * : :::::*  *. .

145595768       NRPVEVAWQHVRQDVPPPSTLVPG-----------LPKALDDLVIRATRRDPGARPADAN
50842217        DSPIQVAWAHVNKDVPAPSQYLANGTKAEHDIAWLVPGYLDSLVRACTSRDPGKRPANGR
126435861       DSALAVAYQRMDNDVPPPSSVISG-----------VPSQFDELVLRATMRDPADRYADAL
145223559       DSSLAVAYQRMDRDVPPPSAAIRG-----------VPRQFDDLVHCATARDPAGRFADAA
41408012        DSMLAIAYRRLDADVPPPSAAIDG-----------VPAQFDDFVQRATARDPADRYADAV
68535808        GTPIETAMARINRPVPAPSTLMPD-----------IPPEIDELVLRACHRNPTQRFVDGT
134098292       DTALSVAYRHVNDDVPAPSERVPE-----------LPPAIDDLVLRATRRDPAVRPADAA
21220588        DSPAAVLYKHLHEDVPPPSAAVPE-----------LAYELDELVAAATARNPEIRPHDAV
29832634        DSPAQILYQHLHEDVPPPSAAVPG-----------LPATLDELVSSAAARNPGLRPPDAV
119026032       DNPVTLVFKHVHEDVPDITTACPG-----------INAGVAAFLARLTARAVEDRPQDAS
23326698        DNPVTLVFKHVHEDVPSVATVCQG-----------IDPSVAAFIAHLTARQVDARPTDGA
28493183        KQPMEVAHKHANLPMPKPSAVNPG-----------VPSVIDDIVLWACSKNPEQRPSDAS
119025056       DSAVAIAYQHVSEVATPPSTIVPG-----------LPKMWDSICAKAMAKDRQNRYATAS
23325797        DSAVAIAYQHVSEVATPPSAAVPG-----------LPKMWDSICAKAMAKDRQNRYATAS
21222231        DSPLAIAYAHVQEQPVAPSAVNRA-----------LPPAVDALVARALKKNPNERFPSAE
                         :        :                :      :      :    *   .

145595768       ALLTRVQV-----
50842217        ALLDLLRRV----
126435861       DMGAELEEIIEEL
145223559       EMADQLDVI----
41408012        EMGADL-------
68535808        EFLEAV-------
134098292       SFLSEV-------
21220588        ALLARAREARESL
29832634        ALLAQV-------
119026032       VAF----------
23326698        AAAEELSQL----
28493183        VLLNAL-------
119025056       EFKNDL-------
23325797        EFKTDI-------
21222231        AMRDECL------
```

Cluster No. 65 multiple sequence alignment

```
CLUSTAL 2.0.10 multiple sequence alignment


108760153       YRVEALIGEGGMGKVFRARQLSLDKVVVLKVLRHTLLSD--ERTVARFQREAKAASRLNH
116623060       YEIIKLLGEGGMGAVYKARDRELDRLIALKVIRPELAGH--PRVLQRFKQELLLARQVTH
108759959       FRILETLGAGGMGRVYKAVQSPLDRLVALKVLNPQYGEGKDPGFQKRFFLEASVTAKLRH
108762484       FSILEPLGIGGMGKVYRALQAPLERVVALKVLNPSFPSSRDPGFQKRFLREASLTSKLRH
108759118       YKIESVLGQGGMGMVFRATQTSVQRPVAVKTLNPSLAAA--PQFFERFRREAELASRLRH
116623034       YEILAPIGAGGMGEVYKAADTSLDREVAIKVLPHAFAQD--PDRLGRFEREAKILASLNH
116626431       YEIIAPLGAGGMGEVYRAIDTRLKREVAVKVLPEAFAHD--PERLARFKREAEVLASLNH
116625010       YEIISPAGAGGMGEVYRARDTRLDRTVAVKVLASNLTAR--PDVLQRFEREARAVSTLNH
116619169       YEILEQLGEGGMGVVYKARDTQLDRFVAIKVLRRDRVAD--SGRKGRFVQEAKAASSLNH
94969743        YKIFSQLGSGGMGVVYEAEDTTLGRRVALKFLPEQYSQN--PASLERFLREARSASALNH
94971422        YRIVEKLGGGGMGVVYKAEDTRLHRFVALKFLPTELAKD--PQARARFEREAQAASALNH
108757782       YQPVRFLGQGGMGRVFLAYDPMLRRNVALKFVR---GDD--PELARRFLSEARAQARVRH
32475633        YKLLGHIGSGGMSSVYLAEHIGLGDKRAIKVLPKKRVND--ASYLARFKLEAKAIASLNH
32477250        FLLLDQIGGGGMGAVFRARDEQLGRTVAVKVIP-FAADD--PDLQRRFRNEAQSAAKLDH
38232689        YRLGDVIGTGGMSEVYEATDVLLGRKVAVKMLRADLARD--VNFRERFRREAQNSGKLNH
126433388       YRLQKLIGRGGMGEVYQAYDTHTDRVVALKVLPPHLAQD--ATFQRRFRREAQAAAGVQD
                :      * ***. *: * .       .:* :              **  *      : .

108760153       PNSISVLDFGQAED---GALFIAMEYVAGQDLHQILS-REWPLNEGRVVRIVSQVLSALS
116623060       KNVIRIFDLGVSEN----LKFITMEFVEGRDLSSLLD--ERKCTVPEALDIIQQTCRALE
108759959       PNTVTVIDYGKTED---GIYYIAMEYLEGLTLGQLLT-QMGPLPWPRALNITQQIARSLR
108762484       PNTVTVIDYGQTDD---GIFYIAMEYLDGRTLAQVLG-QVGPLAWSRAIAITQQICRSLR
108759118       PNVITIFDFGRSPD---GTCYYVMELLEGESLKETVK-RQGPMSLRRALSLVEQASQGLA
116623034       PNIAQIFGVE-GG-------ALVMELVSGQTLSERIA--EGPVPVAEALRIVAQIAEALA
116626431       SHIATIHGVE-ER-------ALVMELVEGESP-------HGPMPFDDAWRIASQIAAALG
116625010       PNICTLHDVG-TDN---GTPYLVMEYVEGETLADRLE--RGPLPLAEAWRIAIQIGDALD
116619169       PNIVTIYEIG-VDE---GADYIVMEFVSGRTLDRLIP--RGGLRLADLLKYAIPIADAMA
94969743        PNICTIYAAE-QFD---NTWVIAMELLEGSTLGQLIV--DGTLNTDRILDIAIQVSDALD
94971422        PNICTIYDIG-DDN---GQGFIAMEFLEGVTLKHRIT--TKPMETGEIVTLAIEIADALD
108757782       ERVCEVYEVG-EVR---GRGYIAMRFVDGQSLGQLAR----SLTLEQKVLVLRQAAEGVH
32475633        PNIVLAHDIDNDG----DVHYIVMEYVDGVDLQVLVR-RDGPLDFSTAAELISQAARGLA
32477250        PLIARVFDVGNDG----PWHYIVFEYIDGANVRDMVA-NGGPLSLDDALFFTTQVAEAIG
38232689        PAIVAVYDTGETPRAGLNTPYIVMELVNGRTLRDIVR-EDGPLTPSQAAHTLIPVCHALQ
126433388       PHVVPIHSYGEID----GRLYLDMRLIEGRNLGTILEGADKPLLPSFAVKVVEQVATALD
                                       :. : *                            .:

108760153       DAHGAGVIHRDLKPENIMVEPRRNEPDFVKVLDFGIAKI------------TDSTDDGPA
116623060       AAHGESVIHRDLKPQNIMLS----EGGKVSVMDFGLARS------------LEST----A
108759959       EAHKVGLIHRDLKPANVMVLNQETDHDVVKVLDFGLVKSFIGD--------AAPVSQDTS
108762484       EAHSQGIVHRDLKPANIMLLNEQ-DQDLVKVLDFGLVKS-VAA--------PQEGQLSPE
108759118       HAHAEGCVHRDLKPHNIMVQQLS-GQDFVKVLDFGLVKA-------------MESEEEEQ
116623034       AAHDRGVIHRDLKPANVKLS----PDGRVKVLDFGLAKSLSGG---GSGADSRAETLTAA
116626431       YAHDKGVVHRDLKPANVRVT----PDGVVKLLDFGLAKAFSVQTQYQSSDSENSPTLTIG
116625010       QAHRRGIVHRDLKPGNVMLAGAR-GSTHIKLLDFGLAKLPEAHSANAAGSLTSLPTVVQS
116619169       CAHAAGIIHRDLKPGNVMVG----VDGQVKLLDFGLAKLSDLH-----DSSDSDLTRPMA
94969743        VAHAHGIVHRDIKPANIFVT----RKSVVKVLDFGLAKLAVDRHAVAQTIGATAADPTSY
94971422        AAHAKGIVHRDIKPANLFIT----DRGHAKVLDFGLAKVTATKSAVAVEN-APTTTVDEH
108757782       AAHGAGLIHRDIKPGNILVERTEDGGLAPFVMDFGLARD-----------------WREE
32475633        HAHNRGVVHRDVKPANLLID----SDGRVRLLDMGLALVS-------------KTGDDES
32477250        HASRRGIVHRDIKPSNVIVT----TEGEVKLVDMGLARSD-------------NFDTSED
38232689        VSHDAGIIHRDIKPANVMIT----NTGAVKIMDFGIARAL-------------DDATS-A
126433388       AAHAAGLIHRDVKPSNILIT----DRDFVYLIDFGLARS-----------------AGEPG
                 :   . :***:** *: :           ::*:*:.
```

```
108760153       LTRAG--FVCGTPEYMSPEQARGS--QLDHRSDLYAVGVILYQLMTGLLPFESDSAVGFA
116623060       LTQAG--ALIGTPAYMSPEQALGN--QLDARSDLFSLGIIFYEMLTGVVPFKAETVLASM
108759959       ITQAG--IILGSPQYMAPEQARN---IADPRSDVYSLGVVLYQMLMGRPPFLAAQSIDVI
108762484       ITQNG--TFLGSPQYMAPEQARN---ATDARSDVYSLGIVLFQMLMGRPPFIARDHIELI
108759118       LTSTG--QVLGTPQYMPPEQAGGE--SVDQRSDLYSMAGVLYFCLTGSSPFGANTVRKAL
116623034       MTEAG--TVLGTPGYMSPEQTRGI--ATDKRTDIWAVGCVLYELLTGRRTFRGGTFGDTI
116626431       ATQVG--VILGTAAYMAPEQAKGR--AIDKRVDIWAFGVLLYELLTGEKLFQGIDVSDTL
116625010       LTTEG--TIVGTFQYMAPEQLEGK--EADARSDIFAFGAVLYEMIAGRKAFEGQSQASLI
116619169       ETEEG--SILGTVSYMSPEQAEAR--RVDARADIFSFGAVLYEMATGQRAFLGRSKISTL
94969743        LTSPG--MAVGTAAYMSPEQARGE--DLDGRSDLFSFGSVLYEMCTGRLPFEGTTSPVIF
94971422        LTSPG--STLGTVAYMSPEQVLAK--DLDPRTDLFSFGVVLYEMATATLPFRGDSSGVIF
108757782       GAAPN--AVQGTPHYMAPEQARGEVSRLDRRADIYALGATLYSLLTGRPPFTGATEAEVI
32475633        LTVANNENVLGTADYLAPEQALNS-HTVDHRVDIYGLGCTLYFLLTGRPPFAEGTLAQRI
32477250        MTASG--VTLGTFDYISPEQARDP-RLADIRSDLYSLGCTLFYMLTGSPPFPGGTMLQKL
38232689        MTQTS--AVIGTAQYLSPEQARG--KLADARSDVYALGCVLYETLTGKPPFEGETPFAVA
126433388       MTTAG--STLGTLAYMAPERFNG--GRIDPRSDIYALTCVLYECLTGVRPYPADSLEEQI
                 :  .     *:  *:.**:        * * *::..   ::    .   :

108760153       TKHLTEEPPPPT--RRRPDARISPAMERLILRALSKNPADRPASAE----AFKAELQ---
116623060       LKRTQGAAPPPI--ELVP--AIPKDVSDVVMKCLATAPADRYQSVG----ELLSDV----
108759959       VKHINELPPAFG--SLYPNHGIPGEVEALVMKCLAKTPPERYQSMD----EVMEAM----
108762484       FAHYKEAPPTFQ--QVRPDLHIPPEIEAVVRRCLEKDPARRYQTMD----ELLEGL----
108759118       TASLTQAVPAVN--TKRQGAPVPAALDAFFKKALAPEKEDRYQNAQ----EFIDAM----
116623034       AAVLEREP-DWD--ALPP--STPPRVLGLLQRCLRKDMGRRARDAGDIAIEIEQVLAEPA
116626431       AHVLTKEP-DVS--KAS------PRSHKLLHRCLEKDPKKRLRDIG----DAESLLEEPV
116625010       SAIMKDEPPSIT--ALQP--MSPPALDRLIRQCLAKEPDERWQTVR----DL--------
116619169       AAILQGDPKPAA--EIHA--GLPRELGKIIERCLRKDPAWRYQSAA----DLKISL----
94969743        AGILERDPLPPE--QVNP--NISPKLSEIICKSLEKDRDLRYQTAA----ELRGDF----
94971422        DAILHAEPVPAV--RLNP--DVPEKLEDILAKLLEKDRDVRYQSAA----ELKADL----
108757782       TRLQHEEPAPPR--ALDA--DIPSDLEAIVLKCLEKQRPARYDSAR----ALAEDL----
32475633        AKHQNEMPKA----IRELRPECPGELEGIVVKMIQKDPRYRYQNAT----DVAEVLEKF-
32477250        LSHGNAAIPD----IREHREDVPAEMTAILNKMLAKLPEERYQ--------RSETL----
38232689        YQHVQEDPVKPSEYIADLSPTAAINVDAVVLTAMAKHPGDRYQTAQ----EMCADL----
126433388       AGHIVRPVPK-----PSDTDPRLAAFDEVIERGMAKKPERRYQSAA----EMAAAARRAL
                                            ..   :      *

108760153       -
116623060       -
108759959       -
108762484       -
108759118       -
116623034       G
116626431       G
116625010       -
116619169       -
94969743        -
94971422        -
108757782       -
32475633        -
32477250        -
38232689        -
126433388       -
```

Cluster No. 66 multiple sequence alignment

```
CLUSTAL 2.0.10 multiple sequence alignment


111022033       FDLMTGLGRGAFARVFLARQRSLQRLVAVKISQDHGTEPQTLAQFDHDYIVRVFDQRLLA
54024689        FDLMTSLGRGAFARVFLARQRSMQRLVAVKISRDKSTEPQTLAQLDHRHIVRVFDQRVLP
111021377       FDLLMGLGSGAFGRVFLARQRSMQRLVAVKISHDKGNEPQTLAQLDHNYIVRVFDQRLLE
111023678       FDLLMRIGSGAFAQVYLARQQSMQRLVAVKISHNHGIEPQTLAQLDHEYIVRIFDQRLIA
111020043       FDLMMELGHGAFARVFLARQRSMQRWVALKISRNIGNEPQTLAQLDHPYIVRVYDQRVLE
32475105        FEIIQKLGEGAFAHVYLARQNSMSRLVALKVANDTGDEPQALAQFDHPNIVRVFDQRQVA
                *:::  :* ***.:*:****.*:.* **:*::.: . ***:***:**  ***::*** :

111022033       GR---------DLRLLYMQYVPGGTLLDVVGRVRETPPDSRS--GAVLLDAIDRALVGRG
54024689        SS---------GLRLLYMQYVPGGTLFSVLERLRAHPAQARARGGALLLDVIDEVLTGKG
111021377       DR---------QLRLLYMQYLPGGTLLRVLRRVRVTPEQDRT--GSLLLDVVDEEMREKG
111023678       DG---------ELKLLYMQYLPGGTLLDVVHLLRSTPPEHRS--GQLLLDAVDGVLAAKG
111020043       DR---------RLRVLFMEYVPGGTLLDVVRLVRKTPPEQRS--GQLLLDSVDRVLAERG
32475105        VRDDEAAGRLSELYLLYMQFHPGGTLADVVRLARSVPLVQRD--GDIHLRAVDQALLESA
                            * :*:*:: *****  *:   *  *   *   * : *  :*  :   .

111022033       EIRPSESGVRQEIATLSWPEAVSWLGRRLAQALDYAGRHGVLHRDVKPANVLLTAEGVPK
54024689        EIRPGESATRAELAGLTWPETIAWLGRRLADALDYADRCGVLHRDIKPANVLLTAEGEPQ
111021377       ELRPTDSSVRDEIASLTWPETIAWLGRRLAEALDYAGKRGVLHRDIKPANILLTAEGVPK
111023678       EVRPSESAVRARASALTWPETVAWLGCRLADALQHASERGVLHRDIKPANVLLSAEGIPK
111020043       EIRPEYSSVRDEIAALSWPETVAWIGLRLADALDYAEEHGVLHRDIKPANVLLGLEGIPK
32475105        QVVPDRSTTRAWLVGCEWPKVVAWVGLQLADSLHTAHEAGVLHRDVKPANVLLTAEGLPQ
                :: *  * .*       **:.::*:* :**::*. * . ******:****:**  ** *:

111022033       LADFNISFSGNIAGASPVAYFGGSLAYMSPEQLEAIHPDRPGTPGDLDTRSDLYSLAVVL
54024689        LADFSISFGSTVTGASPVAYFGGSLAYMSPEQLAAVHPDLPGTAADLDVRSDLYALAVML
111021377       LADFNISFSDTVTGASPLAYFGGSLAYMSPEQLEACHRGMPGTAADLDTRSDIFALGVML
111023678       LADFNVSASNRIKGTSPLAYFGGSLAYMSPEQLEACHPGSPATAADLDTRSDIFALAVML
111020043       LADFNVSSSDSVENRGAGGFIGGSVAYMSPEQLEVIDPSIHRTAADLDTRSDIFALGVML
32475105        LADFNVSMAGTAGRAGAASSLGGSVGYMAPEHLDAMSITRDGRPEDVGEAADLYSLAVLL
                ****.:* ..     .. . :***:.**:**:* .        . *:.  :*:::*.*:*

111022033       WELLTGRRPFDDSDAELHSHPPGDRTTLDAMLARRR-GDAEPAADELPADCP--NALRRV
54024689        WELLTGHKPFGDD----HVTG-GDRTTLDGMLERRRRGPGALPYADLPPDCP--AALRRT
111021377       WELMCGRRPFEDE----DVSS-ESLVALEAMLDRRSRDIDPEFLDQLPYDCP--PSLRRV
111023678       WELLTGRRPFADE-----TSAGESETSLARMLELRRSPVEAKFLSELPPDCP--MALRRV
111020043       WELLTGARPFPDD------DGTTSPPSPRVLLESRRRGVDPE-RSALPEDCP--AALRRI
32475105        WELWQGKRPFDCS---------HETHSWTELLEEQHAARKREFLVPDRLDTPSERVLESV
                ***  * :**  .          .  :   :*  :              * *    *.

111022033       LLKSLAPEPKDRFSSGAELAQQL--
54024689        LARALDPDPDRRWPTGADMAQQF--
111021377       LLTCLAPKPEDRWSSGAELAQQF--
111023678       LLKCLSPDRDDRYPSGSELAQQL--
111020043       LVKSLAPDRDNRWSSGRKLTRQF--
32475105        LRSALSVNPGDRPENGAAMSGSLRL
                *  .*  .   *  .*  :: .:
```

Cluster No. 67 multiple sequence alignment

```
CLUSTAL 2.0.10 multiple sequence alignment


1652588         YLPVKLLGQGGFGAAYLALDRFTPTMRFCVVKQFQPSG------NLNQEQLDLALSLFER
22299975        YVAERVLGRGGFGAAYLARDLDTPGWRYCVIKQFLPN-------VSDPQSLQKAQELFER
86606530        YRAFKRLGQGGFGATFLARDEDMPSKPWRVIKQLRPA-------ENSPQLARLAEELFNR
113476860       YKIIKQLGHGGFGRTYLAEDNH-RFQELCVLKEFAPQ-------VHGSYALQKSQELFER
17131504        YLIVRQVGQGGFGRTYLAEDIN-RFRELCILKEFSPQ-------VQTAYVLQKAEELFQR
17131503        YRIIRQLGQGGFGRTYLAEDIK-KSNKTCVLKEFAPQ-------VEQKEDLQKAKELFER
113474330       YKIIKPLGSGGFGDTYLAQDSDLPGKPKCVVKHLSPK-------SSDPIVLNIARKLFER
113474331       YKIIKPLGSGGFGDTYLAQDSDLPGKPKCVVKHLSPK-------SSDPIVLNIARKLFER
113474332       YKIIKPLGSGGFGDTYLAQDSDLPGKPKCVVKHLSPK-------SSDPMVLSIARKLFER
17131426        YKVTQVLGAGGFGKTYIAEDIKLYNN-LCVVKQLQPM-------ANDPMTLQVARRLFAS
17132907        YQIISHLGGGGFGETFVACDTHLPGLPKCVVKKLQPQ-------ANDPATLEIARRLFDT
17133975        YKILKVLGYGGFSETYIAEDTQRPGNPQCVVKQLKPV-------NTQGKGLQLARRLFNL
37521121        YKILQILGAGGFSQTYLALDTRRPSSPTCVVKHLKPT-------SENPGSLQIARRLFRS
86606961        YEILGLLGQGGFGVTYKAVDRDTFNT-PCVVKRLRPL-------STDPYTLATARKLLER
22299003        YKLIRVLGAGNFGQTFLAEDVHRPIRAKCVVKYLRPA-------RRDAAFLPLARSLFQR
17133132        YQVIRTLGAGGFGETYLAEDTYMPSKRRCVVKQLRPI-------QNNPQIYQLVQERFQR
17133502        YRVIKTLGSGGFGETFLAEDSQMPSNRRCVVKQLRPI-------HNNPQIYQLVQERFQR
17133504        YQVIQILGAGGFGETLLAEDTHMPSRRRCVIKRLKPV-------SNDPQAYQSIQQRFER
17132363        YRVLKPLGQGGFGATFLAHDQILPGEPSCVIKQLRPS-------GTAPHILQMARELFER
22298869        YRAIKVLGRGGFGTTFLAVDTKLPGNPTCVIKQLRPA-------ATAPHILTMARELFLR
17130658        YQILRILGRGGFGITFLARDAVLPGNPLCVIKQLCPK-------VTSAKSWQNACRRFEK
113474678       YRAIKPIGQGSFGKTFLAVDEYKPSLPRCVIKQFFP---------QNSANALKAAELFRR
113477154       YRPIKPIGRGGFGRTFFAVDEDKPSHPPCVIKQFLP---------QNTGDPKKAAELFQQ
113474087       YRLLKPIGMGTFSRTFLAVDEDIPSKSVSVIKQFLPIATPGKNAEQNINEIEKASISFHR
17130683        YRVLGLLGEGGFSKTYAAEDADRLDAP-CVIKQFFP-------QIQGTGQRSKAAEFFKE
                *     :* * *. :  * *         ::* : *                     :

1652588         EAVVLEKLGNRHDQIPDLFAYFPLLVDDPRTGKDQDFFYLVQEFINGQD----LEKTVEK
22299975        EAKVLEELG-QHAQIPDLLAFFREEVAGFNSSSEESYFYLVQEFIDGET----LEDELAQ
86606530        EAEVLERLG-EHSQIPKLYAHF----------QEGGKFYLVQEFIEGIT----LAQEMRR
113476860       EAGVLYKLRH--PQIPGFREMFRYKL------NAEGYLFLVQDYIKGKTYRALLENRKLQ
17131504        EAKVLHQLQH--PQIPRFREIFRVNL------AGKEYLFLVQDYVEGETYSGLLNHRIQQ
17131503        EANVLKKLQH--SQIPRFHGSLQAQI------GTKDFFFLVQDYVEGDNYLQLLEQRQSQ
113474330       EAETLYKLGSDSDQIPRLLAHFQE----------EKEFYLVQEYIEGQD----ISRELTP
113474331       EAETLYKLGSDSDQIPRLLAHFQE----------EKEFYLVQEYIEGQD----ISRELTP
113474332       EAEALYRLGTDSDQIPRLLAHFQE----------EKEFYLVQEYIEGQN----ISQELAP
17131426        EAELLHKLGT-HDQIPQLLAHFEE----------HQEFFLVQQFIDGHP----LSDELTP
17132907        EAQVLYKLGS-CDRIPQLLAYFEE----------DAEFYLVQEFIPGHD----LSKELTP
17133975        EAQSLEKLGS-YQQIPQLLAYFEQ----------EAEFYLVQEYIIGHP----LNQELPS
37521121        EAETLERIGH-HDQIPRLLAYFEE----------DEEFYLVQEFIEGHV----LATELQP
86606961        EAKVLNRLNN-YDRVPRLLAHFEQ----------DQEFYLVEEFIAGHD----LTHELVD
22299003        EAQILERLGT-HDQIPRLLAYFEE----------DNEFYLVQDFIEGQV----LRQELLL
17133132        EAAILEELGGATEQIPALYAYFSA----------DGQFYLVQEWVEGDT----LTGRLQQ
17133502        EAAILEDLGSYSGQIPTLYAYFQS----------NTQFYVVQEWVEGDT----LTAKLKQ
17133504        EAATLEFLGEHSNQIPRLYAYFSE----------NGQFYLVQEWIHGHT----LRQLLVS
17132363        EAKTLGTIGN-HPQVPRLLDYFEE----------QEQFYLVQEYISGST----LQQEVKL
22298869        EATTLGKVGN-HPQLPRLLGYLEN----------ENEFYLIQEYVGGLT----LQQEVKR
17130658        EAKTLATLGS-HSQIPMLLNYFEG----------DGELYLVQEYVRGYT----LAQEVRH
113474678       EAIRLDELGQ-HPQIPELMAHFQQ----------GEQQYLVQEFIDGQN----LEQELVA
113477154       EAVRLDELGK-HPQIPELLAHFEQ----------DNYQYLVQEFIDGSN----LAQESIK
113474087       EALQLDKLGK-HPQIPSLLAYFFV----------SSYKYLVQEYIQGKN----LAQELKE
17130683        EAFRLYELGENHTQIPRLLAYFEQ----------GSSLYLVQEFIKGLT----LLQEVQQ
                **  *  :     ::* :   :                :::::: *      :
```

```
1652588           HGPLSEAEVRWVLTEMLKILSFVHGTGAIHRDIKPSNLMRDQ-----------EGKLYLL
22299975          QGCFSEEEVRQVLRELLPVLQYVHERGSIHRDIKLSNIMRQHPSKTKF---PGQGRLYLL
86606530          NGPFSEEQARQVMQEVLLILSYVHSHNTVHRDIKPANLIRRKE----------DGRLVLI
113476860         GKLFQEAEVIKFLLQLLPVLEYIHNLGVIHRDISPDNIILQTS----------DKLPVLI
17131504          GLRFTEAEIRQLLQQILPVLDYIHSLGVIHRDISPDNLILRSV----------DKLPVLI
17131503          GKTFTEEEVITLLRQTLPILIYIHSQNTVHRDISPDNLILRRS----------DNLPVLI
113474330         GKKLSESDTIALLKGILEALVVAHENNIIHRDIKPQNLMRRGS----------DNKVILI
113474331         GKKLSESDTIALLKGILEALVVAHENNIIHRDIKPQNLMRRGS----------DNKVILI
113474332         GKKLSESDTIALLKGILEALVVTHENNIIHRDIKPQNLVRRGS----------DNKVILI
17131426          GKRLSEPYTIALLKNILQPLAFVHQNNVIHRDIKPPNLIRRKS----------DGKVVLI
17132907          GKVFTQDEVTILLQEILTILEFVHQQNVIHRDINPRNLLRRQ-----------DGKLILI
17133975          GRSISEAETVAIIREILEILVFVHENGVIHRDIKPSNIIRRDS----------DRKLVLI
37521121          DAPMGEARVAAMLQDVLSTLAFVHSQGVIHRDVKPDNLIRRSS----------DGKLVLV
86606961          GKPLSEPEVVQLLWEVLEILRHVHAHQILHRDIKPANLMRRDF----------DGKLVLI
22299003          GSGWPEARVIELLHDALGILSFVHQCGVIHRDIKPDNLIRRQF----------DQRLVLI
17133132          QGLFTESAIQELLVNLLPVLEYVHSKHIVHRDIKPDNIILRHR----------DGKPVLI
17133502          QGVLSESAVRDILINLLPVLEYVHSKRIIHRDIKPDNIILRHR----------DGKPVLI
17133504          QGIQGEGIVKTILLSLLSVLDYVHSKGIIHRDIKPDNIILRDF----------DQKPVLI
17132363          NGTLSEPGVKQFLSEILPLLQYIHEHKVIHRDIKPANLIRRSQ----------DARMVLI
22298869          FGPKSEEEVKQVLQEVLPILDYLHKNGVIHRDIKPANLIRRDI----------DKKLVLI
17130658          HGTKTEAEVKQFLQELLPILQYLYQNQVIHRDIKPQNIVRCAD----------DRRIVLI
113474678         NGTFTENQIQELLWDLLSVLEFVHERQVIHRDIKPENIIRRNSITLPDGGGENKGQLVLV
113477154         NGPFDSNQIQQMLNELLPVLKFIHEQKVIHRDLKPENIICRSSTPETIGWMTNTNKLVLV
113474087         QGTFDEQKIRKFLKEILPVLQFIHKNQVIHRDIKPENIIRRGADKTGYGWGDN---LCLV
17130683          E-PFNEEKIRQLLIDLLPVLDFVHFHHVIHRDIKPENIIRRDGDGK----------LVLI
                       .     .:   *  *   :    :***:.  *::                   *:

1652588           DFGAVKQATAGVGASNEG---------STGIYSMGFAPPEQMAGNQVYPATDLYALAVTC
22299975          DFGAVKQVSQTSMESR-----------STGIYTAHYAPPEQIRGEQVFPSSDLYALAVTC
86606530          DFGAVKQLGPGHTLSEE----------GTAIRSLGFSPPEQMAGQAVGPASDLYALAATC
113476860         DFGGVKEIATKVASEYAQ---LES--GITRLGKLGYAPDEQMRLGVINNDSDLYALAVTA
17131504          DFGGVKQVVAVVASQYYQPGVVASPPAATLLGKVGFAPPEQMQTGNVSPHSDLYALAVTA
17131503          DFGGVKQLPASQGFWSTK---LAG--NNTLLGKKGYAPEEQLRQGKVFINSDLYSLAVTA
113474330         DFGAVKEISTLTITQ------QGATTLTVAVGTPGYIPSEQSN-GKPKLSSDIYAVGMVG
113474331         DFGAVKEISILTIAP------QGATTLTVAVGTPGYIPSEQSN-GKPKLSSDIYAVGMVG
113474332         DFGAVKEISTLTIAP------QGATTLTVAVGTPGYIPSEQSN-GKPKLSSDIYAVGMVG
17131426          DFGAVKQIGTQVVNG------EGVTKMTVSIGTAGYMPSEQSR-GSPRLSSDVYAVGMIG
17132907          DFGAVKQITTQIVTP------TGENKSTVIIGTPGYIPGEQAQ-GNPKFSSDIYAVGIVA
17133975          DFGAVKEISLPQTDN------QEPIPFTIGIGTKGYAPSEQCF-GRPQYNSDIYAVGMIG
37521121          DFGAVKTVWSRPAALPGGQ--RGGTVAGTIIGTPGYMSTEQGR-GKPRPSSDIYALGMIG
86606961          DFGAVKEVGRLVSHA------PGQTQVATVIGSVGYIAPEQLG-GKPQPCSDIYSVGVLA
22299003          DFGAVKEMGVSIGGG-TLV--GDANPQTIAIGTPGYMAPEQAQ-GRPRPASDLYSLGMVA
17133132          DFGAVRESMGTVVNS------QGNPTSSIVIGTPGYMPSEQAA-GRPVYSSDLYSLGLTA
17133502          DFGAVRESMGTVINS------QGNPTSSIVIGTPGYMPSEQAA-GRPVYSSDLYSLGLTA
17133504          DFGAVKETIRSVVSS------PGYATRSLVIGTPGYMPSEQAV-GRPVYATDIYSLGLTA
17132363          DFGAVKNQVSQAITN-QSA--N-TALTAYAIGTPGFAPPEQMA-MRPVYASDIYALGITC
22298869          DFGAVKDKVTQAMVE-NAP--ELSTFTSFAVGTPMYAPPEQMA-MRPIYASDIYALGVTC
17130658          DFGAVKEKLSDLGIS-SFN--Q-NSHTNF-VGTTGFAPPEQFS-LRPVYASDIYALGMTC
113474678         DFGASKVITNSSLGK-----------TGTMIGSAGYVAPEQLA-GKAAPASDFYSLGVTC
113477154         DFGAAKVITGTSLMQ-----------PGTIIGSPEYVAPEQLR-GHAIFASDIYSLGVTC
113474087         GFGSAKAISIHPTVK-----------TDTIIGSPEYTAPEQLM-GKPVFASDIYSLGIVC
17130683          DFGGAKQVTQTSIAR-----------QATAIYTLGYAPTEQMA-GFACHASDLYALGVTC
                  .**. :                        : .  : . **         :*.*::.

1652588           LYLLTGKTAQ--------DLY-DAYHNQWNWRSPGLK----VSQPLADVIDRLLLPTPKD
22299975          IVLLTGKDPE--------KLF-DAYNNRWNWHSYVPS----VSQQLQQILDRMLQPAPSD
86606530          LNLMTLESPA--------KFY-DHNTGQWDWSDR-LQ----LSPEFDAILRRMLHPVISQ
113476860         LVLLTGKEPS--------LLT-DVHNLTFNWRIEIS-----ISENLGNVLDKMLASKPSD
17131504          VVLLTGKQPQ--------ELL-DTYNLSWNWQREIS-----LSPILGQVLDKMLAARPGD
17131503          LVLLTGKEPQ--------KLY-DSYQGVWRWGKEIN-----ASIQLESVLKRMLAYKPGD
113474330         IKALTGKDPQ--------SLPTDPKTGNIIWRNKAE-----VSNDLANVLDKMVREYFSE
113474331         IKALTGKDPQ--------NLPTDPNTGNIIWRNEAE-----VSNDLANVLDRMVRDHFSE
113474332         IKALTGKDPQ--------NLPTDPNTGNIIWRNEAE-----VSNGLANVLDKMVRDHFSE
17131426          IQALTGLMPH--------QLQEDIQTAEIIWRELVQ-----VSPSLADVLDRMVRYDFRQ
```

```
17132907        IQALTGLLPH--------QLEHDADTHEIIWQNHAQ-----VSSEFARFIDKMVCYDFRQ
17133975        IKALTGISPH--------DLPRDENE-EIKWSDKAL-----VSQGFAQILSKMVREDFKQ
37521121        IQALTGLNPM--------ELPEDSRTGEPLWQDNAQ-----ASPGLCAILSKMVSYHFKD
86606961        IQALTGRPPT--------KIRDDPRTGQLLWRDLVP-----VSKELAEIVDRMICPRWQE
22299003        VQALTGLSPL--------QLSQNPYG-CWCWQATEP-----VSDRLVQFVNKLIHPSPYE
17133132        IYLLTGRQPQ--------TLEIDSQTGEIMWRQYASQ----MNPILASVLDKAIAYHPRD
17133502        IYLLTGKQPQ--------ELETEPHSGEIIWHRYALN----ISPTLAAVIDRAIAYHPRE
17133504        IYLLTGKSPE--------ELPTHPQTGEILWQNMAPH----VSQQLVSVLNQAIKPHAGD
17132363        VYLLTSKTPK--------DLDYNPTTGEVMWEHLVQ-----ASDHLIGVLRKMLEVSVRS
22298869        VYLLTGKSPK--------EIERDPRTGEWHWQRHVKN----ISPQFAALLNKMLQDAVKD
17130658        IYLLTGKGPL--------EMESDSKTGEICWYNYVK-----VSDDFAKIISKMVKFSLDE
113474678       IYLLTQVAP---------FELFDVLEGQWVWRQYLQSN---ISEDLGRVIDQLLAPNINE
113477154       IYLLTQISP---------FDLFDVMADKWVWRDYLNQP---FNSKLGKVIDKMLMTNPQI
113474087       IQLMTQLNP---------FDLR--YGEEFLWQQHLPRP---VSKSLSNIINKMLQVSLSE
17130683        VRLLTQCLPQQNLYGHIDDGLYDPMNGKWLWQEYLQDRGITISENLSQILDKLLKHLPSE
                :  :*   .                     *           .  :  .: : :

1652588         RYASAEEVLAVL---
22299975        RYQSAAQVLADL---
86606530        RFATATEALRAL---
113476860       RFSTARQVITAL---
17131504        RYQSAQQVLHAL---
17131503        RYQNAAQILTDL---
113474330       RYQNAMEAMEAL---
113474331       RYKNAMETM------
113474332       RYKNAMEAMEAL---
17131426        RYQSAVEALATV---
17132907        RYAAATTALQAL---
17133975        RYQSALAVLAAL---
37521121        RYQTAHEALEALQHL
86606961        RFQSAAEVMEAL---
22299003        RFATAADALAGL---
17133132        RYATARAMLDNL---
17133502        RFTTAREMLEALQL-
17133504        RYSTASKMLYAL---
17132363        RYQSATDVLKAL---
22298869        RYQTAMDVLADLQL-
17130658        RFQKPQDVIKAL---
113474678       RYQFVEQVLKEL---
113477154       RYQSALEVMKEL---
113474087       RYHSIEEVLKDL---
17130683        RYQSAAEVLHDL---
                *:      :
```

Cluster No. 68 multiple sequence alignment

```
CLUSTAL 2.0.10 multiple sequence alignment


116621584       YAIVDSLGAGGMGEVYRARDSRLQRTVAIKIISPARFADDGHKRRFLQEARAASALTHSG
116622694       YLVVERLGQGGMGVVYKARDSHLDRFVALKVLPPERVADPERKRRFVQEAKAASALNHPN
116621572       YQVTERLGSGGMGEIFKARDPRLNRTVAIKALSIEGMGDPDRRRRFIQEAQAASGLNHPN
116621573       YQVIEKLGAGGMGEIWKAQDARLNRMVAIKVLTNANAGDSDRRRRFIQEAQAASALNHPN
116625897       FGLLEKIGEGGMGQVYKARDTRLERFVAVKVLSDSRLADPGSRSRFTQEAKAASALNHPN
29830915        YRLTGRLGRGGMAEVFAAEDVRLGRTVAVKLLRSDLAEDPVSKARFTREAQSVAGLNHHA
108757908       YQLEQLLGEGSMGRVFQARHTRLGRQVALKVLRPEHARDGGFVRRFFQEARTVNQINHEH
86160512        YELTGHLATGGMGAVFQARHVHLRKDLAVKVLRPELSASPDLVERFRREAEIASALQHDH
86742862        YEVTAMLGHGGHAVVLAGRHRRLGRTVAIKVLS-TSAADGGAHGRFLAEARLLAGLDHPH
                : :   :. *. . :  ... :* : :*:* :      .     **  **.    : *

116621584       IVTIYDIGA----EGSIDYIAMELVEGVTLDKACAR----PYSEKLALAASLASAIAAAH
116622694       IIHIYDIAK----DGDIDYIAMELIEGRTLASVIGQRGL-RTEQILKYGTQVADALAKSH
116621572       IITIHDIIS----DSENEYMVMEYVSGKTLSELIQAGGV-GVEKTLRYGVQMADALAAAH
116621573       IITIYDITS----DEESEYMVMEYVAGKTLADLIPAGGL-GVAKTLQYSVQMADALRAAH
116625897       IIVVHEIGE----HDRQIYIVMELVDGKPLSQLIPKKGM-RLTEALHIAVEIADALAAAH
29830915        VVAVYDSGEDVVGHSTVPYIVMELVEGRTIRDLLLNAEAPGPEQALIIVSGVLEALAYSH
108757908       IVEIFDFVDE-SAMGGHVYCVMELLRGQSLSS-LAQAEPLTLARIQRFVVQVCAALGAAH
86160512        IVHVTDFG---RTEEGWLFLAMELLTGESLFDRLRREGALAPAAAVPVLWQICAGLGAAH
86742862        VVRIYDYVE----SGDLCLLVMERLAGGTVRA--RAANGLTVDVVCAIGLATAAALECAH
                :: : :              .** : * .:                         .:  :*

116621584       EKGIVHRDLKPANIMVTAAG----QIKILDFGLAKFNAPVLKG-DESTRTAVS---LTAE
116622694       GAGVIHRDLKPTNIMVTDAG----LIKVLDFGLAKLADNATDE-FAPTRTVRADEIHTED
116621572       AAGIVHRDLKPGNVMVTESG----LVKILDFGLAKVSMATQLT--EETQTLG--APMTVE
116621573       VAGIVHRDLKPGNVMVTDSG----LVKILDFGLAKVSMATKLT--EETQTMGA-APMTVE
116625897       AAGIVHRDLKPANLMVDRLG----RAKVLDFGLAKMTASAAVATDEETRTLAVTQLQTEE
29830915        QHGIVHRDIKPANVIITHNG----AVKVMDFGIARALHGAQST-------------MTQT
108757908       QVGVVHRDVKPDNLFVIHRAGQPDFVKVLDFGVAKLLTAEGST------------TGTVD
86160512        AMGVVHRDLKPENVFLARTASGREVAKILDFGIAKMTDPS-SG------------CATQA
86742862        AGGILHRDIKPDNILFSADG----LLKVTDFGIAKLIGAS----------------GAAP
                  *::***:** *::.   .     *: ***:*:                       :

116621584       GTVAGTPAYMSPEQVQGRPAESRSDVFSLGAVLYELFTGRRAFQ-GDSTVAVMAAVLERD
116622694       GTVMGTLAYMSPEQAQGKQVDGRSDIFSLGAVLYEMAAGRSAFW-RDSRASTMAAILRDD
116621572       GSILGTVSYMSPEQAQGAKVDARSDIFAFGALMYEMITGTKAFS-GASPIATLTAILRDE
116621573       GSILGTVAYMSPEQAQGKRVDARGDIFSFGVVMYEMLTGTKAFA-HDSAITTLTAILRDE
116625897       GVIMGSIPYMSPEQAEGRPVDARSDIFSFGAVLYEMITGRRAFG-GESRVSTLAAVVEKD
29830915        GMVMGTPQYLSPEQALGKAVDHRSDLYATGCLLYELLALRPPFT-GETPLSVVYQHVQDI
108757908       GTIIGTPAYMAPEQAAGLPVDARSDIYAVGNILYELISGKPPFQ-APAFGHLVVQIITQP
86160512        GMVVGTPEYLAPEQATGGAVDARADLYAVGLIAWRMLAGHHPFT-APDARGLLMKQATAP
86742862        STLVGTPVYMAPEQFDGRPPGPACDLYALGIVLYELLSGRPPFVRALSMAQLMDHHLRVA
                . : *:  *::***  *       *::: * : :.: :   .*         :

116621584       PPNASNICPTLPR---GCAAAIHRALAKKPEARYPSMHEF-----------------
116622694       PVPLP---AEVGA---GLRGVIGRCLVKEPSERYQQATEVRAAL-------------
116621572       VKPVRDSVTDCPP---ELEEIIGRSLRKAPGDRWQSMQEV-----------------
116621573       VKPVGELVAGVPA---EVVEIIALALKKDPKDRWQSMQVVHKV--------------
116625897       PVPPSEIAAGTPP---ELERLIARCLRKEVGRRSQSMADV-----------------
29830915        PVPPSEVAEGAPP---ELDGLVMRSLAKDPDDRFQTAEEMRGLV-------------
108757908       PPPLPSHLPSGEPVPPQLAELVMRCLSKEPEARPQSLTEVTTSLLL-----------
86160512        VPPLAEARPELAAWP-ALVAVVARACEKEPGARPASAAELGEAL-------------
86742862        PQPLDAAPP-------AIAAVVDRALRKEPGARPPSARAFALDLAAATAHALGAGWL
```

```
                                     :  .  *    *      .
```

Cluster No. 69 multiple sequence alignment

```
CLUSTAL 2.0.10 multiple sequence alignment


108762298       YEVLSVLGKGGMAEVYRARVLSGPREGWTVALKRLLPALTRDPE----SVSLFAREAQLS
108762839       FQLLSRLGRGGMAEVFLARMQQGPHAGAQVALKRVRPERLRDAE----AHEQLLHEAELA
86157474        FRIQGLIGRGGMAEVFRAVALEGPLAGQTVALKRLRPELARDPG----FVALFEREAAVT
86157561        FLLLDRIAVGGMAEVYVAIR-RGEAAGRLYALKRILPTLAEDAE----FITMFLDEARLV
117927230       YELGEILGYGGMAEVRRGRD---LRLGRDVAIKTLRVDLARDTS----FQTRFRREAQAA
119714294       YQLGELLGRGGMAEVRKGTD---TRLGRVVAVKRLRTDLASDAT----FQARFRREAQSA
119962857       YELGELIGRGGMADVYRGTD---TLLGRTIAVKVLRADLARDPQ----FQARFKREAQAV
54023701        YRIDAPIARGGMSMVFRGVD---TRLDRPVAIKVMDPKFAGDPQ----FLTRFELEARTV
86741763        YTAQERLAVGGMATVYVAHD---NRLDRLVALKVMHPTLNHDPE----FVTRFHREAKAV
134097469       YELRDEIGSGGMGSVWRAFD---GVLDREVAVKRISPETIAASTAIDVLAQRFQREARVT
114798457       WRIDGIIGRGGMGTVYRVRR-ADGLYEQEAALKLMRPLAPEHLA-------LFESERQFL
                :     :. ***. *               *:* :                 :  *

108762298       KQLHHPNIVTVLDAGALE---------GIYFIVMELVDGRDLGQILRRCKVRGIPLPLDF
108762839       RCLRHPYIVGFVEYGELP---------DGGYLALELVEGPDLGRVLARCRRRRIELPIDI
86157474        RRLRHPGIVEVLETGVAS---------GAPVIVMEYVDGRNLKEILARCAERGILLPVDF
86157561        VQLDHPAIVPIHELGMHG---------EGYYIAMDYLPGKDLRALLDRLRARGEPMPVPL
117927230       AALNHPSIVAVYDTG-ESMLDG----VPVPYIVMEYVEGRTLRDILKSES----HILPRR
119714294       ASLNHPSIVAVYDTGEEPAADGSG--ISQPYIVMEFVAGRTLRDILREGR----KILPER
119962857       AALNHPSIVAIFDTGEYSVPGGPGEDVRVPYIVMEYVAGRTLRDMIKAN-----ELGVED
54023701        ARLKHPSLVAVYDQGVD----G-----EHPFLIMELVEGGTLRELLRERG----PMPPHA
86741763        ARLNTPRVVSILDQGSDHIPAG-----LVNYLVMELVRGRSLRQHLGARG----RLPVTE
134097469       ARIQHHGVPQVYDAGLDEN-------ADELFLVMELVRGVSLRAFISPGE----PLPVSW
114798457       ARLEHPGIARLLDGGGGAD--------GRPWMVMEYASGAPVDVWARQTG-----AGPRE
                  :    :  . : *                : ::   *  :

108762298       AVYLGKVLLEALAYAHSATGPQGERLGIVHCDVSPSNLFISRV-GEIKLGDFGVSRVLVD
108762839       SVLIVRQVLEALSHAHHAVSTTGRPLGVVHCDVSPHNVLLSRT-GEVKLADFGVARSRAG
86157474        AAYAAQVLAEALAHAHAGVDAAGAPLGIVHCDVSPSNVFVSRL-GEIKLGDFGVALTPGA
86157561        AAHVAARVADALDHAHRKRDALGTPLRVVHRDVSPANVLLGFD-GSVRIIDFGIAQAALR
117927230       ALEIVADILAALEYSHRN--------GIVHRDIKPGNIMLTHN-GEVKVMDFGIARAVAQ
119714294       ALEITSGVLSALDYSHRA--------GIIHRDIKPGNVMLTPS-GDVKVMDFGIARAISD
119962857       SVGFTLGVLGALEYSHRA--------GIVHRDIKPANVMVCADTGDVKVMDFGIARAMAD
54023701        VRAVAEPVLAAIGVAHAD--------GLVHRDIKPENVLISDS-GEVKIADFGLVRAVAA
86741763        AIEIIEPVIEALAAAHAA--------GIIHRDIKPENILLGDD-GQVKVADFGLARPVSQ
134097469       AAAFAAQICTVLSHAHAV--------PVVHRDLKPGNVLVTTD-GAIKVLDFGIAAILRS
114798457       ILGAMLQVCEAMVHAHGK--------LIVHRDIKPSNILIDET-GRARVIDFGVARIAGG
                       :  .:  :*           ::* *:.* *:::    *  :: ***:

108762298       GKLQGGEV---LGKPYYLSPESLLGE-VNPEADLWAATVVLYELLTLERPFTGTTP--DA
108762839       -AVQDIRR---LGKQHYRSPELLAGD-VSVAVDLWATAVLLYELLALESPFPSGPE--EQ
86157474        AGAPGPGA---LGKVQYLSPEQLRGERPTPACDLFALGAVLFELLTDRPAFPGRDV--NE
86157561        TRRQDTVL---RGKFGYMSPEMVRGQPVDHRSDVFSLGVVLHEMLTGARLFSGKSE--LS
117927230       STATVTQTAAVIGTAQYLSPEQARGEPVDPRSDIYSTGCVLYELLTGTPPFTGESA--VA
119714294       ASSTMTQTAAVVGTAQYLSPEQARGETVDSRSDVYSAGCLLYELLTGRPPFVGDSP--VA
119962857       SAATMTQTQAVVGTAQYLSPEQARGETVDARSDLYSAGCLLFELLTSRPPFIGDSP--VS
54023701        AN-TTSAS-VILGTAAYLSPEQVTAGSADARSDVYAFGILIFELLTGRVPFTGDNS--LS
86741763        PTQALTGG-VVMGTVGYLAPEQVTHGVADTRSDVYAAGVVLFESLTGQLPHSGATP--MS
134097469       DVTPLTATGERLGTSQYMSPEQVRGDRVTPHSDLYSLGCVLYELLSGYPVFEGEYG--AQ
114798457       DGIHVAPL-----SLDYAAPELFSGEAATTASDVYGLAATLYALLAGRPPLALSDAPVPT
                             .  * :**           *::.    :.  *:

108762298       VFNAIRARQYRPLRELRP-----DIPEALEAVVARAFAERPEDRFPTAEEFAQAL
108762839       VESSIRGGRVTPVRMRVP-----GVPDALALVLDRALAPVPSQRFGSAEQFARAL
86157474        VGRRILAGEARAPSSLRP-----EVPAALDALVLRCLARDAARRPASAAAAAAEI
```

```
86157561         VLERVRRAEVPPPSRARP-----GLPPELDAIVLRALARDPAQRFEWASDLRDAL
117927230        VAYQHVREDPVPPSRLNP-----DVTPDIDAIVMKALAKNPANRYQSAAEMRADI
119714294        VAYQHVREPAVPPSDHDT-----ELPPEIDAIVMKALAKRVEDRYQSAAAMRSDI
119962857        VAYQHVRETPDLASAHNP-----EVSEALDSVLVKALQKSRTDRFQDAAAFRRAL
54023701         VAYQRVEKDVPSPGQFIA-----GVPPEFDELVARATAREPAHRFADANEMVAEL
86741763         VAYQSVHGDVPAPSTMIE-----GIPAELDGLVLRATARDPARRPVDGAALLAEL
134097469        QMYRHVEVAPCPLRDVRT-----EVPAELDRLVLDLLAKSPDQRPVDAYEVYEQL
114798457        LARRAVEEVPPPLSAHIPKKDQTALIRDLDAILAKALARNPADRYPAVEAF----
                                         :   :  ::          *
```

Cluster No. 70 multiple sequence alignment

CLUSTAL 2.0.10 multiple sequence alignment

```
13092968        YLLKAKIGNGGTATVYRGV---------DIR--LDRPVAVKVMD-SRYTGDE----QLLT
111018124       YRVDATIARGGMSTVYRGL---------DTR--LDRPVAIKVMD-PQFAADP----AFVT
19553377        YRIETPIARGGMSTVYRCL---------DLR--LGRSMALKVME-EDFVDDP----IFRQ
23493912        YRIETPIARGGMSTVYRCI---------DLR--LGRSVAVKVMD-AAYVNDP----VFRQ
38234190        YRIEAPIARGGMSTVYRCV---------DTR--LGRLVAAKVMD-QRYLDDP----IFKD
91787132        YDLIRVLGKGAMGLVYEGR---------DPN--LDRRVAIKTIK-VENLSEEEAA-DYEV
94971484        YQLLAEIGRGAMGTVYQAL---------DPE--IDRLVAIKTFS-AFDSTSHEGV-AFRD
111019572       YQLRALLGRGGMGEVYEAF---------DTV--KGRTVAVKLLD-LELANDATFQ----Q
126435989       YELRSLIGVGGMGEVYRAY---------DTV--KGRTVALKLLR-AEMAADPSFQ----E
21225517        YRLLERIGRGGMGEVWRAR---------DES--LGRRIAVKCLKPLGTQHDHSFTRVLRE
29827806        YRLLDLIGRGGMGEVWRAR---------DES--LGRQVAVKCLKPLSTHHDQSFTRVLRE
86743110        YRLNGRIAAGGMGEVWRGL---------DLT--LGRPVAVKLLR-PEYASDESFL----V
124268135       YALKYRIGDGGLGTVYAAD---------DPL--LSRRVAIKTLNLMPAGARTPLETDARE
86159869        FELLREIGRGGFGLVFEAR---------DRE--LGRLVAFKAMRPSRAEP-AALEKPLRE
108758255       YVLKALLASGGHGSVYEAE---------HRI--LGRRAAVKVLH-PHLADQGEML----K
108763488       YELRSRIGVGGMGLVYEGI---------QPL--IGKRVAVKVLR-PELAHSTEQV----E
108759164       YRLVRRLGAGGMGTVYLGE---------QTR--IGARVAVKVLH-PHLGRDESLR----A
108760510       FRILRRLGQGGMGSVYLAE---------QVG--IGQQVAMKFLN-SGLSMDPDVA----R
116619999       YRIISSIGLGGMGSVYLAE---------RADGEIQQRVAIKLLR-ADG-HRPEWR----E
32474038        FEVIREIGRGGMGIVYEAK---------QLS--LERIVALKILPRSITLDQKQVA-----
32476260        YQLLKVLGVGGMGVVYLAK---------QRE--LDRLVAVKMIRSGILAGQDEVK-----
32473866        YQVLRRLGSGGMADVYAAK---------HLK--LRRDAALKVLRRTPQTSQEDLQ-----
108758216       YRLRQRIGVGGMGTVYEAE---------QLD--VGRTVAVKVLRQHLLGEPAVHA-----
108759500       YEIAGVLGRGGMGTVFLAN----------HLRLRGRQVAIKVLRHDAGMGAEAFV-----
108762243       YRIVSLLGRGGMGSVYLAQ----------HLRLPGKQVAVKVLRGGDHLTPEIFA-----
116625893       YRIVSKLGEGGMGAVYRAT-----------DTKLNREVAIKVLPDALANDPDYLA-----
116626707       FEIVERLGEGGMGAVYKAR-----------DRHLDRDVALKVLLPEAVGNADRRR-----
29831643        YLLEEPLGRGATGTVWRARQRETAGAEAAVAGQPGETVAIKVLKEELASDADVVM-----
32476268        FELGAVLGVGTVGTVYDGKILDDVEVHPAAEAIRGQDLAVKKLHPAVSQDDLIQA-----
                : :   :. *  . *:                      * * :

13092968        RFQL----EARSVARLKDPGLVAVYDQGID-----ARHPFLVMELIEGGTLRELLAE-RG
111018124       RFEF----EARSVARLKHPSLVAVYDQGHD-----REHAFLVMELVDGGTLRELLRE-RG
19553377        RFRR----EARSMAQLNHPNLVNVYDFSAT-----DGLVYLVMELITGGTLRELLAE-RG
23493912        RFRR----EARSMAQLSHPNLVNVFDFSSS-----GDHAFIVMELITGGTLRELLAE-RG
38234190        RFRR----EARSMAQLSHPCLVGVYDFSSD-----GDNIFLIMELITGGTLRELLAE-RG
91787132        RFRT----EARSAARLQHPNIVSVYDSDRD-----GDMAYLVMEFIHGDDLKHHMDQ-GK
94971484        RFAQ----EARAAGRLAHPGIVAIYDRGEEPS---TGSPYIVMEYIAGQPLSRLLAQSGG
111019572       RFRR----ESHVAARLQEPHVIPIHDWGEID-----GVLYIDMRLVPGQDLRSLLRSQG-
126435989       RFRR----ESRVAARLQEPHVIPVHDFGDIG-----GVLYIDMRLVEGASLKDVLQAGG-
21225517        RFRR----EARVAAALQHRGVTVVHDFGEWD-----GVLFLVMELLEGNDLSRLLEDNKG
29827806        RFRR----EARVAAALQHRGVTVVHDFGESD-----GVLFLVMELLEGRNLSQLLEDNKH
86743110        RFRG----EARHAARLSHPGVASVYDYGEVATADDYPTAYLVMELVEGEPLSAALHREK-
124268135       PFNAIFLHEARAAAHLSHPHIVTVFDAGLSP-----QGAYIAMELLKGKDLRALLSDGW-
86159869        --------EAEAAARLNHPNVVTLHDFGIHE-----GTPYLILELLRGETLQQRLKRG--
108758255       RFVR----EARVVNQIRHPNIVDVYDFGLMP----DGSPYYVMELLTGRTLSQVVQERGR
108763488       RLLA----EARAVNAIRHRGIIDIFGFGQVP----DGRQYIVMEYLEGQALDAVLTEKNR
108759164       RFYA----EARTVNVVGHPNIVHIFDINEAP----GGIHYFVMEYLEGVPMSHLPRP---
108760510       RFLN----EAKSYARVAHPNAVTLHDFGQDE----EGSLYISMEYVEGDDLKSLLAACGR
116619999       RFLK----ERQLLASLNHPSVVHVIDAGHTQ----DGRPFLVMEHVEG--LPIDQYAAG-
32474038        RFIF----EAQAAGGLHHPNIVPIYGAGIEDG-----IHCYSMPLIRGRSLDEFIYT-DR
```

```
32476260        RFYT----EAKAAAKLKHPNIVAVHQFGRRAG-----HHFFSMQYVEGEDLQKVLAK-GP
32473866        RFER----EAQAAACLNHPSIVQVYEIGDHQG-----THFIAQELIDGSNLKQCLQKSGQ
108758216       RFRR----EAQATAGVKHPNIVEVMDFHDAPDE----PPFLVMELLKGQTLKSLLKKEGP
108759500       RFRR----EAEIASKLGHPNIVEVLDFDSLED----GSPYMVMECLRGMPLSRRLR-KGP
108762243       RFRR----EAEIASRLGHPNIVEVLDYDTLEN----GNPFLVLEYLRGESLQERLA-RGR
116625893       RFER----EAQVLASLNHPNIAIIHGV-EDN--------ALVMELVPGQTLAELIA-SGP
116626707       RFVR----EAKAASGLNHPNIVHIYDI-DESE----GELFIAMEYVAGKTLEQAID-RKG
29831643        RFLR----ERSVLLRLTHPNIVRVRDL-VVEG----DLLALVMDLVDGPDLHRYLRENGP
32476268        RFRR----EMVILERLQHPNIIGYFGG-GSED----GQLFYVMERVDGGTIKDLLETNGA
                        *      : .                           : *  :

13092968        -PMPPHAVVAVLRPVLGGLAAAHRAGLVHRDVKPENILIS-------DDGDVKIADFGLV
111018124       -PMPPHAVAAVAGPVLDALAVAHRAGLVHRDVKPENILIS-------DGGEVKIADFGLV
19553377        -PMPPHAAVGVMRGVLTGLAAAHRAGMVHRDIKPDNVLIN-------SDHQVKLSDFGLV
23493912        -PMPPHAAIGVMRGVLTGLTAAHRAGMVHRDIKPDNVLIT-------RDHRVKLSDFGLV
38234190        -PMPPHAAVAVMRSVLTGLSVAHSAGMVHRDIKPDNVLIN-------ADHQVKLADFGLV
91787132        -LYTLAQTLGIMGDLLSALDYAHRQSVVHRDIKPANLLIQ-------GNGHVKLTDFGVA
94971484        -RLEERYALTIVKELAEALAYAHEKGVVHRDIKPANILIT-------EDGNPKIADFGVA
111019572       -PMDASRAVVIMEQIASALDAAHADGLVHRDVKPENILVT-------ANDFAYLVDFGIA
126435989       -PLDAKRAASIIAQVAAALDAAHADGLVHRDVKPENVLLN-------PDDFAYLVDFGIA
21225517        HPLPVADVVDIAEQVASALAYTHEQGIVHRDLKPANIVRT-------ADGTVKICDFGIA
29827806        HPLPVADVVEIAEQVAAALAYTHQQGIVHRDLKPANIMRL-------ADGTVKICDFGIA
86743110        -RLSPERTLDILGQAADALQAAHALGVVHRDVKPGNLLLR-------PDGAVKVTDFGIA
124268135       -RPTVGQAVLIIRRVADALAYAHSKGIVHRDIKPANIFMV-------GRTQPKVLDFGIA
86159869        -RLQPEEAVRIARDVASGLVHAHSRGVLHRDLKPGNVFLT-------EAGGVKLLDFGLA
108758255       --LSSTRALAYLEPVCGALEAAHRAGVVHRDLKASNILVVEEGE---RPR-VKLLDFGIA
108763488       --LPVQEALALLDEVLAALAAAHGAGVVHRDLKPSNIFLVQQPD---GSRYVKVLDFGLA
108759164       --MVPATLVSLLAQACDALDAAHRCGVVHRDLKPDNLFVVRHAG---EPPSLRVLDFGVA
108760510       --LALDEAVDIVLQVADVLAYAHARQVIHRDLKPENIMVR-QGM---RGWHVKVLDFGIA
116619999       --IAVANRLKLFVRVCDAVSHAHRHLIIHRDLKPQNILVD-STG---Q---PKLLDFGIA
32474038        --PEVEDAIRWALQVANAIDHAHRYGVIHRDIKPSNLIVDQDG-------KIWVTDFGLA
32476260        --LPSRRAAEIVRDVAHAIHHAHSRGVLHRDLKPGNVLIDPSG-------QVHVTDFGLA
32473866        --FTAKEGIEILRCVTEALVVAHAARVTHRDIKPENIMRSGDG-------AIKVTDFGLA
108758216       --LPVERAAAIAHQVANALVVAHQAGVVHRDIKPDNIFLVDTGT---EALHVKLLDFGVA
108759500       --MTLEEVFSCARQMGSALQAAHRAGIVHRDLKPGNVFLVPTEVGGVMMEHVKLLDFGIS
108762243       --LPMEDVVSFTRQMGSALQAAHGAGVIHRDLKPANVFLVPTDSGGVVGERVKLLDFGIS
116625893       --VPLEEALGIARQIAEALEAAHEKGVVHRDLKPANVKVTPEG-------VVKVLDFGLA
116626707       --LTLREALRYAVPMADALAKAHAAGIVHRDFKPSNVMITGER-------TVKVLDFGLA
29831643        --FTPVAAALLTAQVADALAASHADGVVHRDLKPANVLLKQDGG----QMHPMLTDFGIA
32476268        --LAWPVVVDVARQVCSALQCAHNHGVIHRDLKPGNLFLTRD-------AHVKLGDFGIA
                                  :  :*   : ***.*. *:                : ***:

13092968        RAVAAA---------------GITSTSVILGTVAYLSPEQVRDGD--ASPRSDVYSAGIM
111018124       RAAAAS---------------TTTSNSVILGTAAYLSPEQVTSGI--ADTRSDVYSTGVL
19553377        RAAHAG---------------QSQDN-QIVGTVAYLSPEQVEGGE--IGPASDVYSAGIV
23493912        RAASAG---------------QSRDD-KIVGTVAYLSPEQVEGTE--IGPASDVYSAGIV
38234190        RAASAS---------------QATSN-QIVGTVSYLSPEQVSGDD--IGPESDVYSAGIV
91787132        RIQDSSD--------------ATRTRGTMVGTLKYMSPEQVQGRP--IDARADLFAAGIV
94971484        RIDAST----------------MTFHGQLLGTPAYMSPEQLTGGL--VDGRSDLFSLGVI
111019572       HSSSDL---------------RLTTLGSAVGSYAYMAPERFDNAP--VDGRADVYSLACV
126435989       HTGGDP---------------GVTSTGMIVGSSAYMAPERFSGGP--VGPPADVYSLTCL
21225517        RLGHDAGFTA-----------RLTGTGIAMGTPHYMSPEQIGGDE--VDRRSDLYSLGCV
29827806        RLGHDIGFTA-----------RLTGTGIAMGTPHYMSPEQISGSP--VDQRSDLYSFGCV
86743110        RAVDAA---------------PLTATGIMMGTAYYVSPEQASGRP--VTPASDVYSLGVV
124268135       SVAHAR-----------------DSGGAVGGSPHYMAPEQVRNEP--TDRRTDVFSLGVV
86159869        ---RLL-----------------DRASLAGGTPAYMAPEQLRGEP--GDARADVFSAGVV
108758255       KLLHAEPSQE-----------GLTIAGQRLGTAHAMAPEQFRGGP--IGPHTDIYALGVL
108763488       KRGQGPTGRT-----------AQTRTDMVVGTPEYMAPEQARGQE--VGPMTDLYALGVV
108759164       KARRPHPGED------------ETAAGIVLGTPAYMAPEQSAGQP--VDGRADIYALAVT
108760510       RITD---GAT-----------RLTVQGAVAGTPRYMSPEQAMGLD--VDARADVYAVGIV
116619999       KLVN---ETG-----------DATENAEQLLTPNYASPEQFRGEA--QSTATDVYSLGAV
32474038        RCRQGNGSDVN----------GITGSNAVVGTLRYMSPEQSLGKASFVDHRADVYSLGVT
32476260        KHTDADSS--------------VTGSGAAVGTPHYMAPEQALGHSDRVTHHSDIYSLGAI
32473866        RVLSNTDASAV----------DLTRAGLTLGTPRYMSPEQIQGHK--VDGRSDLYSLGVT
```

```
108758216       RLMHEDDATAL-----------GTESGAWVGTPSYMAPEQIRCRP--VDGRADIYSLGAC
108759500       KVIDS--------------QSVHTQGGILLGTPQYMAPEQATGKNGEVDPRTDIFAFGCL
108762243       KVLSS--------------TTVQTQEATIIGTPQYMSPEQAQGKNRDIDARTDVFALGCI
116625893       KAADP--ISNAPAANSPTLTIRATQAGLIMGTAGYMAPEQAAGK--PVDRRADIWSFGVV
116626707       KLMESESATSESAETVPMHEAGQTREGTVVGTAAYMSPEQAEGK--PVDGRSDIFSFGAV
29831643        RLADS---------------PGLTRTHEFVGTPAYVAPESAEGRP-QTS-AVDIYGAGIL
32476268        RDQHS---------------SDLTSQGLTVGTHAYMAPEQITGDE-AISGKADLYALGCV
                                               :    :**              *::.

13092968        TYELLTGHTPFTGDS-ALSIAYQRLEIDVPRASTVITGVPQ-QFDELVARATVRDPSGRY
111018124       LFELLTGRTPFTGDT-SLSVAYQRINQDVPRPGSFIAGVPP-EFDELVAEATHREPSHRF
19553377        LFELLTGTTPFSGED-DLDHAYARLTEVVPAPSSLIDGVPS-LIDELVATATSINPEDRF
23493912        LFELLTGTTPFDGAD-DMDHAYARLTEVVPAPSSLIDGIPS-LVDALVATATALNPEDRF
38234190        MFELLTGTTPFSGDN-QIAHAYARLDSAVPAPSSMIAGIPP-LIDALVASATALRPEERF
91787132        LYQLLTGKRPFDGDT-DFVIIQQIVGHAAEAPTSFNPRLPA-AIDAVVARALAKSPSQRF
94971484        LYTVLMGFRPFQGNG-ASTIGFKVINQHPLPITTFHLDLSK-DTEYVVARAMAKNPRDRY
111019572       LHECLTGAIPFPSNS-ISSAIRAHLAAPAPRPSAVRPDIPA-AFDGVVATGLAKSRDARY
126435989       LYECLTGKPPFETGA-LQQLMSAHMFAPPPRPSIMRRGISR-AFDDVIARGMAKQPGDRF
21225517        LYEMATGVPPFDLGD-AWAILVGHRDTEPEPPRTHRAELPR-YLDRIILDLLAKRPEQRP
29827806        LYEIATGAPPFDLDD-AWAILIGHRDTPPDPPRGHRPELPA-YFEKIILDLLAKLPDERP
86743110        AYECLAGRRPFDDRN-PIVVVMAHQQDTPPP---LPTDIPY-QVRALVDSAMAKDPARRP
124268135       LYELLTGHRAFEGDSLAAITGAVCSFTP-PMADEVSTAVPP-ALASIAARAMAKRPEDRP
86159869        LWQMLTGELPFP-----VVDGRSTVLDPGPPPRLPLEDAPP-ALASLLTAALSQAPTGRP
108758255       LHQLITGRYPFQCED-RMELERLHLEAPAPRPS-AIAAVSP-AVDAVVLRCLEKDGSRRF
108763488       TFEIVTGRLPFVGSS-PVDLLMKHVEARPPRPSEFVSDLPP-ALDAFILQMLTKDPETRP
108759164       AYYLSTGQLPFERGQ-MVELALGTGPVGAPPPHLLVPGVPP-ALSEVLLRALSRRCEDRY
108760510       LFELLTGQQPFDGNS-VSEIMQKQVHQPMPRLAQVAADLQYPTVDAVIQKATAKRREERY
116619999       LYKLLTGSAPREHAR-TGAMPEPAAPS------RLNGDVPR-DVDFVVAKALRPEPEHRY
32474038        LYEMLCG-------------ERFDGSDPASVRRR--NPKVSRDLETVLIKSLAKEPAERY
32476260        LFAAITSRPPIVGDTVMQTLLKVAHQPPPTLRSV--CPEAESDLEVIVAKCLEKQPKDRY
32473866        LYHLLAGQPPFDAEEPLALAVKHLHEMPQPLDHARGSSDLPPWLVATIMRLLRKTPGERF
108758216       LYQMVTGQRPIDVADNVALLSAVMHQVPAPLNGVR--TDVPDGFSQVVARTLEKDPATRY
108759500       VYEMLARRLPFRDNGNLPELIYRIVYDPPEPLEALVPDLPD-HVIAAVEKSLEKRPEDRF
108762243       VYEMMAGKPVFG-SGSLAQMIFRVVYEPPEPLAPLCPEATP-EAISAVMRALAKGVDERY
116625893       LYEMLTGRMLFT-GETISHTLASVLKDR---INFAIPNVPS-PICRLLARCLHRDPKERL
116626707       LYEMLTGRRAFC-GATRMATITSVLRDEPKPVGETRDTVPK-ELERVIARCLRKDPDRRF
29831643        LYELITGRPPFA-GGSALEVLHQHLSAE-PRRPSTVPDP----LWTVIERCLSKNPDRRP
32476268        LFEMLANRKVFA-GENFAQLFEQHLRTKAPTIASIVPDVPP-ELSQVIADCLEKSPDDRP
                                                                          *

13092968        ADAIEMAAQV-------
111018124       ANAEQMGSAL-------
19553377        DDSGEFLSALEDV----
23493912        SDASEFLTAM-------
38234190        ADAAEFLNAL-------
91787132        STAREFNAAL-------
94971484        QNGAAF-----------
111019572       RTAGALAAAARAAL---
126435989       PTAGDLAKA--------
21225517        DDAGELGRRI-------
29827806        HDARELARRI-------
86743110        SSAGAFARSAAGIRRSL
124268135       RSARALSRDLHEWL---
86159869        QTALGLL----------
108758255       GSVTAFLAAL-------
108763488       NSADALRQQL-------
108759164       ATALEFKEALLL-----
108760510       ATMEAFASDLV------
116619999       ASVDEFANDV-------
32474038        SAAGDLAEDL-------
32476260        KTAKNLADDL-------
32473866        ASAMELLDVI-------
108758216       ADSHELAQALEHWI---
108759500       PDVASFI----------
```

```
108762243        PDVSSFI----------
116625893        RDIGEVRIV--------
116626707        QHMEDL-----------
29831643         SAENLARAL--------
32476268         F----------------
```

Cluster No. 71 multiple sequence alignment

```
CLUSTAL 2.0.10 multiple sequence alignment


13881827        YRIERMLGAGGMGTVYLARNPDLPRSEALKVLAAELSRDLDFRARFVREADVAAGLDHPN
31793271        YRIERMLGAGGMGTVYLARNPDLPRSEALKVLAAELSRDLDFRARFVREADVAAGLDHPN
1370255         YRIERMLGAGGMGTVYLARNPDLPRSEALKVLAAELSRDLDFRARFVREADVAAGLDHPN
54022822        YRVIQVLGSGGMGTVYLAQNPILPRRDALKVLSADLSTDDEFRARFEREANLAAGLDHPN
54026639        YTIERLLGRGGMGSVYLARHPRLPRWTALKLLNRELFHDTEIRARFEREADLVAQLDHPN
54026640        YVIERQLGRGGMGSVYLAKHPRLPRMTALKLLNREMFNDKEVRARFEREADLVARLDHPN
54026222        FTVERLLGQGGMGSVYLARHPRLGKLTALKLLNPELFTDRQVRARFDREADLAAQLDHPG
54026641        YEIKRVLGQGGMGTVYLARHPRLPRLTALKLLARELYTDAEIRGRFEREADLVAQLDHPN
111017527       YTIQRVLGVGGMGAVYLARDPHLPRNTALKLLDRSLTTDDCFRSRFELEADHAARLEHPN
111025322       YTIERVLGVGGMGTVYAAAHPRLPRRIALKVLHPALAEDDDARSRFELEADHAARLEHPN
111026929       YTIERVLGTGGMGTVYLARHGSLPRGVAVKVLDG--TADDYVRARFVREAEHAARLEHPN
54024394        FRIERTLGSGGMGTVYLARHPRLPRSVALKVLDAAAGADPEFRARFAREADIAVRLDHPH
54024395        YRIVRRLGAGGMGTVYLAEHPRLPRRDAVKVLDPELGADPGYRARFEREAELAARLEHPN
2131011         FTIVRQLGSGGMGEVYLARHPRLPRQDALKVLRADVSADGEYRARFNREADAAASLWHPH
13881433        FTIVRQLGSGGMGEVYLARHPRLPRQDALKVLRADVSADGEYRARFNREADAAASLWHPH
31792934        FTIVRQLGSGGMGEVYLARHPRLPRQDALKVLRADVSADGEYRARFNREADAAASLWHPH
120406577       YTIVRMLGSGGMGKVYLAKHPRLPRYDALKVLSTTVCADSEYRERFHREADIAATLWHPH
145221764       YTISRMLGAGGMGEVYLAKHPRLPRYDALKVLSASVSTDSEYRERFNREADIAASLWHPH
126437951       YTIMRSLGHGGMGEVYLAQHPRLPRQDALKVLTAAVSADDEYRQRFQREADIAATLWHPH
145224013       YTIVRLLGSGGMGEVYLAQHPRLPRRDALKVLPAAVSADVEYRKRFEREADIAATLWHPH
126437262       YTIVRLVGTGGMGEVYLAQHPRLPRQDALKVLPASFSADDEYRHRFSREADLAAALWHPH
126433399       YTIRRMLGSGGMGEVYLAQHPRLPREDALKVLKSSISADPDFVERFNREADLAAKLWHPH
41407430        YTILRQLGAGGMAEVYLALHPRLPRRDVIKVLAEAVTVDPEFRERFNREADLAATLWHPH
3261603         YTVVRMLGCSAMGEVYLVQHPGFPGWQALKVLSPAMAADDEFRRRFQRETEVAARLFHPH
2078052         YQLLRLLGRGGMGEVYEAEDTRKHRVVALKLISPQYSDNAVFRARMQREADTAGRLTEPH
13880519        YQLLRLLGRGGMGEVYEAEDTRKHRVVALKLISPQYSDNAVFRARMQREADTAGRLTEPH
41409485        YRLVRLLRQGGMGEVYEAEDTRKHRLVALKLISQQFSGNPEFSARLQREADIAGRLTEPH
3261596         YHLKRLLGRGGMGEVYEAEHTVKEWTVAVKLMTAEFSKDPVFRERMKREARIAGRLQEPH
13880901        YHLKRLLGRGGMGEVYEAEHTVKEWTVAVKLMTAEFSKDPVFRERMKREARIAGRLQEPH
31792458        YHLKRLLGRGGMGEVYEAEHTVKEWTVAVKLMTAEFSKDPVFRERMKREARIAGRLQEPH
41408602        YHLKRLLGRGGMGEVYEAEHTVKEWTVAVKLLNESFSSDPVFRERMKREARTAGRLQEPH
111026927       YHLKRLLGKGGMGEVYEAYDLDKDRTVALKLLPDALADDPIFRERLRRESHAAARLQEPH
120405645       YRLRRLLGKGGMGEVYEAEDTVKDRVVALKLLPEAVSHDPVFRKRLQREAHAAGRLQEPH
145222614       YRLQRLLGKGGMGEVYEAYDTVKDRVVALKLLPESASHDPVFRKRLQREAHAAGRLQEPH
134097872       YRVEGLIARGGMGEVLRAYDTRHDRIVALKVLGSGVAADPEYRERFKREALAAARLREPH
134099731       YRVEGLIARGGMGEILRAYDTRHDRVVALKLLAENLAADDEFRERFKREAHAAARLREPH
120402081       YELQSVIGVGGMGEVYRAYDTARERMVAIKLLRPEMAADHSFQERFRRESRVAARLQEPH
                : :   :  ..*. :  . .       .:*::      :     *:  *:  .  * .*

13881827        IVAVHQRGQFEGRLWIAMQFVDG-GNAEDALR--AATMTTARAVYVIGEVAKALDYAHQQ
31793271        IVAVHQRGQFEGRLWIAMQFVDG-GNAEDALR--AATMTTARAVYVIGEVAKALDYAHQQ
1370255         IVAVHQRGQFEGRLWIAMQFVDG-GNAEDALR--AATMTTARAVYVIGEVAKALDYAHQQ
54022822        IVAVYNRGEEDGQLWIAMQYVDGTDAAEEAAKG-PSVMTPQRALRIVSEVGKGLDYAHRR
54026639        IVTVFDRGVDDEQLWISMRYIDG---TDAAALD-VATLPPWRAVQIVGETAKALDFAHAR
54026640        IVTVYDRGLEDEQLWISMQYIDG---VDAASVD-PQTLPPARAVQIVKETADALDYAHSM
54026222        IVAVYDRGSENHQLWISMQYVDG---VDAASVN-PLTLPPERAVQIIEGVADALDYAHGR
54026641        IVTVYDRGAEDEQLWISMQFVPG---SDAAAAD-IDVLAPGRAVQIIGEVAAALDFAHAN
111017527       IVSVFDRGREANQHWIAMQYVAG--TDAAVALR-EGPMDPPRAVHIVAETAKALDYAHEN
111025322       IVAVYDRGREGDRLWIAMQYVDG--TDAETARE-GAPLDPARAVRIITETAKALDYAHEA
111026929       IVTVYDRGCEGDRLWIAMQYVAG--TDAATLLR-RGPLPPDLAVHIVAEIGQALDFAHEH
54024394        VVEIYDRGAEDERLWISMRYVAGPDAARLIR-E-RGRLPARRAVGLVAQAAAGLDAAHRR
54024395        VVAVYDRGREGEFLWIAMRYVDGVDAGELVAAE-PAGLPAERAVGIVAAAARGLDAAHRR
2131011         IVAVHDRGEFDGQLWIDMDFVDGTDTVSLLRDRYPNGMPGPEVTEIITAVAEALDYAHER
13881433        IVAVHDRGEFDGQLWIDMDFVDGTDTVSLLRDRYPNGMPGPEVTEIITAVAEALDYAHER
```

```
31792934        IVAVHDRGEFDGQLWIDMDFVDGTDTVSLLRDRYPNGMPGPEVTEIITAVAEALDYAHER
120406577       IVAVHDRGEYEGRLWISMDHVEGTDAAWLLAERYPHGMPPALVIRIVTAVGEALDYAHQR
145221764       IVQVHDRGEFEGRLWISMDHVEGTDAARLLAERYPDGMPPALVARLVTAVGEALDYAHQR
126437951       IVSVHDRGDFDGLLWISMDFVQGTDAARLLAERYPNGMPPDVVVRIITAVASALDHAHER
145224013       IVGVHDRGEDAGRIWISMDYVEGADAAHLLAEDYRTGLPVAEVAQIVTAVADALDYAHDR
126437262       IVGVHDRGEYDGRLWISMDFVDGHDAARLLVDRYPNGLPAADVIEIVTAVADALDYAHQR
126433399       IVGIHDRGRYRGRLWISMDFVDGTDVGRLLQQKYPDGMPADDALEIVEAVASALDYAHSR
41407430        IVGVHDRGEFNGHLWISMDYVEGTDASRLVKESYPDGMPLDEVSAIVQAVAGALDYAHAR
3261603         ILEVHDRGEFDGQLWIAMDYVDGIDATQHMADRFPAVLPVGEVLAIVTAVAGALDYAHQR
2078052         IVPIHDYGEINGQFFVEMRMIDGTSLRALLKQY--GPLTPARAVAIVRQIAAALDAAHAN
13880519        IVPIHDYGEINGQFFVEMRMIDGTSLRALLKQY--GPLTPARAVAIVRQIAAALDAAHAN
41409485        VVPIHDYGEIDGRFFVEMRLVDGIDLGSLLHRE--GPLAPPRAIAIIRQVAAALDAAHAA
3261596         VVPIHDYGEVDGQMFLEMRLVEGTDLDSVLKRF--GPLTPPRAVAIITQIASALDAAHAD
13880901        VVPIHDYGEVDGQMFLEMRLVEGTDLDSVLKRF--GPLTPPRAVAIITQIASALDAAHAD
31792458        VVPIHDYGEVDGQMFLEMRLVEGTDLDSVLKRF--GPLTPPRAVAIITQIASALDAAHAD
41408602        VVPVHDYGEIDGQMFLEMRLVEGTDLDSVLKRF--GPLPPPRAVAIITQIASALDAAHAA
111026927       VVPIHDYGEVNGVLYIDMRLVQGTDLRSLLTRY--GPLAPPRAVAIISQIAAALDAAHAD
120405645       VVPIHDYGEVDGLLFVDMRLIDGTDLRKLLKEQ--GPMTPARATAIVAQVASALDAAHQN
145222614       VVPIHDYGEIDGLLFVDMRMIDGVDLRRILKEQ--GAMSPARATAIVRQIASALDAAHRA
134097872       VIPIHSFGEIDGRLYLDMRLIEGQDVSRLLAAH--GPMPPADAAEVVHQIAQALDAAHEE
134099731       VIPIHAYGEIDGRLYLDMRLVEGGDVGSLLASR--GPMRPAEAVGVIEQVARALDAAHAE
120402081       VIPVHDFGEIDGVLYIDMRLVEGASLKDVLRAE--GALPPARAVSILRQVAAALDAAHAN
                :: :.  *      :: *  : *              :    .  ::   . .** **

13881827        GVIHRDIKPANFLLSRAAGGDE----RVLLSDFGIARALGDTG---LTSTGSVLATLAYA
31793271        GVIHRDIKPANFLLSRAAGGDE----RVLLSDFGIARALGDTG---LTSTGSVLATLAYA
1370255         GVIHRDIKPANFLLSRAAGGDE----RVLLSDFGIARALGDTG---LTSTGSVLATLAYA
54022822        GLLHRDVKPANFLLSAVDGDDEE---RVLLTDFGVAKSAEDGQD--LTATGNFMATVAYA
54026639        GVLHRDVKPANILLERAEPGVGE---RVYLTDFGIARLRDDTGH--LTRTGTFTATLAFA
54026640        GVLHRDVKPANILLARSTGGRGE---RVYLTDFGIARLRDDTGH--LTQTGTFTATLAYA
54026222        GVLHRDVKPGNILLARASAGQGE---RVFLSDFGIARLREDTTH--LTQTGMFTATLAYA
54026641        GVLHRDVKPANILLAKAPIGQPE---RVLLTDFGIAGVRDADTT--LASGDTITATLAYA
111017527       GVLHRDVKPANILLGMSGAGQPE---RVLLTDFGIAKALDETQH--LTRTGSLVATLQYS
111025322       GVLHRDVKPANILLEHPRPGHPGDPGRVLLADFGIAKALEHTQH--LTKTGMLVASLQYA
111026929       GILHRDVKPANVLLAG-RPAAGGGVWRVLLTDFGIAKNLDETRR--LTRTGMLVATLVYA
54024394        GLLHRDVKPANLLVAADDDGGD----HVFVADFGIARSRDDAVR--LTGAGALPATLGYV
54024395        GLLHRDVKPANILVSTDDDGAD----VVRLTDFGIARSLDAAA----TTSGSVLASFAYA
2131011         RLLHRDVKPANILIANPDSPDR----RIMLADFGIAGWVDDPSG--LTATNMTVGTVSYA
13881433        RLLHRDVKPANILIANPDSPDR----RIMLADFGIAGWVDDPSG--LTATNMTVGTVSYA
31792934        RLLHRDVKPANILIANPDSPDR----RIMLADFGIAGWVDDPSG--LTATNMTVGTVSYA
120406577       GLLHRDVKPANILIADPETENE----RIMLADFGIARRVGEVST--LTGTSMTVGTVAYS
145221764       GLLHRDVKPANILIADPETENE----RIMLADFGIARRVGEVSA--LTGTNMTVGTVAYS
126437951       GLYHRDVKPANILIANPGSPDE----RAMLADFGIARQAGDASG--LTGTNMTVGTVAYA
145224013       HLLHRDVKPANILIARPDSNTR----RIMLADFGIARWDNDISG--LTATNMTVGTVSYA
126437262       QLLHRDVKPANILITGPDRARR----RILLADFGIARHTEDNTG--LTSSNIAVGSMSYS
126433399       GLLHRDVKPANILIADVENDER----RILLGDFGVARDLADDAGGGLTQTNMTVGTAAYA
41407430        GLLHRDVKPANILLTHPEAGER----RILLADFGVARHLGDISG--ITETNVAVGTVAYA
3261603         GLLHRDVNPANVVLTSQSAGDQ----RILLADFGIASQP------------------SYP
2078052         GVTHRDVKPENILVTASD--------FAYLVDFGIARAASDPG---LTQTGTAVGTYNYM
13880519        GVTHRDVKPENILVTASD--------FAYLVDFGIARAASDPG---LTQTGTAVGTYNYM
41409485        GVTHRDVTPGNILVTPSD--------FAYLADFGIARAASDPG---LTQVGTAIGTYYYM
3261596         GVMHRDVKPQNILITRDD--------FAYLVDFGIASATTDEK---LTQLGTAVGTWKYM
13880901        GVMHRDVKPQNILITRDD--------FAYLVDFGIASATTDEK---LTQLGTAVGTWKYM
31792458        GVMHRDVKPQNILITRDD--------FAYLVDFGIASATTDEK---LTQLGTAVGTWKYM
41408602        GVMHRDVKPQNILVTRDD--------FAYLVDFGIASATTDEK---LTQLGTAVGTWKYM
111026927       KLVHRDVKPENVLVTRED--------FAYLVDFGIANTATDEK---LTTLGTAVGTYDYM
120405645       GIMHRDVKPENILINRDD--------FAYLVDFGIANAVTDEA---LTELGTAVGTYAYM
145222614       GIMHRDVKPENILVTRDD--------FAYLVDFGIANAATDES---LTELGTAVGTYAYM
134097872       GLVHRDVKPSNIIIGRGG--------FAYLVDFGIAHWAGNRTS--LTTTGIAVGTLDYM
134099731       GLVHRDVKPSNILLGDGG--------FAYLVDFGIAHSVQAGT---LTSTGFTVGTLDYM
120402081       GLVHRDIKPENVLLTPDD--------FAYLVDFGIAHGGGEAS---VTSTGLVVGSSAYM
                 : ***:.* *.::               : ***:*                      :

13881827        APEVLAG--QGFDGRADLYSLGCALFRLLTGEAPFAAGAGAAVAVVAGHLHQPPPTVSDR
```

```
31793271        APEVLAG--QGFDGRADLYSLGCALFRLLTGEAPFAAGAGAAVAVVAGHLHQPPPTVSDR
1370255         APEVLAG--QGFDGRADLYSLGCALFRLLTGEAPFAAGAGAAVAVVAGHLHQPPPTVSDR
54022822        SPEQLLG--ERLDHRSDIYSLGCSFYRMLTGQNPFPS--TVPAVVMMGHLHEAPPKPSAA
54026639        SPEQLSG--APLDHRSDQYSLACTLFRLLTGTVPFAA--DNPVAVIGGHLHRPPPAAAEL
54026640        SPEQLTG--ASLDHRSDQYSLACSLFWLFTGSGPFAA--TNPAQVIQGHLQAPPPALSSA
54026222        SPEQMTG--APLGNRSDQYSLACALYWLLVGVGPFDA--ANPADIIHGHLNLAPVPVSVR
54026641        APEQLSG--HTLDHRADQYSLACTLFWLLTGSVPFPG--ANPAVVLNGHLFGPPPSARAL
111017527       APEAFQG--IPLDLRADVYSLGCTLFCLLTGHPPFTG--ST-QEVMRGHLCGPVPRISAV
111025322       APEQFAS--IPLDARADVYSLGCTLFRLLTGQQPYPG--STLAQLMAGHLNSPIPRPAAL
111026929       APEALVAG-VELDPRADVYSLGCMLFQLLTGRLPYSG--SA-AAVVDAHLNQPIPRPTVL
54024394        APEQIDG--REPDHRSDLYSLGVTLYQMLTGALPFTG--TTPAELLRAHLLEPPPPVTRR
54024395        APETFAG--GPLDARTDVYALGCTLYEMLTGAVPFAR--RSPAAAMQAHLYEPPPRPSAT
2131011         APEQLMG--NELDGRADQYALAATAFHLLTGSPPFQH--ANPAVVISQHLSASPPAIGDR
13881433        APEQLMG--NELDGRADQYALAATAFHLLTGSPPFQH--ANPAVVISQHLSASPPAIGDR
31792934        APEQLMG--NELDGRADQYALAATAFHLLTGSPPFQH--ANPAVVISQHLSASPPAIGDR
120406577       APEQLTAD-EHIDGRADQYALAATAFQLLTGSPPFQH--SNPAIVISQHLTAQPPSISVH
145221764       APEQLTAD-EAIDGRADQYALAATAFQLLTGKAPFQH--SNPAIVISQHLTAQPPSIAVH
126437951       APEQLRG--DHIDGRADQYALAATAYQLLTGTPPFTH--TNPAVVISAHLTSDPPVIGDV
145224013       APEQLMG--QDLDGRADQYALAATAFHLLTGSPPFSH--SNPAVVISRHLNSAPATVAAH
126437262       APEQLMG--HPIDGRADQYSLAATAYRLFTGSPPFPH--SNPAVIISHHLNTPPPRLGDT
126433399       APEQLMG--LDVDGRTDQYSLAVTAFHMLTGAPPFQN--SRPTVVVGQHLNTPPPLLADT
41407430        APEQLTG--SPIDGRADQYALAATAFHLLTGAPPFQH--SNPIAVIGQHLHEDPPRLSDF
3261603         APELSAG--ADVDGRADQYALALTAIHLFAGAPPVDR--SHTGPLQ-------PPKLSAF
2078052         APERFTGD--EVTYRADIYALACVLGECLTGAPPYRA--DSVERLIAAHLMDPAPQPSQL
13880519        APERFTGD--EVTYRADIYALACVLGECLTGAPPYRA--DSVERLIAAHLMDPAPQPSQL
41409485        APERFTDD--EVTNSVDIYSLACVLTECLTGVPPYRA--DTVERLVAAHLTKTAPPLSQL
3261596         APERFSND--EVTYRADIYALACVLHECLTGAPPYRA--DSAGTLVSSHLMGPIPQPSAI
13880901        APERFSND--EVTYRADIYALACVLHECLTGAPPYRA--DSAGTLVSSHLMGPIPQPSAI
31792458        APERFSND--EVTYRADIYALACVLHECLTGAPPYRA--DSAGTLVSSHLMGPIPQPSAI
41408602        APERFSDA--EVTYRADIYALACVLFECLTGSAPYRA--DSAGVLVSAHVMDPIPAPSAR
111026927       APERFEGDGSEVTYRADIYSLACVLHECLTGARPYPA--DSIRVKISSHLFEPPPRPSVA
120405645       APERFTNG--EVTHRADVYALTCVLHECLTGAQPFQG--DSVSVVITAHLNDPAPRPSQS
145222614       APERFAAG--EVTHRADVYALTCVLHECLTGTQPFEG--DSVSVVITAHLNQPAPRPSSL
134097872       APERFGDG--PVDHRADVYSLACVFYQCLTGAKPYAG--HTAESLINAHLNRVPPRPSSH
134099731       APERFDDA--PVDHRADVYSLACVLYQCLTGAKPFSG--DTAASLINAHLNHQPPLPSSA
120402081       APERFSGE--RGGPASDVYSLACLLYESLTGRAPFEA--ADVRQVWSAHMFAPPPRPSIM
                :**             * *:*       :.*  *

13881827        VPG-LSAAMDAVIATAMAKDPMRRFTSAGEFAHAAAAALYG-
31793271        VPG-LSAAMDAVIATAMAKDPMRRFTSAGEFAHAAAAALYG-
1370255         VPG-LSAAMDAVIATAMAKDPMRRFTSAGEFAHAAAAALYG-
54022822        RPG-LPPALDAVMAKVLAKNPADRYASCREFVQD--------
54026639        RPG-LPAAIDAVLARALAKDPRDRFATCLEFVDAAWR-----
54026640        RPG-LPYALDGVLAKAMAKRPEDRFDSCSEFAAAAQAAL---
54026222        RPG-LNPALDAVLAAGLAKHPDHRYRTCTEFATAARKAL---
54026641        NPA-LPPALDAVLARAMAKLPADRFASCAEFAGAARHAL---
111017527       RGD-LPPTFDDIVSAALTKRREDRLPSCHALSAG--------
111025322       RAG-LPTGFDAVITRAMAKDREHRYPSCGALAAAAQHAL---
111026929       RPS-LPDAMDPVIRQALAKDRDTRFDSCGALAQAARSAL---
54024394        AAD-LPRALDDVLATALAKDPAERYPDCRALAAAAAAAL---
54024395        HPA-LA-AFDPVIARALAKVPAHRYGSCGELARAATAAL---
2131011         VPE-LT-PLDPVFAKALAKQPKDRYQRCVDFARALGHRLG--
13881433        VPE-LT-PLDPVFAKALAKQPKDRYQRCVDFARALGHRLG--
31792934        VPE-LT-PLDPVFAKALAKQPKDRYQRCVDFARALGHRLG--
120406577       RPE-LS-SLGMAFEKALAKSPADRFDRCVDF-----------
145221764       RPE-LS-GLGAAFQKALAKSPADRYDRCVDFARALSNRSTV-
126437951       RPE-LS-GLGPVFGRALAKSPDKRFERCVDFARALEH-----
145224013       RPE-LA-AVDPVLTRALAKNPADRYPRCADF-----------
126437262       RPE-LR-AFDAAMARALAKDPAARFGSCHDFA----------
126433399       HPE-LA-PLDAAMQRALAKNPDERFDTCTEFARAL-------
41407430        RPE-LA-GLDEVFCQALAKAPEDRFDRCRAFAAAV-------
3261603         RPD-LA-RLDGVLSRALATAPADRFGSCREFADAM-------
2078052         RPGRVPPALDQVIAKGMAKNPAERFMSAGDLAIAAHDALTT-
13880519        RPGRVPPALDQVIAKGMAKNPAERFMSAGDLAIAAHDALTT-
```

```
41409485              RPGAFPPALDRVIAKGMAKRPEDRYRTAGEFAAAAHEALTT-
3261596               RPG-IPKAFDAVVARGMAKKPEDRYASAGDLALAAHEALSD-
13880901              RPG-IPKAFDAVVARGMAKKPEDRYASAGDLALAAHEALSD-
31792458              RPG-IPKAFDAVVARGMAKKPEDRYASAGDLALAAHEALSD-
41408602              RPG-VPKAFDAVIARGMAKKPEDRYASAGDLALAAKEAL---
111026927             RPG-VPAGFDAIIARGMAKSPQDRYATAGDLALAAHDALSAG
120405645             RPG-IPAGLDDVISRGMAKRPEDRYASAGEMARAATAAL---
145222614             RQG-IPAGLDEVIARGMAKRPEDRFASAGELADAATDAL---
134097872             NPA-LA-AFDRVVETGMAKDPRRRFATAGEFARAARQAL---
134099731             RSD-VPREFDRIVARGMAKNPAERFSSAGELARAARQALTTM
120402081             RRG-VSRTFDDVVARGMAKQPHDRYPTAGELARA--------
                          .   ..  .   ::.    *   .  :
```

Cluster No. 72 multiple sequence alignment

```
CLUSTAL 2.0.10 multiple sequence alignment


108757646       WSVVRELGNGGFAVVYLVEKHGLRCALKLARHRDSSGDDKQTHARTLRELSALLLLDHPN
108763095       WSVVREIGNGGFAVVYLVEKHGRRCALKLARHRDSSGDDKQTHARTLRELSALLLLDHPN
108762185       WRVIRELGNGGFAVVFLVEKHGRRSALKVARHRDSSGDEKQTHARTLRELAALLLLDHPN
108763545       WRVIKEIGNGGFAVVFLVEKNGRRSALKLARHRDSSGDDKQTHARTLRELSALLLLGHPN
                * *::*:********:****:* *.***:*********:***********:*****.***

108757646       IVKHRGYGYSEQGNVYLALEYVDGWTLAEWAERKHPTVQEVLHVFDKISAALSYMHGRGV
108763095       IVKHRGYGYSEQGNVYLALEYVEGWTLAEWAERKHPTVQEVLQVFDKISAALSYMHGRGV
108762185       IVKHRGYGYSEHGNVYLALEYIDGWTLAEWAERKHPTVREVLQVFDKLCSALSYMHSRGV
108763545       IVKHRGYGYSEHGNVYLALEYIDGWTLAEWAERKHPTVREVLQVFDKLCSALSYMHSRGV
                ***********:*********::***************:***:****:.:******.***

108757646       LHRDLKLSNVLIRKSDGEPVIIDFSCASYSLAEELTDWGLPPGTDRFRAPEQFTWLREHK
108763095       LHRDLKLSNVLIRKSDGEPVIIDFSCASYSLAEELTDWGLPPGTDRFRAPEQFAWLREHK
108762185       LHRDLKLSNVLIRKSDGEPIIIDFSCANYSLAEELTDAGLPPGTDRFRAPEQFQWLREHK
108763545       LHRDLKLSNVLIRKSDGEPIIIDFSCANYSLAEELTDAGLPPGTDRFRAPEQFKWLREHK
                *******************:*******.********* *************** ******

108757646       AEQRAKYAFQVADEIFAVGAMLYELLTDPRPTEVQARVTLNSTVMKPPPARALNVRVPEA
108763095       AEQRAKYAFRVADEIFAVGAMLYELLTDPRPTEVQARVTLNSTVMKPPPARALNVRVPEA
108762185       AEHRAKYAFQVADEIFAIGAMLYELLTDPRPTEIQARFSLNSTVALPPPARALNVRVPEA
108763545       AEHRAKYAFRVADEIFAVGAMLYELLTDPRPTEIQARFSLNSTVILPPPARALNGRVPEA
                **:******:*******:***************:***.:*****  ******** *****

108757646       LNDLVDCILSREPARRPVDTEALRRELG
108763095       LNDLVDCILSRDPAKRPVDSEALRRELG
108762185       LNDLVASVLSRDPAKRPVDTEALRRELG
108763545       LNDLVASILSREPAKRPVDTEALRRELG
                ***** .:***:**:****:********
```
